# Effectiveness of non-pharmaceutical interventions for COVID-19 in USA

**DOI:** 10.1038/s41598-024-71984-1

**Published:** 2024-09-13

**Authors:** Yuhang Liu, Weihao Wang, Weng-Kee Wong, Wei Zhu

**Affiliations:** 1 Department of Applied Mathematics and Statistics, State University of New York at Stony Brook, Stony Brook, NY 11794-3600 USA; 2 Department of Biostatistics, University of California at Los Angeles, Los Angeles, CA 90095-1772 USA

**Keywords:** Public health, Statistics

## Abstract

Worldwide, governments imposed non-pharmaceutical interventions (NPIs) during the COVID-19 pandemic to contain the pandemic more effectively. We examined the effectiveness of individual NPIs in the United States during the first wave of the pandemic. Three types of analyses were performed. First, a prototypical Bayesian hierarchical model was employed to gauge the effectiveness of five NPIs and they are gathering restriction, restaurant capacity restriction, business closure, school closure, and stay-at-home order in the 42 states with over 100 deaths by the end of the wave. Second, we examined the effectiveness of the face mask mandate, the sixth and most controversial NPI by counterfactual modeling, which is a variant of the prototypical Bayesian hierarchical model allowing us to answer the question of what if the state had imposed the mandate or not. The third analysis used an advanced Bayesian hierarchical model to evaluate the effectiveness of all six NPIs in all 50 states and the District of Columbia, and thereby provide a full-scale estimation of the effectiveness of NPIs and the relative effectiveness of each NPI in the entire United States. Our results have enhanced the collective knowledge on the general effectiveness of NPIs in arresting the spread of COVID-19.

## Introduction

The COVID-19 pandemic is a global pandemic of the Severe Acute Respiratory Syndrome Coronavirus 2 (SARS-CoV-2)^1^. The World Health Organization (WHO) declared a public health emergency for COVID-19 on January 30, 2020, and declared COVID-19 a pandemic on March 11, 2020^2^,^3^. Even though its origin is still under investigation, many early reported cases of COVID-19 occurred in December 2019 in Wuhan City, Hubei Province, China^4^. Human-to-human transmission had quickly spread worldwide, and by the end of January 2020, regions, and countries outside of China, e.g., Europe, South Asia, and the United States, had started to see confirmed cases^5^. On January 31, 2020, the United States declared the coronavirus outbreak a public health emergency^6^. On March 13, 2020, the United States declared the outbreak a national emergency—all 50 US states, the District of Columbia, and five territories declared a state of emergency due to COVID-19^7^.

With the exponential growth of confirmed cases, governments tried to utilize all resources to contain the pandemic. In addition to exploring many medical interventions immediately, non-pharmaceutical interventions (NPIs) have also been employed to reduce the infection within the population. The infection rate can be measured by the reproduction number, which is the expected number of cases directly generated by one case in a population, and previous studies have shown that NPIs were effective in reducing the reproduction number^8–15^. Supplementary Table 1 contains a summary of methods used to evaluate factors that contribute significantly to the success of NPIs in various ways, and how NPIs differ significantly from one another in their effectiveness by outcomes. The outcomes include weekly average growth rate of infection of mobility rates, or death rates. Supplementary Table 2 describes methods used to estimate the joint effectiveness of NPI portfolios using the reproduction number. However, to better assist the government with policy making decisions and to equip the population with knowledge and understanding, it is important to quantify the effects of individual interventions so that only the most effective NPIs are strictly imposed. This is important especially when the pandemic has a long-time span and fatigue with NPIs sets in and there are serious consequences on the economy, including rise in mental health and social costs. To this end, Supplementary Table 3 presents a summary of methods used to estimate the effectiveness of individual NPIs.

Many studies for estimating the individual NPI effectiveness were done in Europe. Banholzer et al.^16^ analyzed the effectiveness of NPIs in 16 European countries and 4 non-European countries and concluded that issuing venue closure and gathering bans were able to reduce the percentage of new cases. Brauner et al.^17^ studied the effects of interventions in 34 European countries and 7 non-European countries and found that closing all educational institutions, limiting gatherings to ten people or fewer, and closing face to face businesses would reduce the transmission considerably. Similarly, Flaxman et al.^18^ estimated the effectiveness of NPIs in 11 European countries and concluded that lockdowns have had a large effect on reducing transmissions. Supplementary Table 4 contains a detailed summary of 24 studies on use of NPIs in arresting the pandemic in various ways and some of them may be still ongoing^19^.

Interestingly, but not too surprising, people from different regions and countries have different cultural backgrounds and social ideologies and may react differently to various NPIs. Some of the completed studies aimed at studying the effectiveness of NPIs and based in the United States are summarized in Supplementary Table 5. The purpose of this paper is to estimate the effectiveness of NPIs in the U.S. The NPIs of interest are gathering size restrictions, business closure, restaurant capacity limit, school closure, and the stay-at-home order; the sixth one is the face mask mandate, which is the most controversial NPI and opposition to the mandate varies substantially by states.

We analyzed the first wave of the pandemic in the US which is from February 1 to June 15, 2020. In the first analysis, we used a prototypical model to estimate the effectiveness of the 5 NPIs and in the second analysis, we used a counterfactual model to estimate the effectiveness of the face mask mandate. These two analyses covered 42 states (Supplementary Fig. 1), where each state had more than 100 deaths by the end of the study period. Further, 14 states (Supplementary Fig. 2, Green) initiated face mask mandates during the study period and the rest of the 28 states (Supplementary Fig. 2, Pink) did not. In the third analysis, we used an advanced model to estimate the effectiveness of all 6 NPIs for all 50 states in the United States and the District of Columbia. The last analysis distinguishes itself from the first two by employing a more advanced model that uses observed daily new infection counts rather than death counts. This approach allows us to include data from states with low death counts, thereby eliminating the bias that their exclusion might have introduced in our study. Supplementary Table 6 lists the states’ demographics data and the basic reproduction number before NPIs were implemented. For each of the 5 NPIs imposed in the US, fewer than 5% of the states didn’t introduce the intervention throughout the study period.

Bayesian hierarchical models are hierarchical models analyzed using Bayesian approaches. A hierarchical model is one that is written modularly, or in terms of sub-models. Rather than using classical statistics which focuses on frequencies of events, Bayesian approaches assume that probabilities of interest are expressions of prior belief. The Bayes’ Theorem integrates the sub-models with the observed data and accounts for all the uncertainties that are present, and the result of the integration is the updated probability estimate. We have employed a Bayesian hierarchical model, which is particularly well-suited to our dataset and research objectives. This approach allows us to establish a dynamic link between the time-varying reproduction number, the number of new infections, and the number of deaths. The hierarchical nature of the model also enables us to account for the variation in policy responses and epidemic characteristics across different states, providing a robust framework for our analyses. Additionally, the Bayesian framework enables us to incorporate prior information and uncertainty in a principled manner, which is crucial when dealing with the limited and noisy data typically encountered in real-time during a pandemic.

The first analysis used in estimating the effectiveness of 5 commonly imposed NPIs was based on the prototypical Bayesian hierarchical modeling framework^18^. Since the response to COVID-19 was far less coordinated within the US, different states implemented NPIs in different orders and on different days, which renders the effect of each individual NPI identifiable.

Face mask mandate policy has created increasing divisions between different cultural backgrounds globally. For this reason, only a limited number of regions and countries imposed face mask mandate, and thus only a limited number of studies provide estimates of the effectiveness of face mask mandate. Supplementary Table 7 displays a summary of previous studies on estimating the effectiveness of mask mandate. The assessment of face mask mandates' effectiveness is challenged by the fact that, during the study period, 28 states did not implement such mandates, rendering a uniform model application infeasible. This issue echoes the 2020 findings^20^ in Soltesz's paper, which showed that the effects of NPIs on the pandemic's trajectory can be substantially influenced when a model is constrained from providing special adjustments based on the last NPI parameter unique to each state. Nevertheless, our approach circumvents this obstacle by employing a modified Bayesian hierarchical model that incorporates a counterfactual scenario, enabling us to isolate and evaluate the impact of face mask mandates independently. The counterfactual model allows preservations of the specific characteristics of each group while transposing the influence of contact pattern change on disease transmission. Specifically, our second analysis examined how the pandemic situation would be in the 28 non-face mask mandate states had they initiated the mask mandate policy, and conversely, what would happen in the 14 states with face mask mandate had they not implemented such policy.

The analysis using the prototypical Bayesian hierarchical model and the counterfactual Bayesian hierarchical model required a backward calculation from the observed number of deaths to estimate the transmission rates that occurred from several weeks ago. To avoid possible model biasing caused by the low death count, we excluded 9 outlier states in the United States with death counts fewer than 100 by the end of the study period. However, removing about 18% of the states may not accurately reflect the situation in the entire United States. Therefore, it is helpful to have a model that can incorporate data from all 50 states and the District of Columbia in the analysis and verify whether results drawn from the prototypical Bayesian hierarchical model and the counterfactual Bayesian hierarchical model still hold. To this end, in our third analysis, we used the advanced Bayesian hierarchical model developed by Banholzer et al.^16^ to overcome the challenge. In Supplementary Table 8, we compare the pros and cons of the three Bayesian hierarchical models, and Supplementary Table 9 lists previous COVID-19 studies that used these models.

## Methods

### Data collection

NPIs are also known as community mitigation strategies. They are actions apart from vaccination and medication that the community can take to reduce contacts within the population and help slow down the spread of disease^22^.We categorized the major NPIs implemented in the US based on the nature of the intervention. In this study, we estimated the effectiveness of the following NPIs: (1) gathering restriction, where the state set a size limit on gatherings; (2) business closure, where the states closed the operations of most face-to-face businesses unless they are designated as essential businesses; (3) restaurant capacity restriction, where the state issued capacity restrictions in restaurants; (4) school closure, where the state closed schools statewide; (5) stay-at-home order, where the state issued an order for the general public to stay at home and exemptions were only granted for grocery shopping, going to hospital, etc. and (6) face mask mandate, which specifically refers to the official requirement for face mask usage and enforced by state authorities across the entire state, as opposed to merely suggesting or encouraging the voluntary use of face masks.

The window of time for all our analyses is between February 1 and June 15, 2020, corresponding to the first wave of the COVID-19 pandemic in the US. Since some states started lifting NPIs in late June 2020, we chose June 15, 2020, to be the end date of our study period to avoid possible distribution shifts. For instance, non-essential businesses usually resumed with precautionary measures (e.g., allowing a small percentage of the original customer capacity, wearing face coverings, maintaining social distancing, etc.), thus the number of contacts after reopening would have been different from that before. The start date of each NPI for each state has been gathered from public sources. The daily confirmed cases and death data were obtained from the Johns Hopkins University CSSE COVID-19 Dashboard^23^.

### Modeling

In the first analysis, the effectiveness of five commonly imposed NPIs in the US was estimated using the prototypical Bayesian hierarchical model developed by Flaxman et al.^18^. One cannot, however, analyze the effectiveness of the face mask mandate using the same prototypical Bayesian hierarchical model because 28 states did not impose the face mask mandate during the study period. Instead, the second analysis used to estimate the effectiveness of face mask mandates was examined using the counterfactual model, a variant of the prototypical Bayesian hierarchical model, developed by Mishra et al.^21^ The counterfactual model allows preservations of the specific characteristics of each group while transposing the influence of contact pattern change on disease transmission. Specifically, we examined how the pandemic situation would be in the 28 states without-face mask mandate had they initiated the mask mandate policy, and conversely, what would have happened in the 14 states with face mask mandate had they not implemented such policy.

**Fig. 1:**
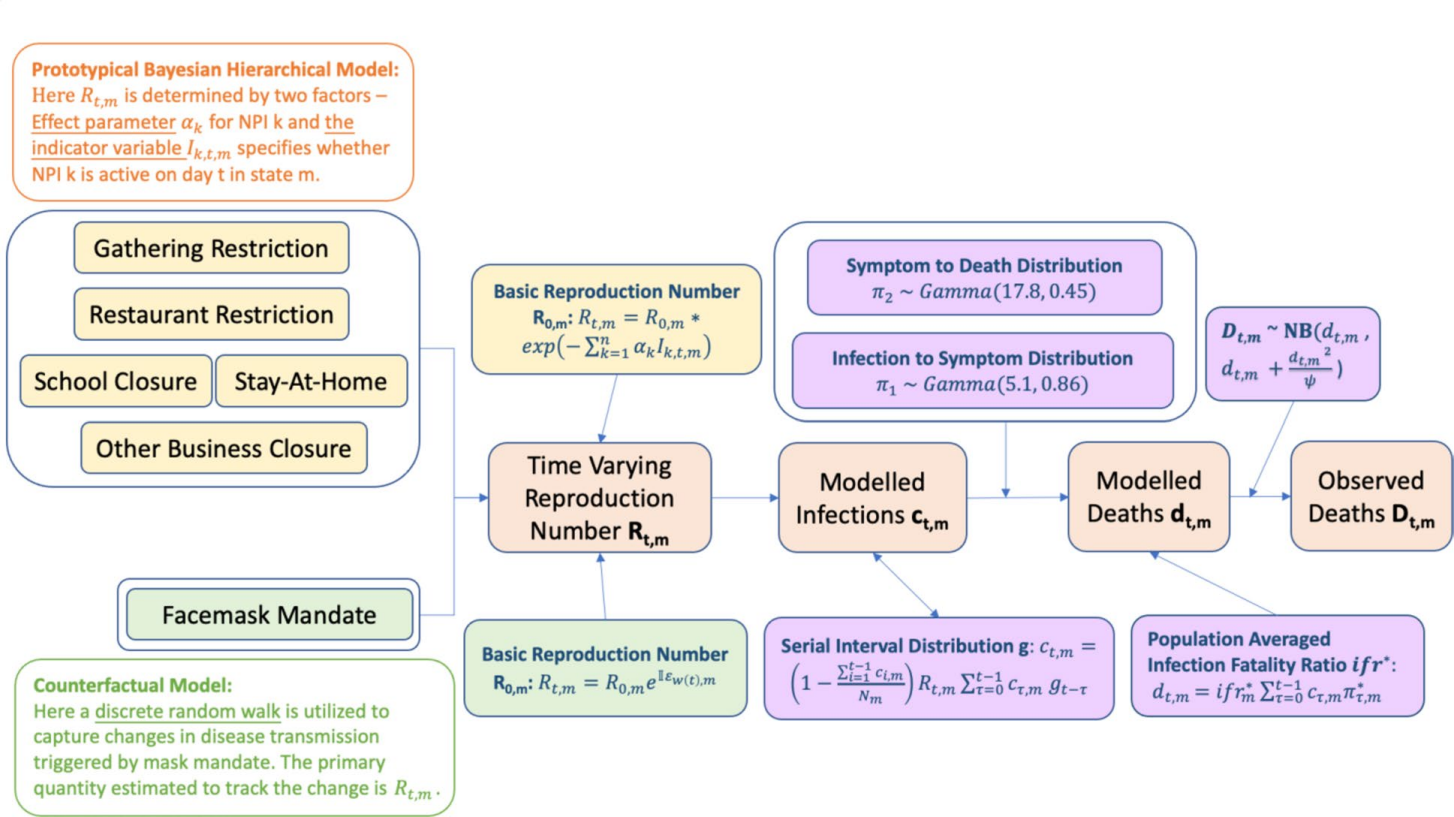
Flow chart of the first two sets of analyses: (1) Analysis 1 uses the prototypical Bayesian hierarchical model. In the first analysis, a piecewise constant function is used to scale ${\text{R}}_{\text{t},\text{m}}$ from the baseline reproduction number ${\text{R}}_{0,\text{m}}$ and the NPI in different states and times. The model sets an indicator variable ${\text{I}}_{\text{k},\text{ t},\text{ m}}$ which equals to 1 for the kth intervention group in state $\text{m}$ at time $\text{t}$, and 0 otherwise. The functional form of ${\text{R}}_{\text{t},\text{m}}$ is given by ${\text{R}}_{{{\text{t}},{\text{m}}}} = {\text{R}}_{{0,{\text{m}}}} *{\text{exp}}\left( { - \sum _{{{\text{k}} = 1}}^{{\text{n}}} \alpha _{{\text{k}}} {\text{I}}_{{{\text{k}},{\text{t}},{\text{m}}}} } \right)$ and the intervention-specific effects α_k_ are shared among all states; (2) Analysis 2 uses the Counterfactual model, where the functional form for the time-varying reproduction number is given by ${\text{R}}_{\text{t},\text{m}}={\text{e}}^{{\alpha }_{0,\text{ m}}+{\mathbb{I}}{\varepsilon }_{\text{w}\left(\text{t}\right),\text{m}}}={\text{R}}_{0,\text{m}}{\text{e}}^{{\mathbb{I}}{\varepsilon }_{\text{w}\left(\text{t}\right),\text{m}}}$ as described in the text. Once reached the specific date set by the researcher, the reproduction number of the recipient group will be replaced by the reproduction number of the donor group such that the contact pattern change on disease transmission can be transposed rendering the model counterfactual.

**Fig. 2:**
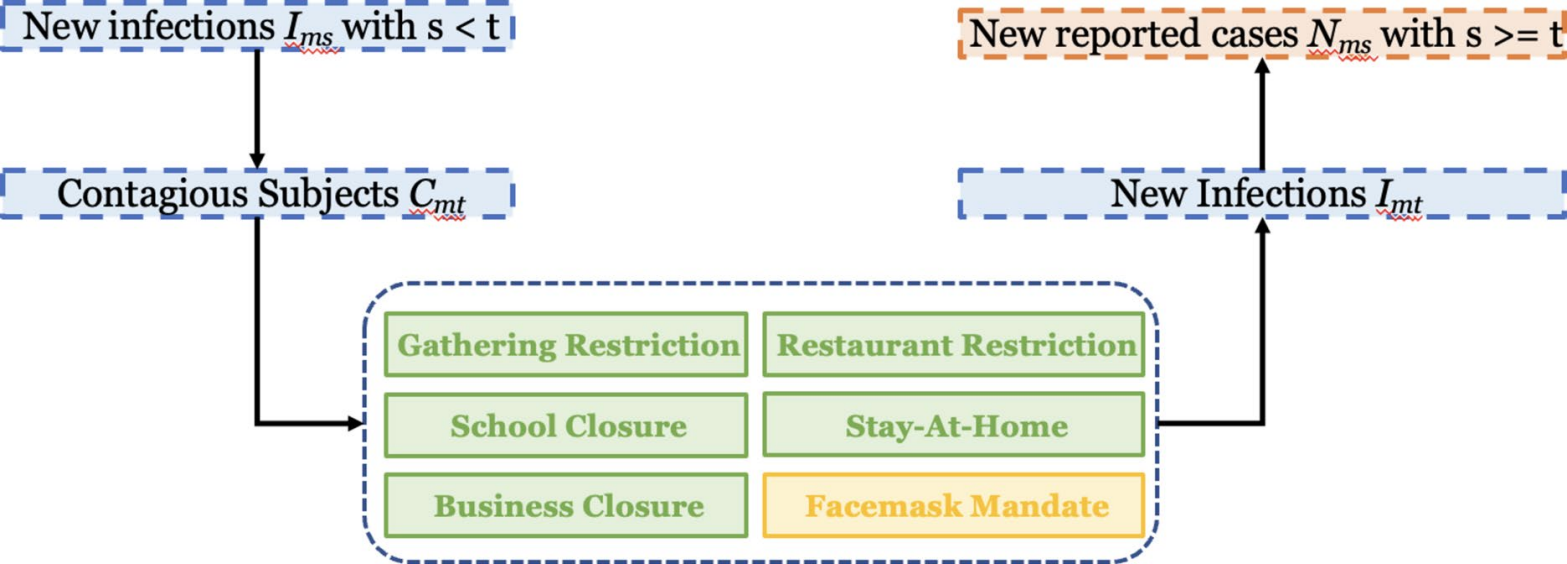
Analysis 3 uses the Advanced Bayesian Hierarchical Model Framework, where it is assumed that (i) the daily number of true new infections is related to the daily number of contagious subjects and the presence of non-pharmaceutical interventions, (2) the observed daily number of new infections is to the daily number of true new infections; and (3) the daily number of contagious subjects is related to the daily number of true new infections.

Figures 1 and 2 shows the flow diagram for Prototypical and Counterfactual Bayesian Hierarchical models and Advanced Bayesian Hierarchical model, respectively. Detailed explanation of the methodologies is available in the supplementary material.

## Results

**Fig. 3:**
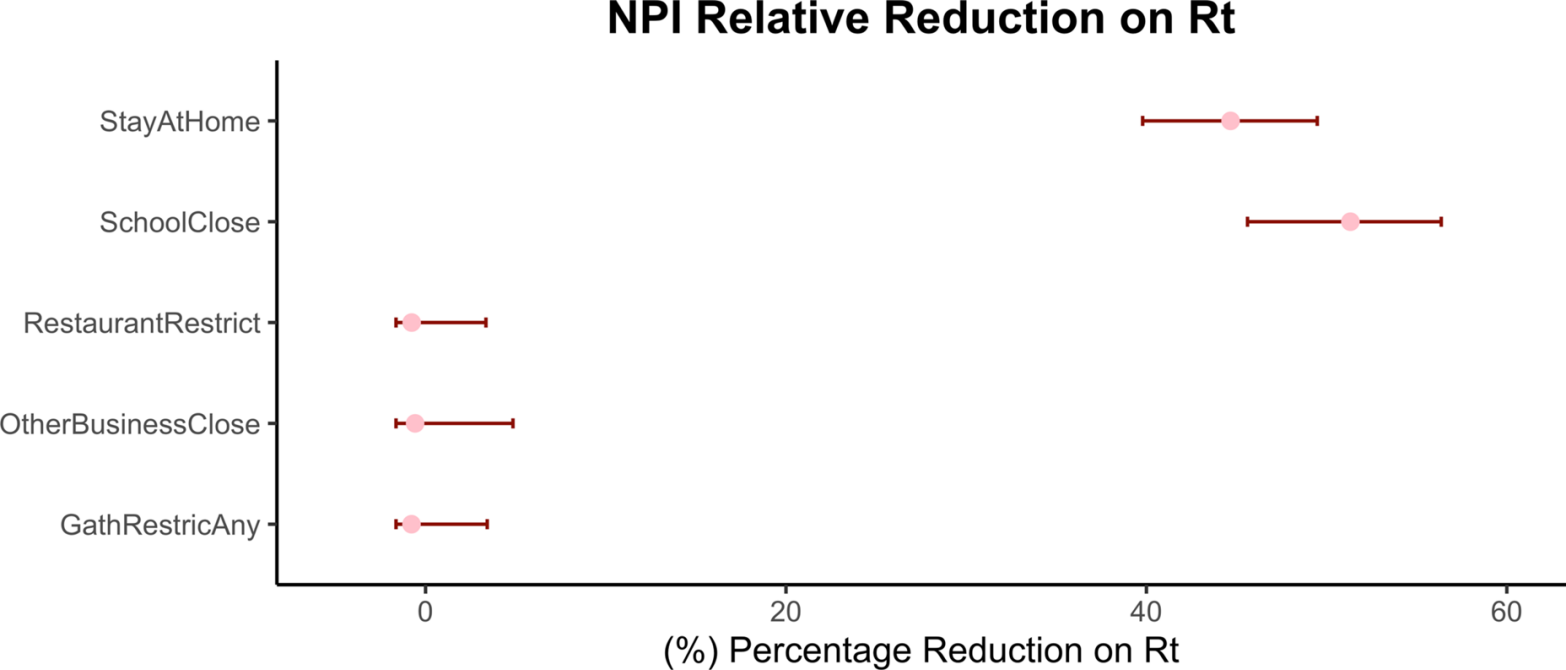
Mean relative percentage reduction in ${\text{R}}_{\text{t}}$ and its 95% credible interval for each of the NPIs from Analysis 1. Smaller relative percentage reduction in ${\text{R}}_{\text{t}}$ suggests less effectiveness of the NPI while larger relative percentage reduction indicates that the NPI contributes more to reducing the COVID-19 transmission. Figure shows School Closure is the most effective NPI while the Stay-at-Home order is the second and the other three NPIs are deemed to be ineffective all at the 95% confidence level.

Our first analysis using the prototypical Bayesian hierarchical model on 42 selected states shows that school closure is the most significant intervention in reducing the reproduction number, closely followed by the stay-at-home order. Restriction on restaurants, limitations on gathering and other business closure appears to be less effective according to our analysis. Figure 3 shows the percentage reduction on ${R}_{t}$, the reproduction number, for each NPI under the models that excluded the outlier states.

**Fig. 4:**
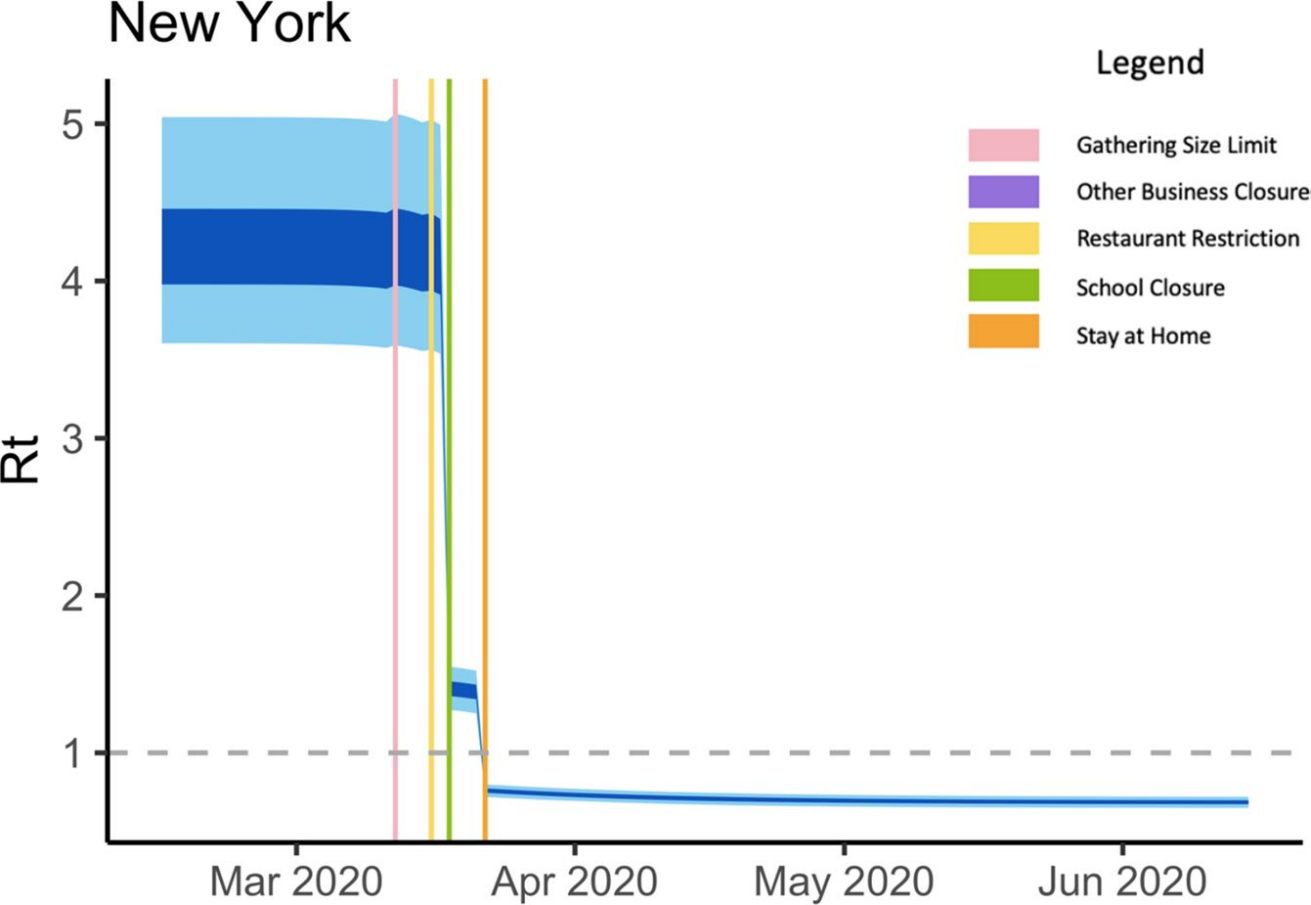
R_t_ estimates for New York State over the study period using analysis 1 with the 95% and 50% credible intervals in light blue and dark blue, respectively. The start date of gathering size limit, other business closure, restaurant restriction, school closure, and stay at home orders are marked in pink, purple, yellow, green, and orange, respectively.

The prototypical Bayesian hierarchical model also estimated the time-varying reproduction number over the study period. Here ${R}_{t}$ is a piecewise function and is assumed to change after an intervention took place. Each state has its own initial reproduction number, which is the basic reproduction number ${R}_{0}$ estimated before the state employed any NPIs. In our study, the initial reproduction number ${R}_{0}$ has been estimated to range from 2.24 (Washington) to 4.93 (New York) and Supplementary Table 6 shows the complete list of our estimates for the other states. According to a systematic review by Park et al.^24^, the basic reproduction number ${R}_{0}$ for COVID-19 has been estimated to range from 1.9 to 6.5 based on 16 related studies and our estimates are within the range for all states covered. The time varying basic reproduction number at time t, ${R}_{t}$, as estimated for the epicenter of the first wave, the New York State^25^, is shown in Fig. 4. The complete list of ${R}_{t}$ estimate plots for all 42 states is provided in the Supplementary Figure List 1.

**Fig. 5:**
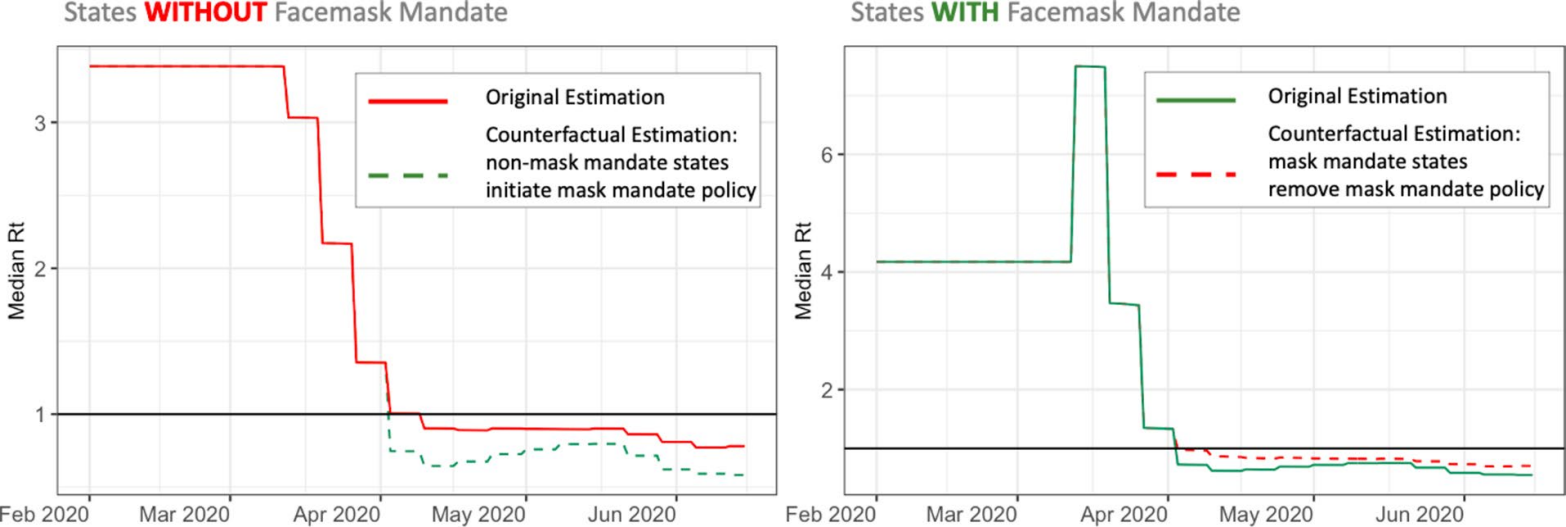
Estimated median time varying reproduction numbers from analysis 2 using the Counterfactual Modeling approach. The left figure shows the counterfactual ${\text{R}}_{\text{t}}$ profiles for non-mask mandate states adopting the mask mandate policy in green and the original fitted ${\text{R}}_{\text{t}}$ profiles in red. The right figure shows the counterfactual ${\text{R}}_{\text{t}}$ profiles for mask mandate states if they had removed the mask mandate policy in red and the original fitted ${\text{R}}_{\text{t}}$ profiles in green.

Secondly, we conducted the counterfactual modeling to examine the effectiveness of the mask-mandate. According to the counterfactual model, the median basic reproduction number ${R}_{0}$ is estimated to be 3.38 for states without face mask mandate and 4.17 for states with face mask mandate. The time-varying reproduction number ${R}_{t}$ at the end of the study period is estimated to be 0.78 for states without face mask mandate and 0.55 for states with face mask mandate. The median time-varying reproduction number ${R}_{t}$ in non-face mask mandate states at the end of the study period would have decreased by 25.34% had they imposed the mask-mandate. Conversely, the time-varying reproduction number ${R}_{t}$ in mask-mandate states at the end of the study period would have increased by 28.09% had they not implemented the mask-mandate. Figure 5 shows the original fitted and counterfactual estimated time-varying reproduction number profiles for the entire study period. The estimated cumulative counterfactual number of infections in non-mask-mandate states would have decreased by 45.87% had they imposed the mask-mandate. Similarly, the estimated cumulative counterfactual number of infections in states with mask-mandate would have increased by 65.95% had the mask-mandate been removed.

Thirdly, the advanced Bayesian hierarchical model was used to conduct analysis to estimate the effectiveness of individual non-pharmaceutical interventions for all 50 states in the United States and the District of Columbia. The study period remains the same as the previous two analyses, considering it was the time that states imposed non-pharmaceutical interventions most widely and most heavily in terms of the extent of strictness.

When implementing the prototypical Bayesian hierarchical model and counterfactual Bayesian hierarchical model, only 42 states were included. Nine states were not included because the death count in those states were too stratified and low, especially during the early stage of the pandemic; otherwise, the low death count would significantly bias the model and our results would be less accurate. However, nine states, out of the total 51 states (50 states in the United States and the District of Columbia), is about 18% and the analysis results obtained from 82% states would not be a complete representation for the entire United States. To have a completely full-scale estimation of the non-pharmaceutical intervention effectiveness for the entire US, we resort to use an advanced Bayesian hierarchical model that only uses the daily number of reported infections data and not the death data in the analysis. The number of infections is much higher than the number of deaths and so allows all 50 states in the United States and the District of Columbia to be included into the analysis. The daily number of reported cases were obtained from the Johns Hopkins University COVID-19 Dashboard^23^.

**Fig. 6:**
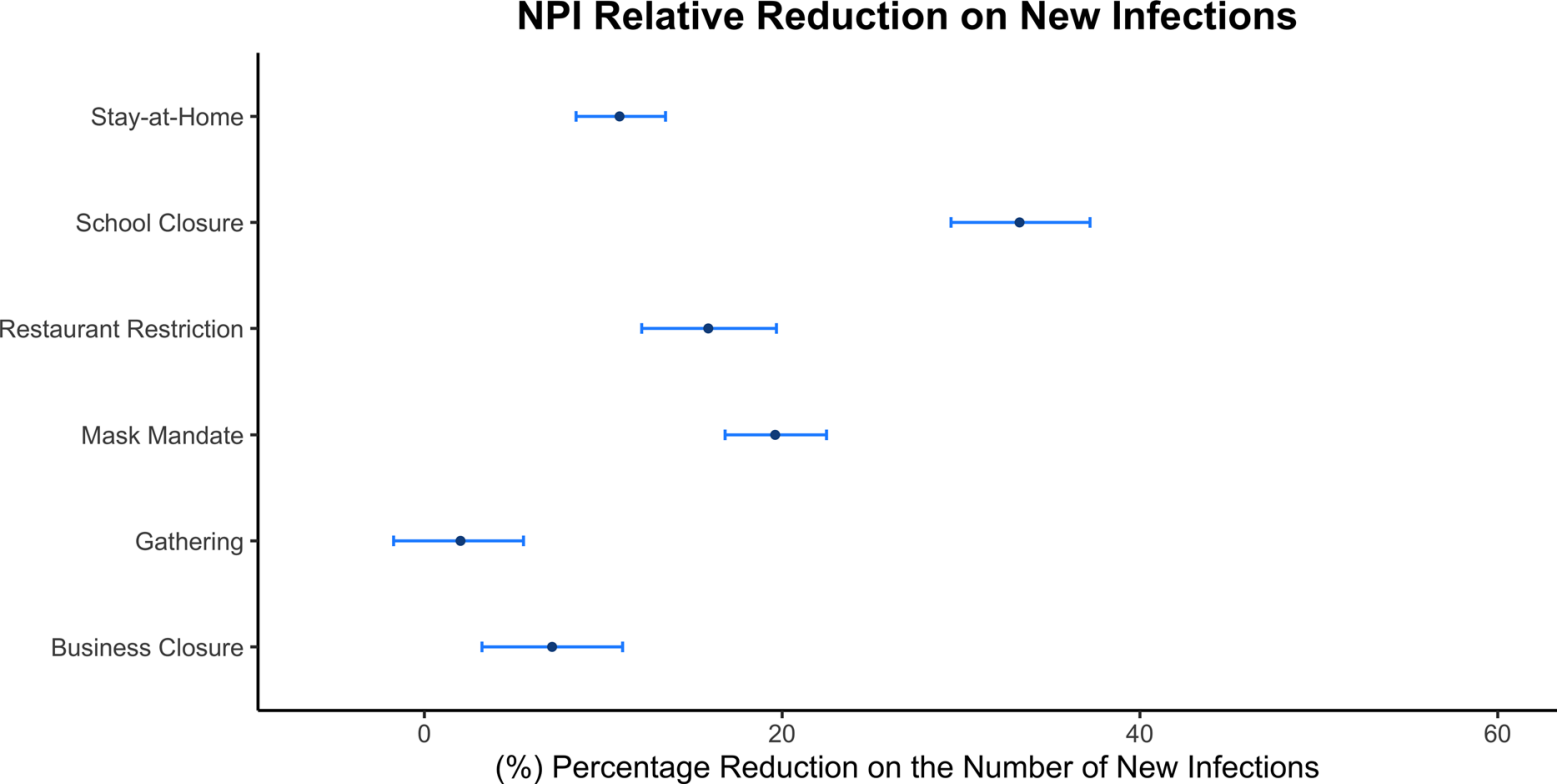
Relative Reductions in percentage of each NPI in new infections using Analysis 3. For each NPI, the posterior mean percentage reduction in the number of new infections is shown in solid dark dots and its 95% credible interval are shown in blue error bars. Smaller relative percentage reduction in the number of new infections suggests less effectiveness of the NPI and larger relative percentage reduction indicates the NPI contributes more to curbing the transmission of COVID-19.

The advanced Bayesian hierarchical model was implemented on the state-level data, and the effectiveness of each NPI was estimated by the relative reduction in the number of new infections. Figure 6 shows the results.

From our estimation, school closure was found to be the most significant intervention with 33.27 mean percentage of reduction in the number of new infections, and its 95% credible interval was from 29.44 to 37.21. This was followed by face mask mandate with 19.61 mean percentage of reduction in the number of new infections and 95% credible interval from 16.81 to 22.48. Restaurant restriction has a mean percentage reduction in the number of new infections at 15.87 with 95% credible interval from 12.15 to 19.68. The stay-at-home order has a mean percentage reduction in the number of new infections at 10.91 with 95% credible interval from 8.48 to 13.48. Other business closure has a mean percentage reduction in the number of new infections at 7.14 with 95% credible interval from 3.22 to 11.08. The restriction on gathering size was found to be the least effective NPI since its mean percentage reduction in the number of new infections is at 2.02 with 95% credible interval from − 1.72 to 5.54. Therefore, we concluded that school closure, face mask mandate, restaurant restriction, and stay-at-home are effective in mitigating the transmission of COVID-19 since the percentage reduction in the number of new infections were all above 10%; other business closure and restrictions on gathering size were found to be less effective since the percentage reduction on the number of new infections were less than 10%.

**Fig. 7:**
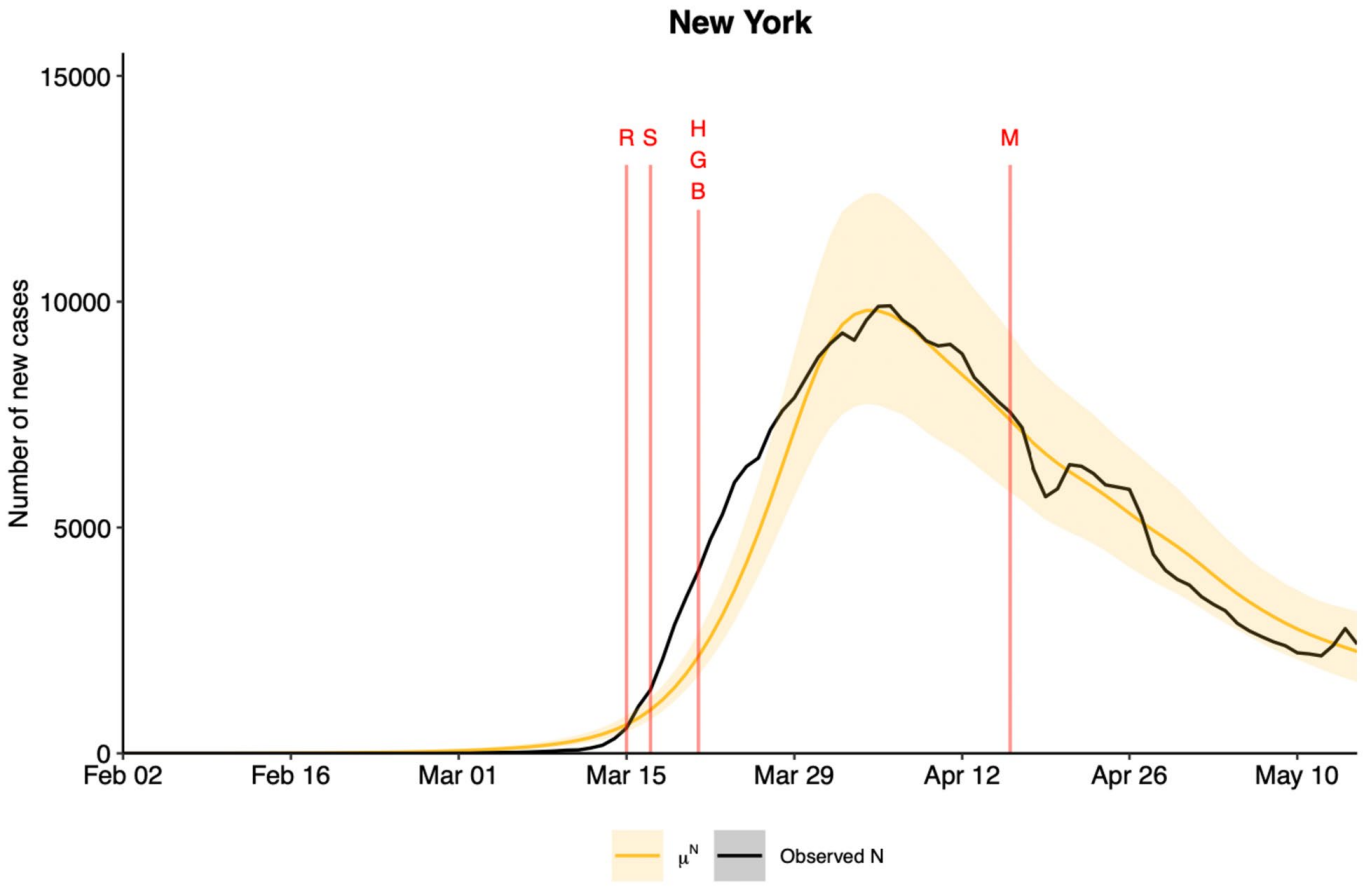
Expected reported daily number of new infections (the posterior mean) in yellow and 95% credible bands shaded in light yellow from Analysis 3 and the daily number of new infections reported by the Johns Hopkins University Dashboard in black for the State of New York over the study period. The vertical line in red indicates the first day that each NPI was implemented in New York (S: School closure; R: Restaurant Restriction; G: Restriction on Gathering Size; B: Other Business Closure; H: Stay-at-Home Order; M: Face mask Mandate).

We also compared the expected daily number of observed cases of new infections obtained from our analyses with the real daily number of observed cases to visually inspect the model fit. Figure 7 depicts two trajectories: one represents the actual daily count of observed cases, while the other, derived from the advanced Bayesian hierarchical model. The data for Fig. 7 comes from the State of New York and the corresponding prediction results for all other states in the United States and the District of Columbia are given in the Supplementary Figure List 3. Similar to the deductions we made from Fig. 7, the below figure also shows the observed cases nearly fall within the 95% credible intervals over the entire study period.

**Fig. 8:**
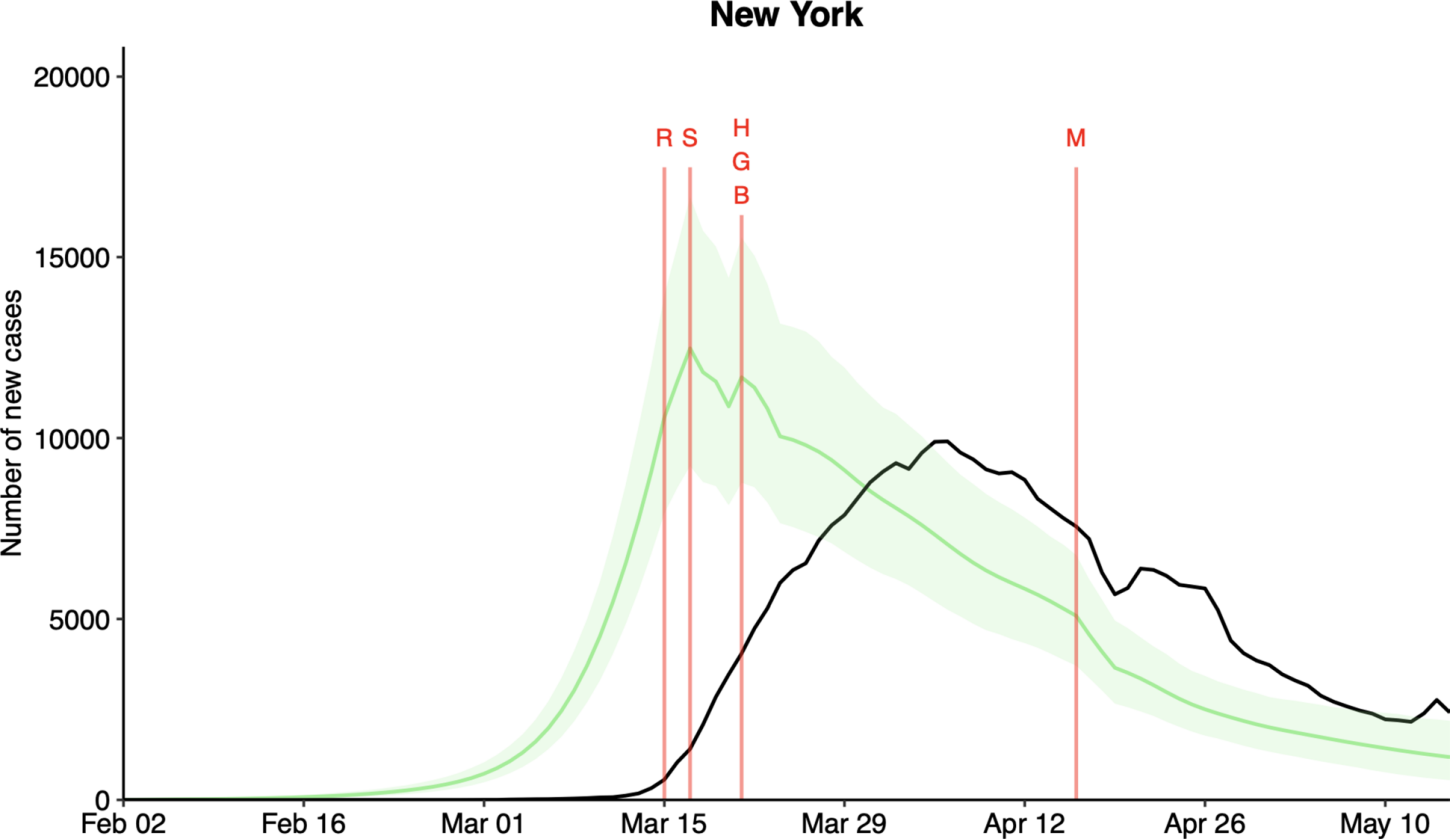
Expected Daily Number of True New Infections (the posterior mean) in green and 95% credible interval shaded in light green using Analysis 3 and the daily number of new infections reported by the Johns Hopkins University Dashboard in black for the State of New York over the study period. The vertical line in red indicates the first day that the NPI was implemented in New York (S: School closure; R: Restaurant Restriction; G: Restriction on Gathering Size; B: Other Business Closure; H: Stay-at-Home Order; M: Face mask Mandate).

Models and results in this work can help government authorities making timely decisions on the NPI implementation strategies against COVID-19 in all 50 states in the United States and the District of Columbia. More specifically, by restoring the expected daily number of true new infections using the advanced Bayesian hierarchical model, we can examine the disease transmission pattern in the state and monitor whether there is a sudden decrease in the expected daily number of true new infections after implementation of an NPI. Furthermore, a potential new exponential outbreak in the state would be indicated by an increment in the width of the credible interval. The distribution shift was due to the time delay between infection and reporting. Figure 8 shows the plots for the State of New York, and a complete list for all 50 states in the United States and the District of Columbia can be found in the Supplementary Figure List 4.

The State of New York was the epicenter of the COVID-19 outbreak in the United States during the first wave of the pandemic, and the state government imposed a variety of NPIs to mitigate the disease spreading. School closure, restrictions on gathering size, other business closure, and the stay-at-home order were found to be effective in reducing the daily number of new infections in the State of New York, whereas restaurant restriction was not. The shift is an intentional feature of the model, designed to account for the reporting delay between the actual occurrence of new infections (represented by the green curve) and the subsequent reporting of these cases in the JHU Dashboard (depicted by the black curve)^23^. This delay is a well-documented aspect of epidemiological reporting, primarily arising from the time taken to develop symptoms, seek testing, process tests, and finally report confirmed cases. Our model incorporates this lag to more accurately reflect the real-world process of infection reporting.

What is the optimal number of NPIs to be implemented? Supplementary Fig. 3 suggests an answer to this practical question, which was motivated by the need to ease the burden put on the population. We estimated that implementing five NPIs would have positive effects on reducing the number of new infections, whereas implementing four non-pharmaceutical interventions would result in greater than 10% in the percentage reduction in the number of new infections. Supplementary Fig. 2 shows the frequency of at least $m$ non-pharmaceutical interventions with positive effects and greater than 10%.

## Discussion

In this study, we have performed three analyses to study the effectiveness of six NPIs. The first analysis via the prototypical Bayesian hierarchical model investigated five NPIs: gathering size restrictions, business closures, restaurant capacity limits, school closures, and stay-at-home orders. This analysis identified school closure as the most significant preventive measure, with the stay-at-home order being the second most effective. Our second analysis using the counterfactual model showed that the face mask mandate was significant in reducing both new infections and death. The third analysis employed the advanced Bayesian hierarchical model and using the data from the entire United States verified our findings from the first two analyses. Supplementary Table 5 shows our results are consistent with seven previous studies^26–32^ based on the United States. The studies conducted by Olney et al.^26^, Dreher et al.^27^, Li et al.^28^ and Courtemanche et al.^29^ have provided valuable early insights into the effectiveness of NPIs. However, their analyses were limited to periods before the end of April 2020. By this time, several states had yet to implement a full range of NPIs. Our study extends the analysis period to June 15, 2020, encompassing additional measures that were implemented post-April. This extended timeframe allows us to capture a more comprehensive picture of the NPIs' impacts as the pandemic evolved. Furthermore, the studies by Ebrahim et al.^30^ and Jalali et al.^31^, while contributing to the discussion, did not employ statistical modeling techniques that would account for potential confounders. Instead, they relied on hypothesis testing without the benefit of predictive modeling. Our study, in contrast, has utilized robust statistical modeling to estimate the effects of NPIs yielding a more nuanced understanding of their effectiveness. Lastly, the research by Zhang et al.^32^ focused on only two NPIs. In contrast, our study examined six different NPIs and provide a more detailed analysis of the varied effects of multiple interventions. Additionally, we have also enhanced the granularity of the findings allowing a more in-depth assessment of how each different NPIs would contribute to the observed outcomes. In summary, our study builds upon and extends the findings of previous research by covering a longer study period, incorporating a wider array of NPIs, and utilizing advanced statistical modeling techniques that could account for confounding factors. The upshot is that we have a more complete understanding of the effectiveness of NPIs during the initial stage of the COVID-19 pandemic in the United States.

In closing, we note there are clear limitations of our work. Firstly, the epidemiological parameters used in our models, such as the infection fatality ratio (IFR)^24^ and time-to-event distributions^33–35^, were based on studies from Europe, which may exhibit different characteristics due to different geological, sociological, and cultural backgrounds. Secondly, we only modelled the NPIs that were mandated statewide and ignored those mandated regionally or adopted voluntarily. Thirdly, the reproduction number was modelled as a piecewise function that only changes when a new intervention was introduced. In other words, the effect of NPI was assumed to be constant once introduced whilst the population compliance towards the NPI could be changing over time. Furthermore, our counterfactual model's assumptions include the basic reproduction number estimates, which are inherently confounded with estimates of infection seeding in the two groups. This leads to a degree of uncertainty in our findings. Another key presumption is that the Rt values calculated for both groups inherently encapsulate a broad spectrum of geographic, social, cultural, and demographic disparities in disease transmission within each group. It's important to note that these heterogeneities could also influence the relative success of the overall COVID-19 response, beyond the differences in the face mask mandate policy. Lastly, our study only focused on the first wave of the COVID-19 pandemic in the United States ranging from February 1, 2020, to June 15, 2020, to reduce confounding effects such as different virus strains and their distributions among different states.

## Supplementary Information

Supplementary Information.

## Data Availability

The datasets analyzed during the current study does not require administrative permissions to access, and are available in the “Effectiveness_of_NPI” repository, https://github.com/cutsuper/Effectiveness_of_NPI/tree/main
