# Supplementary material for: Effectiveness of non-pharmaceutical interventions for COVID-19 in USA
Source: Sci Rep. 2024 Sep 13;14:21387. doi: 10.1038/s41598-024-71984-1 (PMC11399256; doi:10.1038/s41598-024-71984-1)
Supplement: Supplementary file 1 — Supplementary Information. [file 41598_2024_71984_MOESM1_ESM.docx]

### **Supplementary Material**

- **Methodology Details**

The effectiveness of each NPI is expressed as a relative percentage reduction in the basic reproduction number $R_{0}$, which is the expected number of cases directly generated by one case in a population where all individuals susceptible to infection. The interpretation of *R_0_* is that the disease is expected that to spread wider if *R_0_ > 1*, become stable if *R_0_ = 1*, or die down if *R_0_ < 1*. Each state will have its own basic reproduction number, denoted by R_0, m._

Let $D_{t,m}$be the number of deaths on day *t* in state *m* and assume it follows a negative binomial distribution with mean $d_{t,m}$and variance given by $d_{t,m}+\frac{{d_{t,m}}^{2}}{\psi}$ , where $\psi$ follows a half normal distribution.^1^ The expected number of deaths is obtained by a discrete sum:

$d_{t,m}={ifr}_{m}^{*}\sum_{\tau=0}^{t-1} c_{\tau,m}\pi_{\tau,m}$ (1)

where $\pi_{\tau,m}$ denotes the time between infection and death and is calculated as the sum of the incubation period and the time between onset of symptoms and death for state *m* at day $\tau$. Furthermore, each of the prior distributions $\pi$ is discretized by $\pi_{s,m}=\int_{s-0.5}^{s+0.5} \pi\left( \tau\right) d\tau$ ,  *s* = 2, 3, …, with $\pi_{1,m}=\int_{0}^{1.5} \pi\left( \tau\right) d\tau$. The incubation period is assumed to have a gamma prior $\pi_{1}$with mean 5.1 days and a coefficient of variation 0.86 and the time between onset of symptoms and death is assumed to have a gamma prior $\pi_{2}$ with mean 17.8 days and coefficient of variation 0.45^2,3^. The adjusted infection fatality ratio for each state, denoted as ${ifr}_{m}^{*}$, is assumed to follow a normal prior distribution with a mean of 1 and a variance of 0.1, reflecting the average infection fatality ratio of 1% for states, as detailed in Supplementary Table 3 of Flaxman's study^1^. To accommodate expected discrepancies among states, we incorporate random noise effects into each state, characterized by a variance of 0.1. The term $c_{\tau,m}$ is the number of new infections on day $\tau$ in state m and is given by a discrete convolution function:

$c_{t,m}=\left( 1-\frac{\sum_{\tau=1}^{t-1} c_{\tau,m}}{N_{m}} \right)R_{t,m}\sum_{\tau=0}^{t-1} c_{\tau,m} g_{t-\tau}$ (2)

Here *g* is the generation interval function which is the time lag between an infected case and his or her infector and is estimated to be gamma distributed with mean 6.5 days and coefficient of variation 0.62 days^4^. Further, g is discretized by $g_{s}=\int_{s-0.5}^{s+0.5} g\left( \tau\right) d\tau$*, s = 2, 3, …,* with $g_{1}=\int_{0}^{1.5} g\left( \tau\right) d\tau$. The term $N_{m}$ is the total population in state *m*. The framework also includes a population adjustment factor $1-\frac{\sum_{\tau=1}^{t-1} c_{\tau,m}}{N_{m}}$ to account for the population saturation of susceptible subjects. The time-varying reproduction number $R_{t,m}$ can be obtained through the prototypical Bayesian hierarchical model for estimating the effectiveness of the 5 commonly used NPIs and the counterfactual model for estimating the effectiveness of the face mask mandate.

a) The Prototypical Bayesian Hierarchical Model

The renewal equation is scaled by the state-specific time-varying reproduction number $R_{t,m}$, which models the average number of secondary infections at a given time. A piecewise constant function is used to scale $R_{t,m}$ from a baseline prior $R_{0,m}, which is chosen to be N+(3.28, ||) with \sim N(0, 0.5). This choice$is motivated by earlier results from major non-pharmaceutical interventions occurring in different states and times.^5^ The model installs an indicator variable $I_{k, t, m}$ for interventions, which equals to 1 if intervention $k$ is in place in state $m$ at time $t$ and 0 otherwise. The effect of each intervention is assumed to be multiplicative. Therefore $R_{t,m}$ is a function of intervention indicators $I_{k, t, m}$ in place at time *t* in

state *m*, and is given by:

$R_{t,m}=R_{0,m}*exp\left( -\sum_{k=1}^{n} \alpha_{k}I_{k,t,m} \right)$ (3)

The intervention-specific effects $\alpha_{k}$ are shared among all states. In short, the time-varying reproduction number in the prototypical Bayesian hierarchical model is dependent upon the basic reproduction number and the active NPIs.

b) The Counterfactual Bayesian Hierarchical Model

For the face mask mandate policy, we estimated its effectiveness through the counterfactual model because 28 states did not impose the face mask mandate during the study period. The primary quantity of interest to estimate is the time-varying reproduction number, $R_{t,m}$, using the daily death data with its initial values assumed to be equal to the basic reproduction number *R_0_* before March 13, 2020, which is the week when the states imposed most of these NPI measures. Unlike the prototypical Bayesian hierarchical model, we used a random walk to capture the trajectory changes in disease transmission triggered by the implementation of the face mask mandate. We modelled the time-varying reproduction number $R_{t,m}$ by a random process given by:

$R_{t,m}=R_{0,m}e^{\mathbb{I}\varepsilon_{w\left( t \right),m}}$ (4)

Here $\varepsilon_{w\left( t \right),m}$ is a stochastic process for week $w\left( t \right)$ and is defined to be a discrete random walk. To specify this process, the parameter $\gamma$ is introduced^6^ with $\gamma\sim N(0, \sigma_{0})$ and subsequently we model the stochastic process as $\varepsilon_{w\left( t \right),m}\sim N(\varepsilon_{w\left( t \right)-1,m}, \gamma)$.

The "counterfactual" refers to a hypothetical scenario used for comparison: what would have happened in the recipient group if they had adopted donor groups’ policy. The terms "donor group" and "recipient group" are being used to distinguish between two sets of states based on whether they have implemented face mask mandate policy. Considering the counterfactual part of the modeling, we let *y* be the donor group (e.g. non-facemask mandate states) and let *x* be the recipient group (e.g. facemask mandate states). We chose the start date to be March 13, 2020, and replaced the reproduction number of the recipient group with the reproduction number of the donor group. Specifically, we used the actual (i.e., the fitted) $R_{t}$ values for groups x and y given by $R_{t,x}=e^{\alpha_{0, x}\mathbb{+I}\varepsilon_{w\left( t \right),x}}$ and $R_{t,y}=e^{\alpha_{0, y}\mathbb{+I}\varepsilon_{w\left( t \right),y}}$, respectively, and we have the following counterfactual model:

$R_{t, y\to x}=\left\{ \begin{matrix} e^{\alpha_{0, x}}e^{\varepsilon_{w\left( t \right),x}} if t<start date \\ \\ e^{\alpha_{0, y}}e^{\varepsilon_{w\left( t \right),y}} if t\geq start date \end{matrix} \right.$ (5)

c) The Advanced Bayesian Hierarchical Model

Our general goal in this paper is to evaluate the effectiveness of each of the individual non-pharmaceutical interventions through the daily number of new infections. The motivation for the advanced Bayesian hierarchical model is that it connects two unobserved quantities to an observed quantity. The two unobserved quantities are: 1) the daily number of new infections in region m on day *t* (*I_mt_*), and 2) the daily number of contagious subjects in region m on day *t* (*C_mt_*)*.* The former refers to individuals who might have not developed any symptoms or have received a COVID-19 test but are already infected, and the latter refers to individuals, who are capable of infecting others. The observed quantity from this model refers to the observed daily number of new infections in region *m* on day *t* reported by Johns Hopkins University (JHU) CSSE COVID-19 Dashboard^7^. This number is denoted by *N_mt_* and refers to the daily number of new infections reported by local authorities.

The first sub-model is giving the expected daily number of true new infections and is a function of the daily number of contagious individuals, the region-specific transmission rate, and the presence of preventive interventions. This expected daily number is modelled by:

$\mu^{I_{mt}}=C_{mt}\cdot\delta_{m}\cdot\prod_{k=1}^{6} (1-\theta_{k}\sum_{r=1}^{R_{m}} p_{rm} f(T_{krmt}))$ (6)

Here *krmt* referring to the *r^th^* region in state *m* at day *t* for intervention *k*. In the absence of all preventive measures, the expected daily number of true new infections $\mu^{I_{mt}}$ are only determined by the daily number of contagious subjects $C_{mt}$ and the region-specific transmission rate $\delta_{m}$. The region-specific transmission rates $\delta_{m}$ are modelled by the following exponential function:

$\delta_{m}=\exp\left( \beta+\beta_{m} \right)$ (7)

The log global transmission rate when there is no intervention implemented is $\beta$ and its prior distribution follow a student-t distribution with mean 0, standard deviation 10, and has 7 degrees of freedom^8^. The term, $\beta_{m}$, is a regional variation term and its prior distribution is a normal distribution with mean 0 and standard deviation $\tau$ . The advanced Bayesian hierarchical model framework further assumes that the prior distribution of the standard deviation $\tau$ is modeled using a half Student-t distribution with mean 0, standard deviation 1, and degrees of freedom 4.

When non-pharmaceutical interventions are introduced, a scaling term should be used to reflect the implementation of the preventive measures. In Equation 6, the product term$\prod_{k=1}^{6} (1-\theta_{k}\sum_{r=1}^{R_{m}} p_{rm} f(T_{krmt}))$ accounts for the fraction of unavoidable infections, *f* is a function to be explained later and $\theta_{k}$ is the relative reduction in new infections when the k^th^ NPI is fully implemented, i.e. $\theta_{k}$ is the fraction of avoided new infections compared to the situation without the intervention. Thus the prior distribution for $\theta_{k}$ should have the following properties: (a) it takes values between negative infinity and 1 to model negative effects from an increase in the number of new infections and complete elimination of disease spreading, and (b) the prior distribution should give higher probability to positive effects than to negative effects, as the primary purpose of implementing preventive measures is expected to change contact patterns within the population and curb the disease spreading. Therefore, half normal distribution for the negative effects with probability $\omega$ and uniform distribution for the positive effects with probability (1- $\omega$) was selected as the prior distribution of $\theta_{k}$:

$$\theta_{k}\sim Mixture\left( \omega\right)=\left\{ \begin{matrix} Half Normal \left( 0, \sigma\right) with probability \omega\\ Uniform \left( 0, 1 \right) with probability (1-\omega) \end{matrix} \right.$$

where $\omega\in[0,1]$ and $\sigma=\frac{\omega}{(1-\omega)\sqrt{2\pi}}$ . The half-normal distribution accounted for the negative effects and the standard uniform distribution with probability $1-\omega$ accounted for the positive effects. The mixing ratio, $\omega$, is chosen to be 0.1 by default, which implies that the NPI can cause an increment in the number of new infections with probability 0.1.

The advanced Bayesian hierarchical model also takes the sub-regional effects into account. Specifically, the summation term sums up the effects in $r$ sub-regions. The share of sub-region $r$’s population out of the total population in the region, is denoted by $p_{rm}$. When sub-regional effects won’t affect the analysis, all sub-regions are integrated together and $r$ is set to be 1.

The number of days since NPI $k$ took effect in sub-region $r$ of region $m$on day $t$ is denoted by $T_{krmt}$. The function $f(t)$ in (6) takes values between 0 and 1 and describes the degree and extent of the non-pharmaceutical intervention, and it can be interpreted as a time-delayed response function. Larger values of $f(t)$ suggest greater intervention effect of the NPI. Ideally, the non-pharmaceutical intervention is fully effective once it is imposed, but it usually requires a few days to let the intervention become fully implemented. The time-delayed response function accounted for the period needed for the public to fully respond to the imposed non-pharmaceutical intervention. We expect that the effect of a preventive measure increases linearly since its implementation on day $t_{0}$until it achieves full efficacy on day $t_{1}$. Consequently, the time-delayed response function f(t) is modelled simply as follows:

$f\left( t \right)=\left\{ \begin{matrix} 0, if t\leq t_{0} \\ \frac{t-t_{0}}{t_{1}-t_{0}}, if t_{0}<t<t_{1} \\ 1, if t\geq t_{1} \end{matrix} \right.$ (8)

The daily number of true new infections was modelled by a lognormal distribution, with mean $\mu^{I_{mt}}$and standard deviation $\sigma^{I_{mt}}=\sqrt{\mu^{I_{mt}}(1+\frac{\mu^{I_{mt}}}{\phi^{I}})}$ where $\phi^{I}={(\frac{1}{\xi^{I}})}^{2}$ is the overdispersion parameter. The prior distribution of $\xi^{I}$ follows normal distribution with mean 0 and standard deviation 1^8^.

The second sub-model uses the weighted sum of the number of true new infections in the previous day to calculate the expected daily number of observed new infections. This implies that the expected number of new observed cases $\mu^{N_{mt}}$ in state *m* at day *t* is determined by the number of new infections in the previous days, as shown in Equation 9:

$\mu^{N_{mt}}=\sum_{s<t} I_{ms}\cdot p_{IN}(t-s)$ (9)

The term $p_{IN}(t)$ is the probability that a newly infected individual being reported $t$ days after infection and it has two parts. It can be written as the sum of the time from infection to symptomatic, which is usually referred to as the incubation period, and the time from symptomatic to being reported, which is the reporting delay. A systematic review concluded that the incubation period follows a lognormal distribution with mean 1.63 and standard deviation 0.5, which translated into an average incubation period of about 6 days^9^. A study on the COVID-19 outbreak in Italy concluded that the time delay in reporting follows a gamma distribution with shape parameter 1.88 and scale parameter 0.26, which translates into an average reporting delay of about 7 days^10^.

It is reasonable to assume the time from infection to symptomatic and the time from symptomatic to being reported are independent. Therefore, the distribution of the time from infection to being reported of a new case is the sum of independent and identically distributed variables. The prior distribution of the time from infection to reporting distribution of a new case is log-normally distributed with mean 2.47 and standard deviation 0.45, which translates into an average delay of about 13 days and standard deviation of about 6 days^1^.

A fixed choice for the parameters in the time from infection to reporting distribution may ignore the uncertainty about the shape of the distribution, therefore prior distributions were placed over the mean and the standard deviation and estimated as part of the overall model fitting procedure. In line with a hierarchical Bayesian model, a normal prior with mean 2.47 and standard deviation 0.5 was assumed for the mean of the time from infection to reporting distribution, and a gamma distribution with shape parameter 2 and scale parameter 4.48 was selected for the standard deviation of the time from infection to reporting distribution. The choice of the prior distributions reflects the prior knowledge about the means and variance, while also factors in the uncertainties.

It is helpful to discretize the time from infection to reporting time using the distribution of $p_{IN}(t)$ to simply the calculation. This is done through Equation 10 below:

$p_{IN}\left( t \right)=\left\{ \begin{matrix} \int_{0}^{0.5} p_{IN}\left( \tau\right)d\tau for t=0 \\ \int_{t-0.5}^{t+0.5} p_{IN}\left( \tau\right)d\tau for t=1, 2,\ldots\end{matrix} \right.$ (10)

The daily number of observed new infections was modelled by a negative binomial distribution, with mean $\mu^{N_{mt}}$and standard deviation $\sigma^{I_{mt}}=\sqrt{\mu^{n_{mt}}(1+\frac{\mu^{N_{mt}}}{\phi^{N}})}$ where $\phi^{N}={(\frac{1}{\xi^{N}})}^{2}$ is the overdispersion parameter. For simplicity, the prior distribution of $\xi^{N}$ follows a standard normal distribution.

The third sub-model uses the weighted sum of the number of true new infections in the previous day to calculate the expected daily number of contagious individuals. Accordingly, the expected number of contagious individuals $\mu^{c_{mt}}$ in state *m* at day *t* is determined by the number of new infections in the previous days, as shown in Equation 11.

$\mu^{C_{mt}}=\sum_{s<t} I_{ms}\cdot p_{IC}(t-s)$ (11)

The probability that a newly infected individual becomes contagious at day $t$ after infection is denoted by $p_{IC}(t)$. The data does not directly provide information on q(t), the probability that an infected individual infects another random individual in the population at day $t$ after his/her own infection, so we model it simply in terms of the generation interval distribution as follows:

$q\left( t \right)=p_{IC}(t)\cdot\eta$ (12)

where $\eta$ is the probability that a contagious individual infects another random individual in the population within one day.

Let $p_{G}\left( t \right)$ denote the density of the generation interval distribution, where the generation interval refers to the time between the infection time of an infected person and the infection time of his or her infector. In other words, given a person had infected another person, $p_{G}\left( t \right)$ is the probability that this happens $t$ days since his or her infection. Equation 13 shows the relationship between the generation interval distribution $p_{G}\left( t \right)$ and $p_{IC}(t)$, after noting that the proportionality factor could be discarded since $q(t)$ is a probability, and the probabilities on the entire domain always sum to 1.

$p_{G}\left( t \right)=\frac{q(t)}{\sum_{all t} q(t)}=\frac{p_{IC}(t)\cdot\gamma}{\sum_{all t} p_{IC}(t)\cdot\gamma}=\frac{p_{IC}(t)}{\sum_{all t} p_{IC}(t)}$ (13)

The prototypical Bayesian hierarchical model and the counterfactual Bayesian hierarchical model use serial interval distribution to approximate the generation interval distribution. The serial interval distribution refers to the duration between symptom onset of a primary case and symptom onset of its secondary cases. However, this approximation is controversial because it assumes that the incubation period is independent from the infectiousness profile^11^. If this strong assumption is not met, the difference between the serial interval distribution and the generation interval distribution could be substantial. By utilizing the data on the exposure for both index and secondary cases, a study estimated the generation interval distribution to be that of a Weibull distribution with shape parameter 3.28 and scale parameter 6.12. This translates, in practical term, into an average of about 5.49 days and standard deviation of 1.84 days^12^.

The generation interval distribution $p_{G}(t)$ is then discretized via Equation 14 for easier computation. On day 0, $p_{G}(t)$ has been explicitly set to 0 since we do not want to include the new infections on the same day that the number of contagious individuals is counted.

$p_{G}\left( t \right)=\left\{ \begin{matrix} 0 for t=0 \\ \int_{0}^{1.5} p_{G}\left( \tau\right)d\tau for t=1 \\ \int_{t-0.5}^{t+0.5} p_{G}\left( \tau\right)d\tau for t=2, 3,\ldots\end{matrix} \right.$ (14)

The advanced model treats the relationship between the expected number of contagious individuals $\mu^{C_{mt}}$ and the number of contagious individuals $C_{mt}$ as deterministic, which means that they are assumed to be equal.

- **Supplementary Tables**
- **Table 1:** Methods for Identifying Significant NPIs

| **Model/Methods** | **Goals** | **Data Needed** | **End Product** |
| --- | --- | --- | --- |
| Linear Regression  Leffler et al., 2020 (11) | Identifying significant predictors of COVID-19 related deaths (log-scale) | Daily number of deaths (observed); Disease duration; Duration of each NPI’s implementation in each region; Region-specific demographics and geographic info | P-values reported by the multiple linear regression indicating the significance |
| Logistic Regression  Dreher et al., 2021 (5) | Identifying significant predictors of dichotomized Rt (above 1 coded as 1, less than 1 coded as 0) | Each region’s Rt estimate on the day of interest; Duration of each NPI’s implementation in each region; | P-values reported by the logistic linear regression indicating the significance |
| Cox PH Model  Dreher et al., 2021 (5) | Identifying significant NPIs affecting time to 1000^th^ case | Each regions’ time to 1000^th^ case; Duration of each NPI’s implementation in each region; | P-values reported by the Cox PH regression indicating the significance |
| Linear Mixed Model  Pozo-Martin et al., 2021 (15) | Identifying significant NPIs affecting the weekly average growth rate | Each region’s cumulative number of confirmed cases (observed); Each NPI’s stringency index in each region; Total number of tests performed per thousand population in each region during the study period; region-specific geographical, social, and economical features | P-values reported by the linear mixed model indicating the significance |
| Panel Regression  Li et al., 2021 (12) | Identifying significant NPIs affecting mobility and COVID-19 growth rates | Each region’s daily number of confirmed cases and deaths (observed); Google mobility data for each region; Daily counts of positive and total tests in each region; Total population in each region; Each NPI’s start date/duration in each region | P-values reported by the panel regression model indicating the significance |

- **Table 2:** Methods on Estimating Joint Effectiveness of NPI Portfolios

| **Model/Methods** | **Goals** | **Data Needed** | **End Product** |
| --- | --- | --- | --- |
| SEIR-type Model  Ivorra et al., 2020 (9) | Estimating the joint effectiveness of NPI portfolios through reproduction number | Epidemiological parameters (disease contact rates, transition rates, infection reported rate, etc.); Each region’s daily number of infections and recoveries (observed); | Time-varying daily effective reproduction number Rt |
| Maximum Likelihood Approach  Azimi et al., 2020 (1) | Estimating the joint effectiveness of NPI portfolios through reproduction number | Each region’s daily number of infections (observed) | Time-varying daily effective reproduction number Rt |

- **Table 3:** Methods on Estimating the Effectiveness of Individual NPIs

| **Model/Methods** | **Goals** | **Data Needed** | **End Product** |
| --- | --- | --- | --- |
| Prototypical Model  Flaxman et al., 2020 (8) | Estimating the effectiveness of individual NPI | Daily number of deaths (observed); Start date of each NPI in each state | Each NPI’s relative percentage reduction on the reproduction number Rt |
| Counterfactual Model  Mishra et al., 2021 (13) | Estimating the pandemic situation having a region/group adopting other region/group’s NPI policy portfolios | Daily number of deaths (observed) | Percentage change on the reproduction number Rt, number of infections, and number of deaths |
| Advanced Model  Banholzer et al., 2021 (2) | Estimating the effectiveness of individual NPI | Daily number of infections (observed); Start date of each NPI in each state | Each NPI’s relative percentage reduction on the number of infections |

- **Table 4:** Summary on Previous Studies

| **Author** | **Model Used & Data Source** | **Setting & Study Period** | **NPIs Studied** |
| --- | --- | --- | --- |
| Chernozhukov et al. (4) | SEM & SEIR,  New York Times | USA, all states,  Mar 7 – Jun 3, 2020 | (1) Stay at-home / (2) Closed nonessential businesses / (3) Closed K-12 schools / (4) Face mask mandates for employees in public facing businesses |
| Courtemanche et al. (19) | Event Study Model, John Hopkins University | USA, 3138 counties,  Mar 1 – Apr 27, 2020 | (1) Shelter-in-place orders / (2) Public school closing / (3) Bans on large social gatherings / (4) Closures of entertainment-related businesses |
| Dreher et al. (5) | Linear & Logistic Regression,  John Hopkins University for cases and deaths, effective reproduction number were collected from Rt.live | USA, all states,  Up to Apr 30, 2020 | (1) Stay-at-home orders / (2) Educational facilities closure / (3) Non-essential business closure / (4) Limitations on mass gatherings |
| Ebrahim et al. (6) | T-test, Chi-square test,  Data collected by 104 trained volunteers | USA, 1320 counties, March to July 2020 | (1) Closure of nonessential workplaces / (2) Shelter-in-place/stay-at-home orders / (3) Enforcement of shelter-in-place or stay-at-home / (4) Size restrictions on public gatherings / (5) School closing / (6) Public transport closures / (7) Publicly available testing |
| Jalali et al. (10) | Kruskal-Wallis tests and Correlation Tests,  John Hopkins University | USA, 30 most populous counties and 10 most populous counties of CA, FL, NY and TX,  Mar 1 – May 31, 2020 | 3 broad categories: (1) Restrictions on mass gatherings / (2) Stay-at-home orders / (3) Face mask requirements |
| Li et al. (12) | Regression,  Google community mobility data | USA, all states,  Mar 1 – Jul 13, 2020 | (1) School closing / (2) Workplace closures / (3) Public event cancellations / (4) Public information campaigns / (5) Public transport closures / (6) Stay-at-home orders / (7) International/national travel controls |
| Olney et al. (14) | Bayesian Hierarchical Model, New York Times or cases and deaths, manually collected NPI start dates | USA, all states,  Feb 29 - Apr 25, 2020 | (1) Social distancing encouraged / (2) Schools or universities closing / (3) Public events (ban for more than 100 people)/ (4) Lockdown / (5) Self-isolating ill / (6) Sports (public event ban of more than 1000 people) |
| Banholzer et al. (2) | Bayesian Framework | 20 countries: 15 EU countries, USA, Canada, Australia, Norway and Switzerland | (1) School closing / (2) Border closures / (3) Public event bans / (4) Gathering bans / (5) Venue closing / (6) Lockdowns prohibiting public movements without valid reason / (7) Work bans on non-essential business activities |
| Bo et al. (3) | Generalized Linear Mixed Model | Worldwide, 190 countries | (1) Mandatory face mask in public / (2) Isolation or quarantine (3) Social distancing / (4) Traffic restrictions |
| Brauner et al. (20) | Bayesian Hierarchical Model | Worldwide, 41 countries: 34 European and 7 non-European | (1–3) Gatherings limited to 1000/100/10 people or less / (4–5) Some/ All but essential shops closed / (6–7) Schools or universities closed / (8) Stay-at-home orders with exemptions |
| Esra et al. (7) | Bayesian Framework | Worldwide, 26 countries and 34 US states | (1) Quarantine and isolation policies / (2) Limits on gatherings / (3) School closing (primary, secondary and tertiary educational institutions) / (4) Mask policies / (5) Household confinements (stay-at-home-orders, shelter-in-place orders and lockdowns) |
| Flaxman et al. (21) | Bayesian Hierarchical Model | Europe, 11 countries | (1) Lockdown / (2) Cancel public events / (3) School closing / (4) Self-isolation / (5) Social distancing encouraged |
| Fountoulakis et al. (22) | Correlation Test & Linear Regression | Europe, 40 countries | (1) School closing / (2) Workplace closing / (3) Public events ban / (4) Gathering ban / (5) Public transport closure / (6) Lockdown implementation / (7) Domestic travel ban / (8) International travel ban |
| Haug et al. (23) | LASSO, RF, TF | Worldwide, 79 territories, 56 countries | Different categories of NPIs in their hierarchical levels (42,151 measures) : School closing and gathering ban |
| Hunter et al. (24) | Bayesian Model | Europe, 30 countries | (1) Mass gathering restrictions / (2) Initial business closure / (3) Educational facilities closed / (4) Non-essential services closed / (5) Stay-at-home order / (6) Travel severely limited - none European country |
| Islam et al. (25) | Regression Model | Worldwide, 149 countries | (1) Closures of schools / (2) Workplace / (3) Public transport / (4) Restrictions on mass gatherings and public events / (5) Restrictions on movement (stay-at-home regulations and restrictions on movements within a country) |
| Koh et al. (26) | Regression | Worldwide, 142 countries | (1) International travel controls (including screening, quarantine and bans on international movement) / (2) Restrictions on mass gatherings (including public event bans and size restrictions on gatherings) / (3) Lockdown-type measures (including workplace closure, internal movement restrictions) |
| Li et al. (12) | Regression | Worldwide, 131 countries | (1) Closure of schools / (2) Closure of workplaces / (3) Public events bans / (4) Restrictions on the size of gatherings / (5) Closure of public transport / (6) Stay-at-home orders / (7) Restrictions on internal movement / (8) Restrictions on international travel |
| Liu et al. (27) | Regression | Worldwide, 130 countries | (1) Internal containment and closure (School and workplace closure, public event cancelation, limits on gathering sizes, public transport closure, stay-at-home requirement, internal movement restriction) / (2) International travel restrictions / (3) Economic policies / (4) Health systems policies (Public information campaign, testing policy, contact tracing) |
| Papadopoulos et al. (28) | Linear Regression | Worldwide, 137 countries | (1) School closing / (2) Workplace closing / (3) Cancelling of public events / (4) Restriction on gatherings / (5) Closure of public transport / (6) Stay-at-home restrictions / (7) Domestic travel restrictions / (8) International travel restrictions / (9) Public information / (10) Testing framework / (11) Contact tracing |
| Piovani et al. (29) | Multivariable Negative Binomial Regression for Panel Data | OECD countries | (1) Mass gathering ban (more than 1000 people) / (2) School closures |
| Pozo-Martin et al. (15) | Generalized Linear Mixed Model | OECD countries | (1) School closing requirements / (2) Workplace closing requirements / (3) Public events cancelling requirements / (4) Restrictions on gatherings / (5) Public transport restrictions / (6) Stay-at-home requirements / (7) Restrictions on internal travel / (8) International travel controls / (9) Public health information campaigns / (10) Mask wearing requirements / (11) Testing policy / (12) Contact tracing policy |
| Stokes et al. (30) | Linear Regression | Worldwide, 130 countries | (1) School closing / (2) Workplace closing / (3) Public event cancelling / (4) Gathering restrictions / (5) Public transport closure / (6) Stay-at-home requirements / (7) Restrictions on internal movement / (8) International travel controls / (9) Public information campaigns |
| Wibbens et al. (31) | Bayesian Framework | 40 territories: 17 countries and 23 US states | (1) Closing of schools / (2) Closing of workplaces / (3) Public event cancelling / (4) Gathering bans / (5) Public transport closure / (6) Shelter-in-place orders and home confinement / (7) Restrictions on internal movement / (8) Restrictions on international travel / (9) Public information campaigns / (10) Testing access / (11) Contact tracing |

- **Table 5:** Summary of Previous Studies on Estimating NPI Effectiveness in the United States

| **Author** | **Study Period** | **Model Used** | **Setting** | **NPIs Studied** |
| --- | --- | --- | --- | --- |
| **Olney et al., 2021 (14)** | 2/29/20 – 4/25/20 | Bayesian Hierarchical Model | USA, all states | (1) Social distancing encouraged / (2) Schools or universities closing / (3) Public events (ban for more than 100 people)/ (4) Lockdown / (5) Self-isolating ill / (6) Sports (public event ban of more than 1000 people) |
| **Dreher et al., 2021 (5)** | Early Outbreak in 2020 | Regression | USA, all states | (1) Stay-at-home orders / (2) Educational facilities closure / (3) Non-essential business closure / (4) Limitations on mass gatherings |
| **Li et al., 2021 (12)** | March – April 2020 | Regression | USA, all states | (1) School closing / (2) Workplace closures / (3) Public event cancellations / (4) Public information campaigns / (5) Public transport closures / (6) Stay-at-home orders / (7) International/national travel controls |
| **Courtemanche et al., 2021 (19)** | 3/1/20 - 4/27/20 | Regression | USA, 3138 counties | (1) Shelter-in-place orders / (2) Public school closing / (3) Bans on large social gatherings / (4) Closures of entertainment-related businesses |
| **Ebrahim et al., 2020 (6)** | March – July 2020 | T-test, Chi-square test | USA, 1320 counties | (1) Closure of nonessential workplaces / (2) Shelter-in-place/stay-at-home orders / (3) Enforcement of shelter-in-place or stay-at-home / (4) Size restrictions on public gatherings / (5) School closing / (6) Public transport closures / (7) Publicly available testing |
| **Jalali et al., 2020 (10)** | 3/1/20 - 5/31/20 | Kruskal-Wallis tests and Correlation Tests | USA, 30 most populous counties and 10 most populous counties of CA, FL, NY and TX | 3 broad categories: (1) Restrictions on mass gatherings / (2) Stay-at-home orders / (3) Face mask requirements |
| **Zhang et al., 2021 (18)** | February - August 2020 | Regression | US, all states | (1) Implementation of shutdowns / (2) Mask mandates |

- **Table 6:** State demographics and the basic reproduction number R_0_ before implementation of any NPIs.

| **State**^a^ | **Cumulative**  **cases** | **Cumulative**  **deaths** | **Population** | **Case perc (%)** | **Death perc (%)** | **R_0_** |
| --- | --- | --- | --- | --- | --- | --- |
| Alaska | 676 | 12 | 738432 | 0.0915 | 0.0016 |  |
| Alabama | 26485 | 774 | 4858979 | 0.5451 | 0.0159 | 3.22 |
| Arkansas | 12917 | 182 | 2978204 | 0.4337 | 0.0061 | 2.54 |
| Arizona | 36844 | 1203 | 6828065 | 0.5396 | 0.0176 | 3.46 |
| California | 157620 | 5114 | 39144818 | 0.4027 | 0.0131 | 3.52 |
| Colorado | 29299 | 1605 | 5456574 | 0.5369 | 0.0294 | 3.14 |
| Connecticut | 45235 | 4204 | 3590886 | 1.2597 | 0.1171 | 3.78 |
| District of Columbia | 9799 | 515 | 672228 | 1.4577 | 0.0766 | 3.47 |
| Delaware | 10340 | 543 | 945934 | 1.0931 | 0.0574 | 3.53 |
| Florida | 77326 | 2938 | 20271272 | 0.3815 | 0.0145 | 3.30 |
| Georgia | 58414 | 2494 | 10214860 | 0.5719 | 0.0244 | 2.83 |
| Hawaii | 736 | 17 | 1431603 | 0.0514 | 0.0012 |  |
| Iowa | 24082 | 658 | 3123899 | 0.7709 | 0.0211 | 2.76 |
| Idaho | 3399 | 87 | 1654930 | 0.2054 | 0.0053 |  |
| Illinois | 133016 | 6326 | 12859995 | 1.0343 | 0.0492 | 3.85 |
| Indiana | 40430 | 2386 | 6619680 | 0.6108 | 0.0360 | 3.53 |
| Kansas | 11319 | 246 | 2911641 | 0.3887 | 0.0084 | 3.15 |
| Kentucky | 12647 | 505 | 4425092 | 0.2858 | 0.0114 | 3.35 |
| Louisiana | 47172 | 3018 | 4670724 | 1.0100 | 0.0646 | 3.50 |
| Massachusetts | 105690 | 7647 | 6794422 | 1.5555 | 0.1125 | 3.91 |
| Maryland | 62032 | 2947 | 6006401 | 1.0328 | 0.0491 | 3.66 |
| Maine | 2810 | 101 | 1329328 | 0.2114 | 0.0076 | 2.87 |
| Michigan | 66085 | 6018 | 9922576 | 0.6660 | 0.0606 | 3.71 |
| Minnesota | 30693 | 1335 | 5489594 | 0.5591 | 0.0243 | 3.62 |
| Missouri | 16607 | 896 | 6083672 | 0.2730 | 0.0147 | 3.11 |
| Mississippi | 19799 | 895 | 2992333 | 0.6617 | 0.0299 | 3.31 |
| Montana | 609 | 19 | 1032949 | 0.0590 | 0.0018 |  |
| North Carolina | 45114 | 1140 | 10042802 | 0.4492 | 0.0114 | 3.52 |
| North Dakota | 3101 | 74 | 756927 | 0.4097 | 0.0098 |  |
| Nebraska | 16851 | 220 | 1896190 | 0.8887 | 0.0116 | 2.40 |
| New Hampshire | 5345 | 320 | 1330608 | 0.4017 | 0.0240 | 3.61 |
| New Jersey | 167669 | 12708 | 8958013 | 1.8717 | 0.1419 | 4.12 |
| New Mexico | 9845 | 440 | 2085109 | 0.4722 | 0.0211 | 3.60 |
| New York | 383944 | 30856 | 19795791 | 1.9395 | 0.1559 | 4.19 |
| Nevada | 11315 | 465 | 2890845 | 0.3914 | 0.0161 | 3.13 |
| Ohio | 41576 | 2404 | 11614373 | 0.3580 | 0.0207 | 3.60 |
| Oklahoma | 8417 | 360 | 3911338 | 0.2152 | 0.0092 | 2.40 |
| Oregon | 5820 | 180 | 4028977 | 0.1445 | 0.0045 | 3.14 |
| Pennsylvania | 83589 | 6243 | 12802503 | 0.6529 | 0.0488 | 3.65 |
| Rhode Island | 16093 | 914 | 1056298 | 1.5235 | 0.0865 | 3.60 |
| South Carolina | 19378 | 602 | 4896146 | 0.3958 | 0.0123 | 3.16 |
| South Dakota | 5928 | 75 | 858469 | 0.6905 | 0.0087 |  |
| Tennessee | 31004 | 482 | 6600299 | 0.4697 | 0.0073 | 3.24 |
| Texas | 90488 | 2097 | 27469114 | 0.3294 | 0.0076 | 3.15 |
| Utah | 14608 | 143 | 2995919 | 0.4876 | 0.0048 | 3.53 |
| Virginia | 54886 | 1552 | 8382993 | 0.6547 | 0.0185 | 3.46 |
| Vermont | 1128 | 55 | 626042 | 0.1802 | 0.0088 |  |
| Washington | 26530 | 1211 | 7170351 | 0.3700 | 0.0169 | 1.97 |
| Wisconsin | 22932 | 694 | 5771337 | 0.3973 | 0.0120 | 3.29 |
| West Virginia | 2322 | 88 | 1844128 | 0.1259 | 0.0048 |  |
| Wyoming | 1079 | 18 | 586107 | 0.1841 | 0.0031 |  |

*^a^States in green issued mask mandate policy during the study period (from 02/01/2020 to 06/15/2020) and states in pink did not. States in grey were excluded from the analysis due to low death count.*

- **Table 7:** Summary of Previous Studies on Estimating NPI Effectiveness of Mask Mandate

| **Author** | **Study Period** | **Model Used** | **Setting** | **NPIs Studied** |
| --- | --- | --- | --- | --- |
| **Bo et al, 2021 (3)** | 1/23/20 – 4/13/20 | Linear Mixed Model | Worldwide | (1) Mandatory face mask in public / (2) Isolation or quarantine (3) Social distancing / (4) Traffic restrictions |
| **Pozo-Martin et al., 2021 (15)** | October – December 2020 | Regression | Worldwide | (1) School closing requirements / (2) Workplace closing requirements / (3) Public events cancelling requirements / (4) Restrictions on gatherings / (5) Public transport restrictions / (6) Stay-at-home requirements / (7) Restrictions on internal travel / (8) International travel controls / (9) Public health information campaigns / (10) Mask wearing requirements / (11) Testing policy / (12) Contact tracing policy |
| **Esra et al., 2020 (7)** | Start of the pandemic -5/2/20 | Linear Mixed Model | Worldwide | (1) Quarantine and isolation policies / (2) Limits on gatherings / (3) School closing (primary, secondary and tertiary educational institutions) / (4) Mask policies / (5) Household confinements |
| **Jalali et al., 2020 (10)** | 3/1/20 - 5/31/20 | Kruskal-Wallis tests and Correlation Tests | USA, 30 most populous counties and 10 most populous counties of CA, FL, NY and TX | 3 broad categories: (1) Restrictions on mass gatherings / (2) Stay-at-home orders / (3) Face mask requirements |
| **Leffler et al., 2020 (11)** | Early Outbreak in 2020 | Regression | Worldwide | (1) School closing / (2) Workplace closing / (3) Cancel public events / (4) Restrictions on gatherings / (5) Close public transport / (6) Stay-at-home requirements / (7) Internal movement restrictions / (8) International travel restrictions / (9) Income support / (10) Public information campaigns / (11) Testing policy / (12) Contact tracing policy / (13) Public wearing of masks |
| **Zhang et al., 2021 (18)** | February - August 2020 | Regression | US, all states | (1) Implementation of shutdowns / (2) Mask mandates |

- **Table 8:** Bayesian hierarchical model comparisons

| Bayesian Hierarchical Framework | Prototypical Model  by Flaxman et al., 2020 (8) | Counterfactual Model  by Mishra et al., 2021 (13) | Advanced Model  by Banholzer et al., 2021 (2) |
| --- | --- | --- | --- |
| Pros | Effective in restoring the reproduction number (Rt) in each state over time  Using indicator variables to reflect the NPI implementation status in each state | Comparing the result that actually happened with the result which would happen under different situation but didn’t happen.  Switching the NPI portfolios in different regions and provide counterfactual results | Introducing a time-delayed response function, allowing a few days to let the NPIs become fully effective.  Using daily observed case data to allow “outlier states” to be included into the analysis. |
| Cons | States with low death counts were excluded since the model use observed death data  Assuming fully effective once NPI was being implemented, which is unlikely to be true in reality. | Assuming the population of each group would respond in the same way to the same intervention | Assuming definition of a confirmed case and the reporting practice are the same among states  When NPIs in a state were implemented concurrently, it’s hard to distinguish between the effects of single NPI. |

- **Table 9:** Summary of previous studies using Bayesian hierarchical models

| **Author** | **Model Used** | **Region Studied** | **Time Frame** |
| --- | --- | --- | --- |
| Flaxman et al., 2020 (8) | Prototypical Model | 11 European Countries | Feb 2020 until May 4^th^, 2020 |
| Olney et al., 2021 (14) | Prototypical Model | United States, all states | Feb 29^th^ to Apr 25^th^, 2020 |
| Sharma et al., 2021 (16) | Prototypical Model | 7 European Countries | Between Aug 2020 and Jan 2021 |
| Mishra et al., 2021 (13) | Counterfactual Model | United Kingdom, Sweden, and Denmark | Mar 13^th^ to Jul 1^st^, 2020 |
| Banholzer et al., 2021 (2) | Advanced Model | 15 European Countries, Norway, Switzerland, Australia, United States, and Canada | Between Feb and May 2020 |

- **Figure 1:** U.S. Map – States Included (green) and Excluded (grey) from Analysis 1
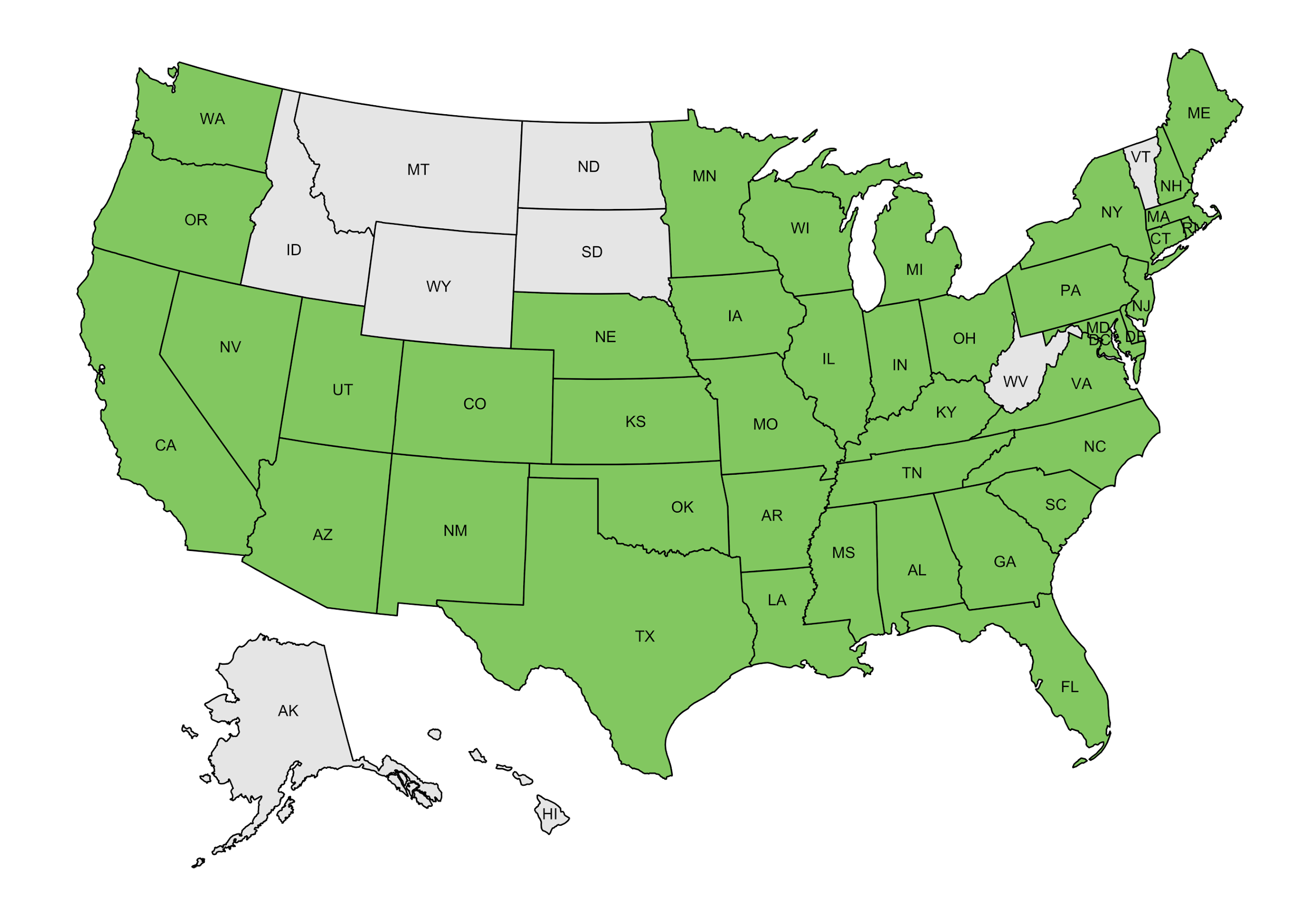

- **Figure 2:** US map of facemask mandate policy during the study period. States highlighted in green initiated facemask mandate policy during the study period (February 1 to June 15, 2020) and states highlighted in pink did not introduce facemask mandate policy. States in grey had very low death count which cumulatively less than 100 by the end of the study
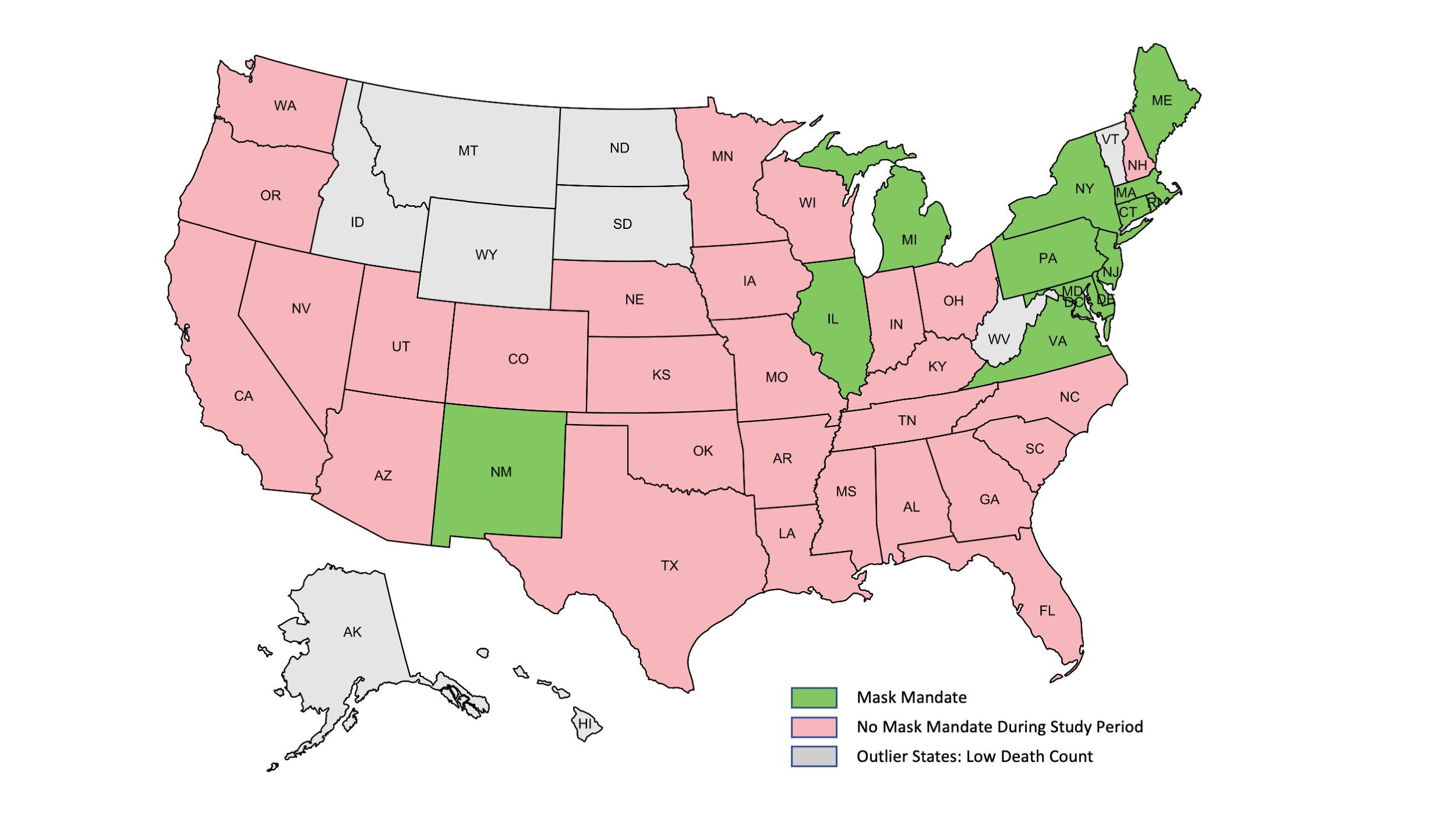
 period, indicating COVID-19 was not prevalent in these states and thus excluded from the analysis.
- **Figure 3:** Frequency of at least $m$ Non-Pharmaceutical Interventions have positive effects (in yellow) and greater than 10% (in green)


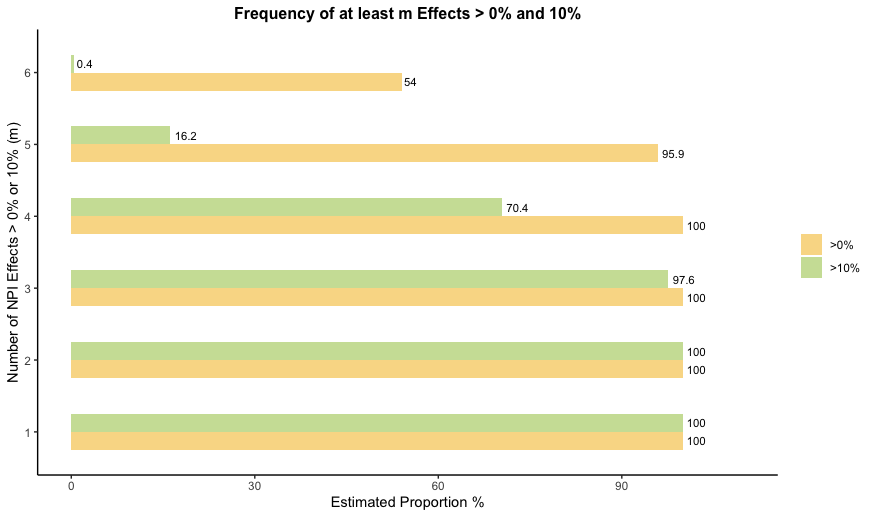


### **Figure List 1:** Prototypical Bayesian Hierarchical Model Rt estimation over the Study Period


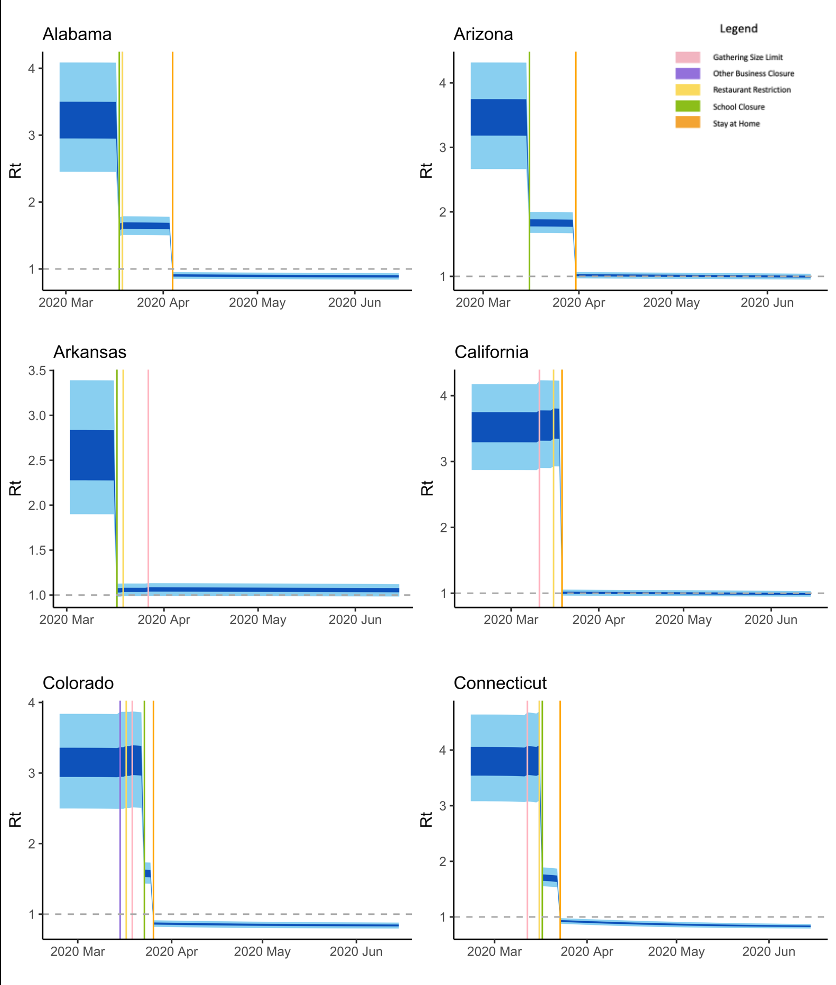


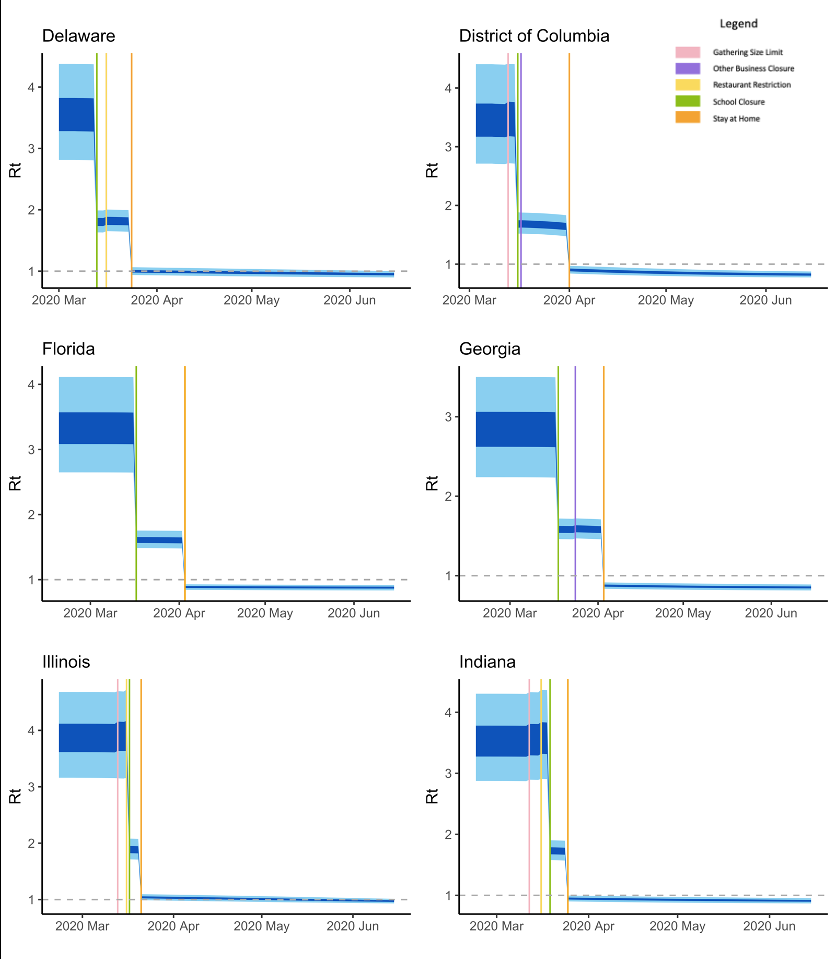


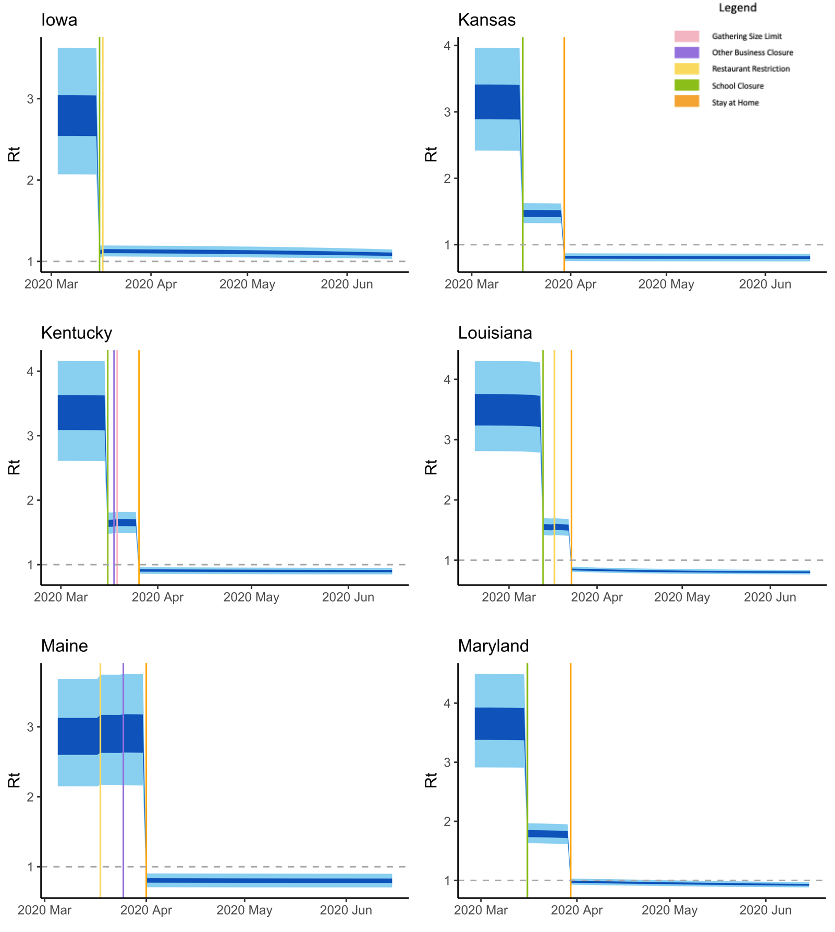


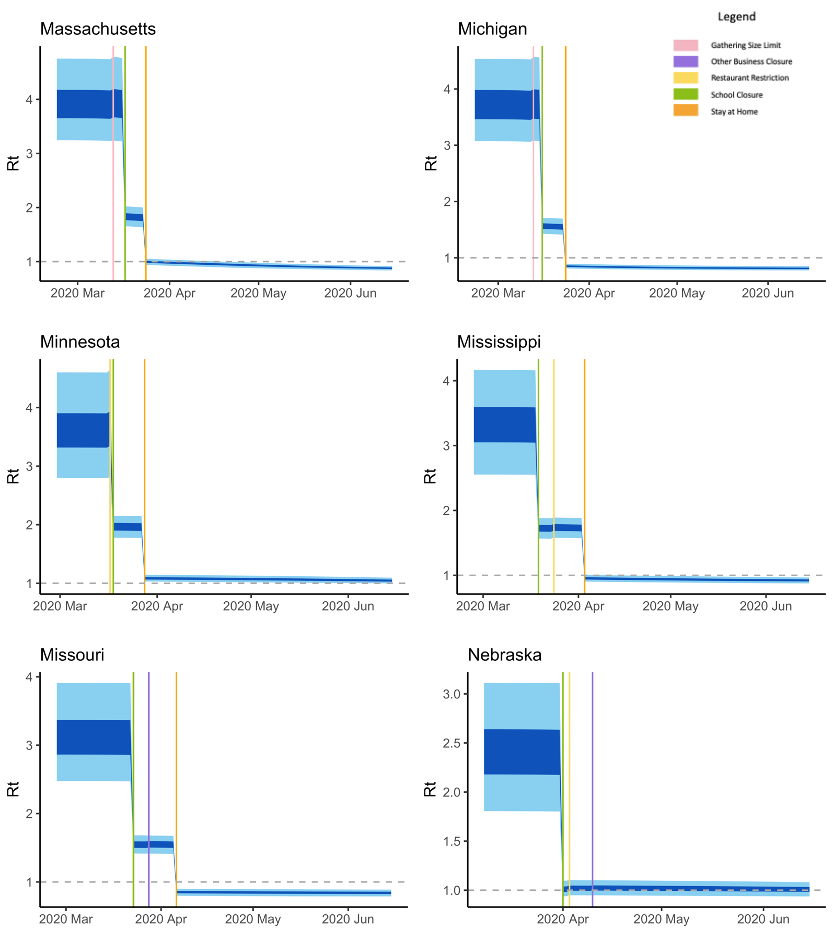


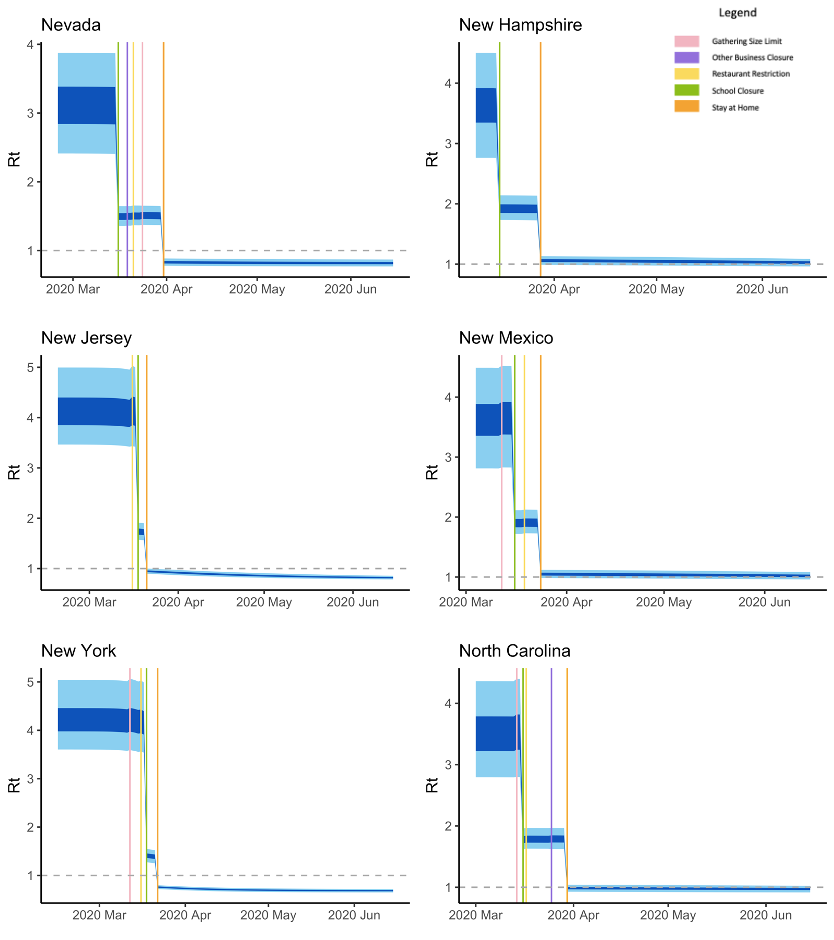

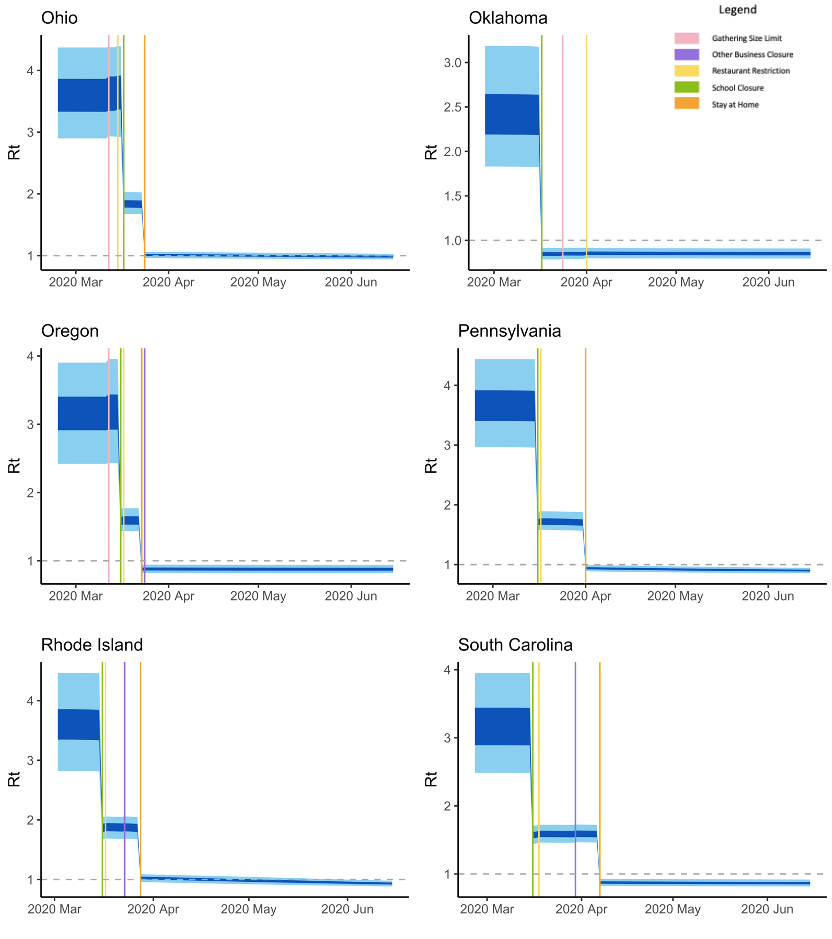


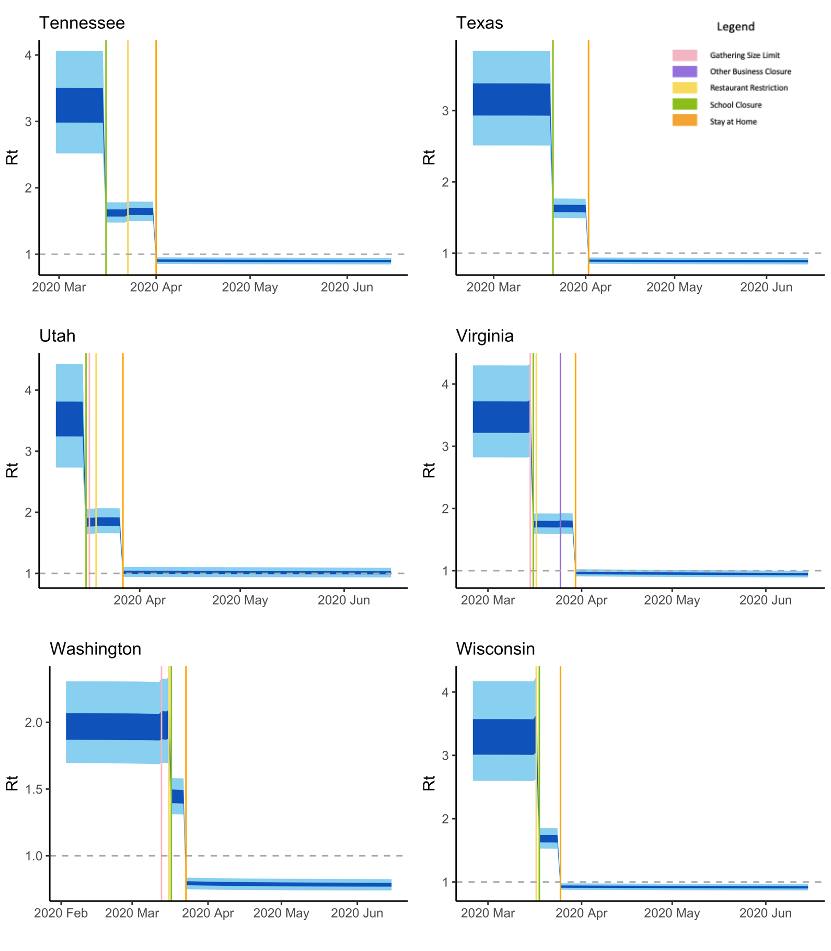


### **Figure List 2:** Prototypical Model Expected Daily Number of Deaths Estimation Over the Study Period by States:


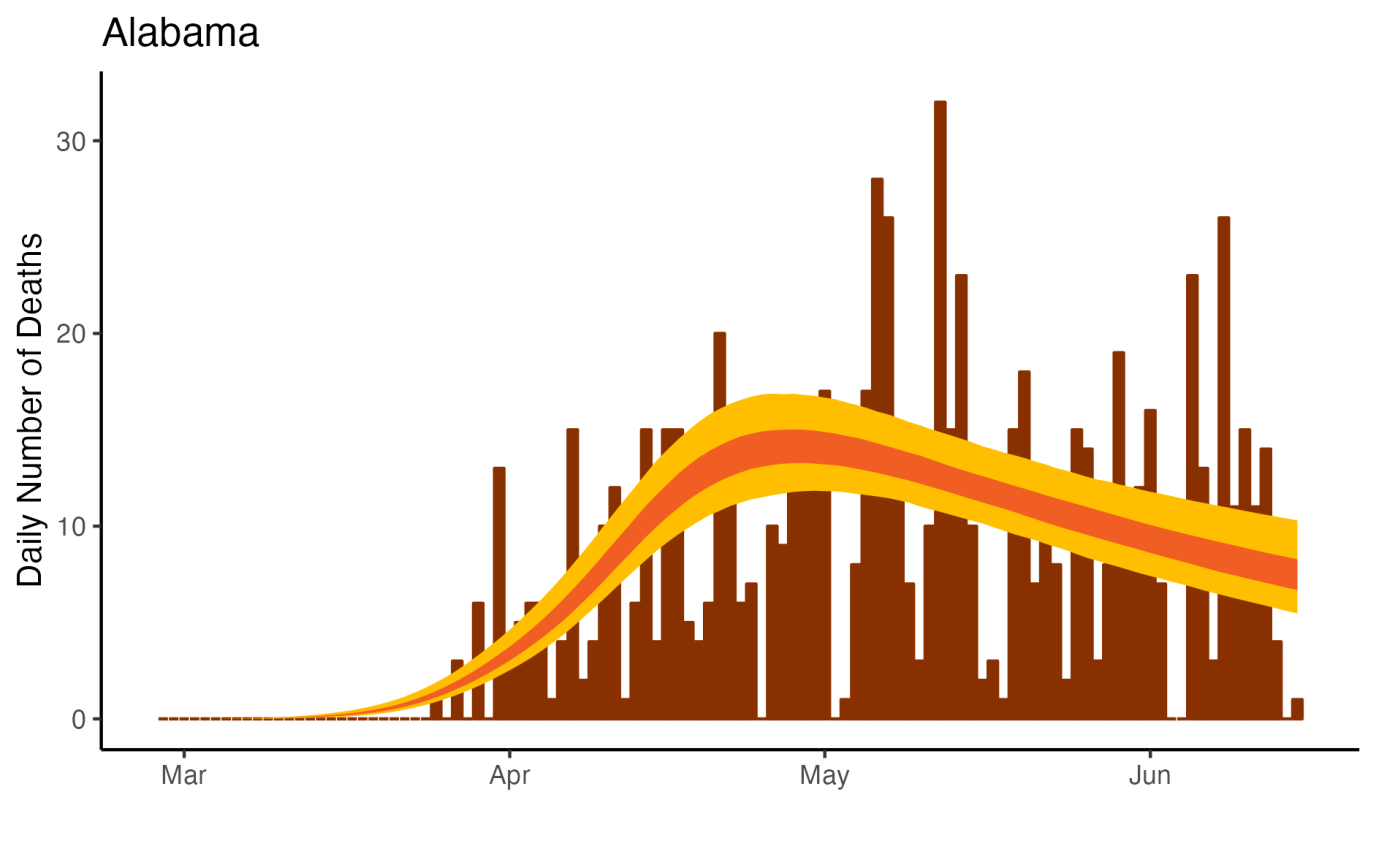

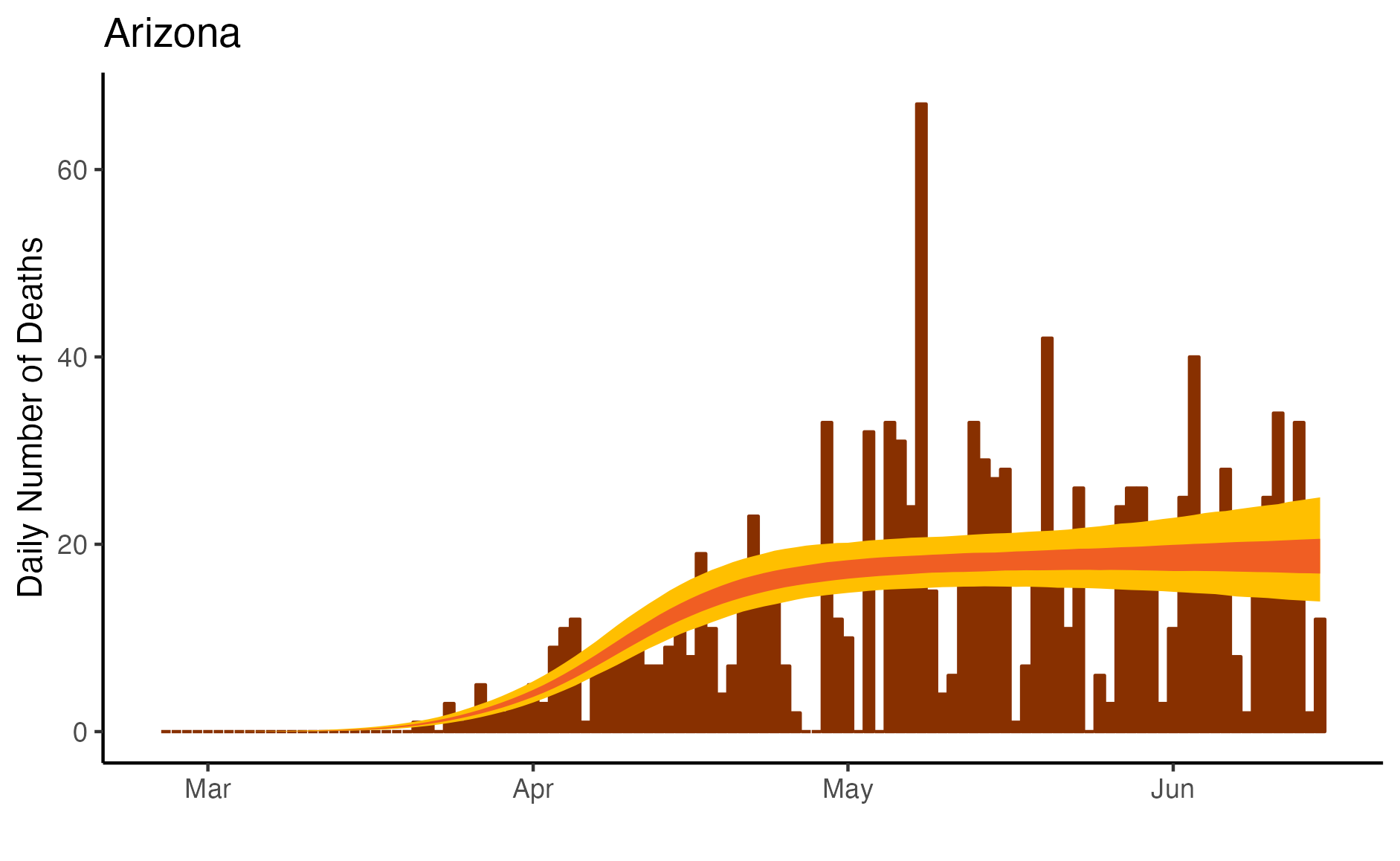

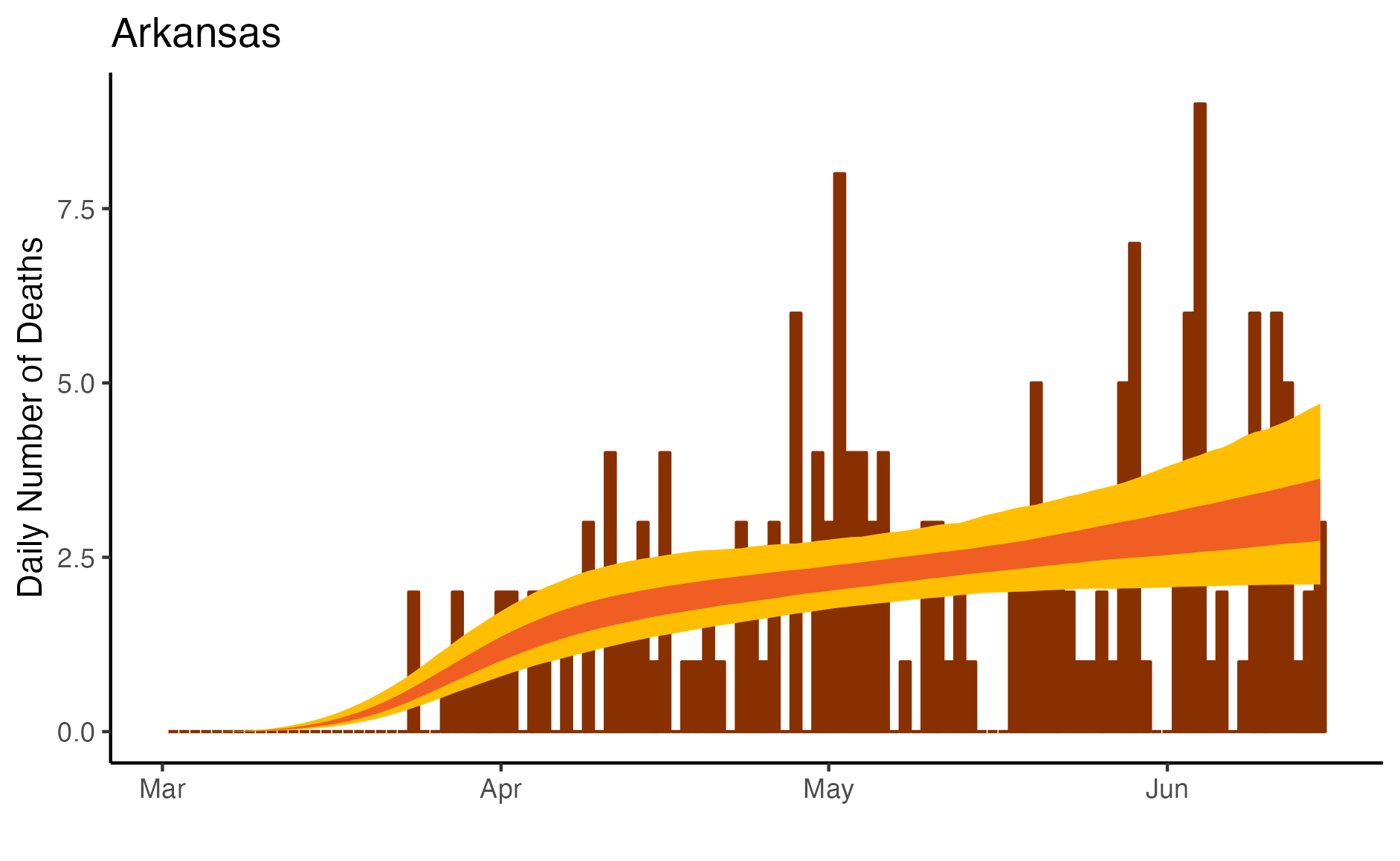

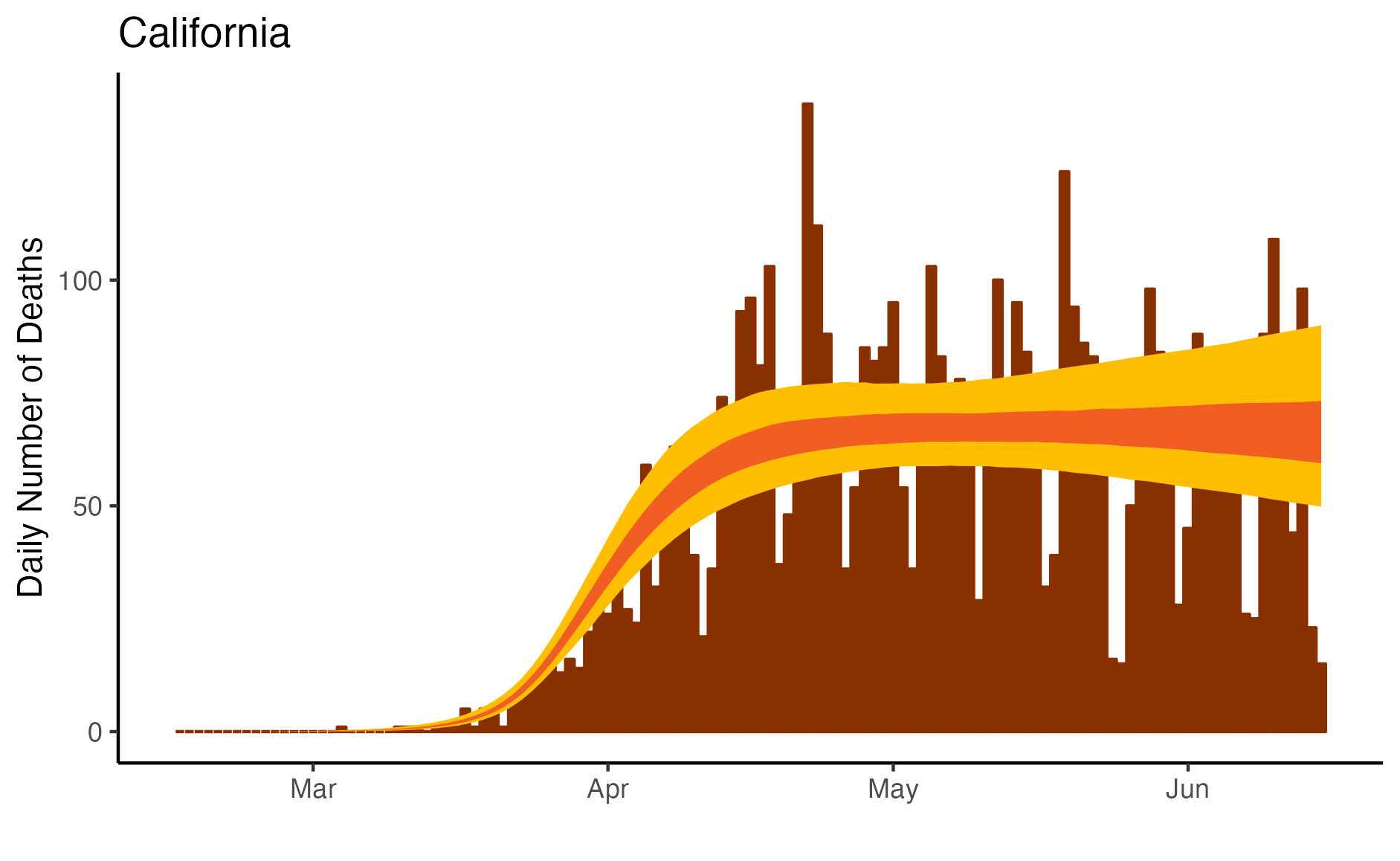

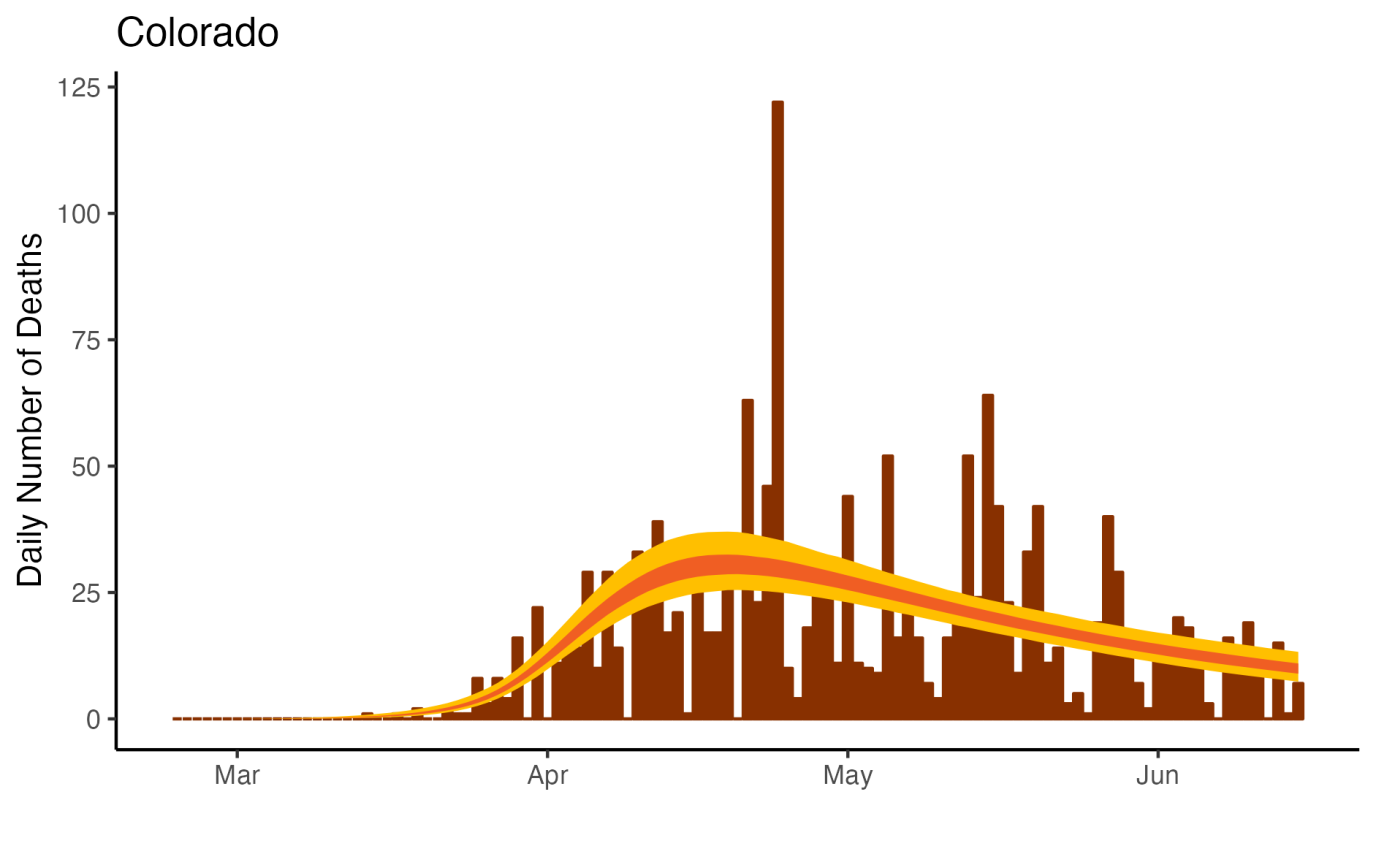

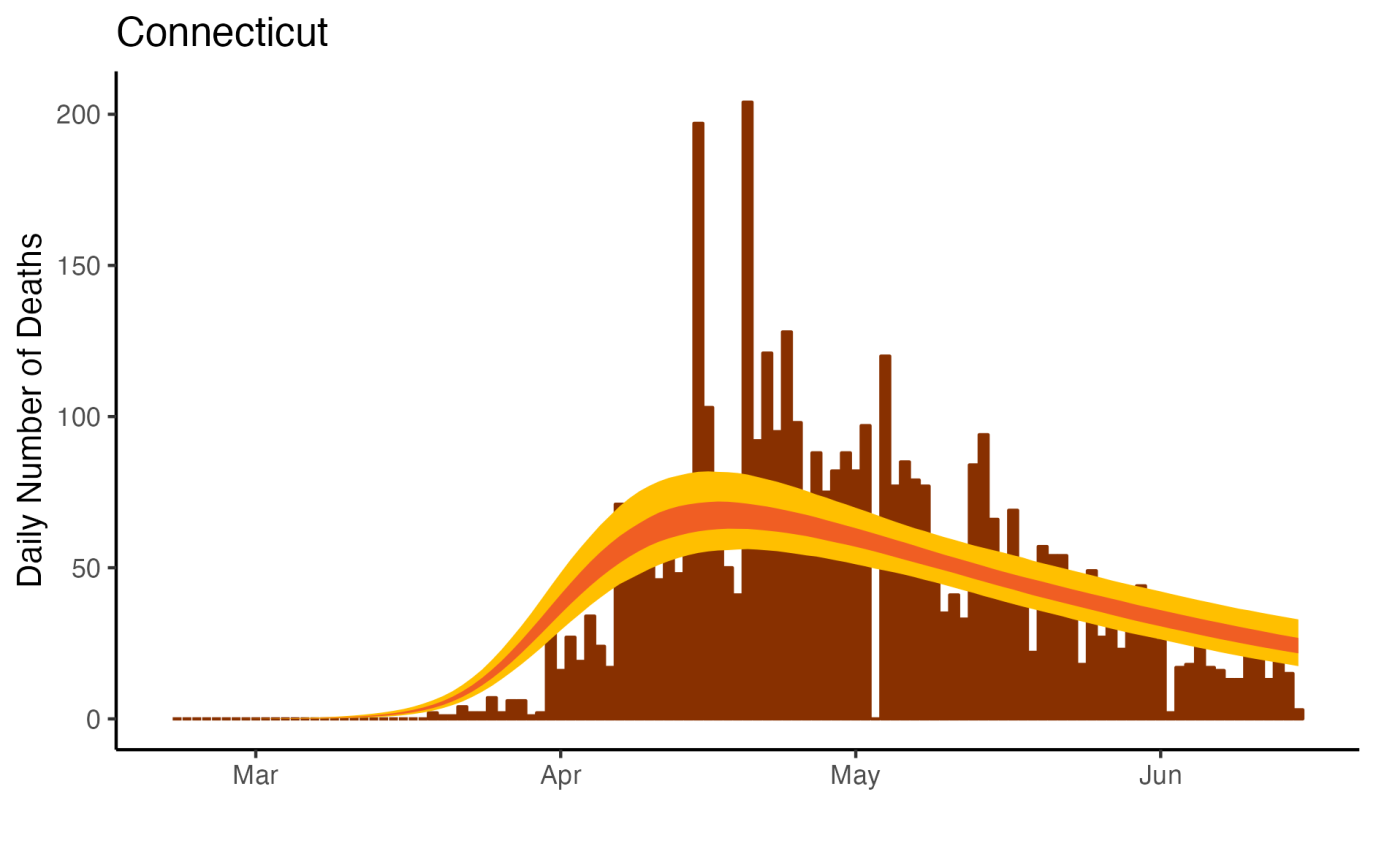

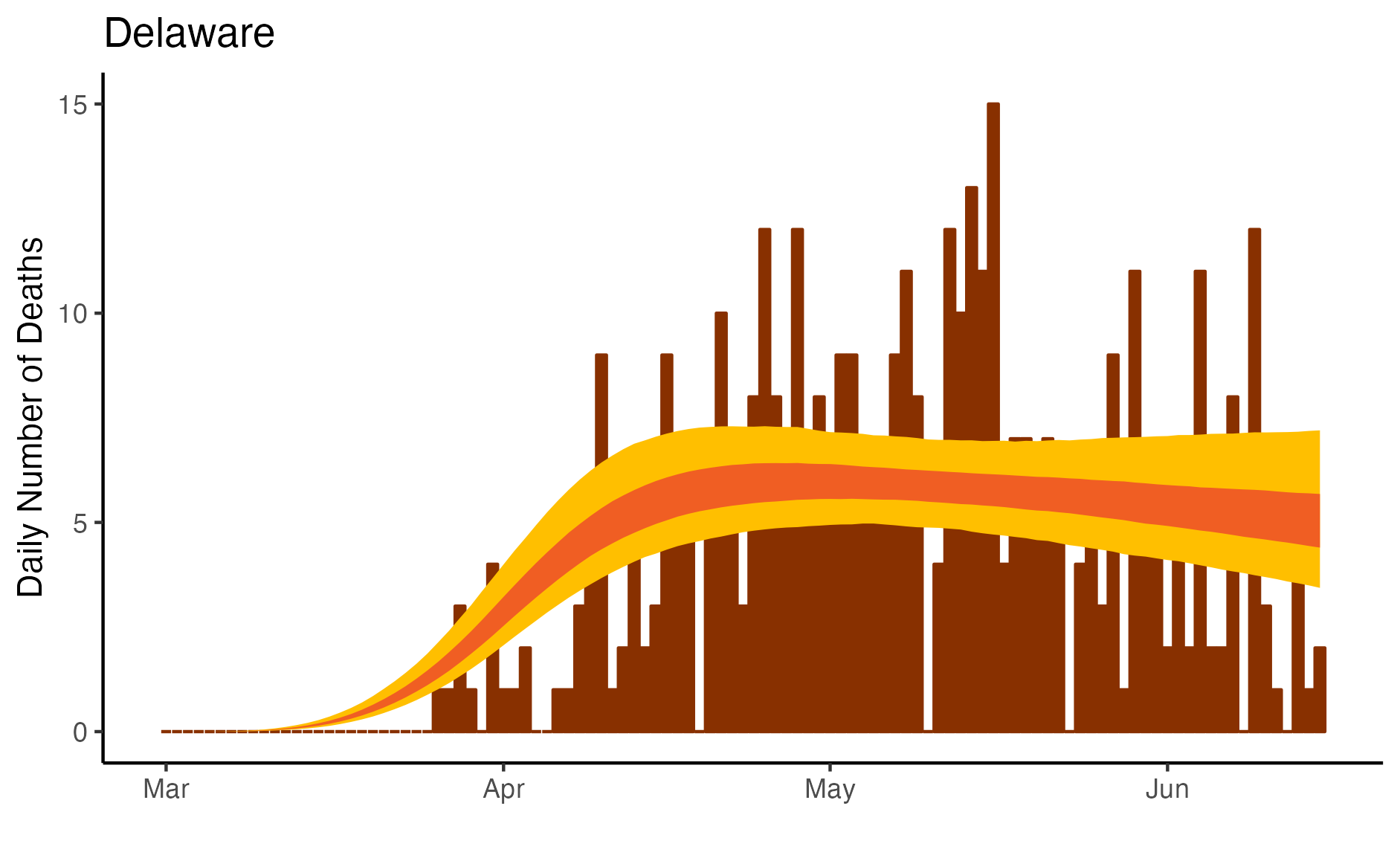

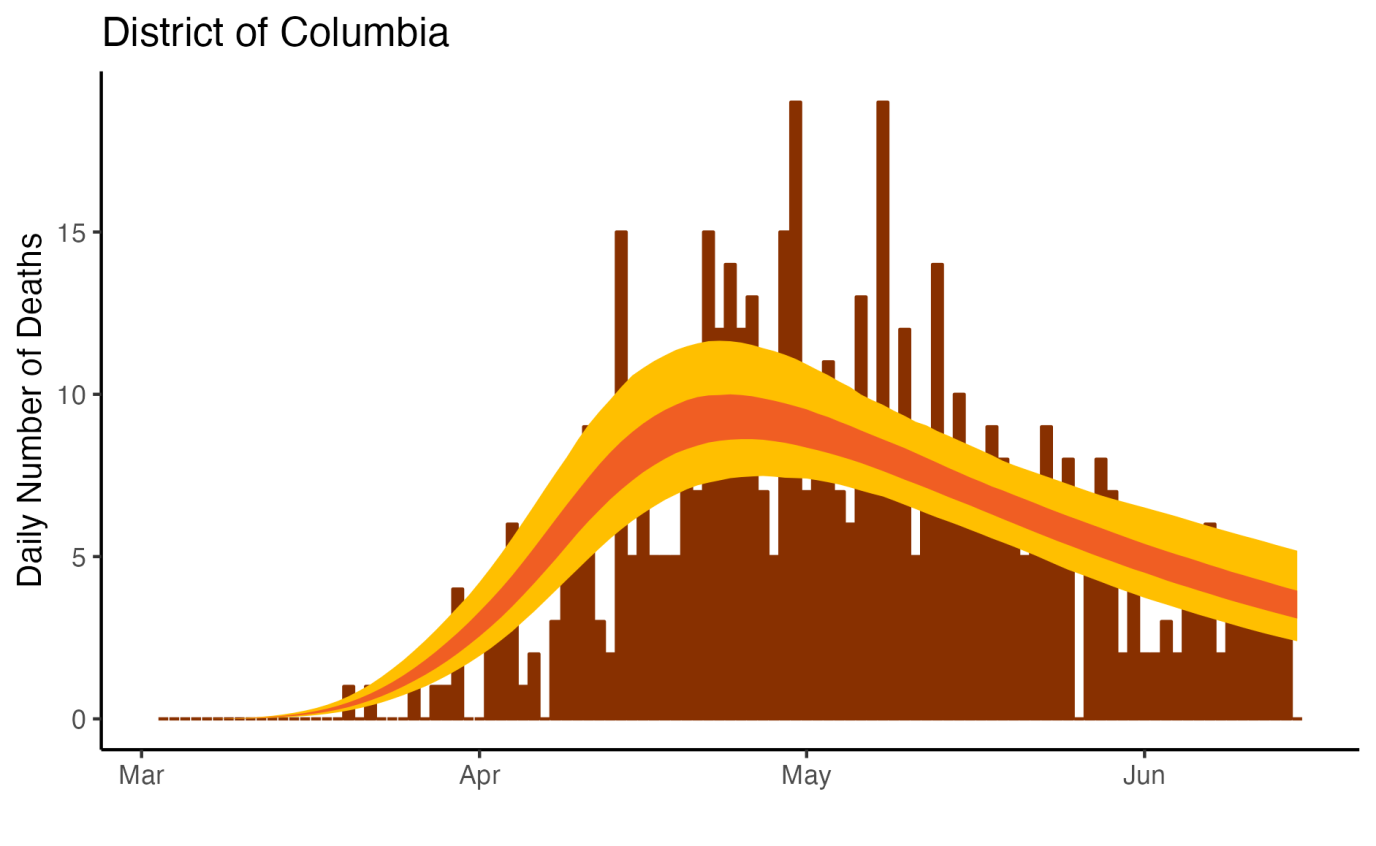

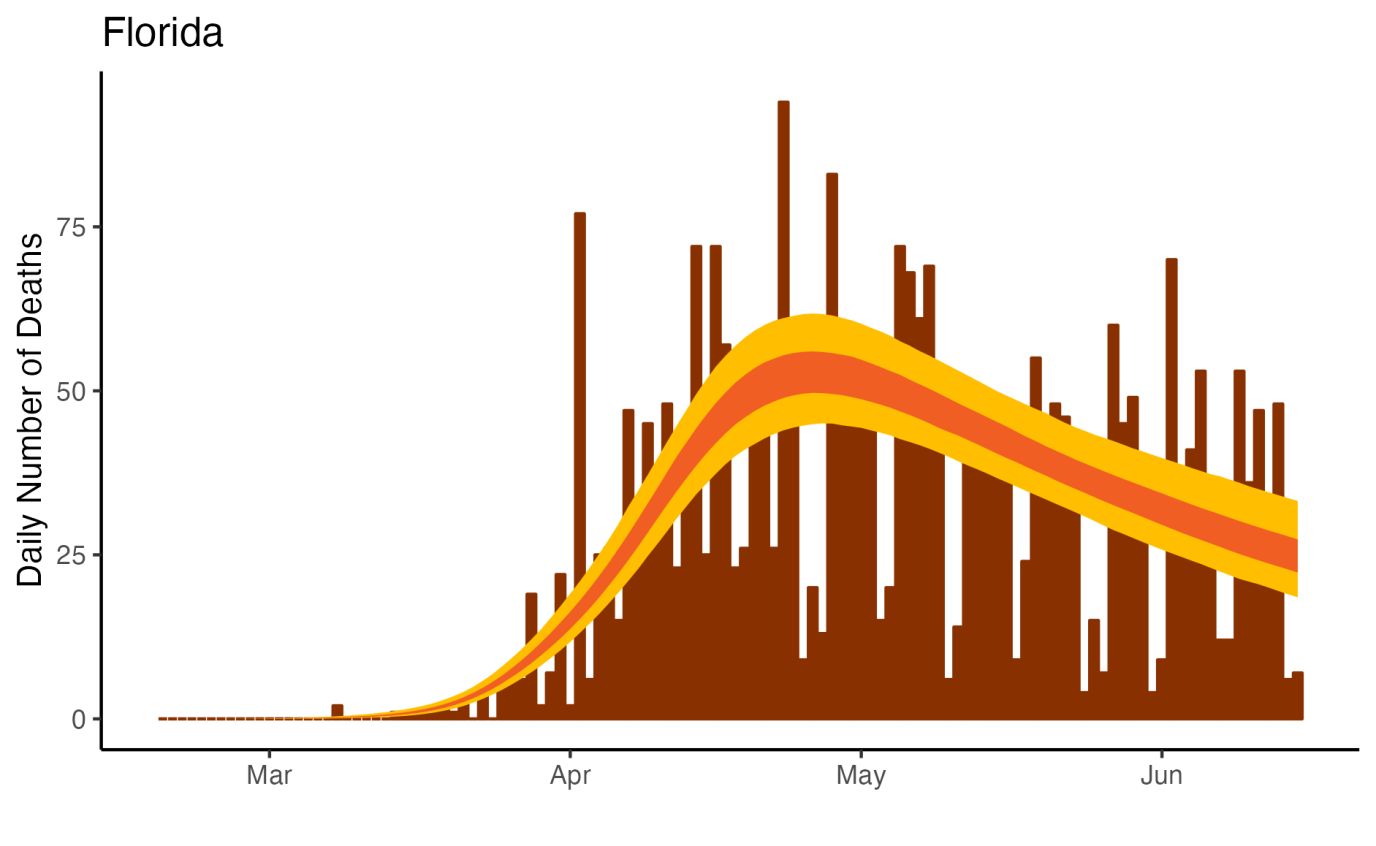

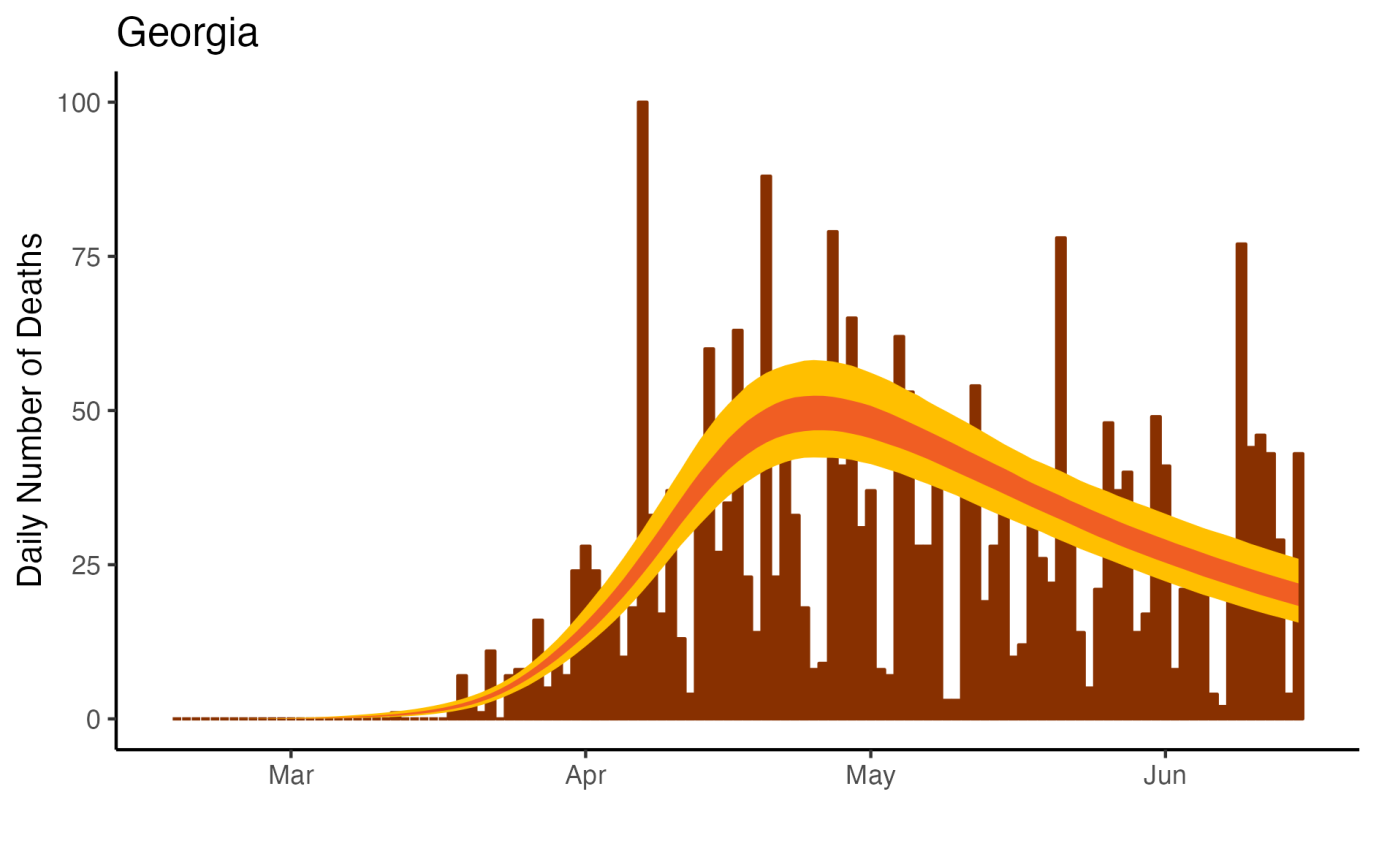

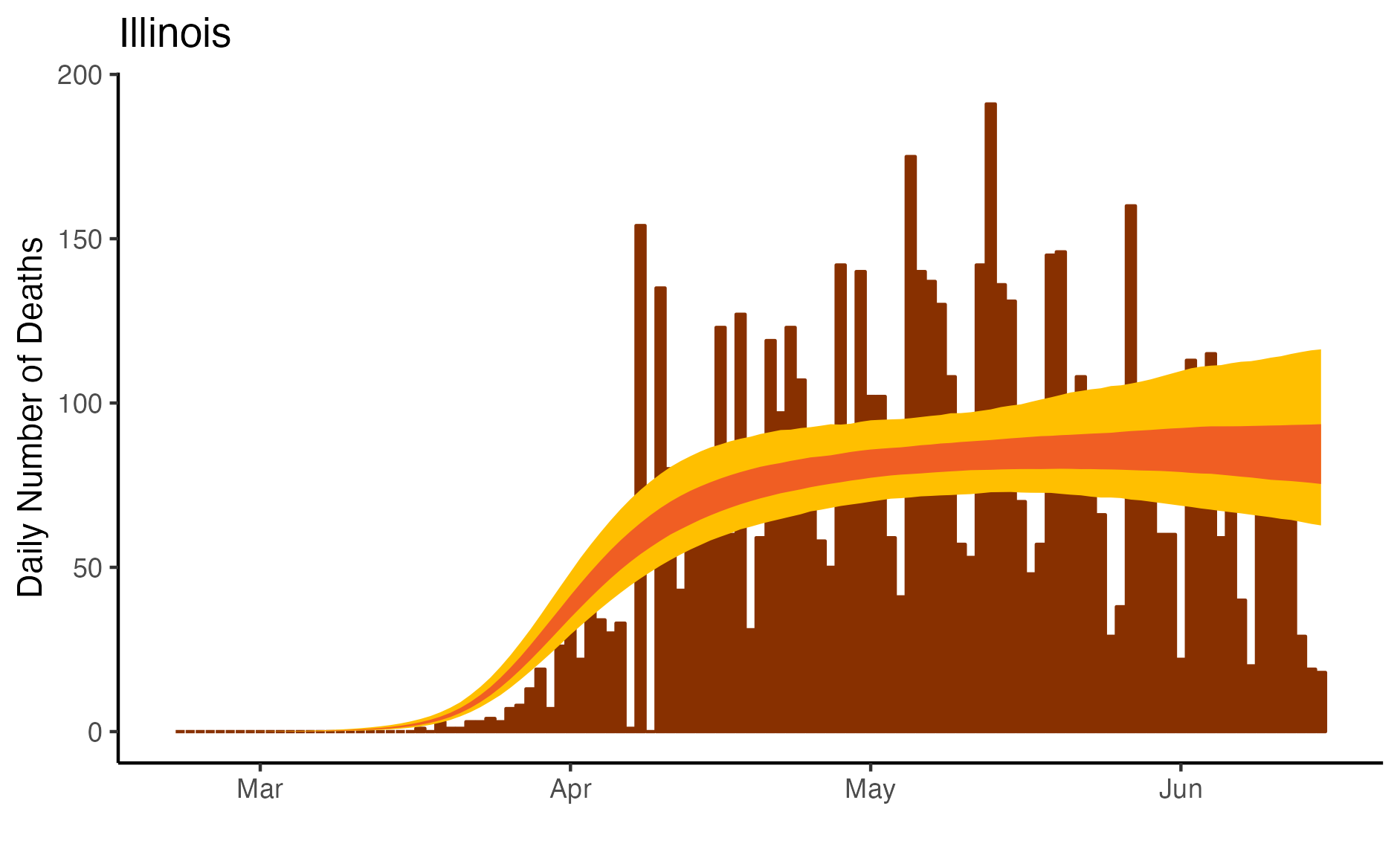

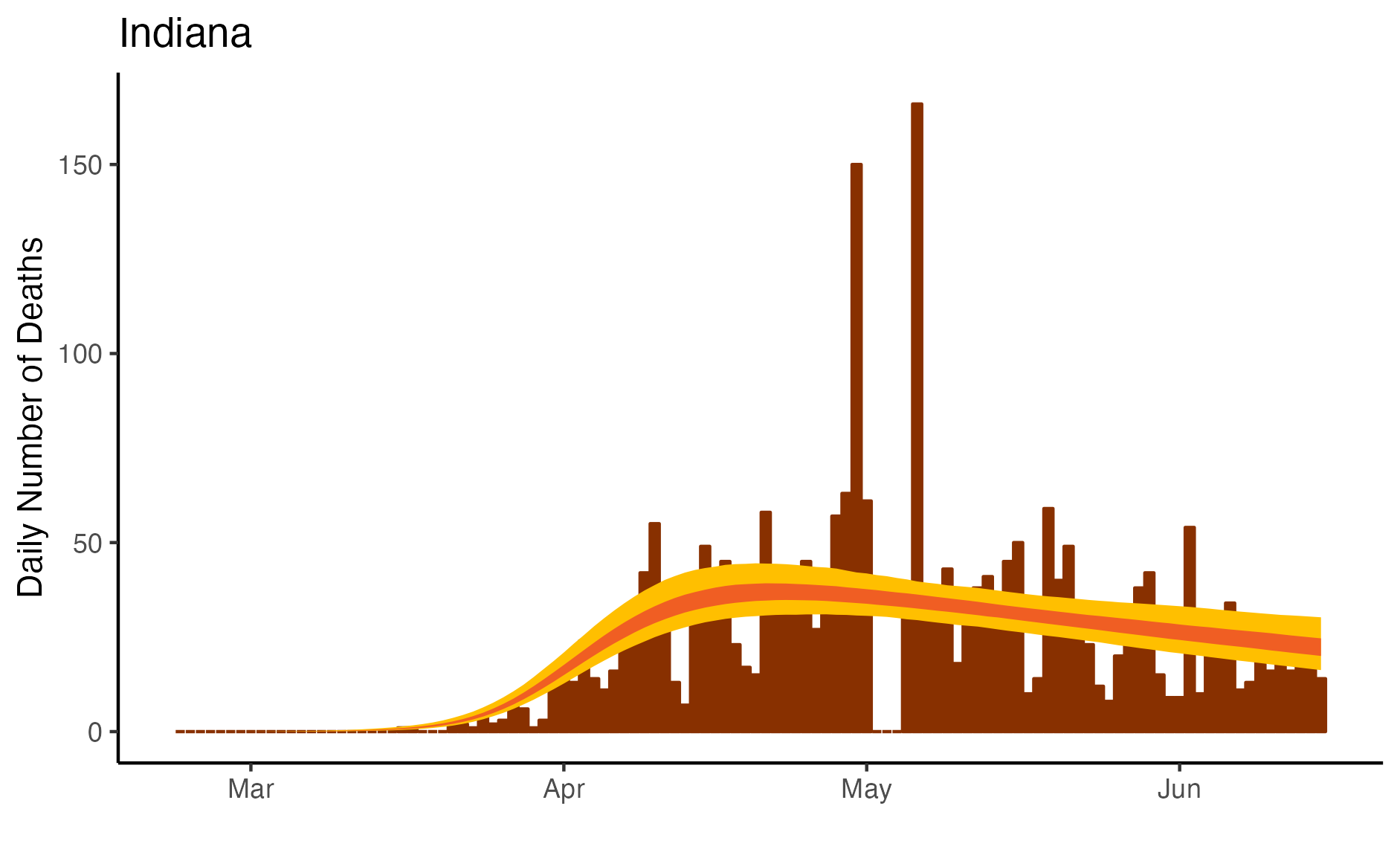

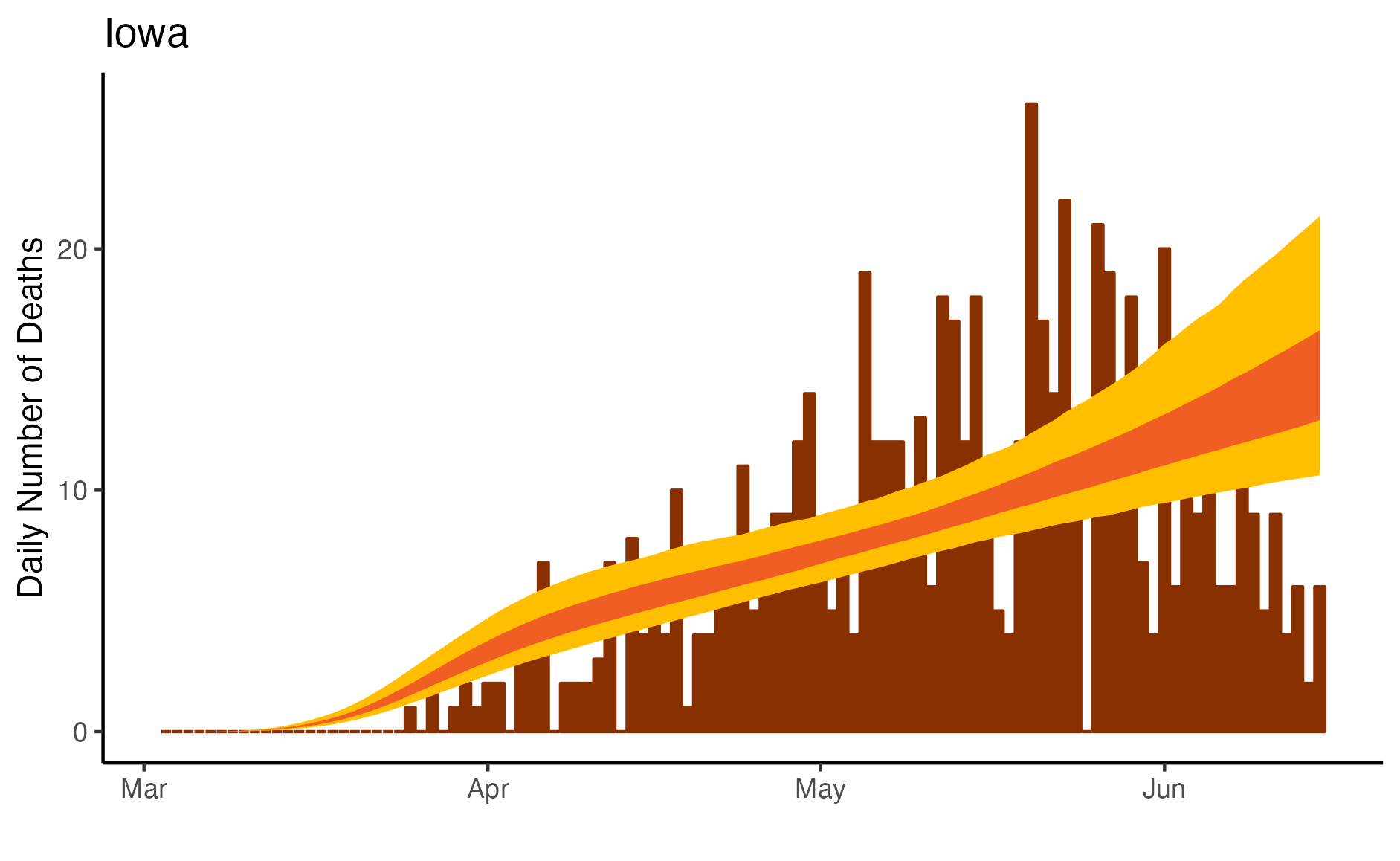

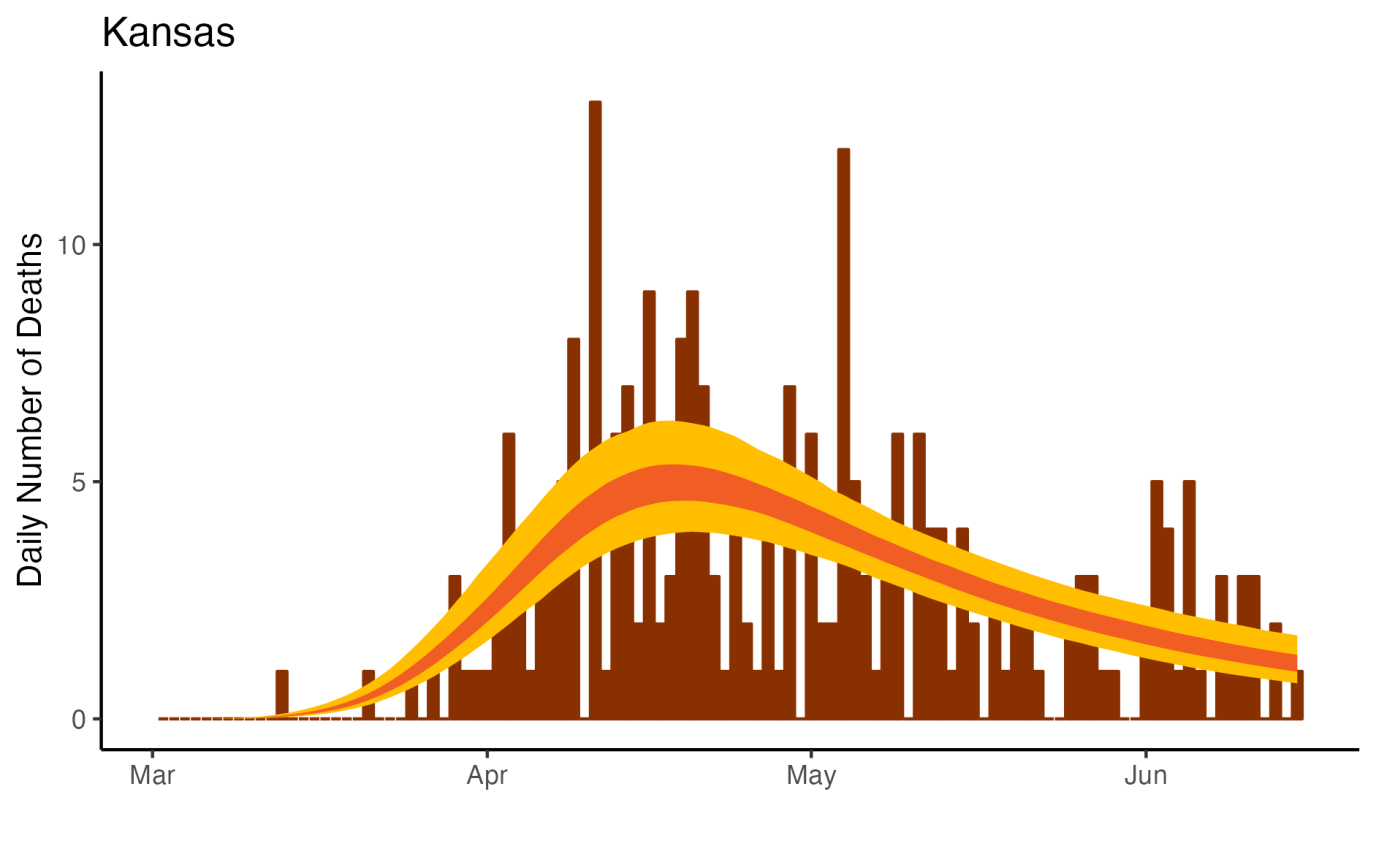

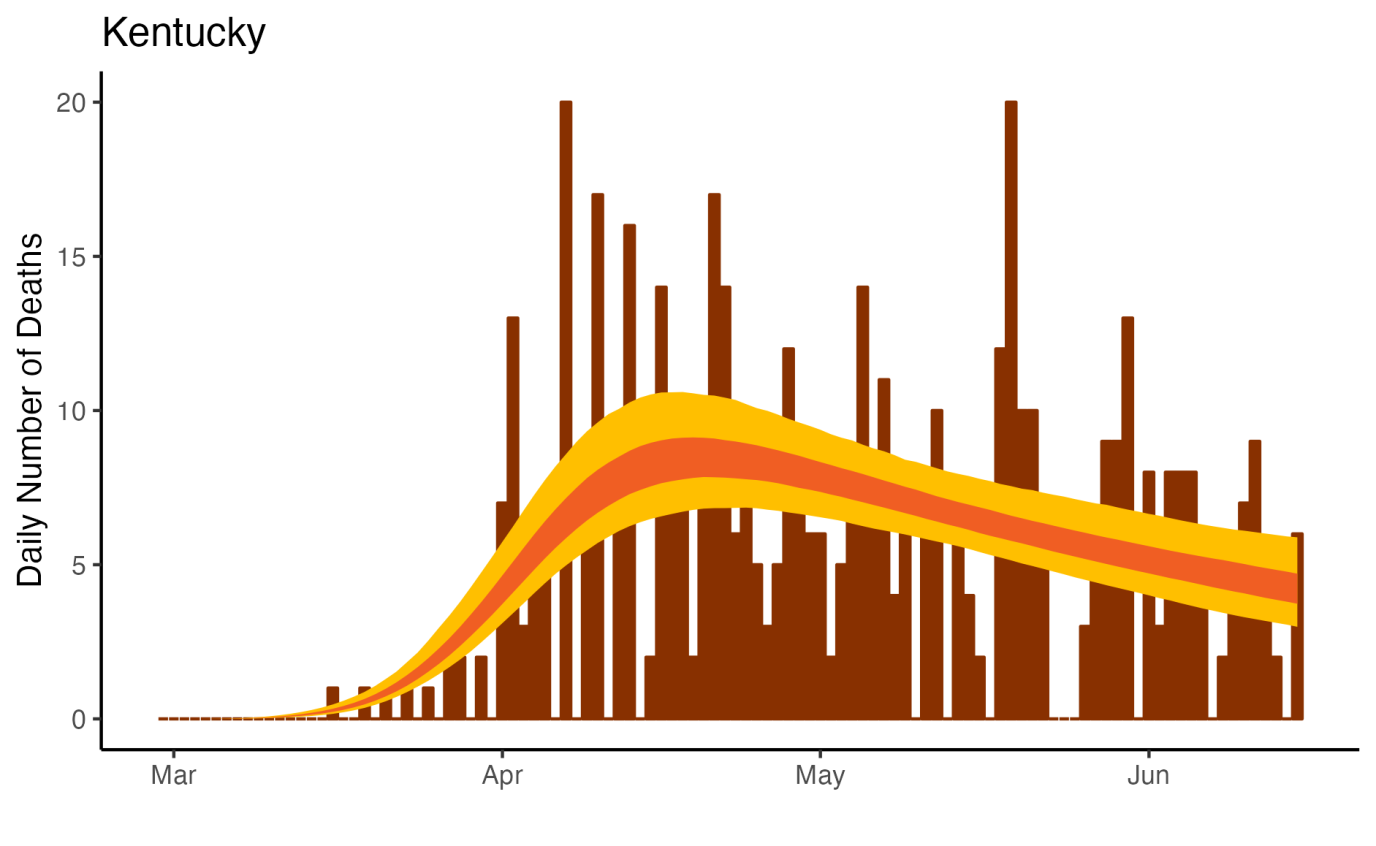

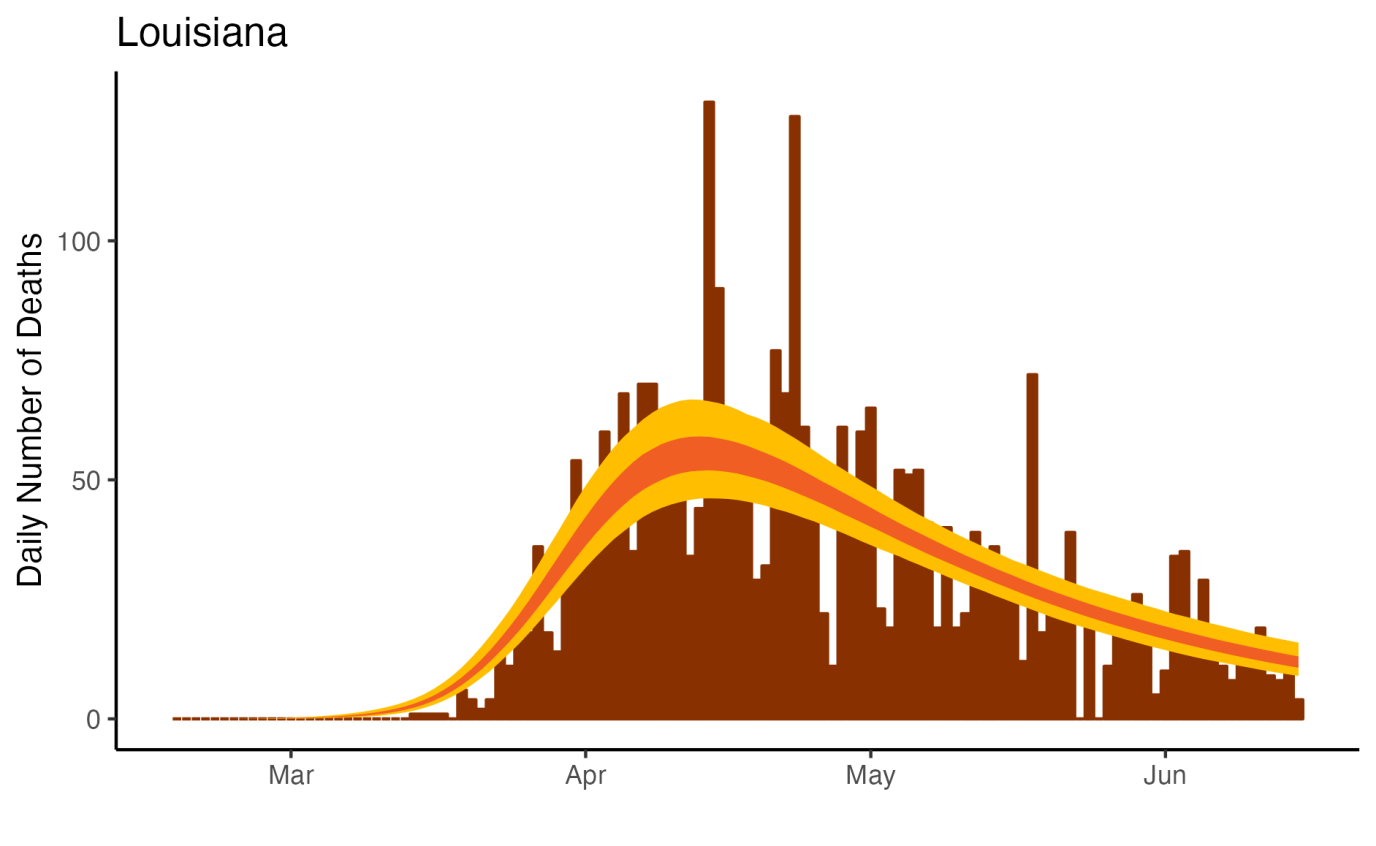

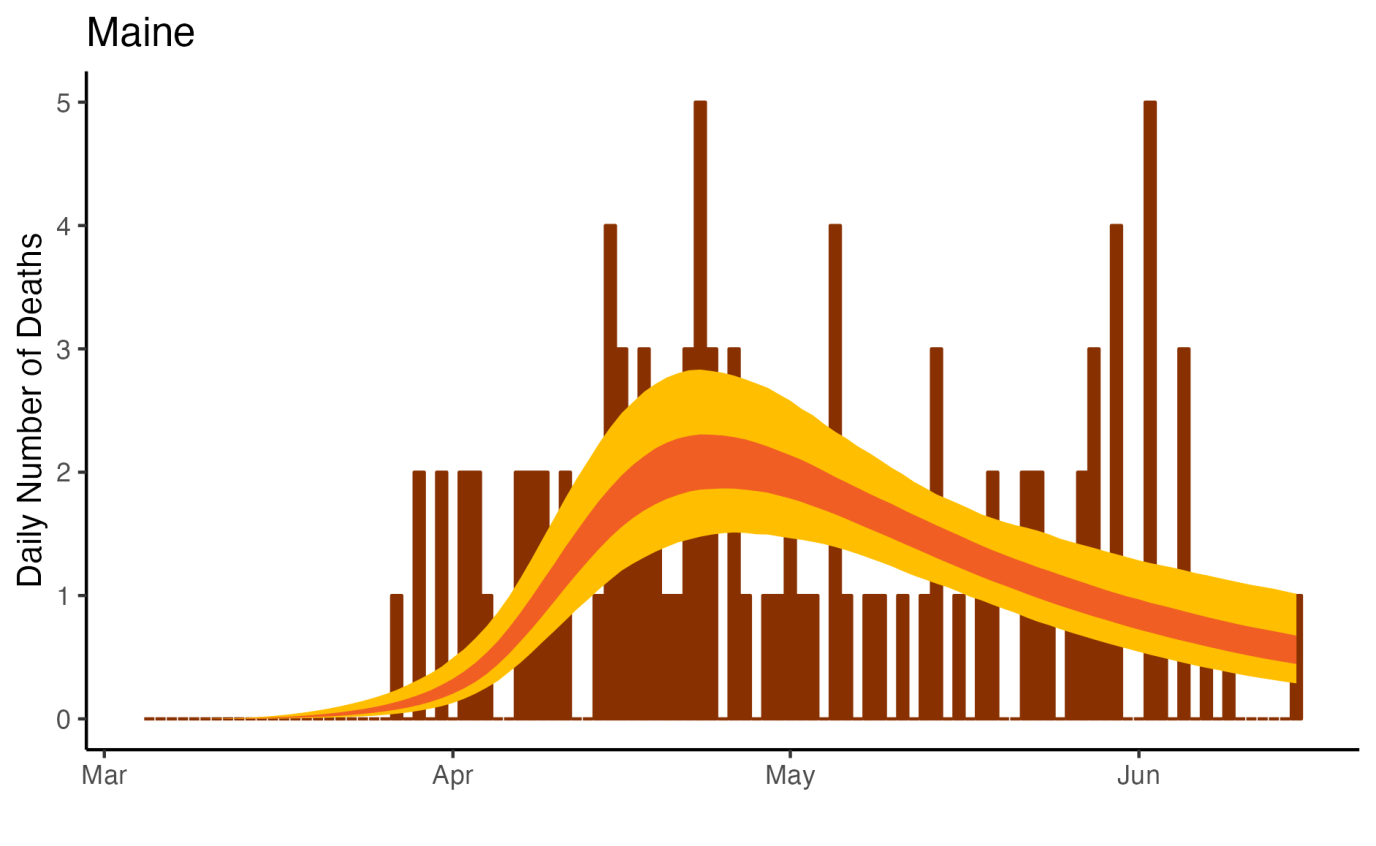

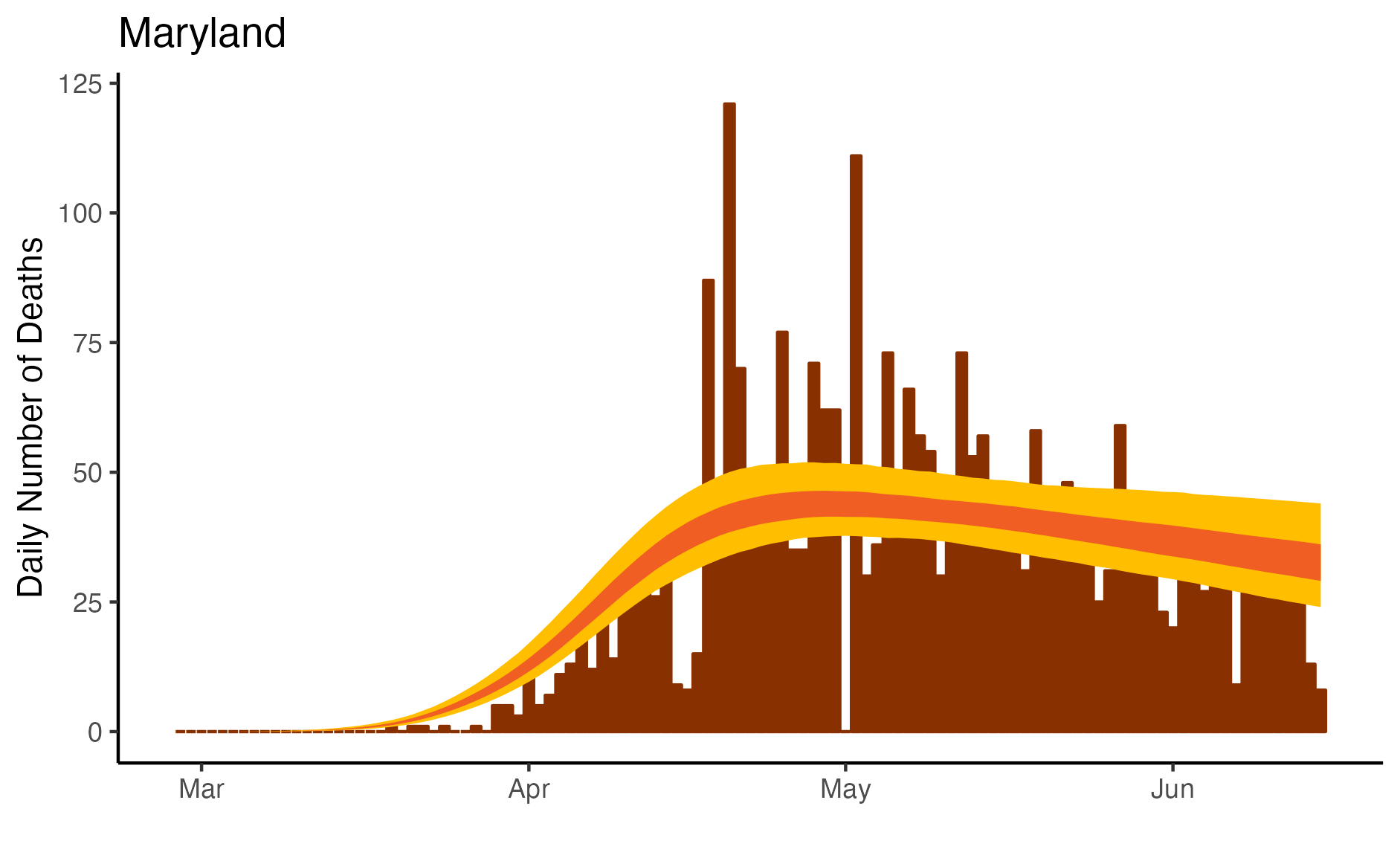

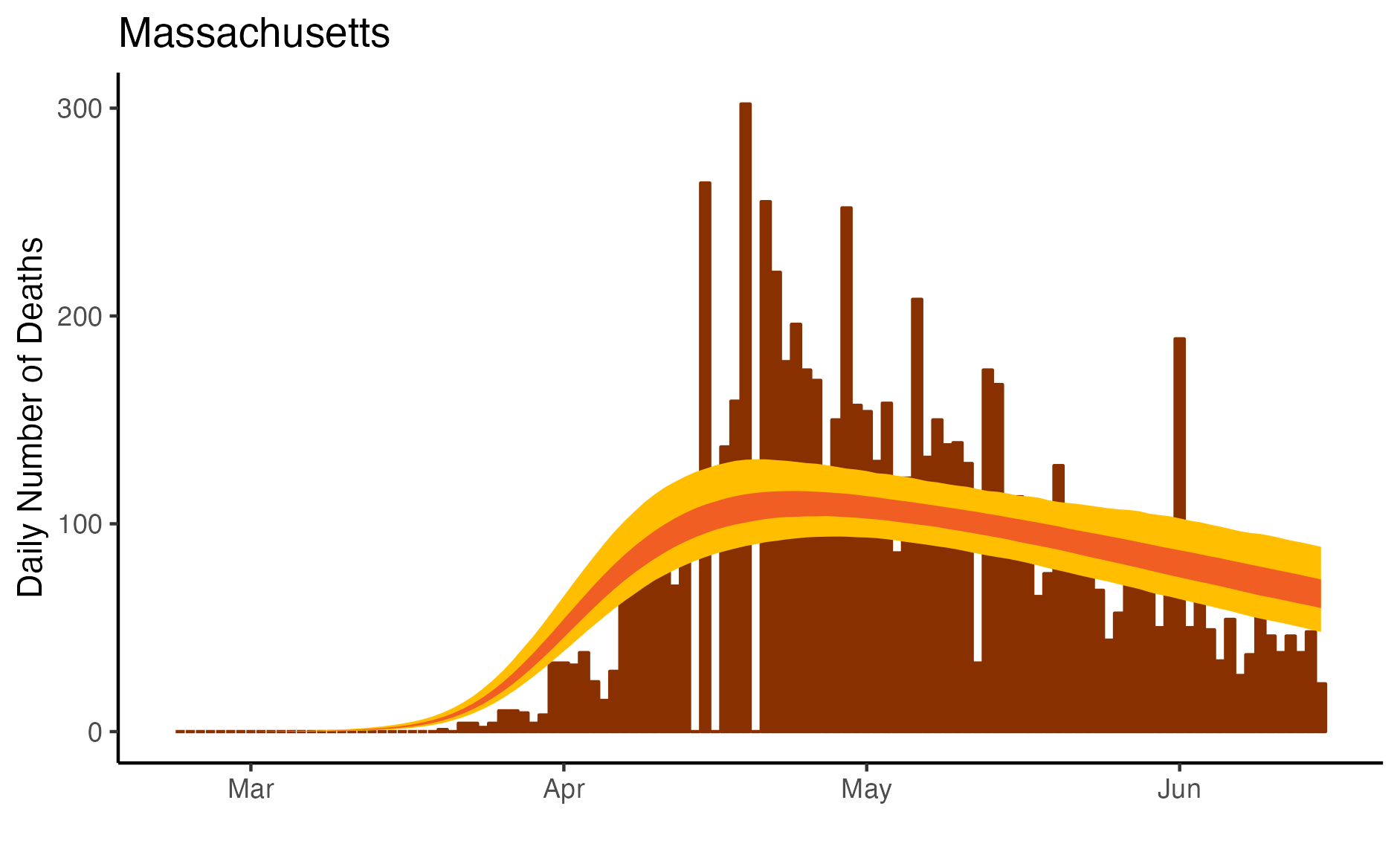

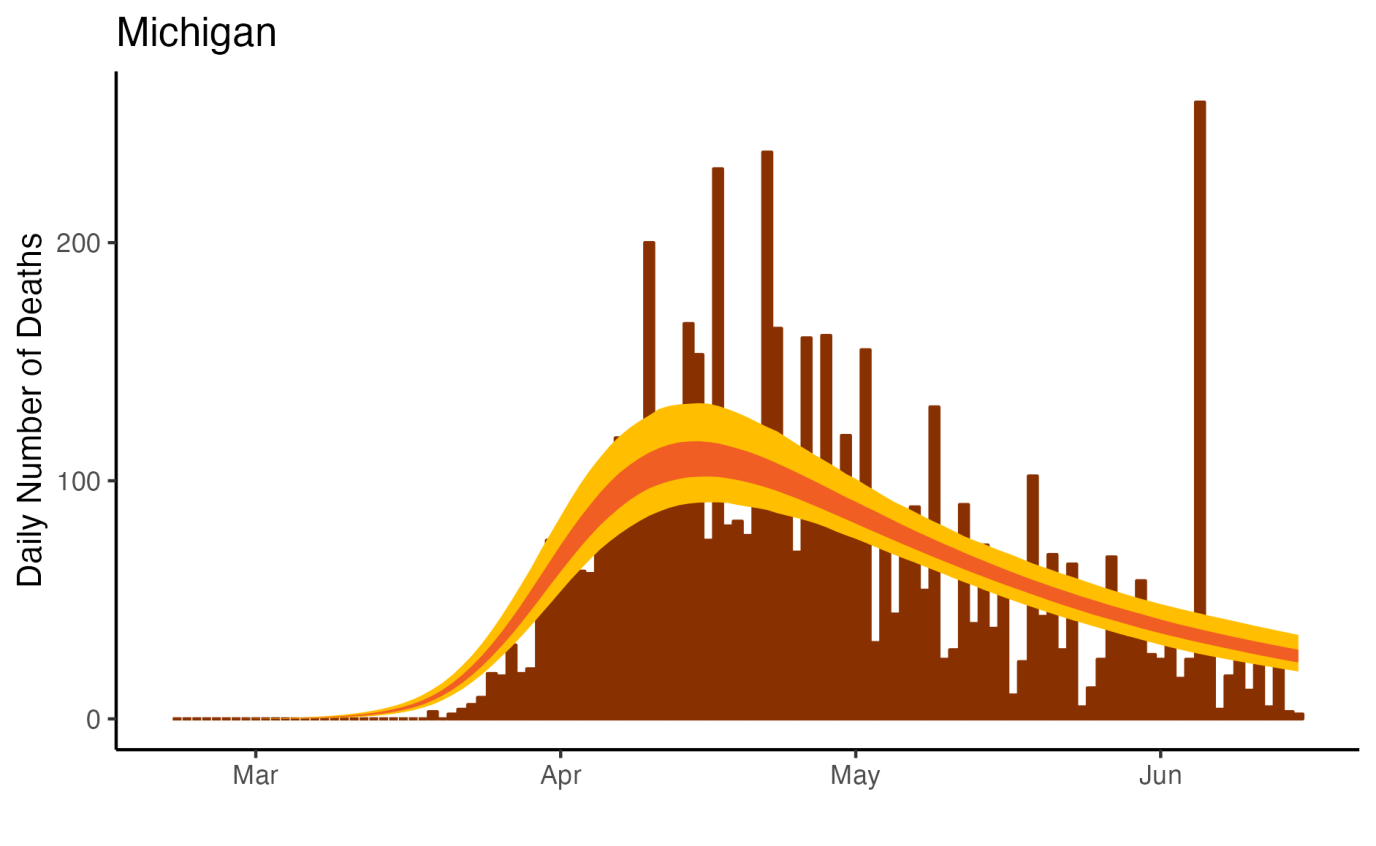

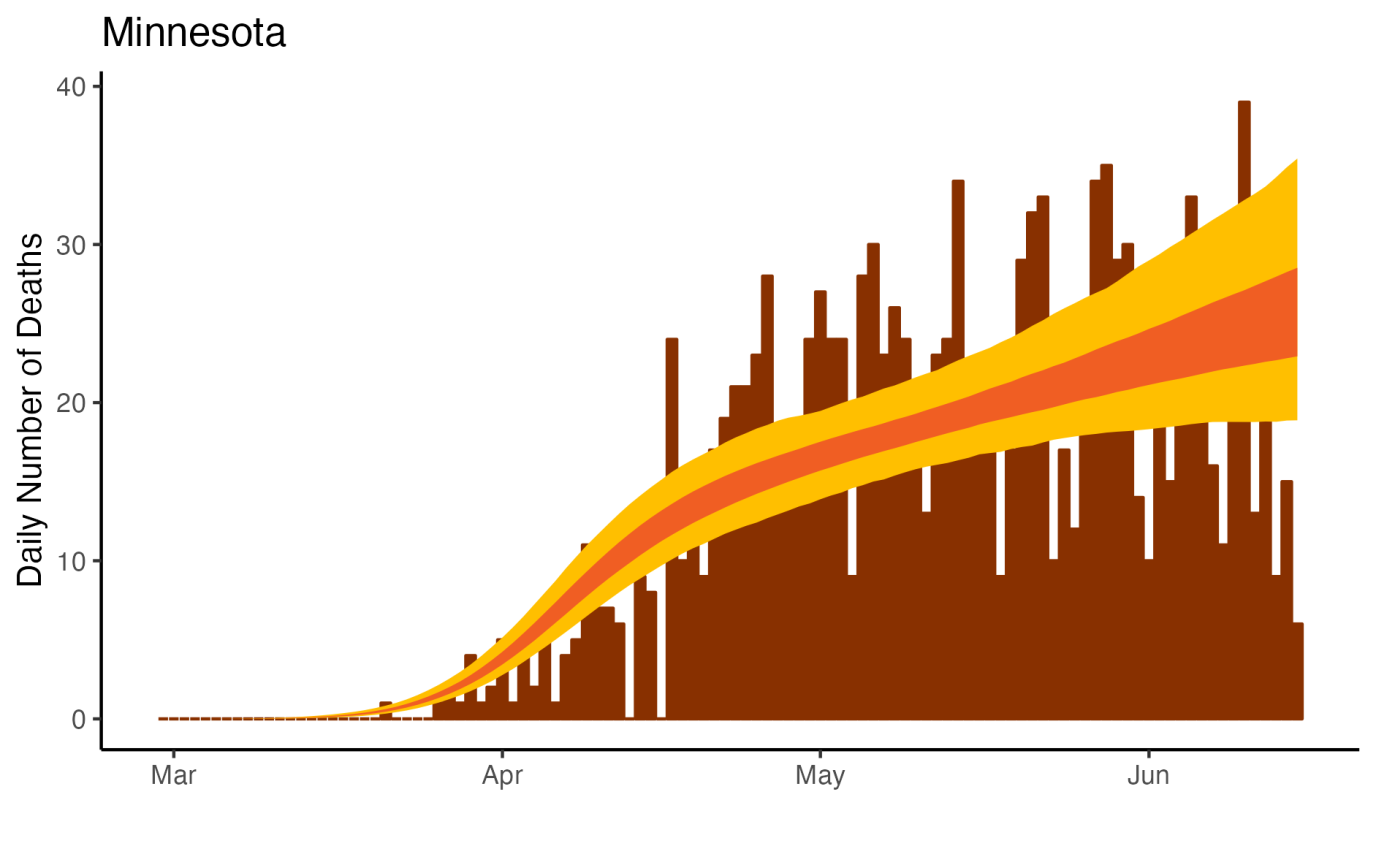

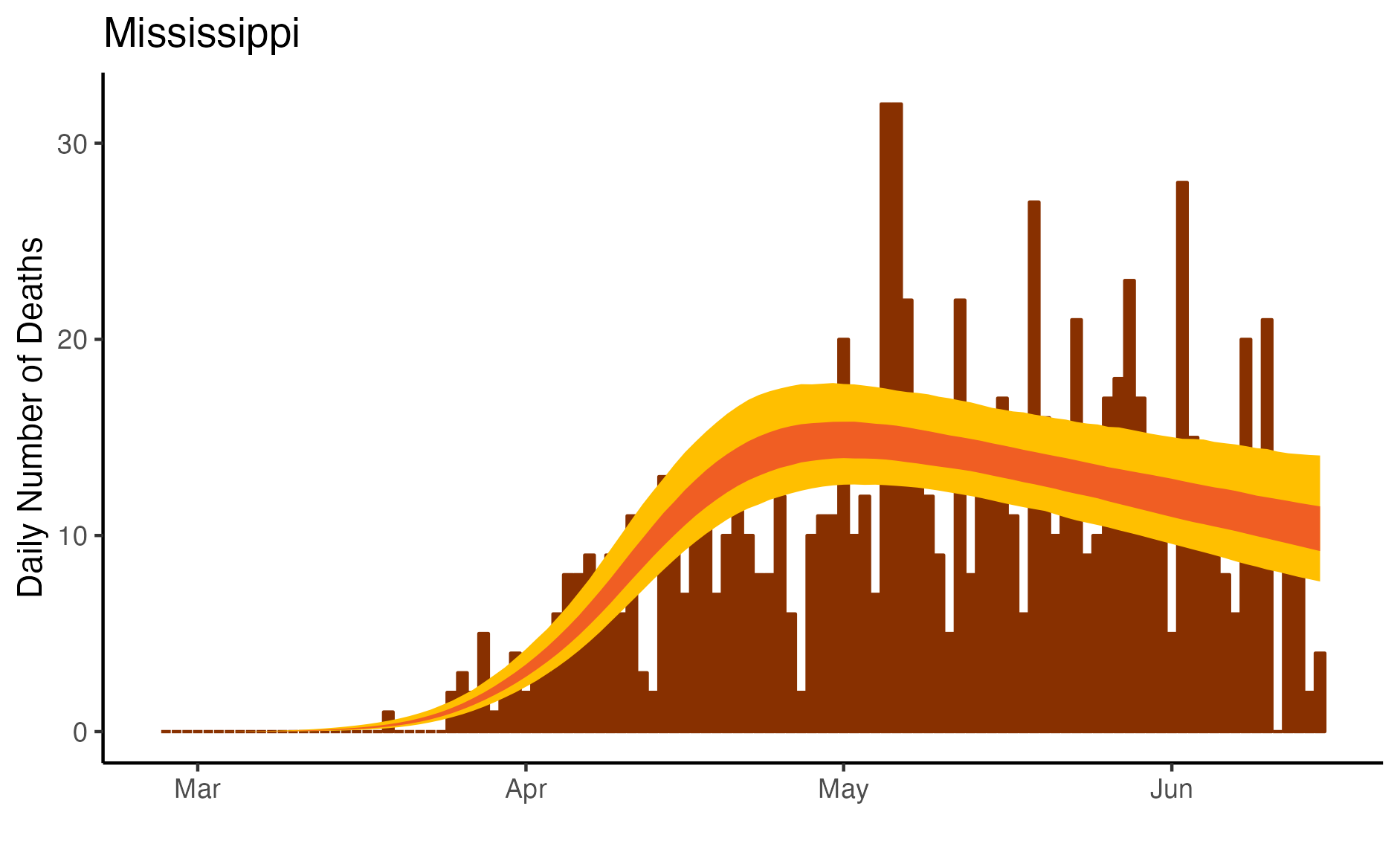

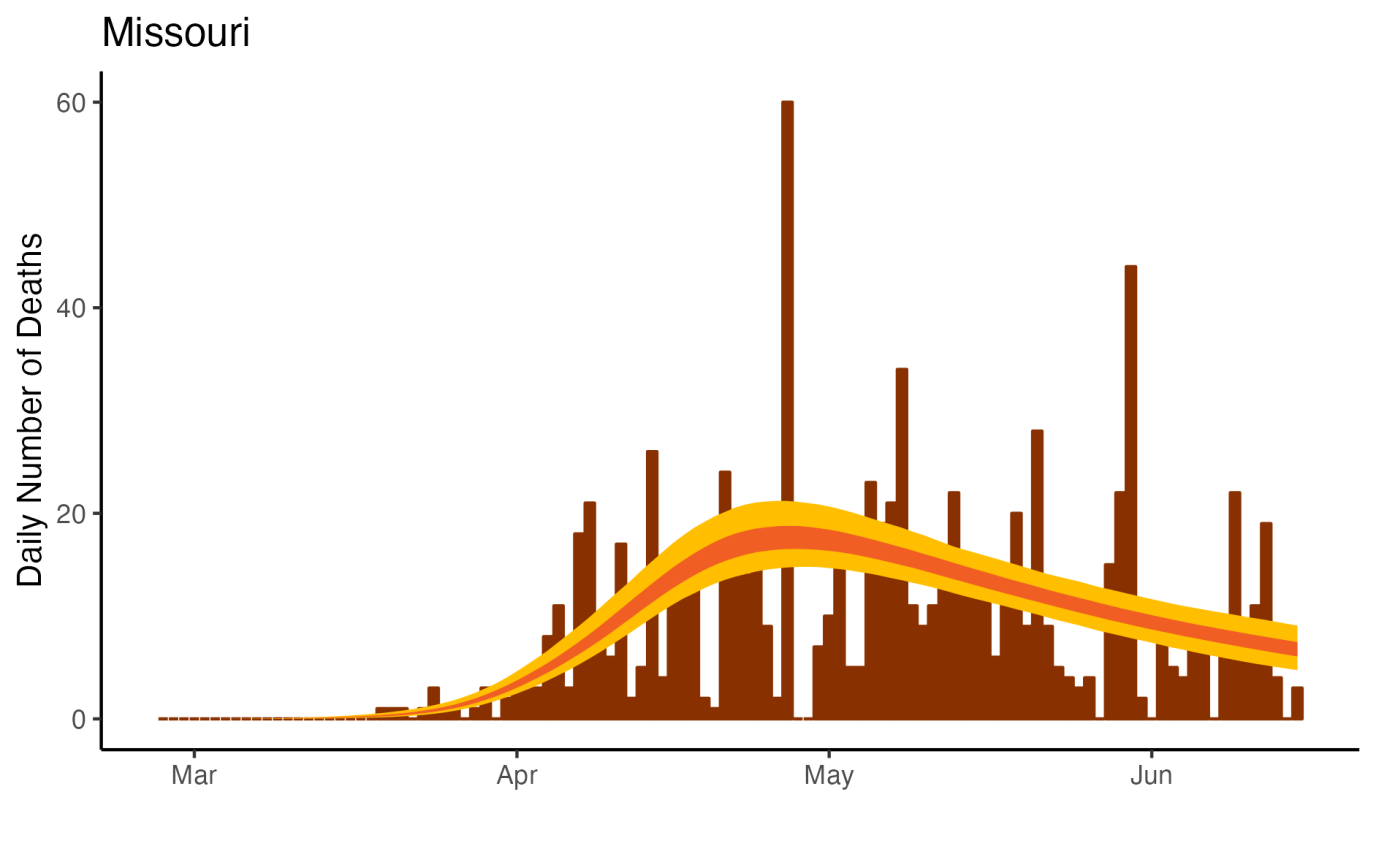

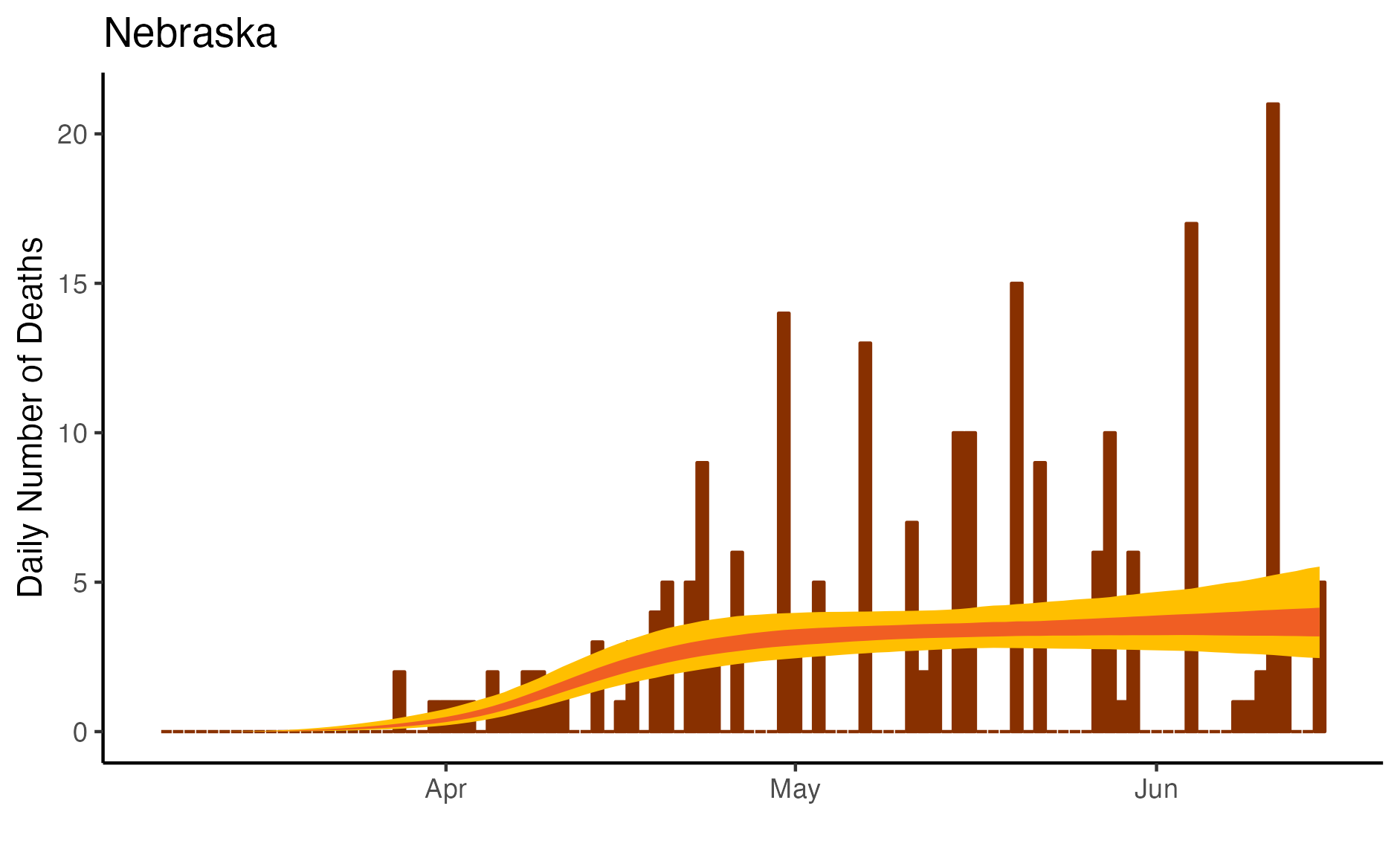

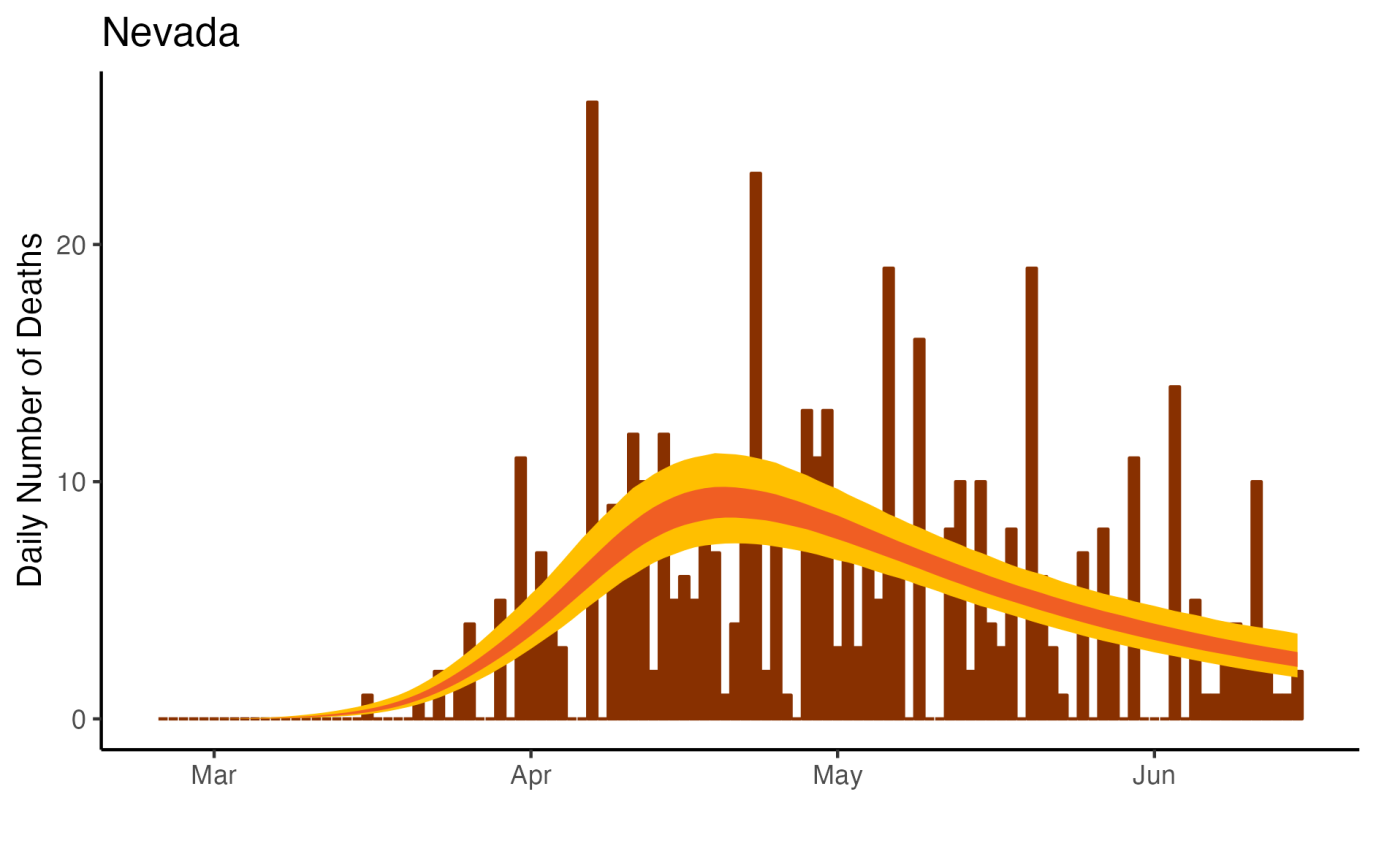

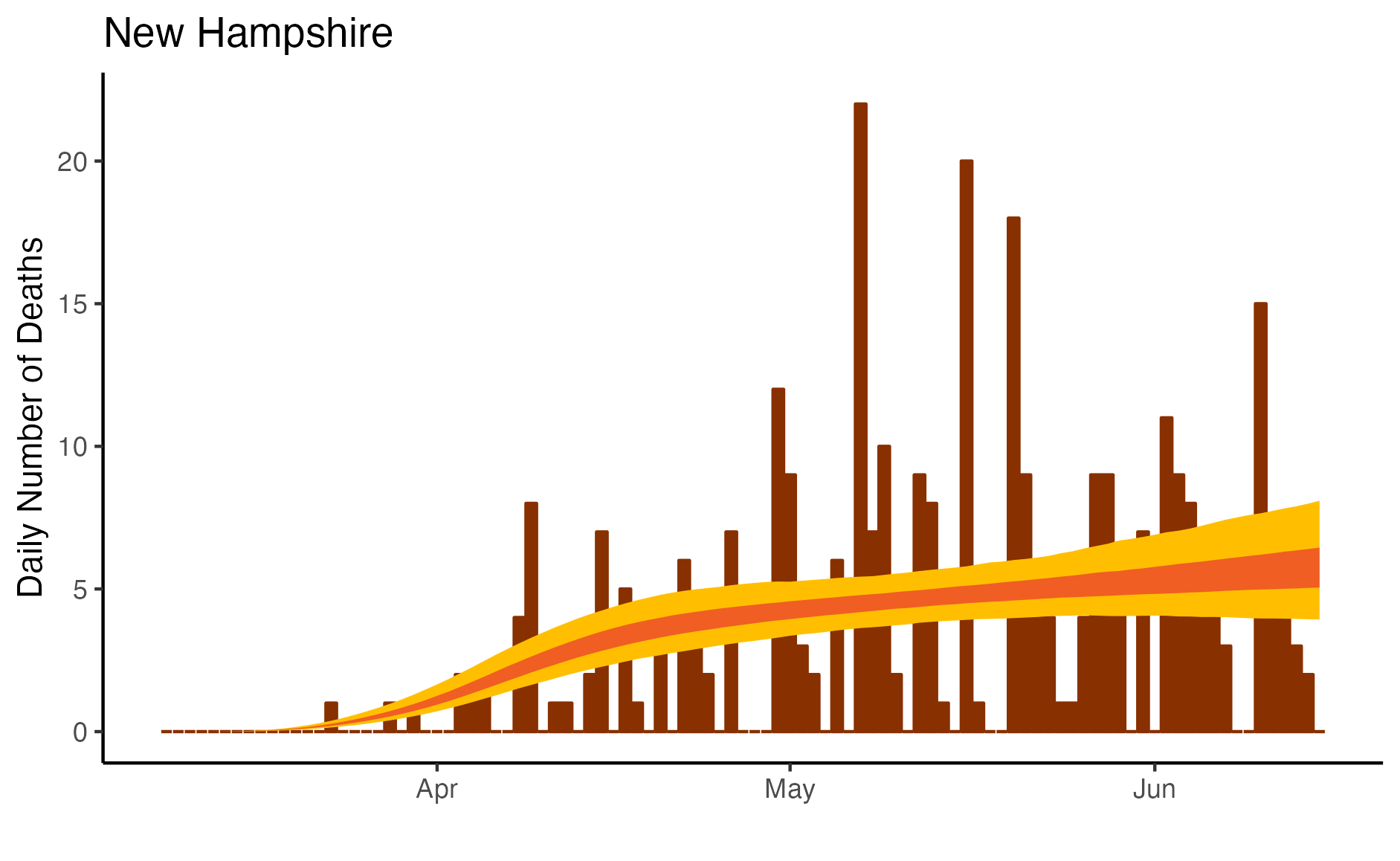

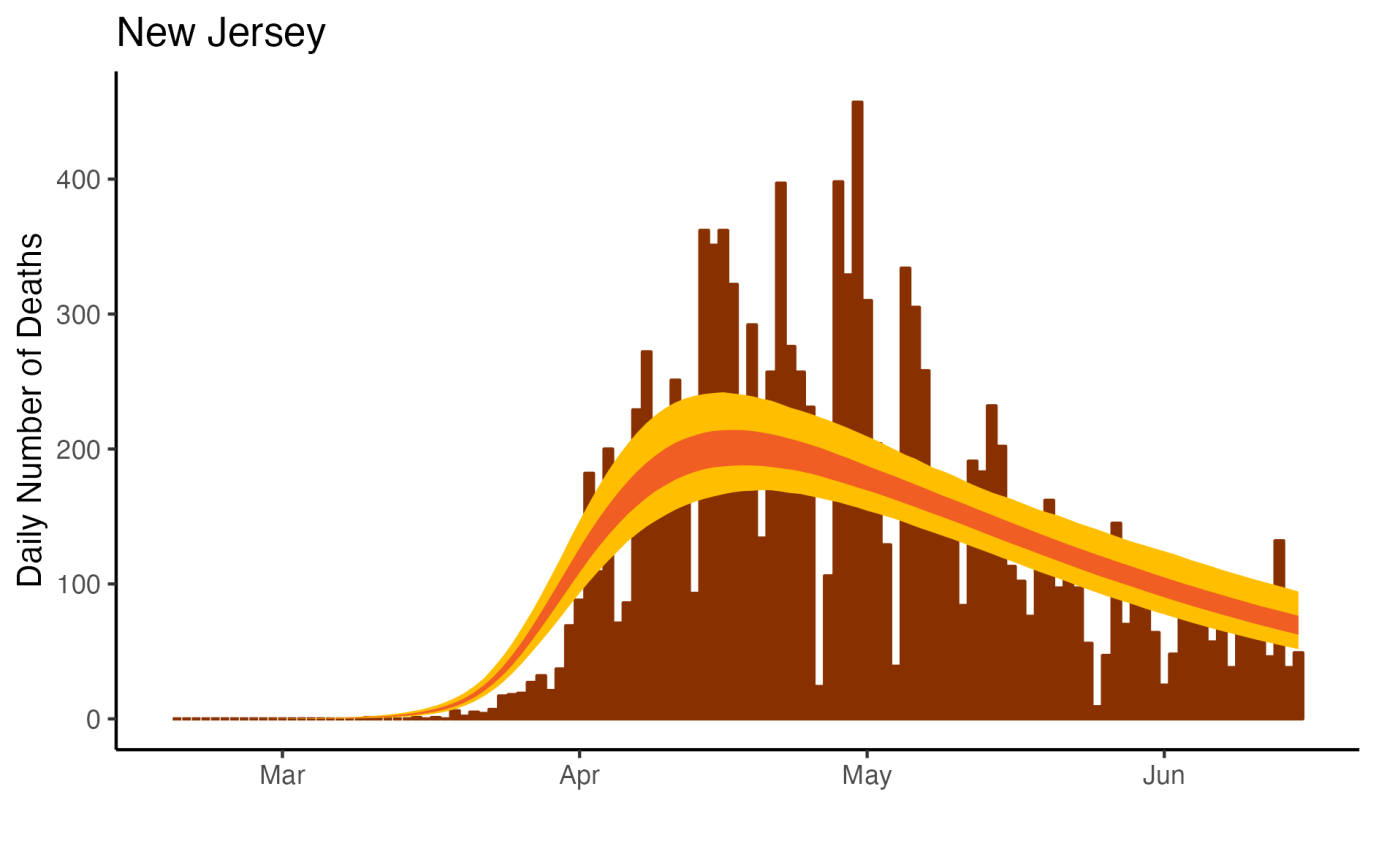

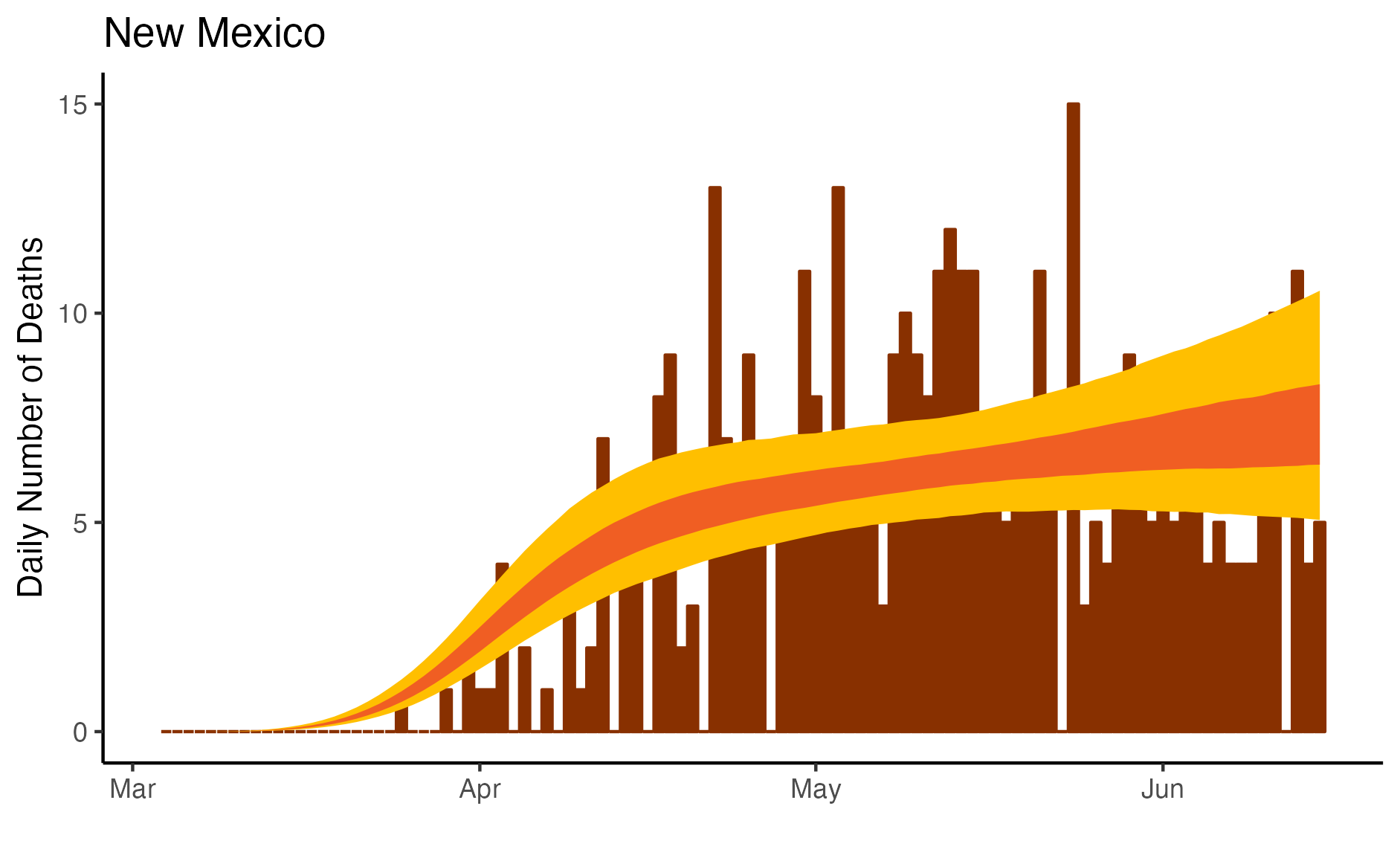

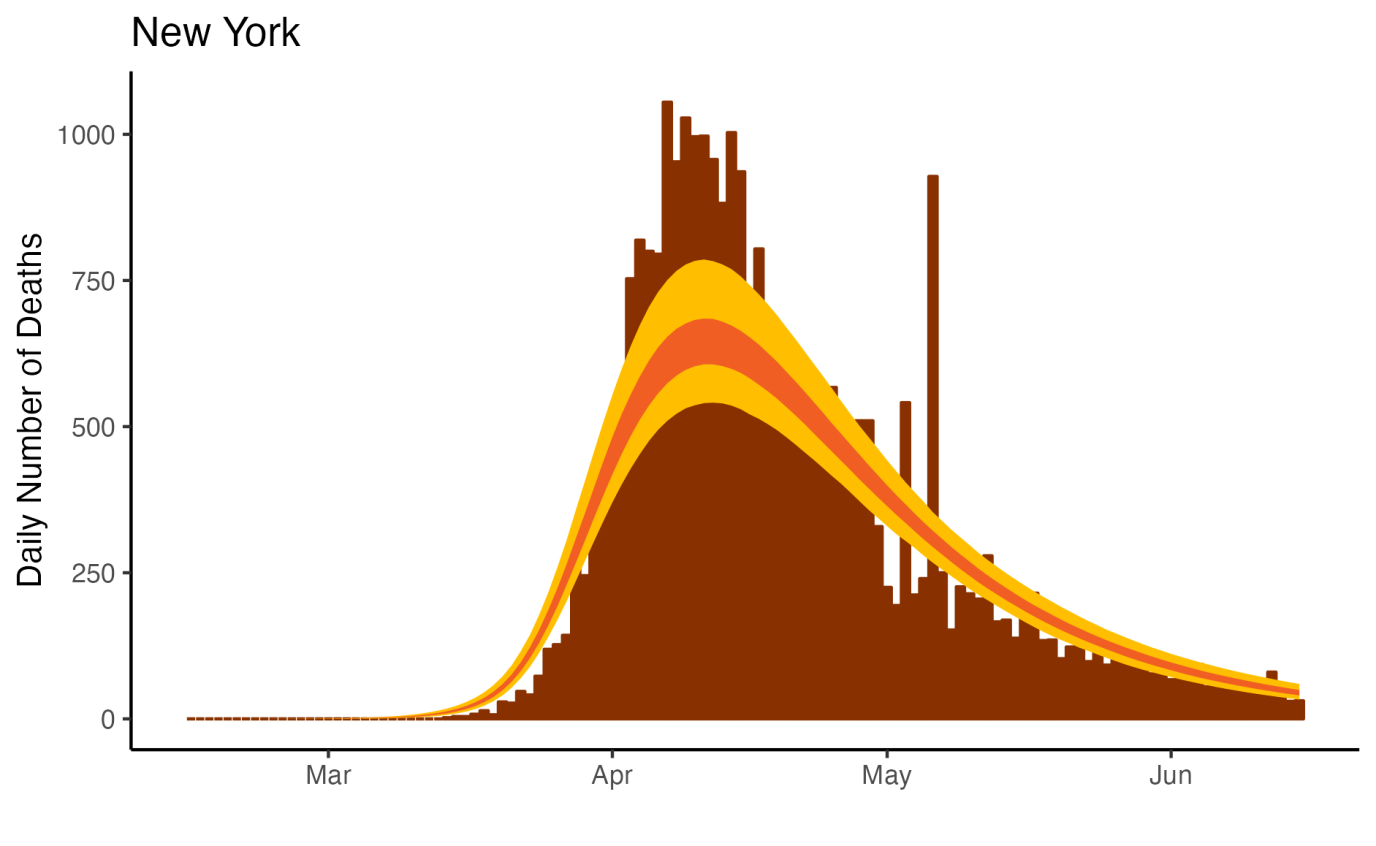

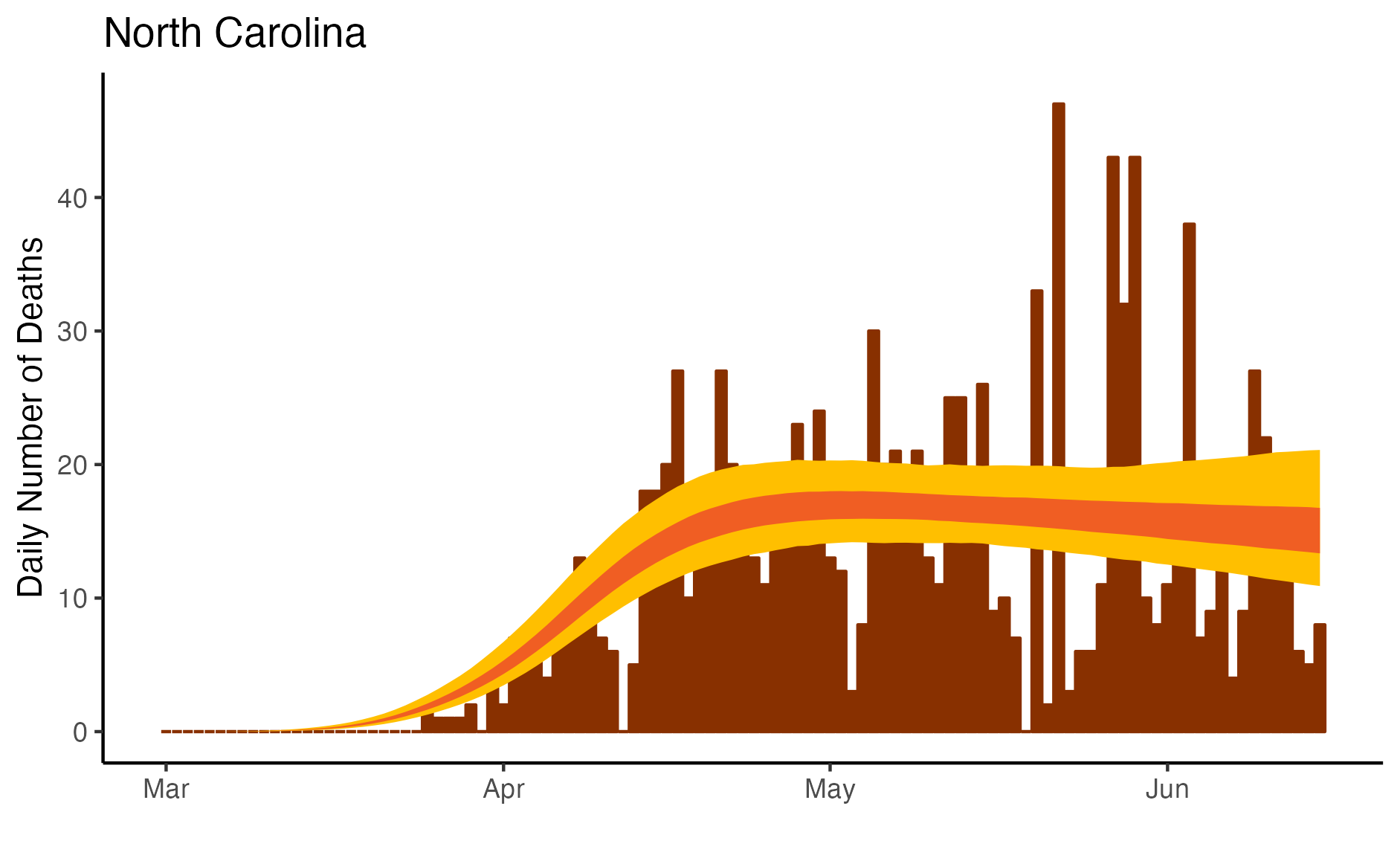

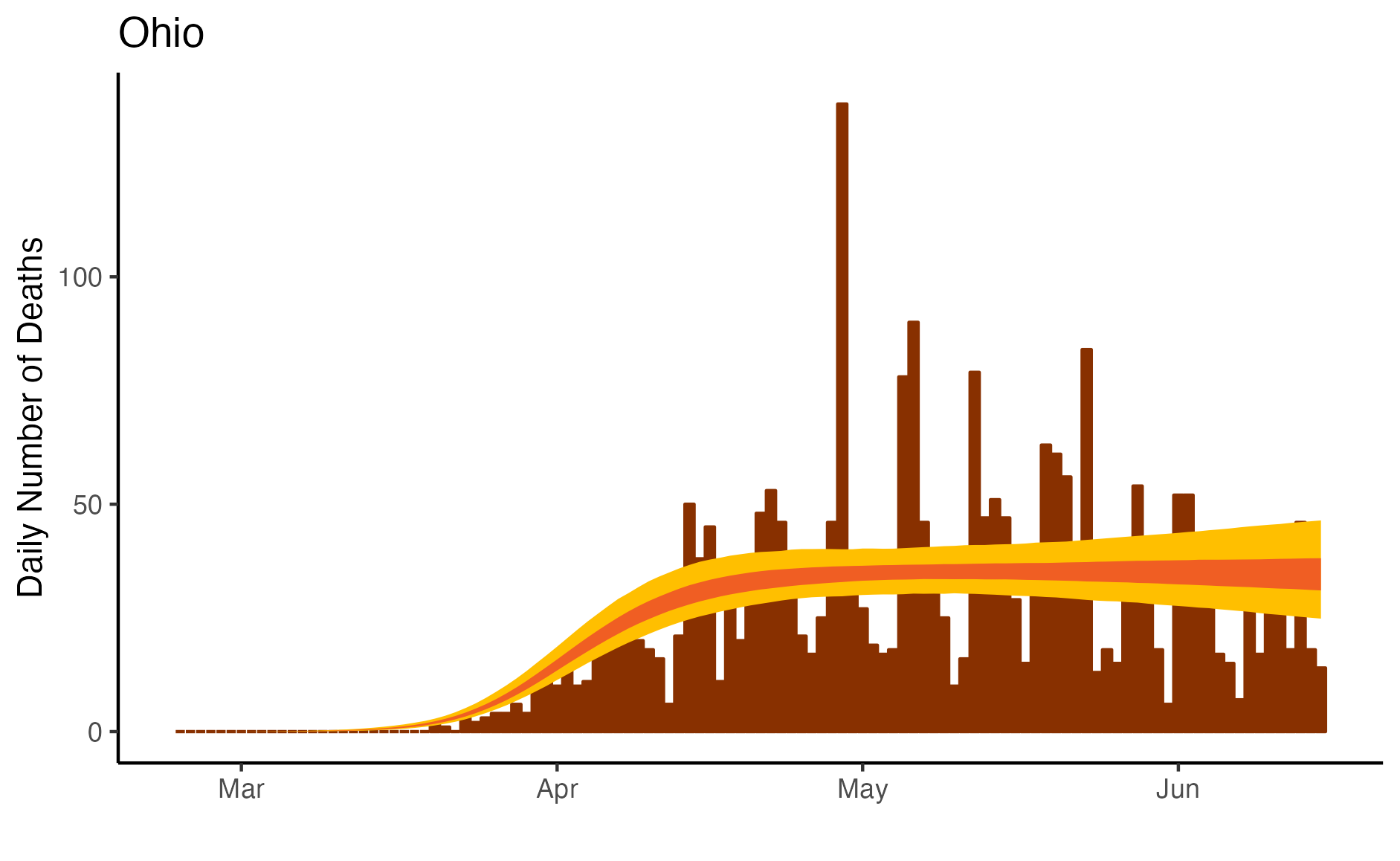

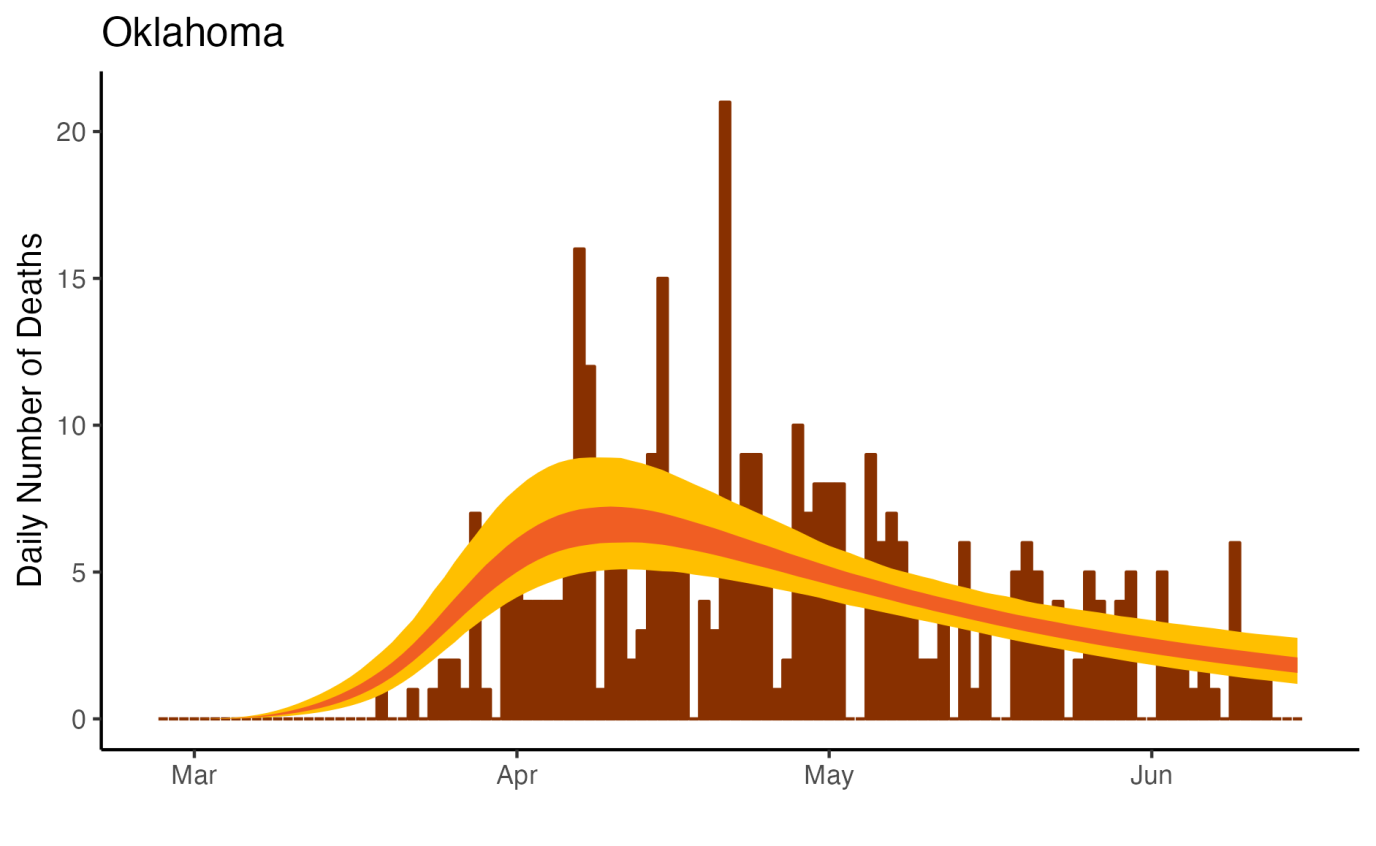

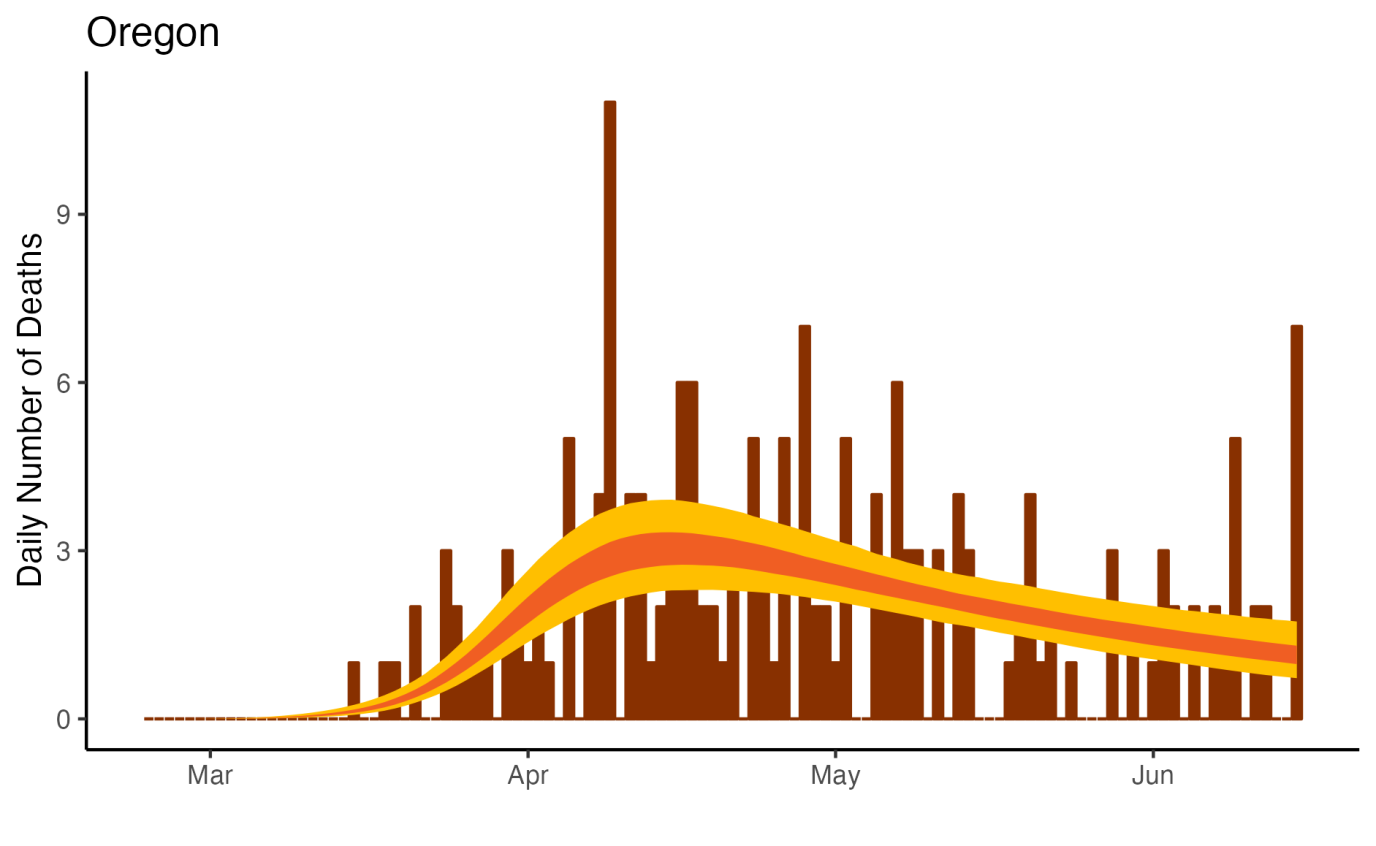

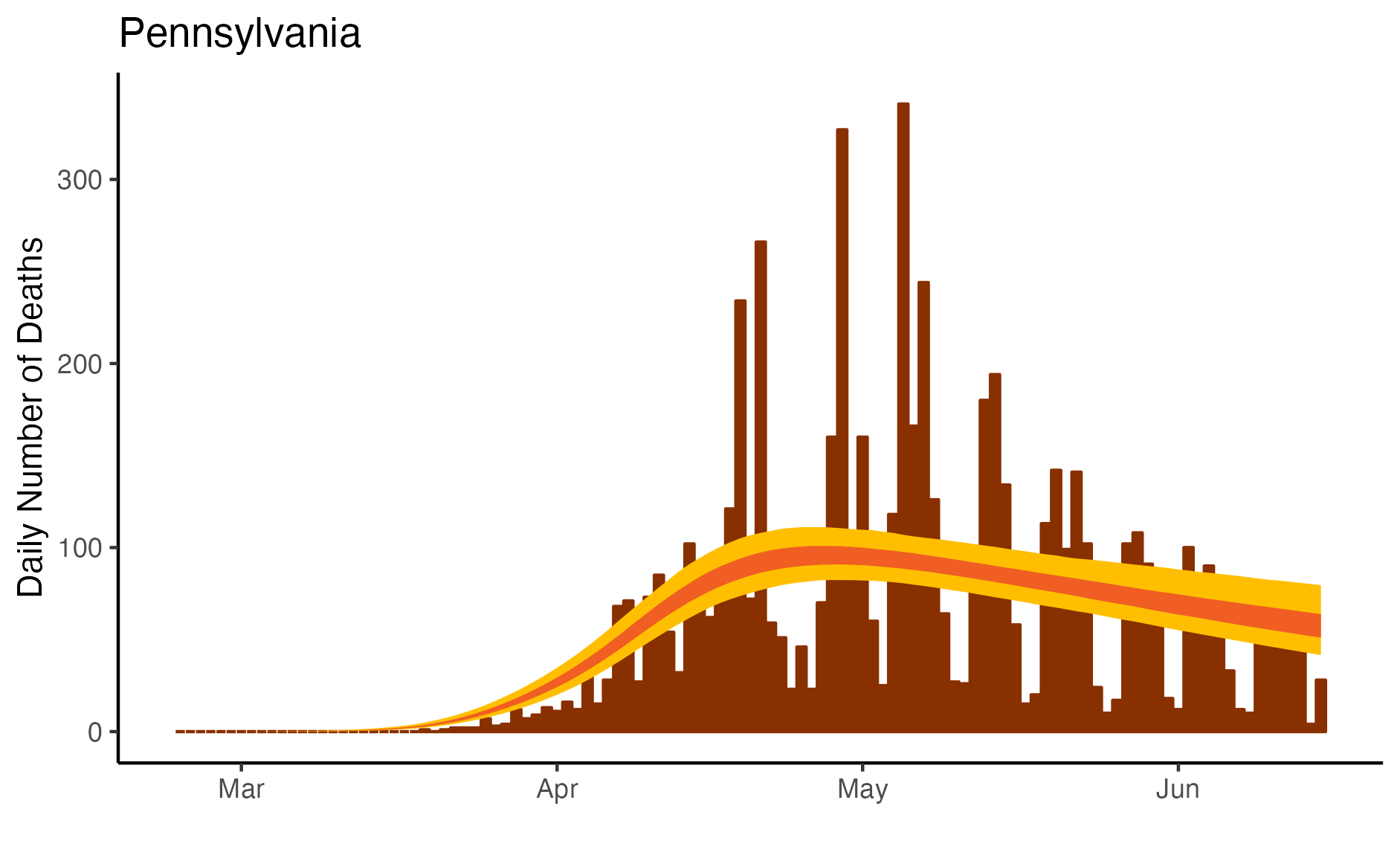

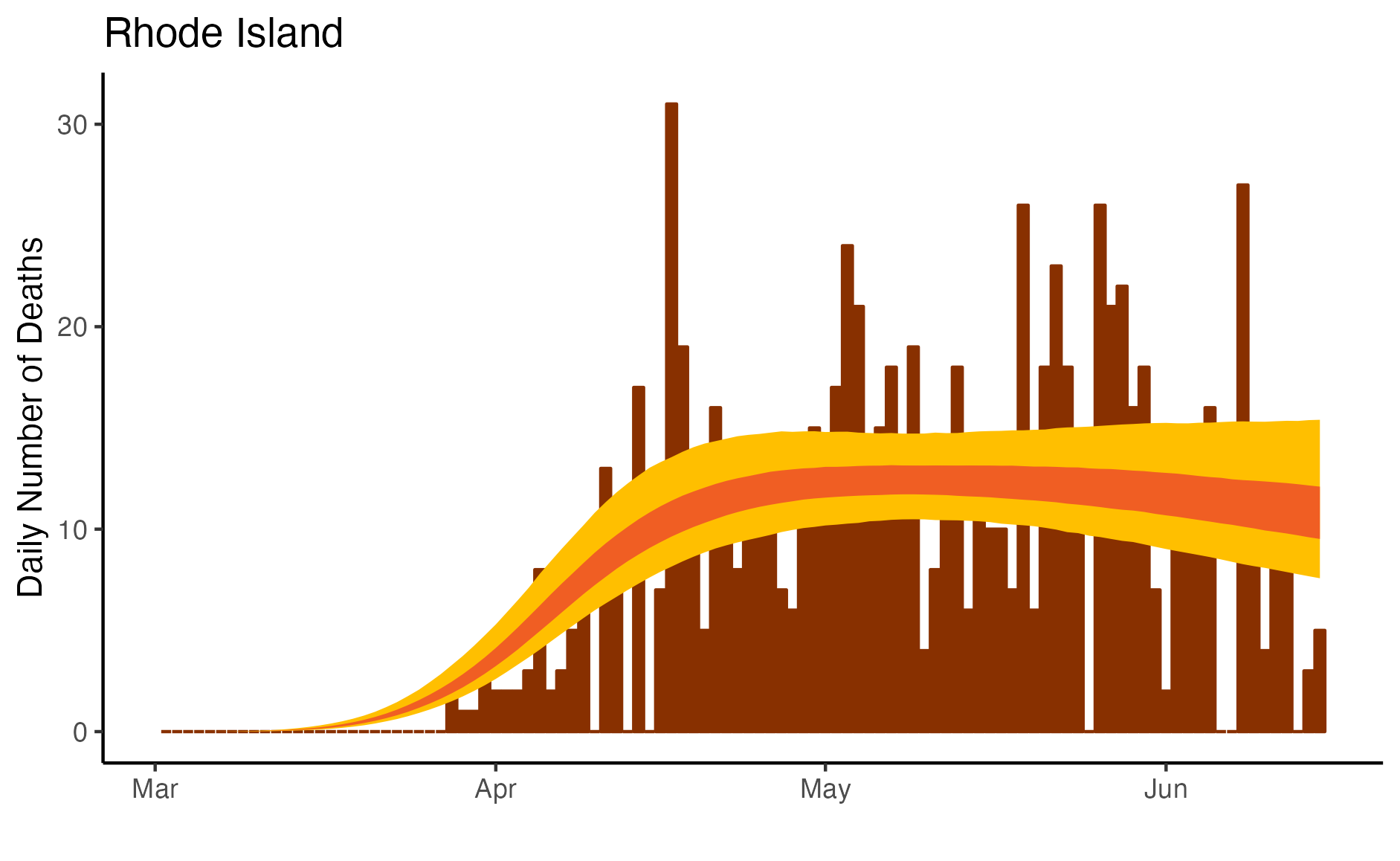

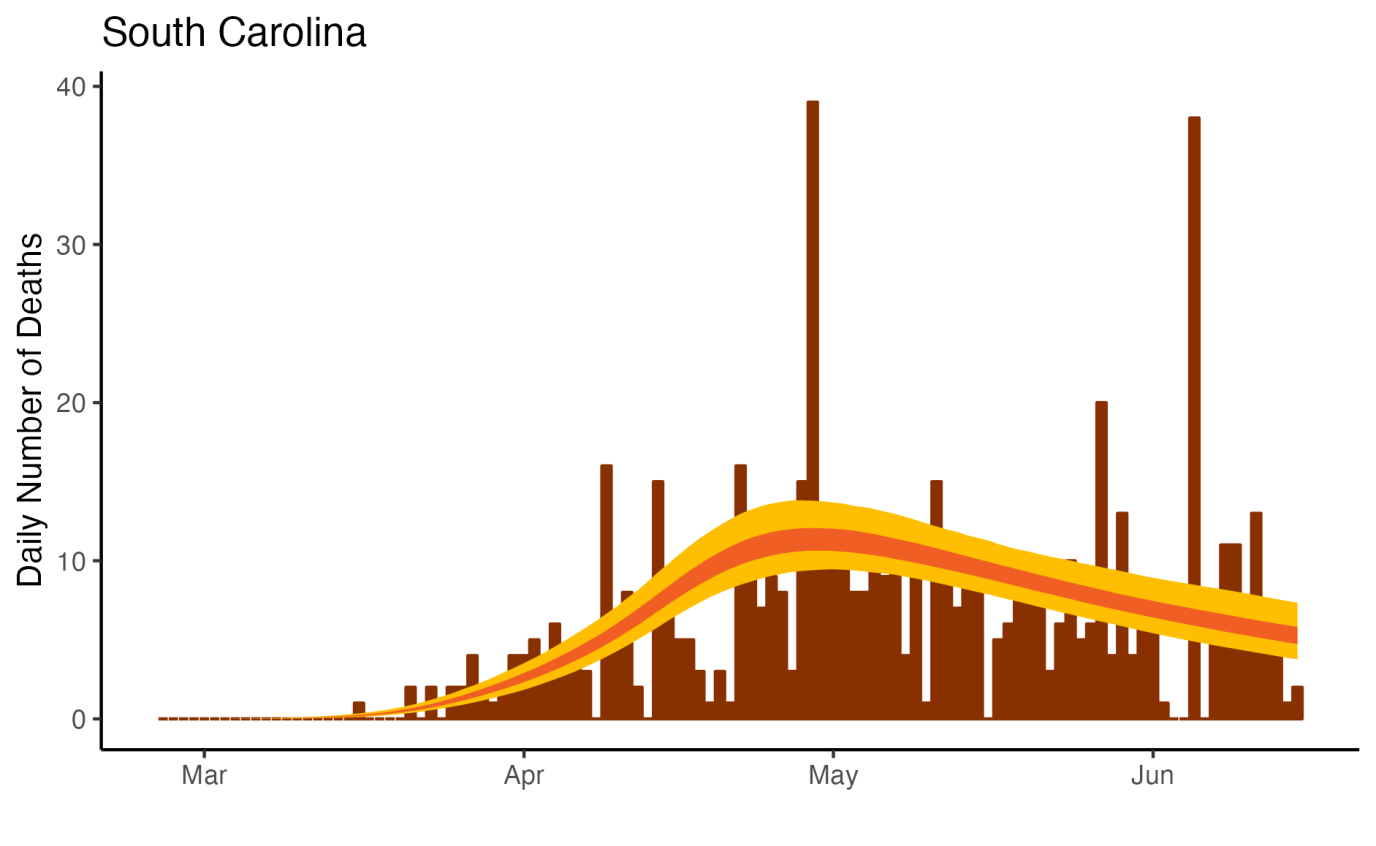

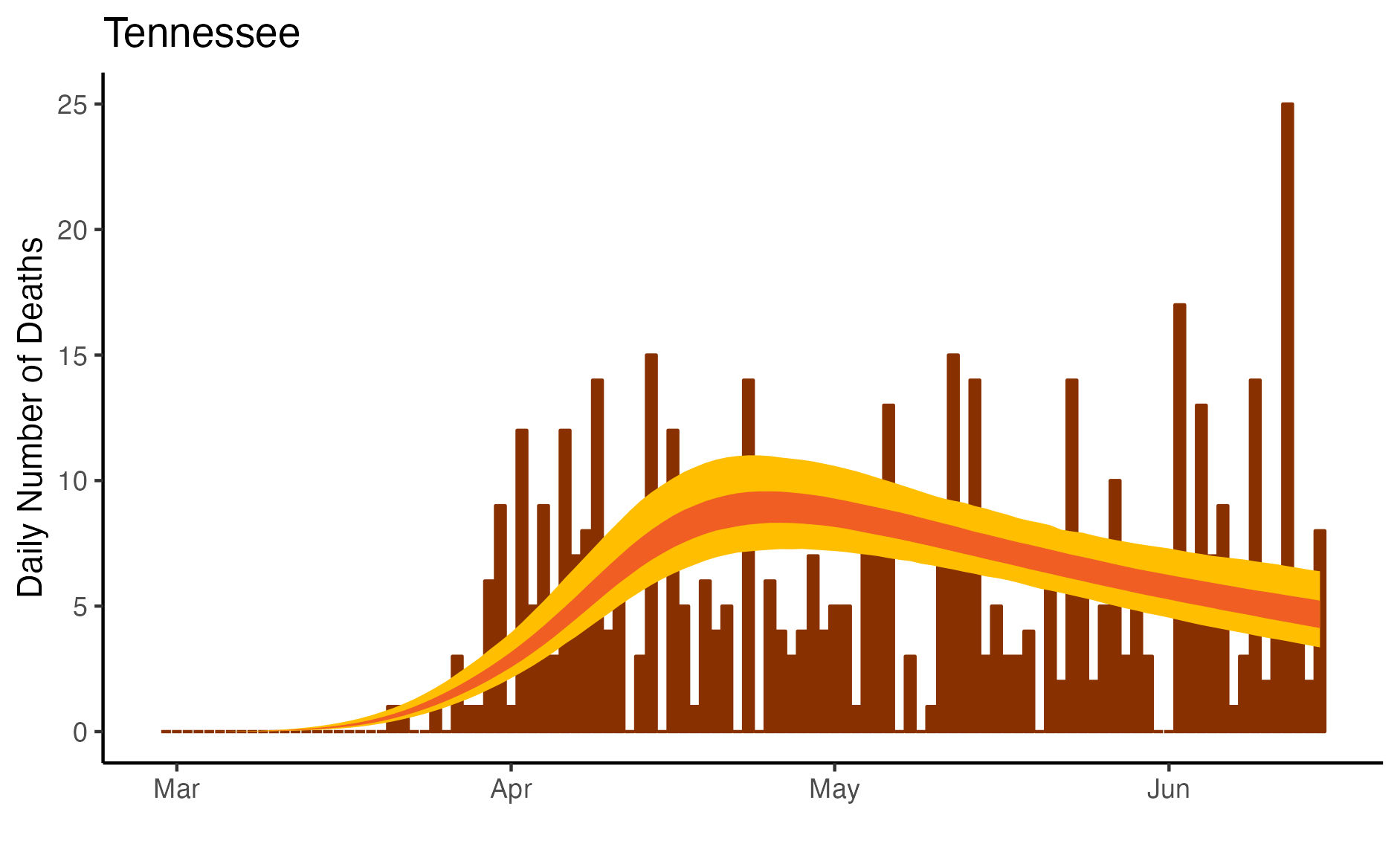

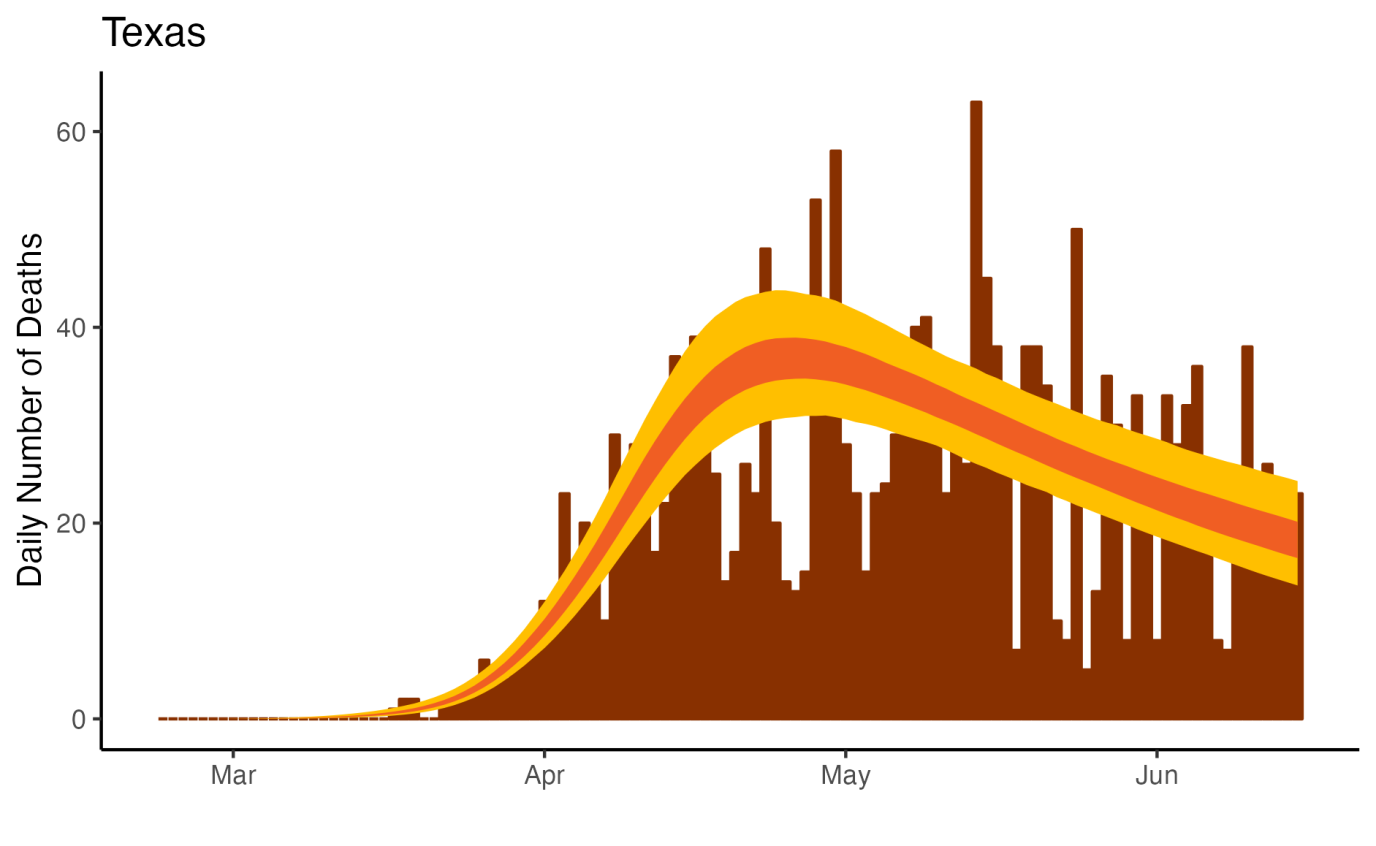

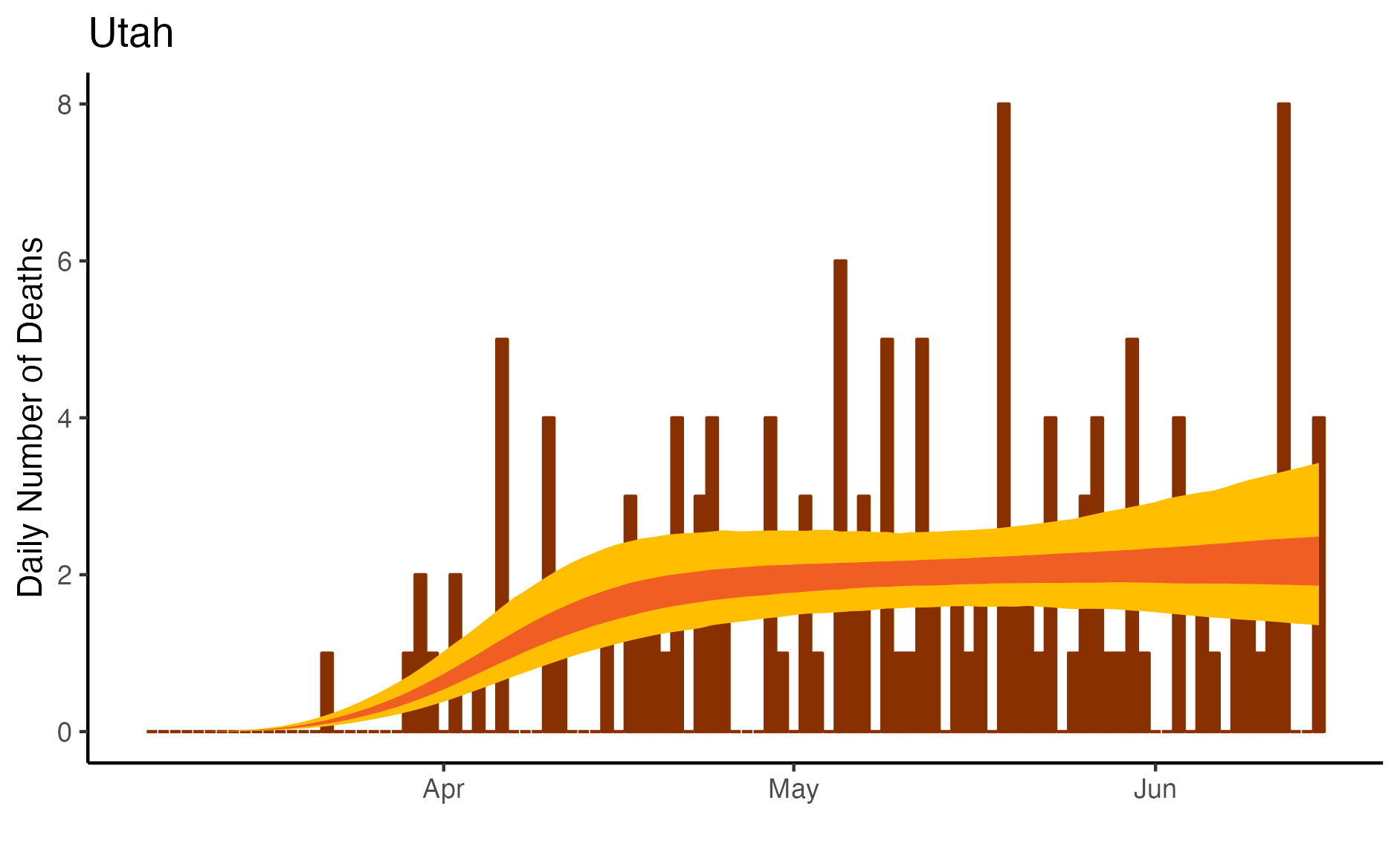

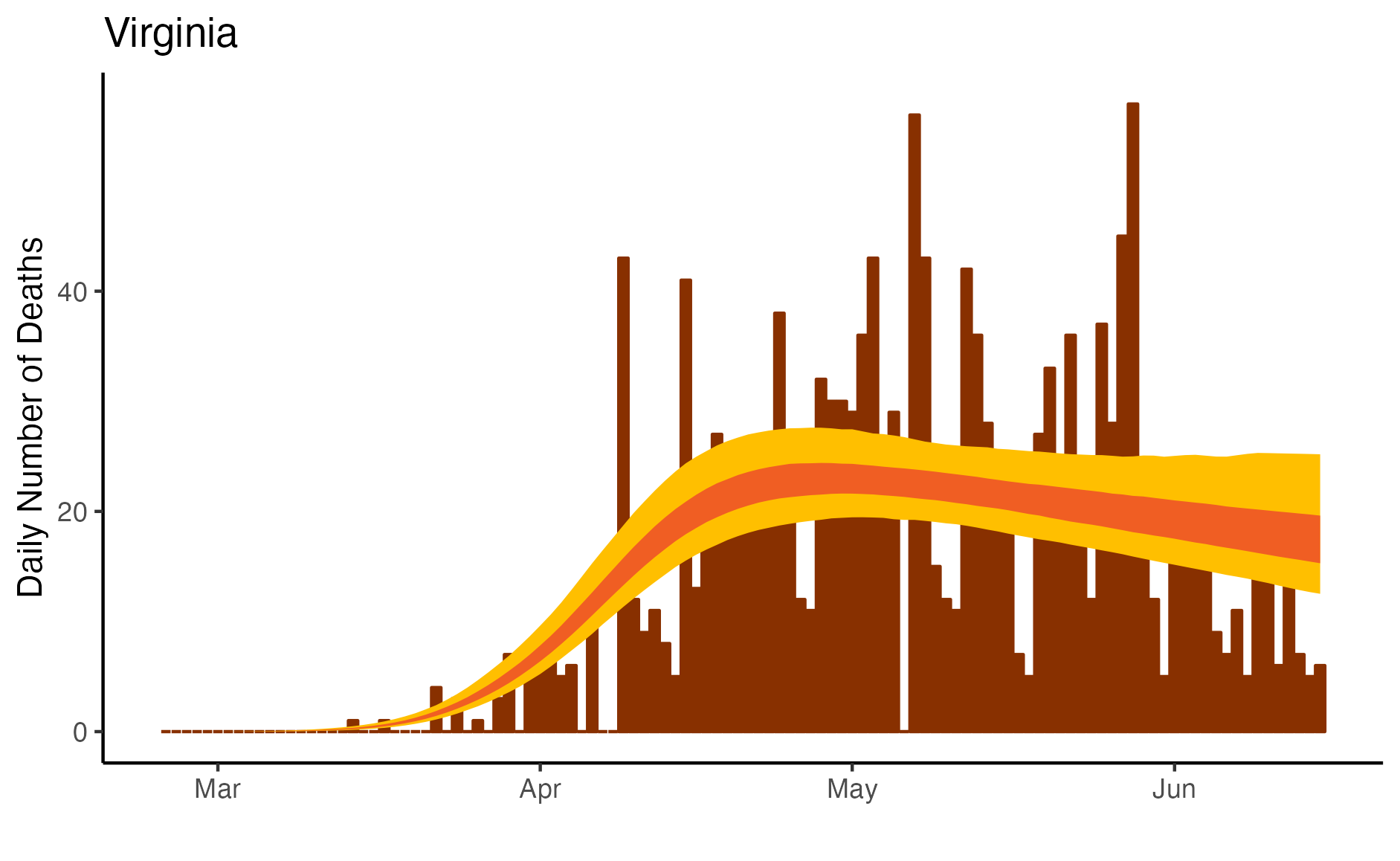

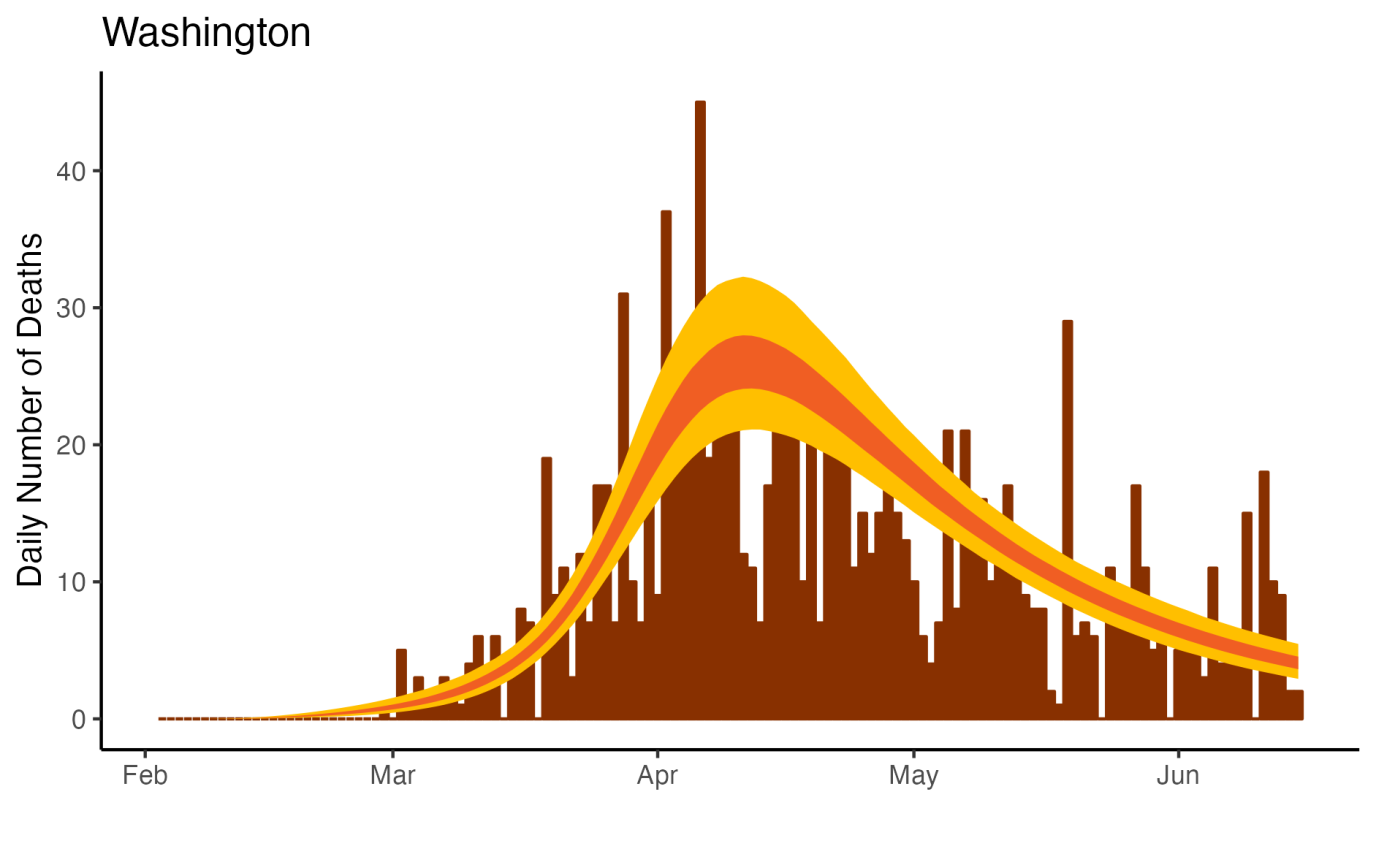

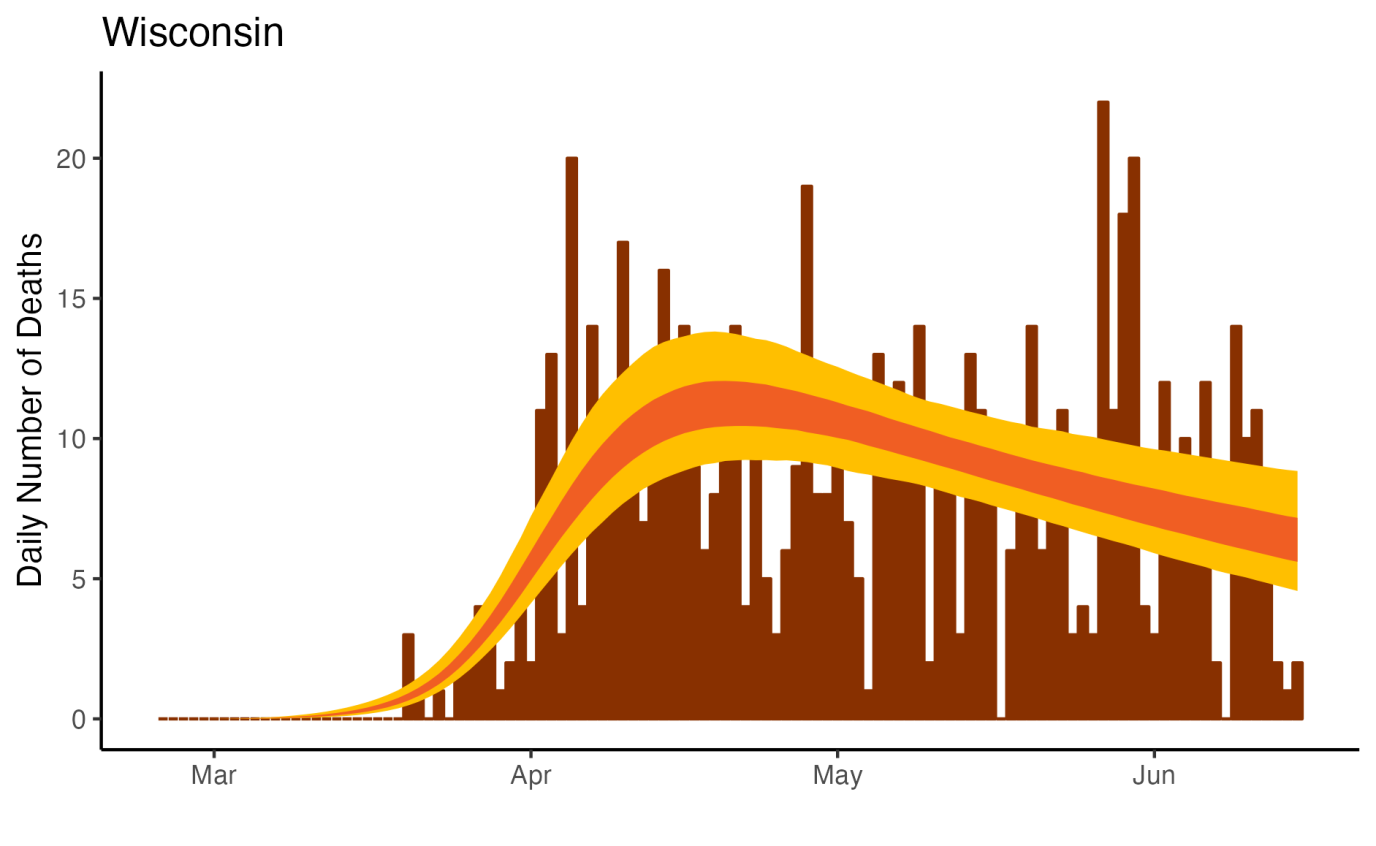


**Figure List 3:** Advanced Prototypical Bayesian Hierarchical Model Expected Daily Number of Reported Infection Estimation Over the Study Period by states (**S**: School Closure, **R**: Restaurant Capacity Restriction, **G**: Gathering Restriction, **B**: Business Closure, **H**: Stay-at-Home Order, and **M**: Mask Mandate)
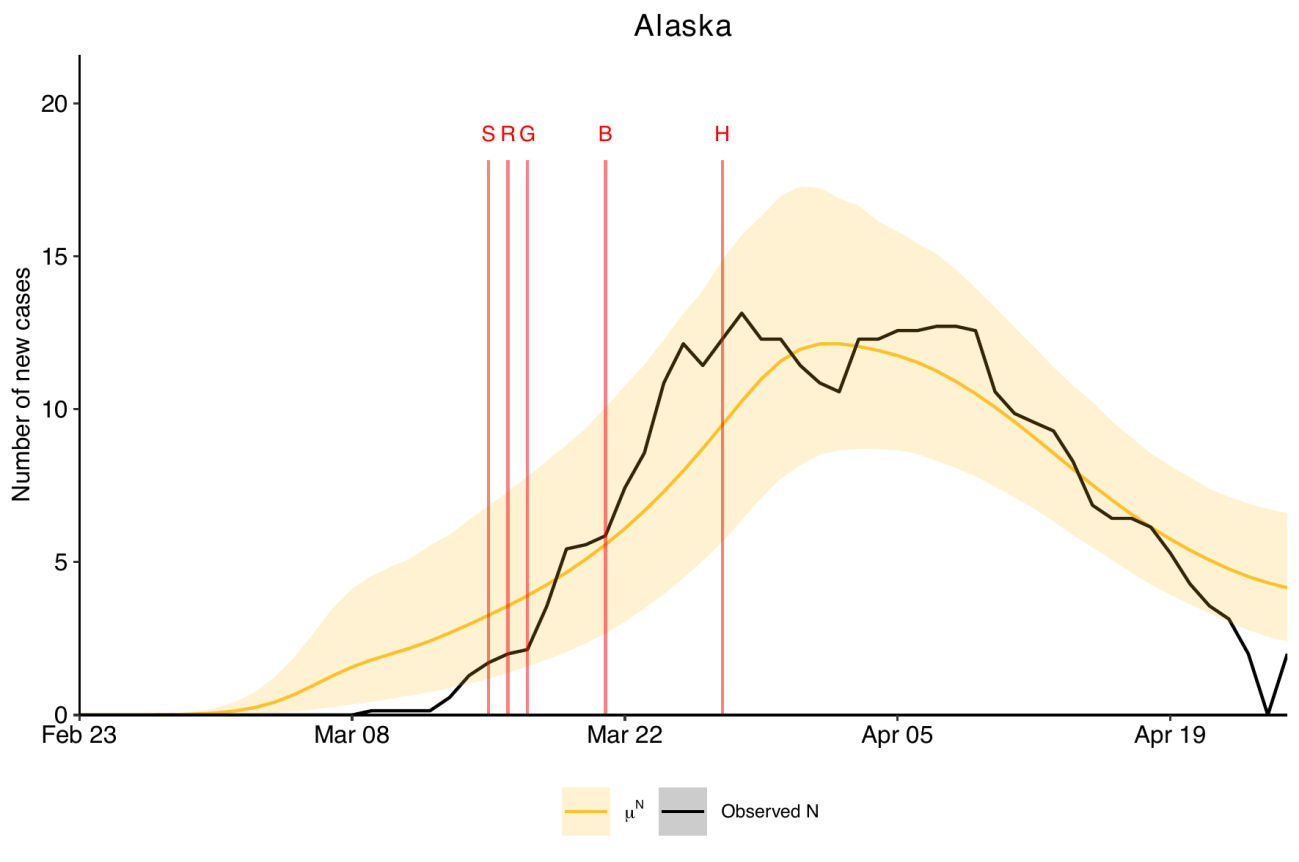

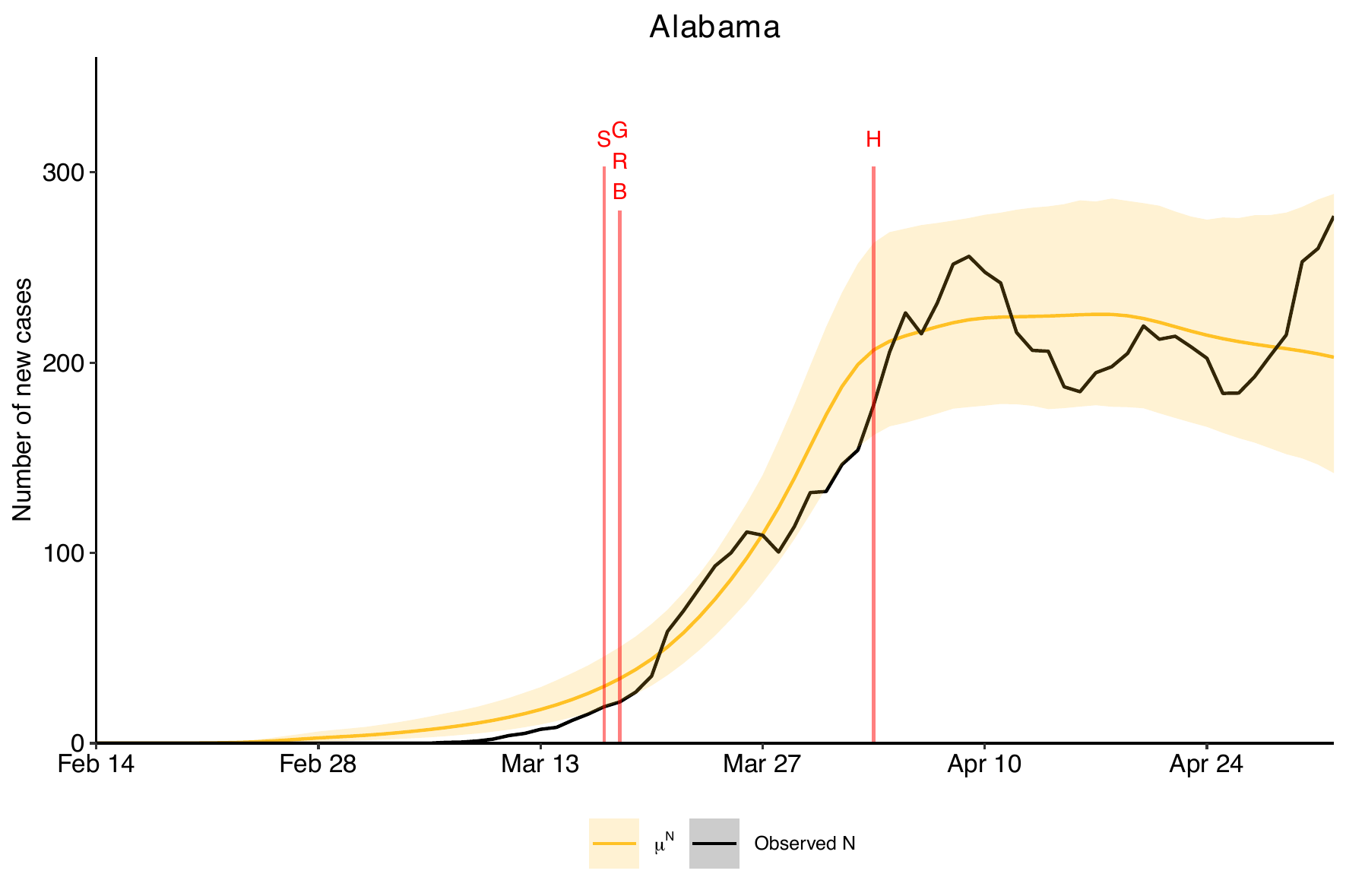

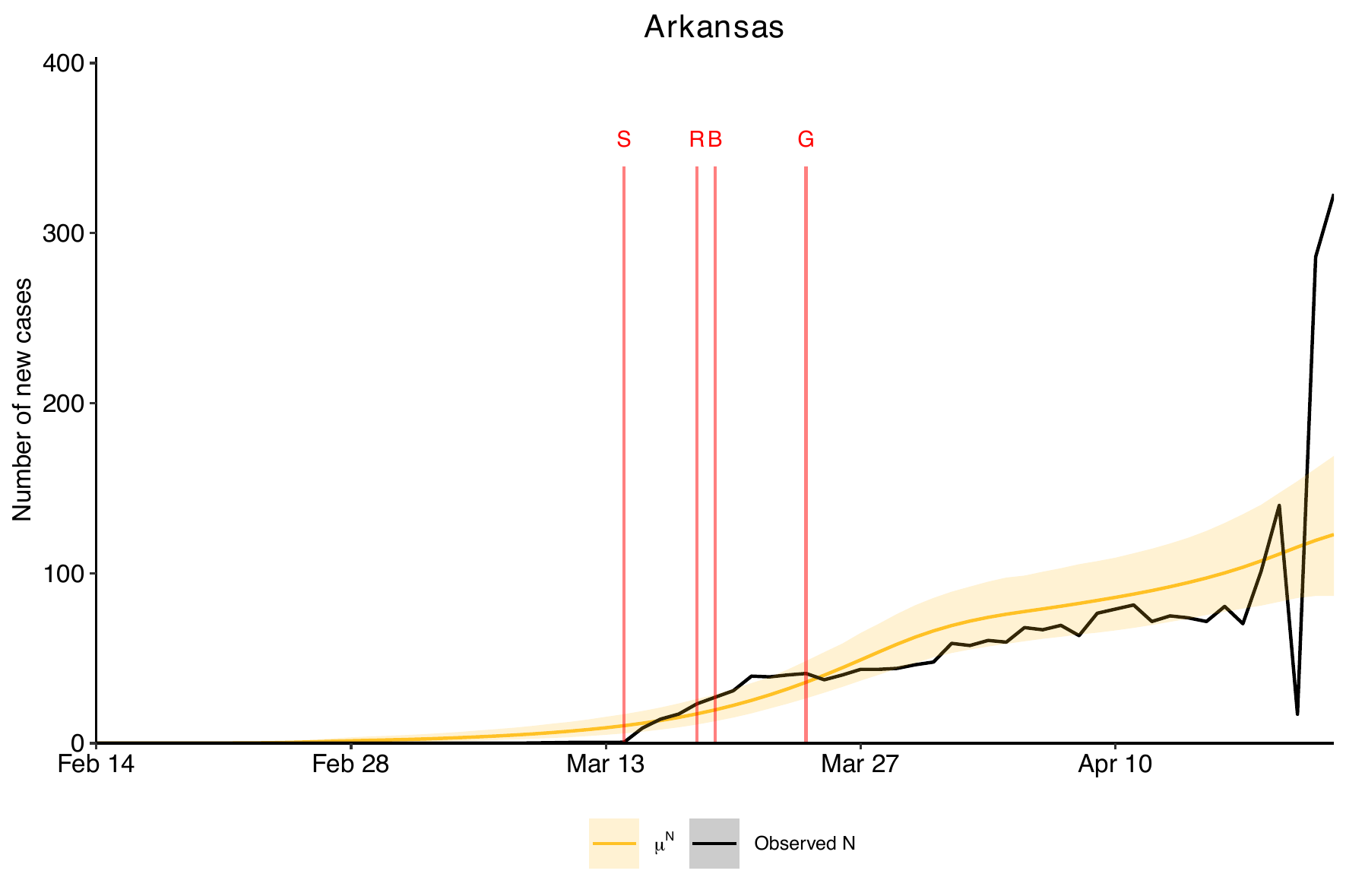

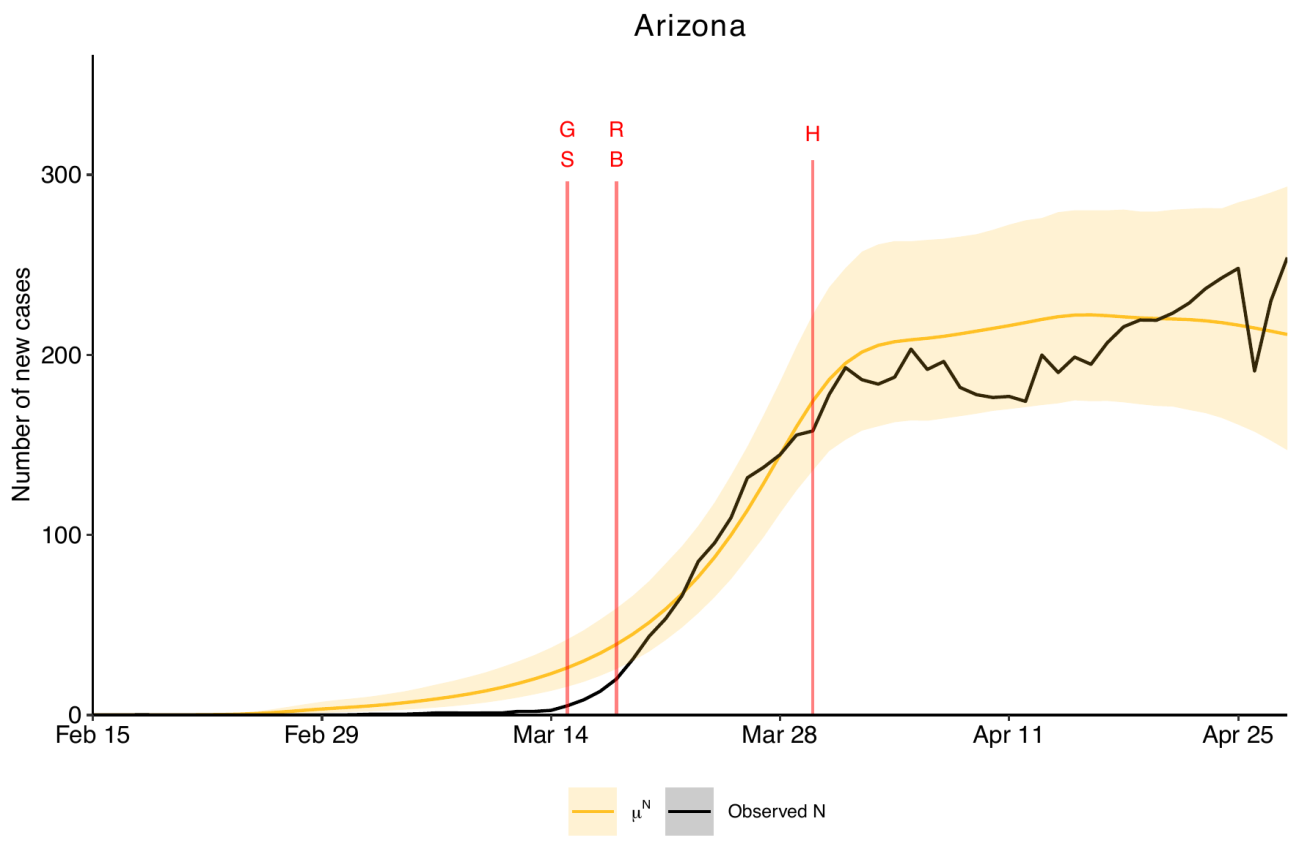

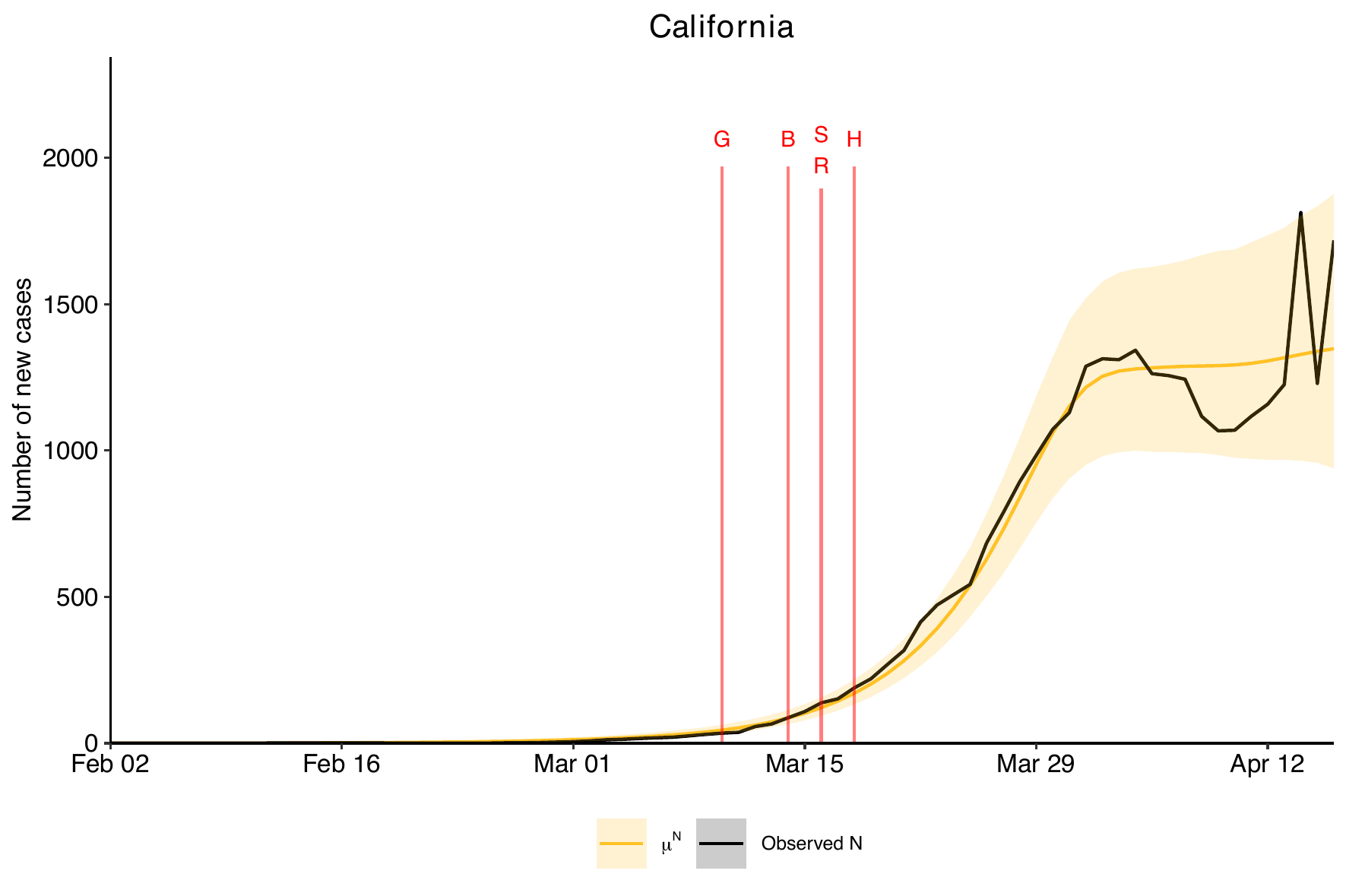

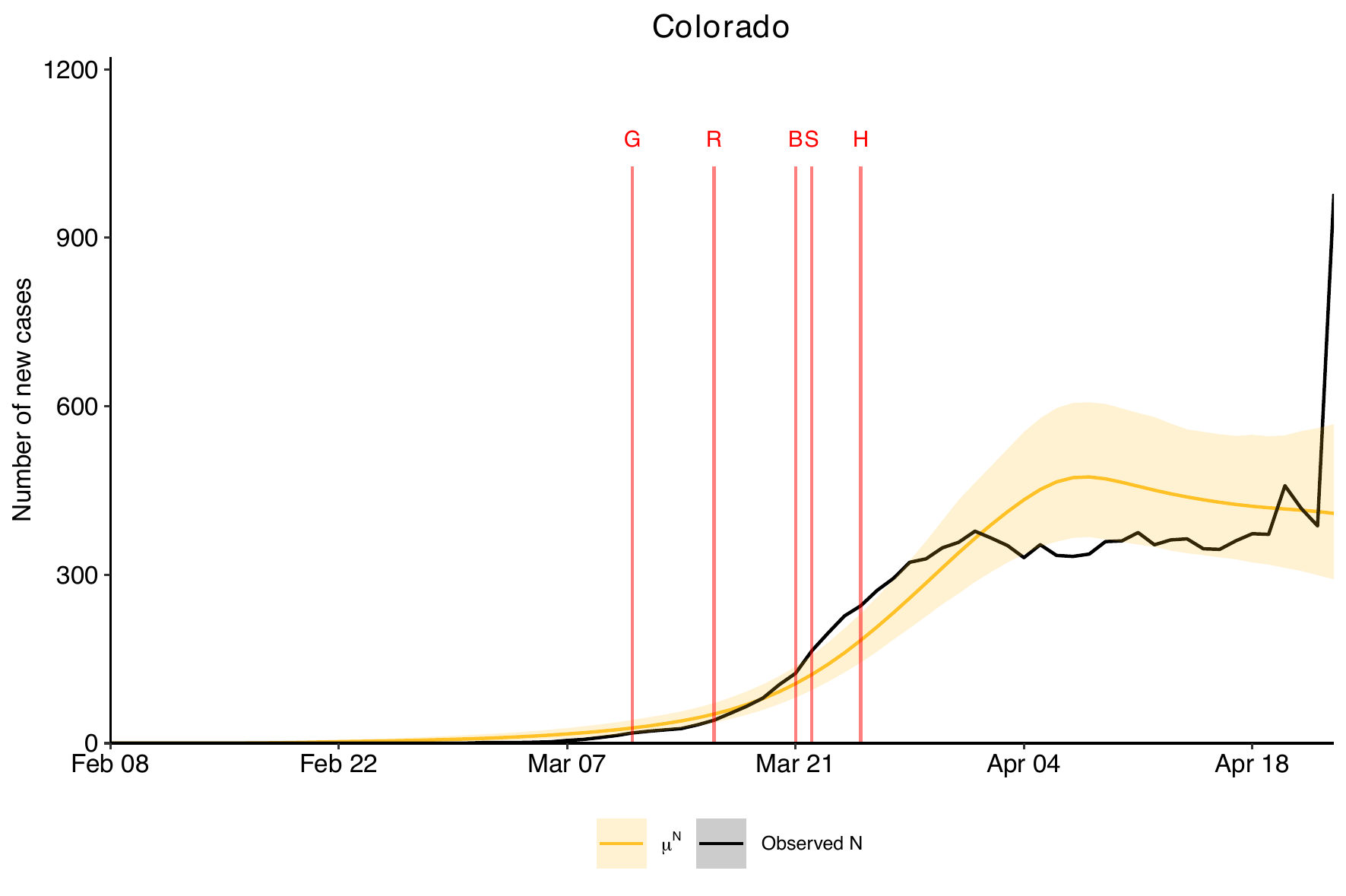

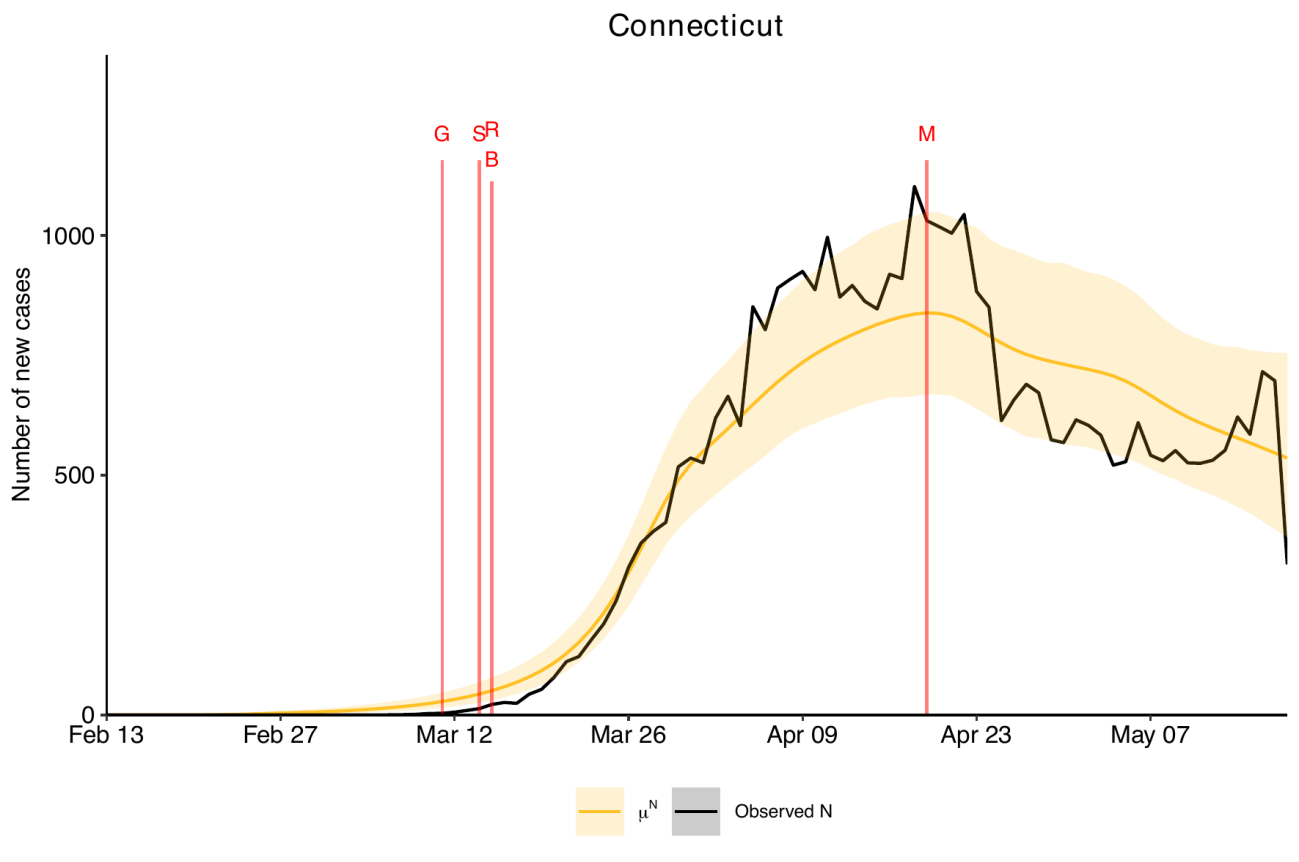

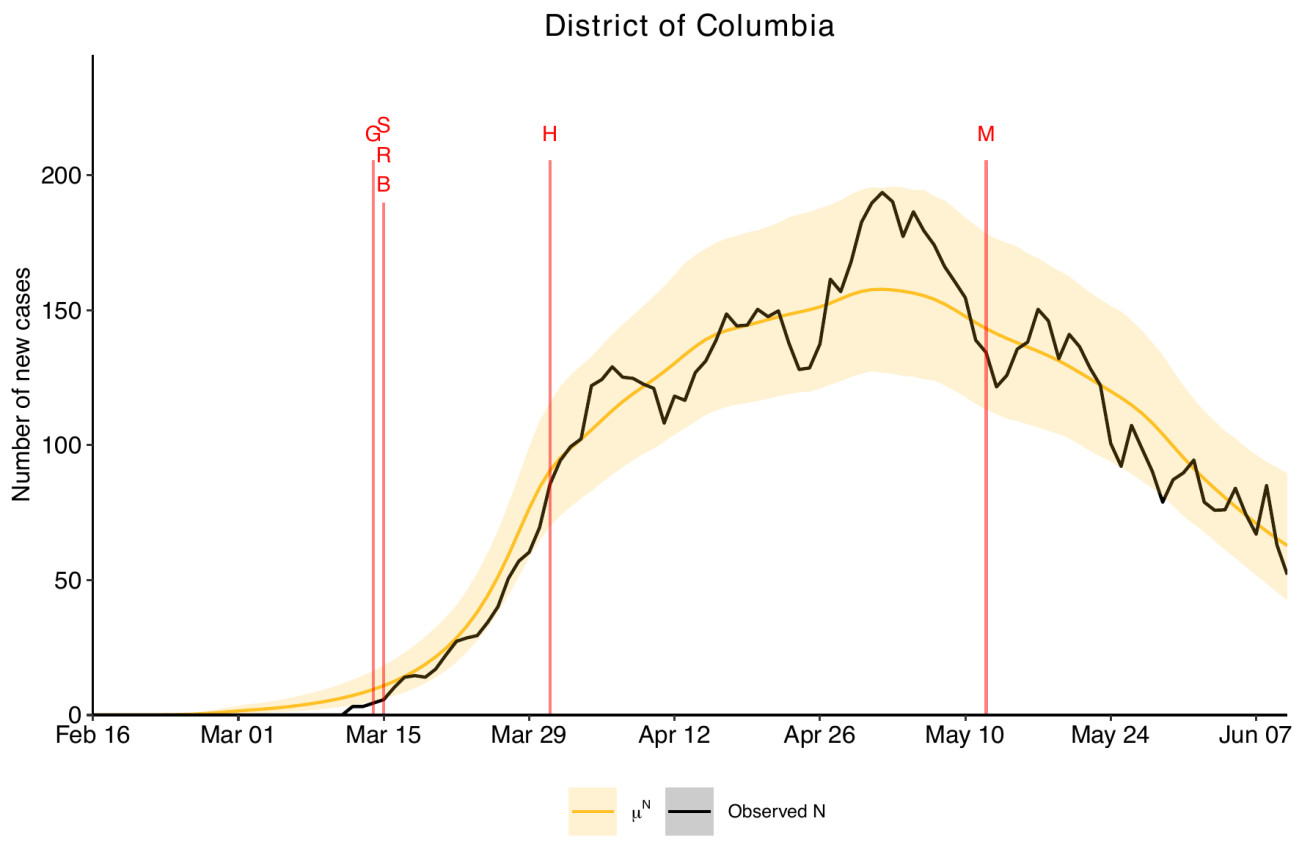

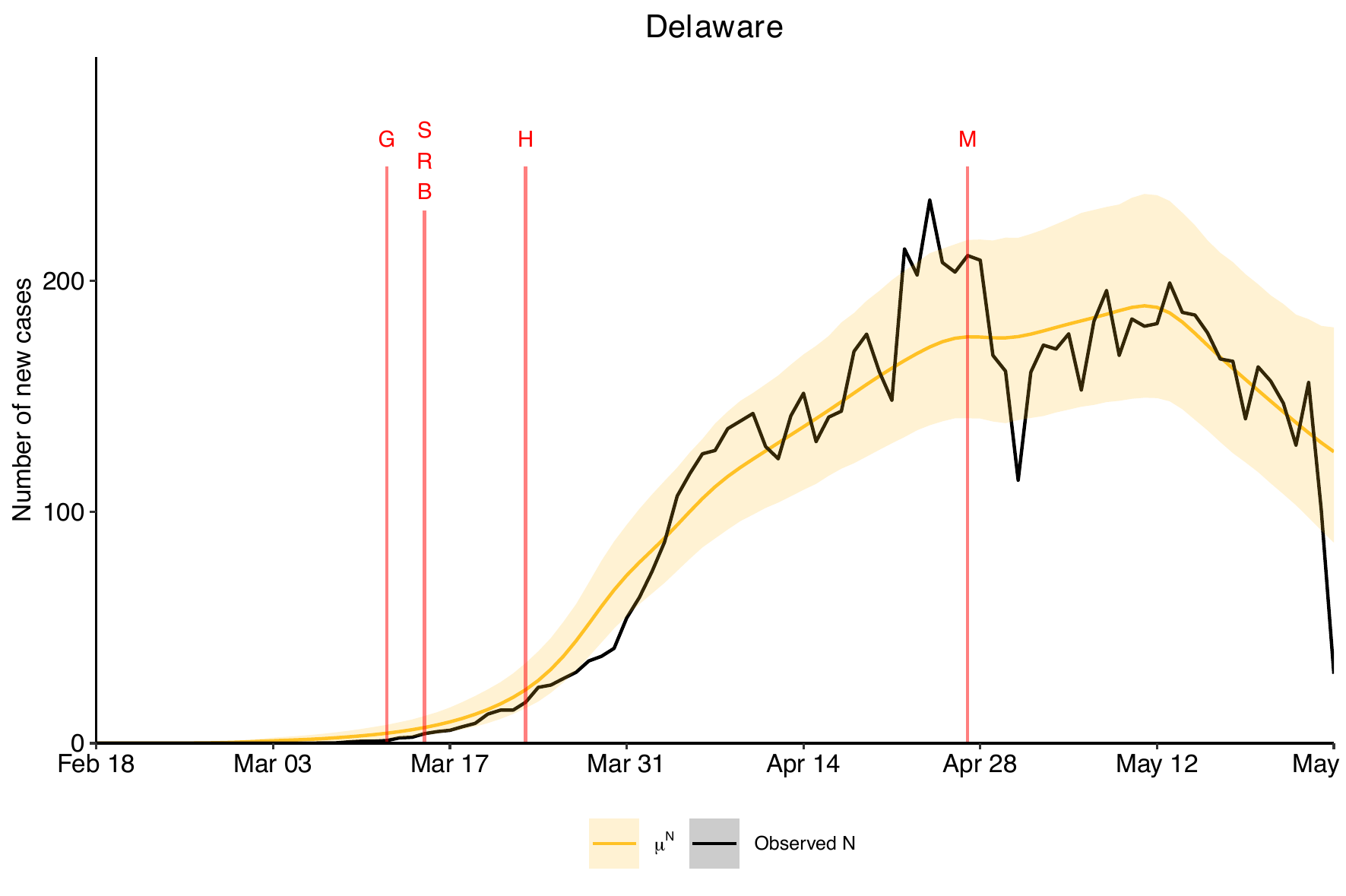

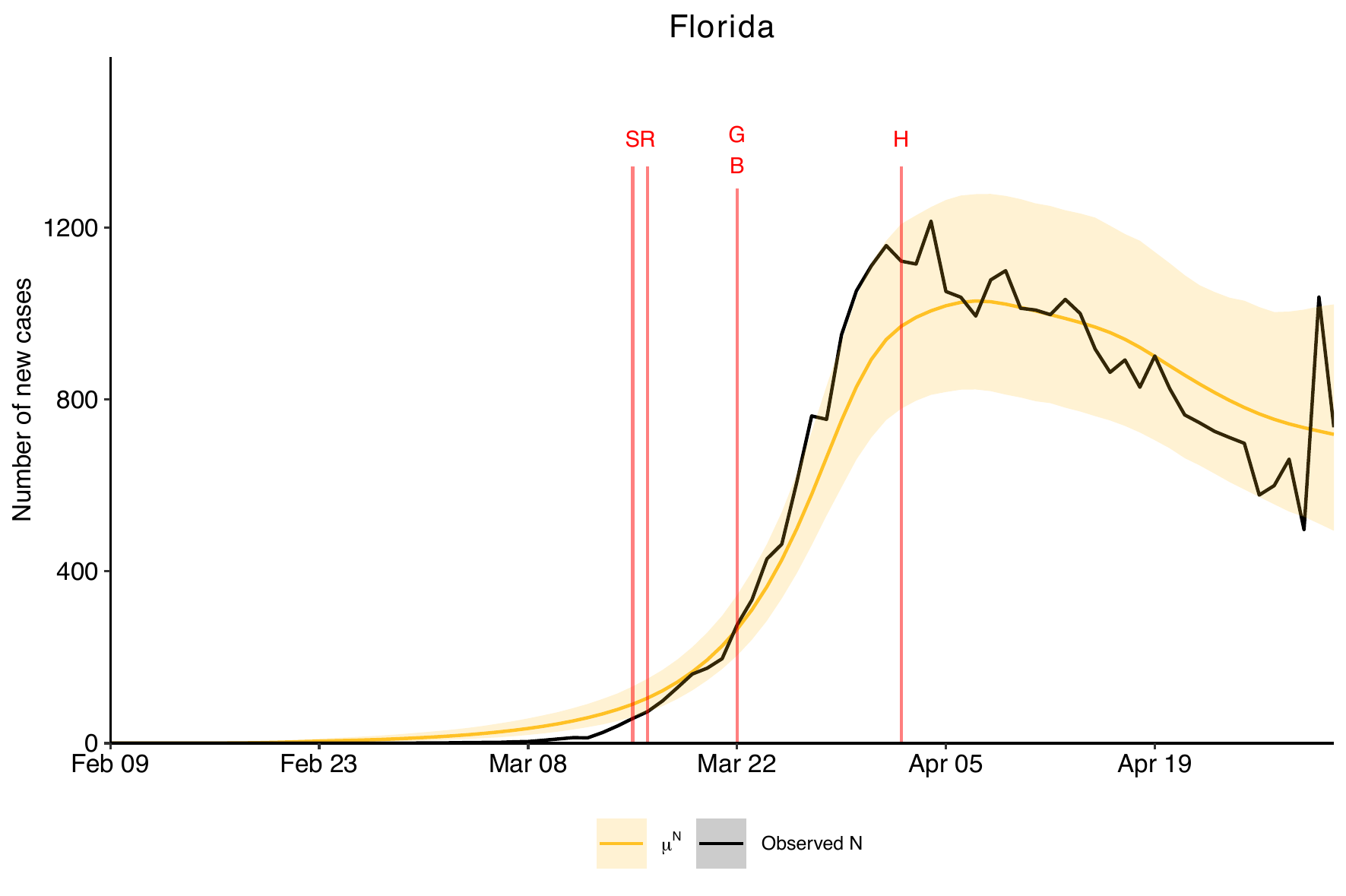

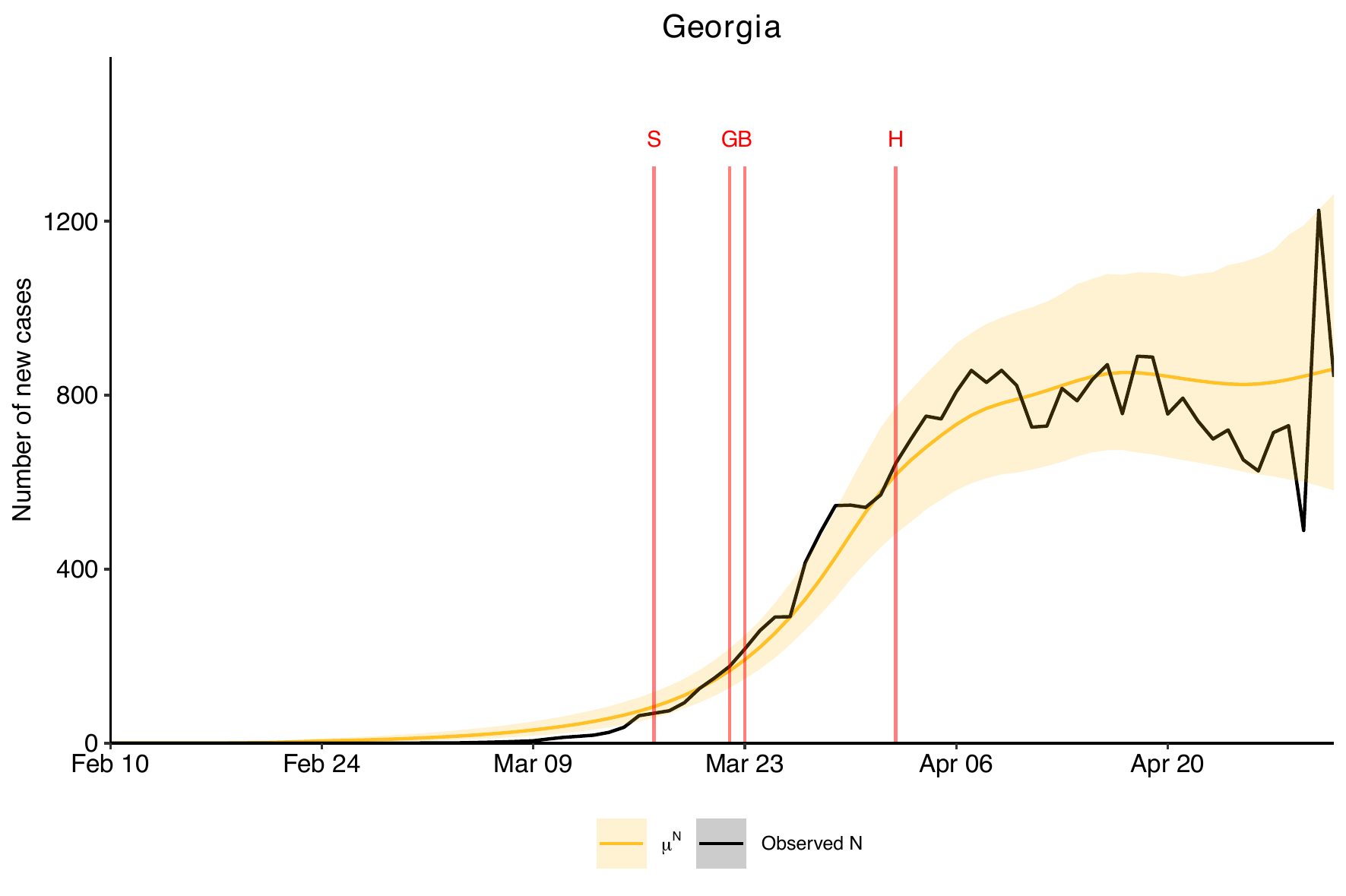

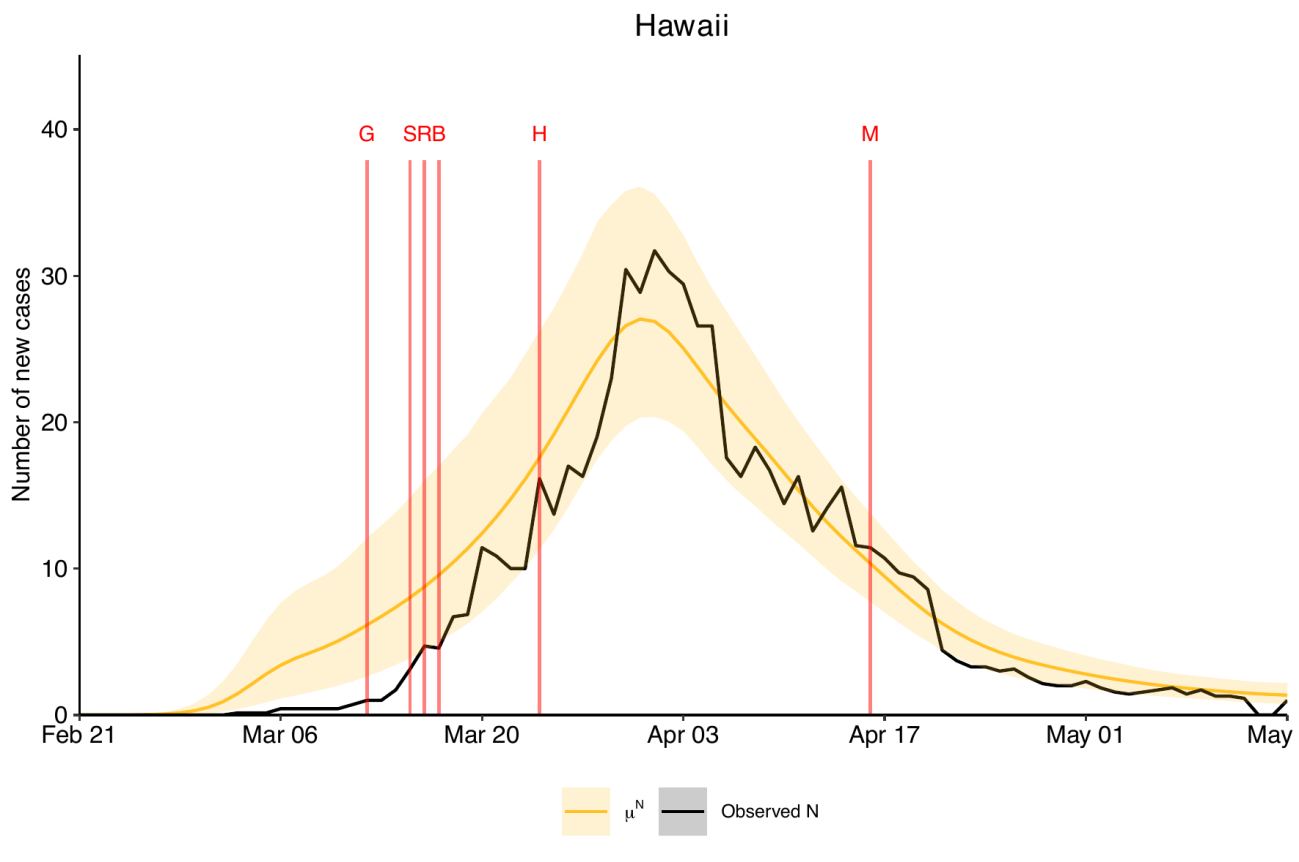

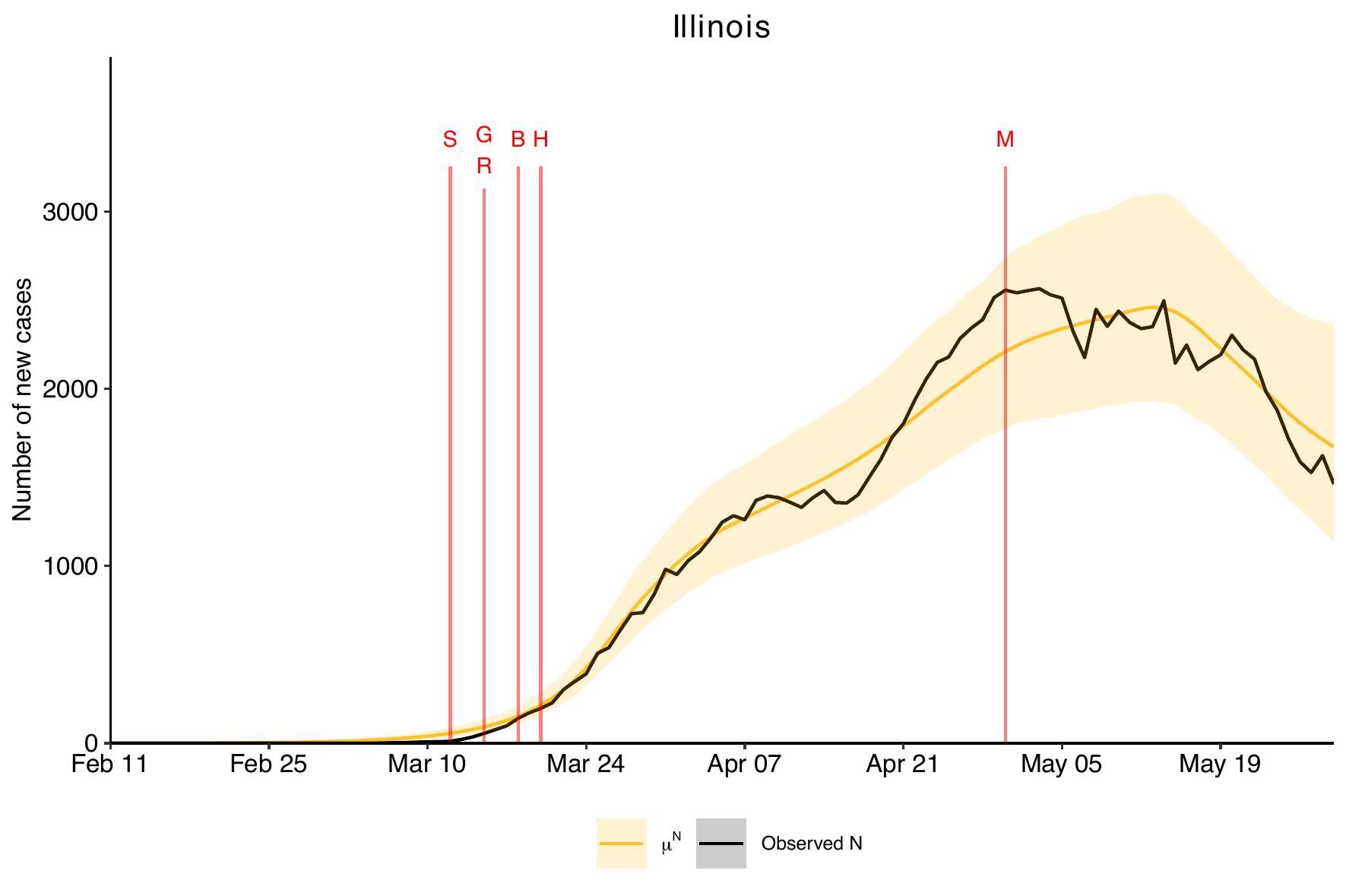

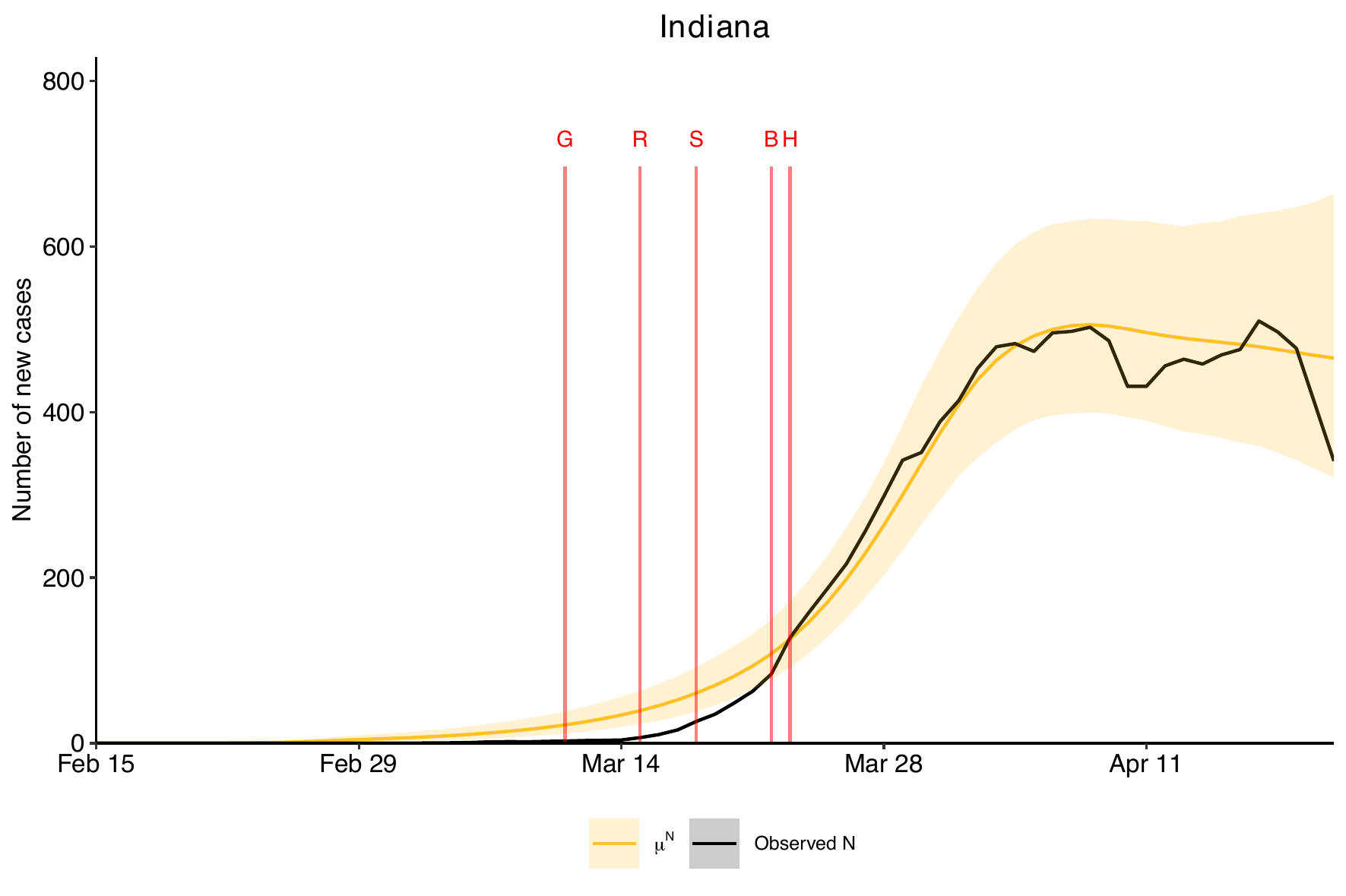

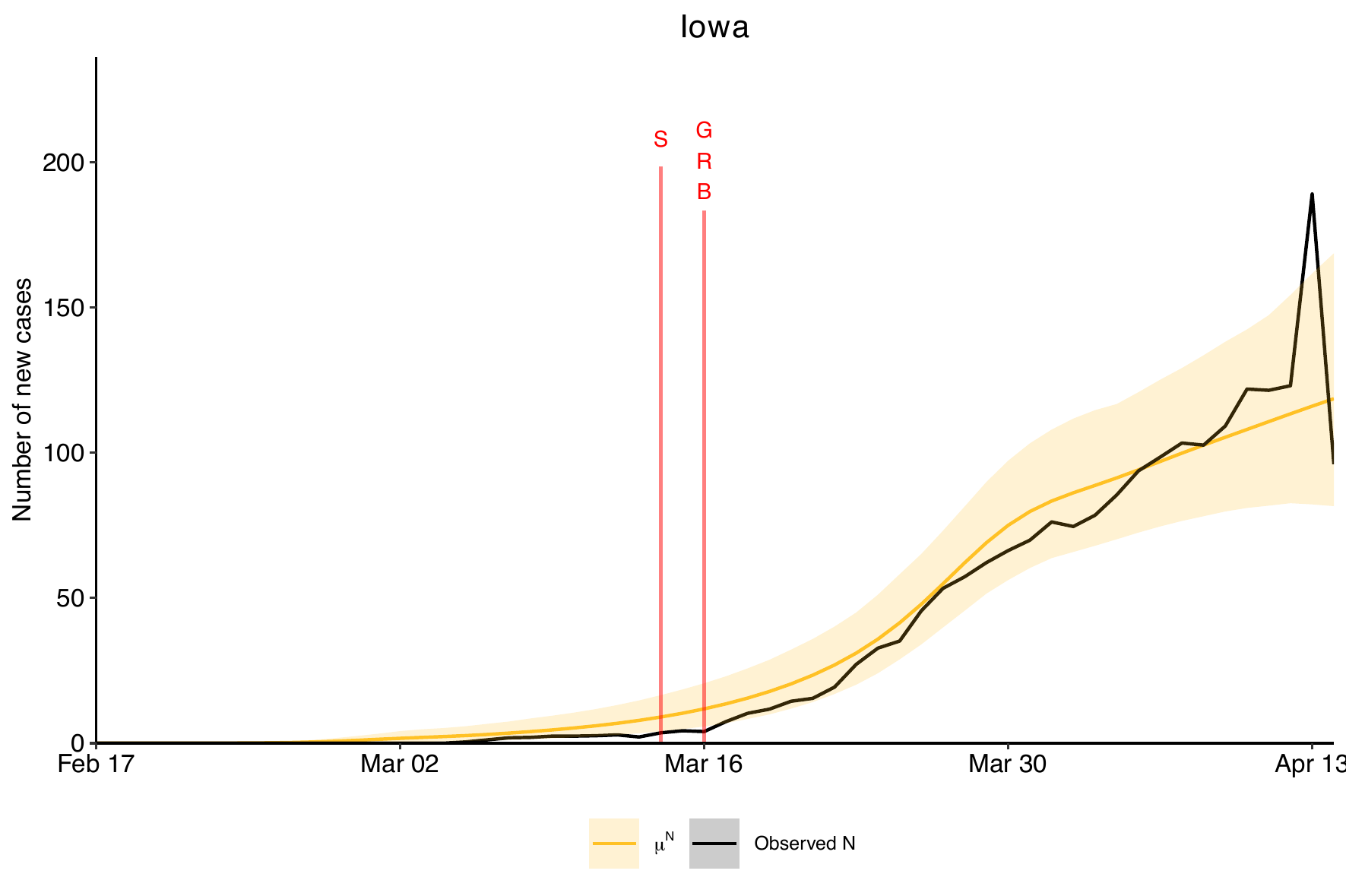

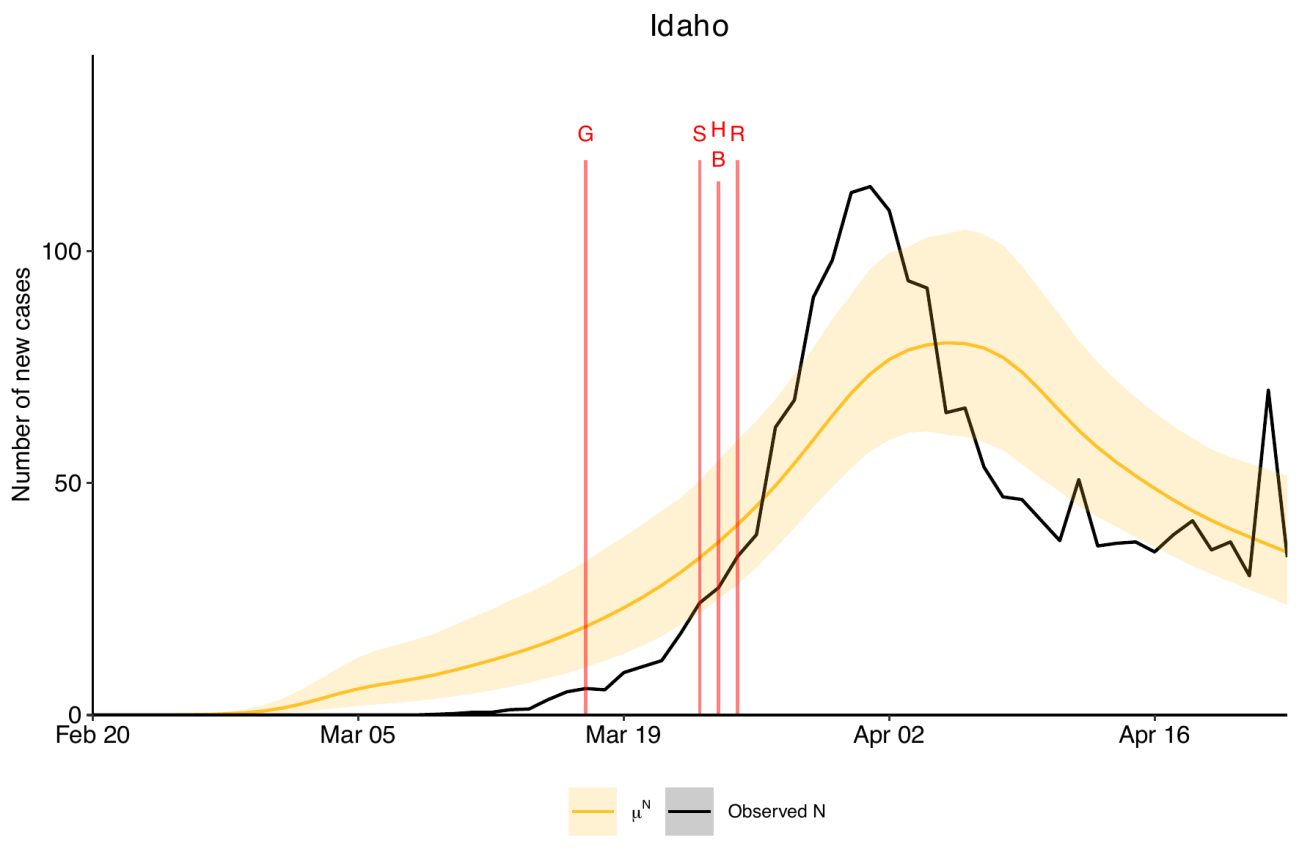

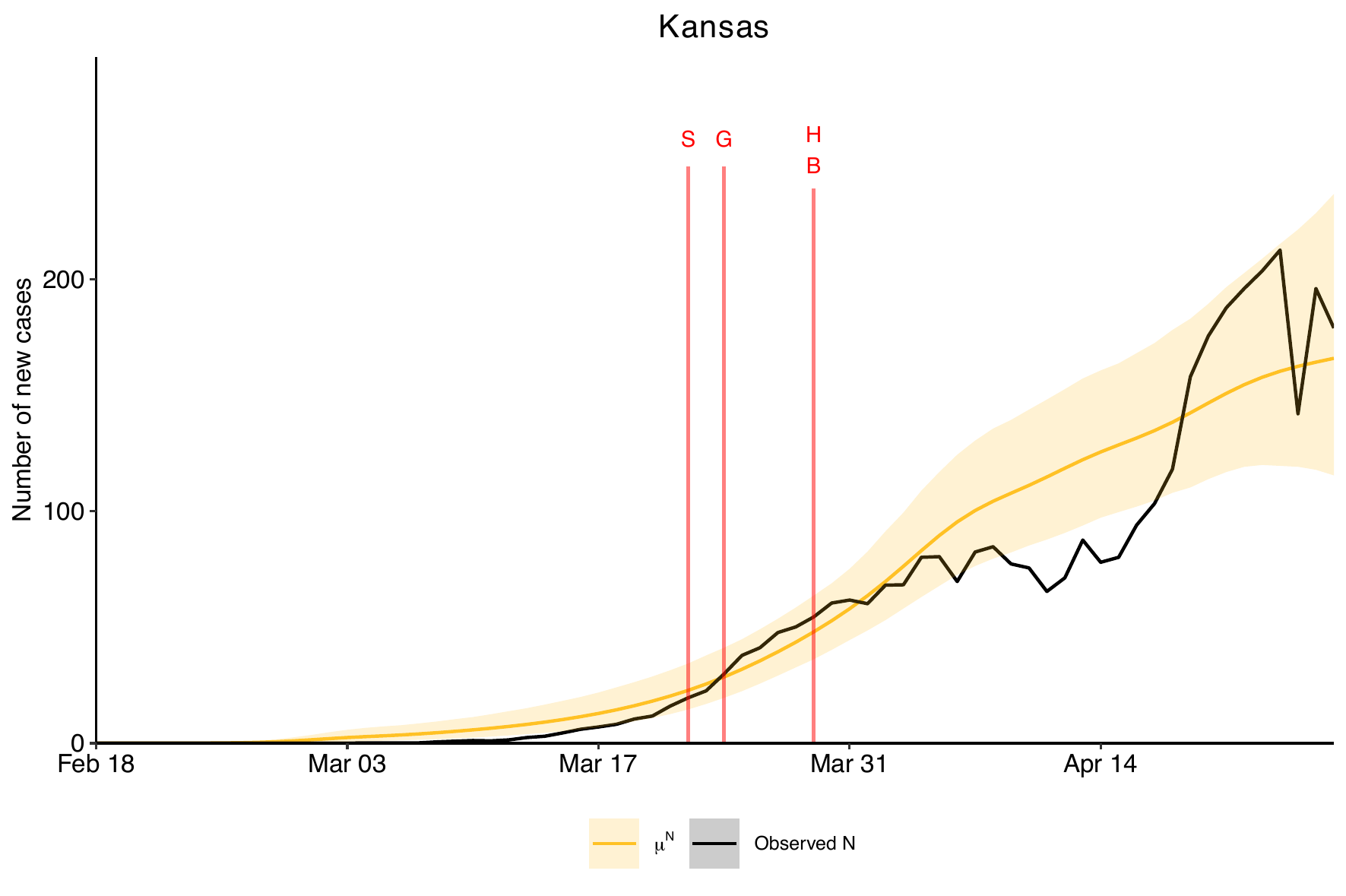

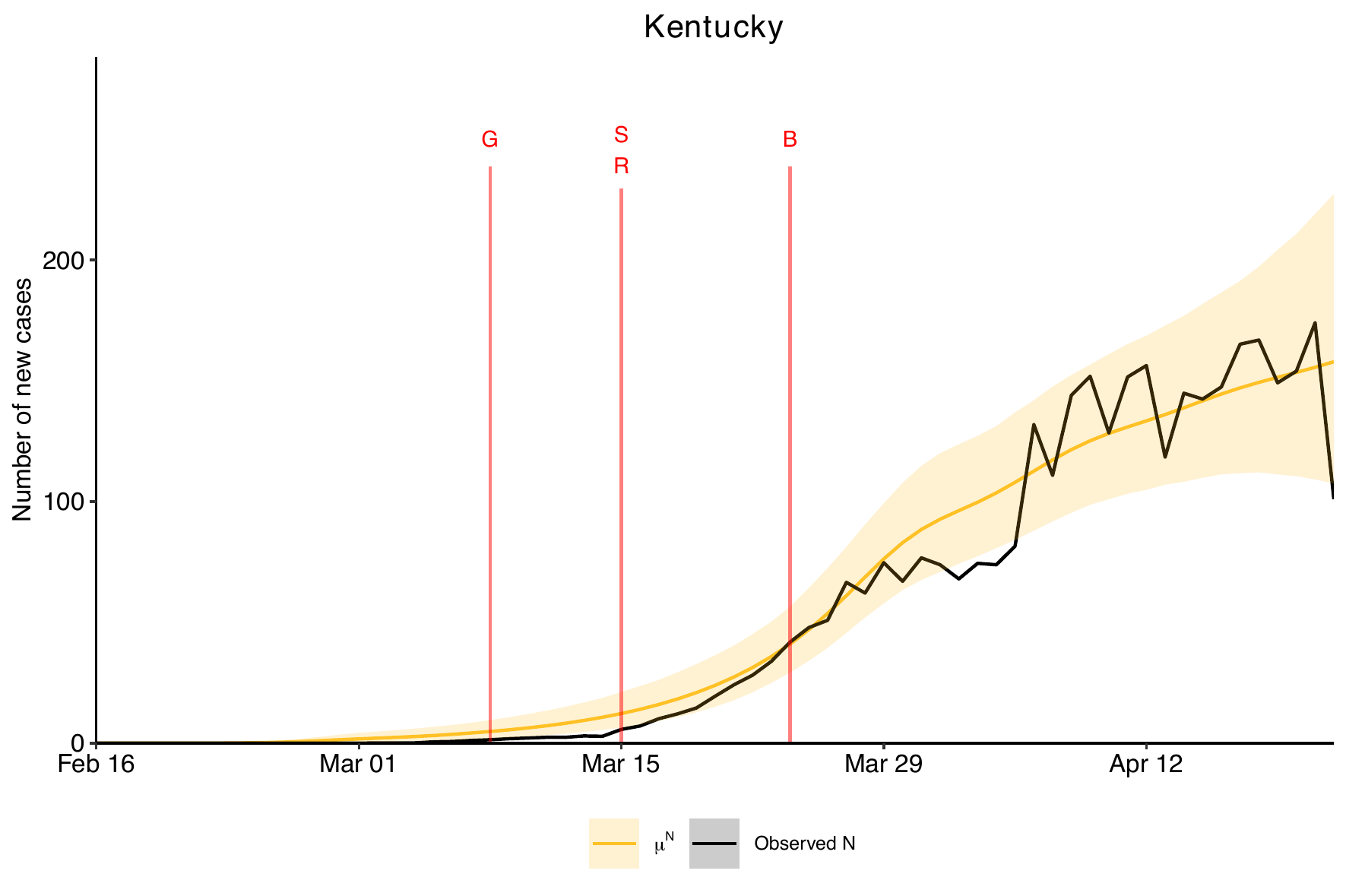

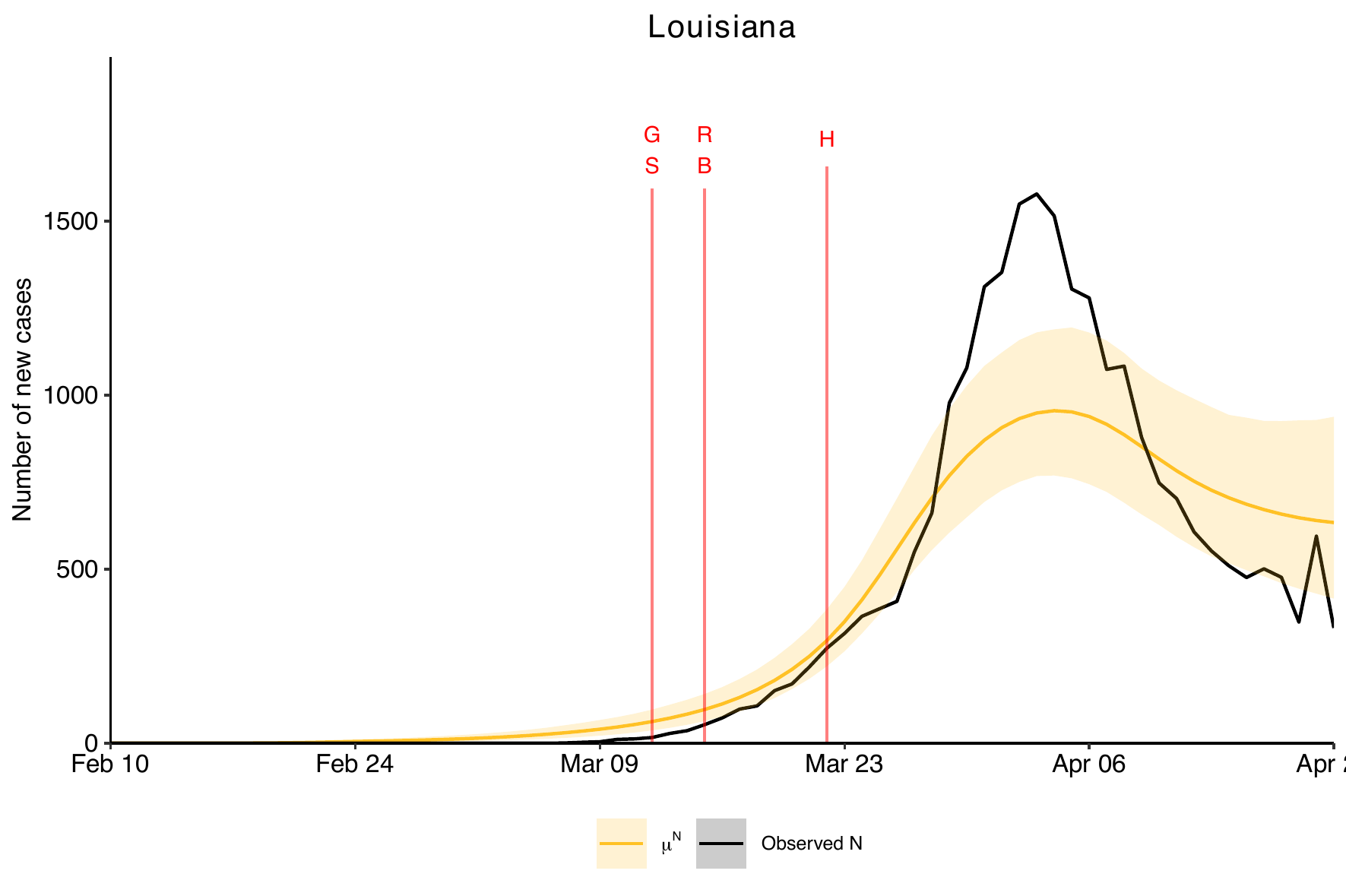

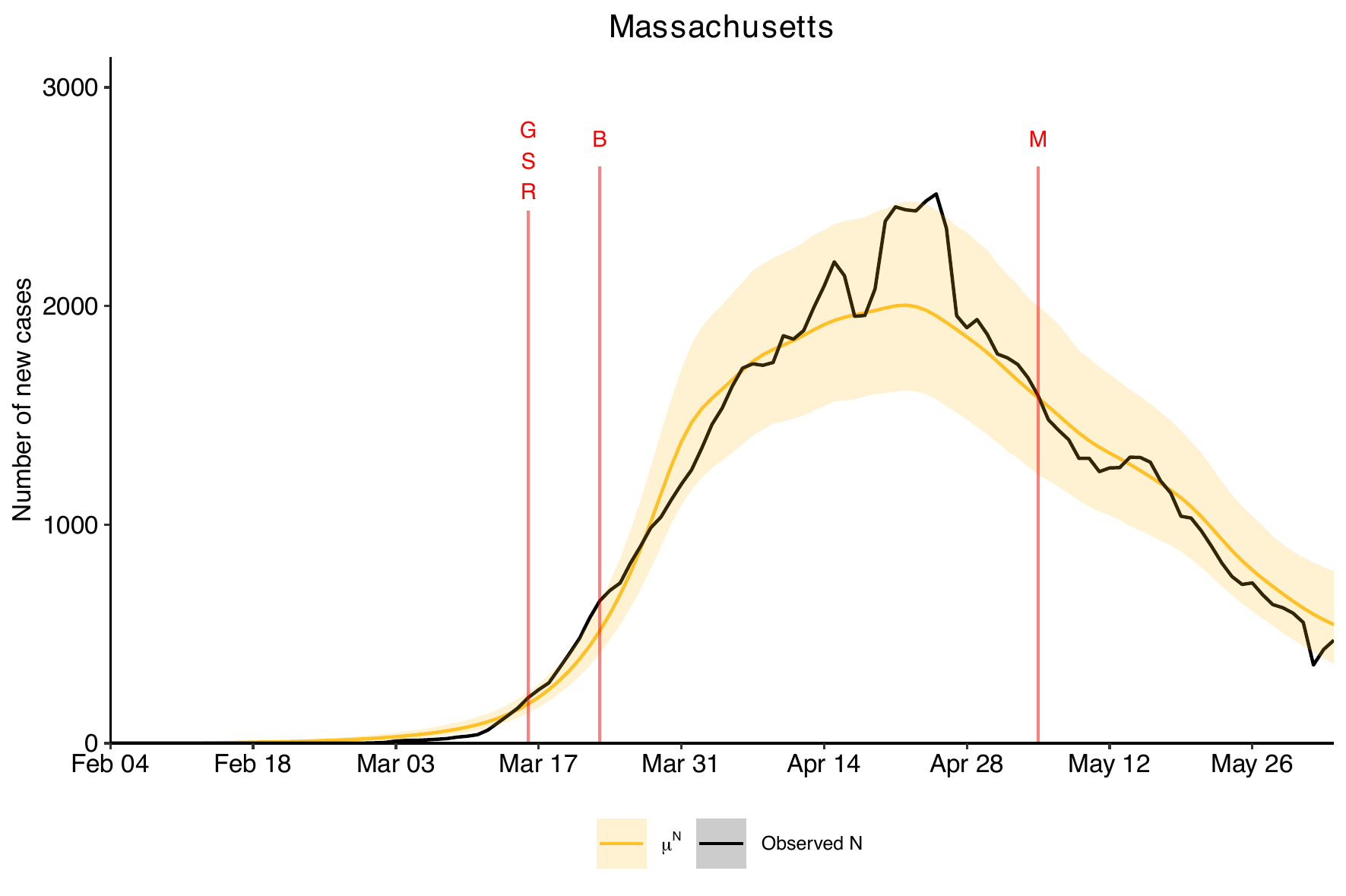

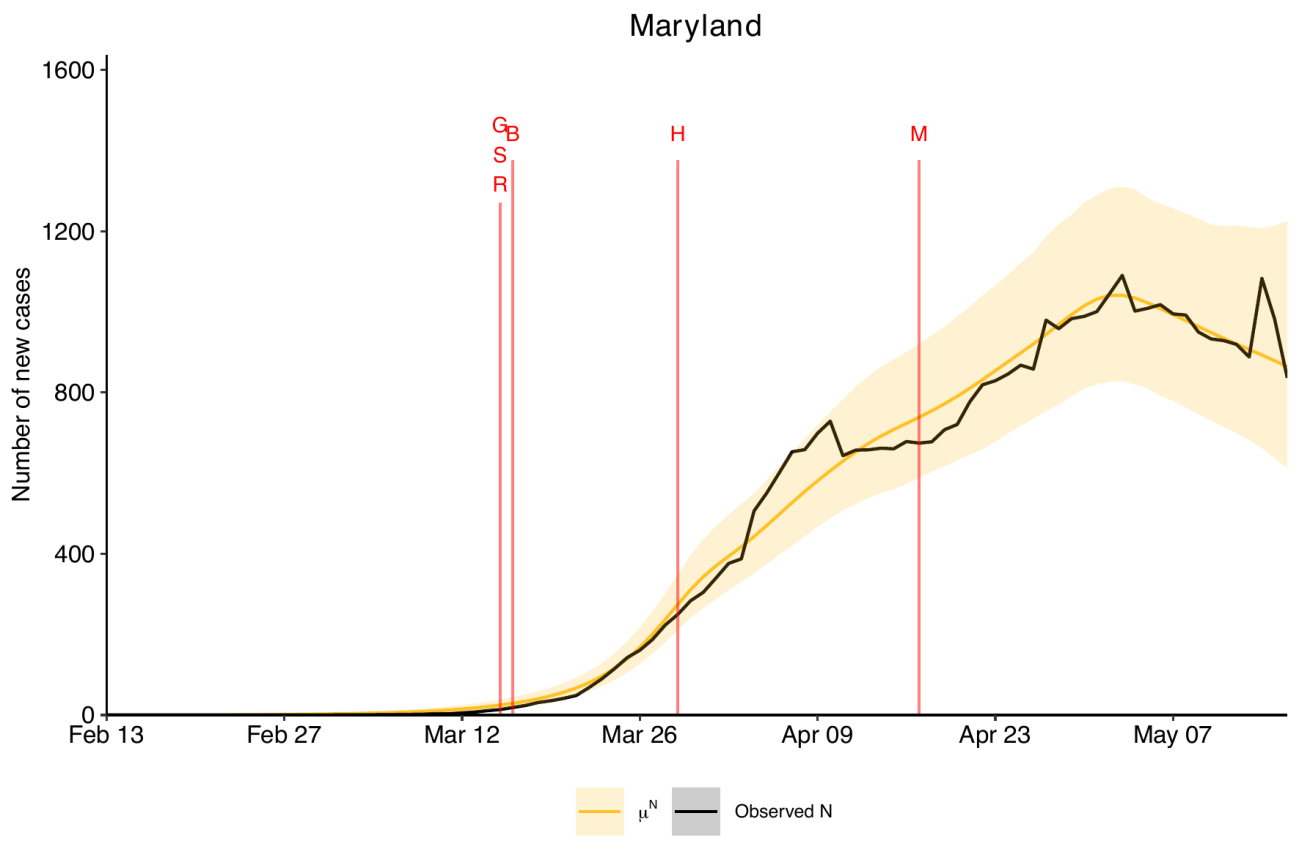

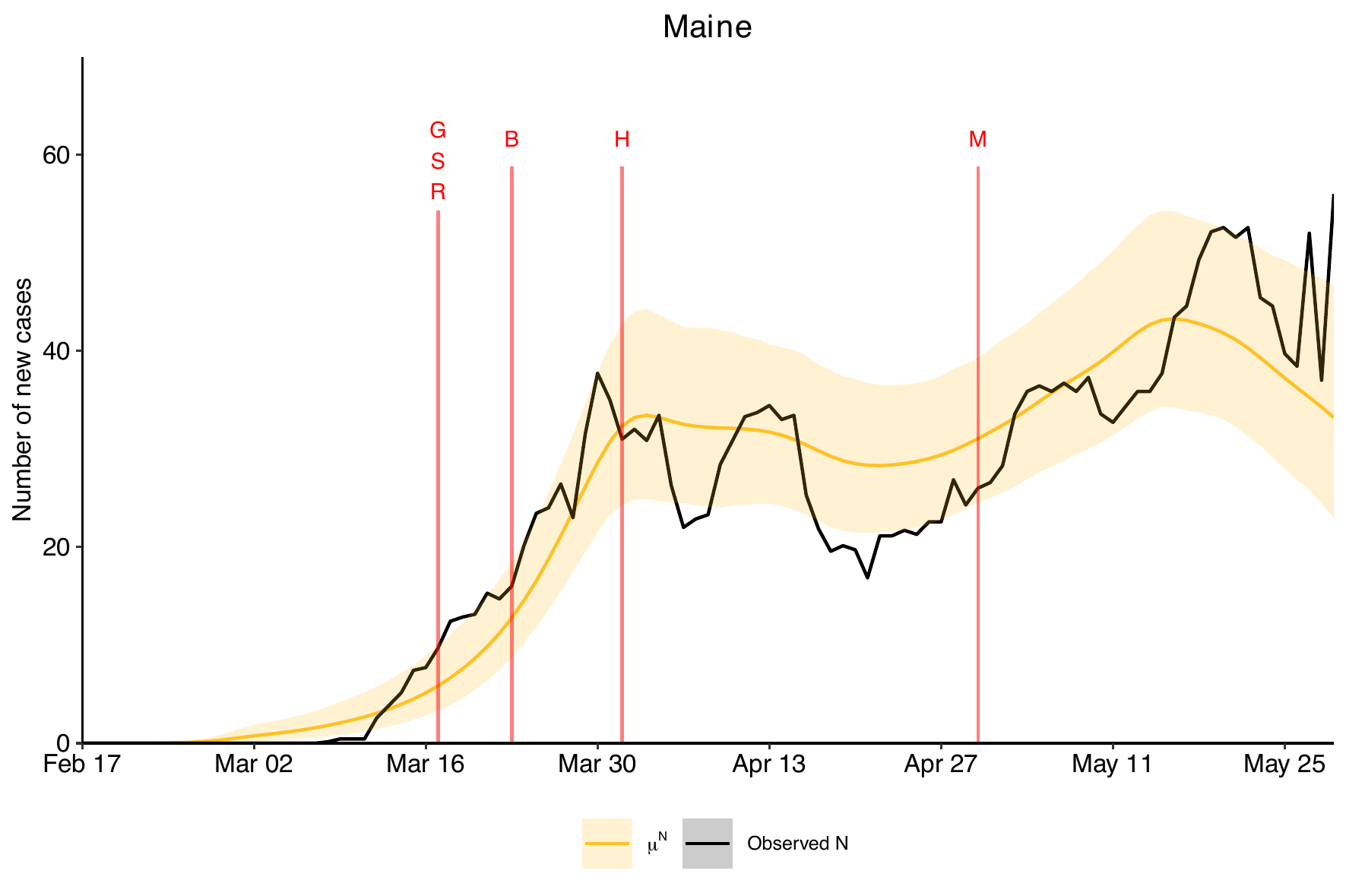

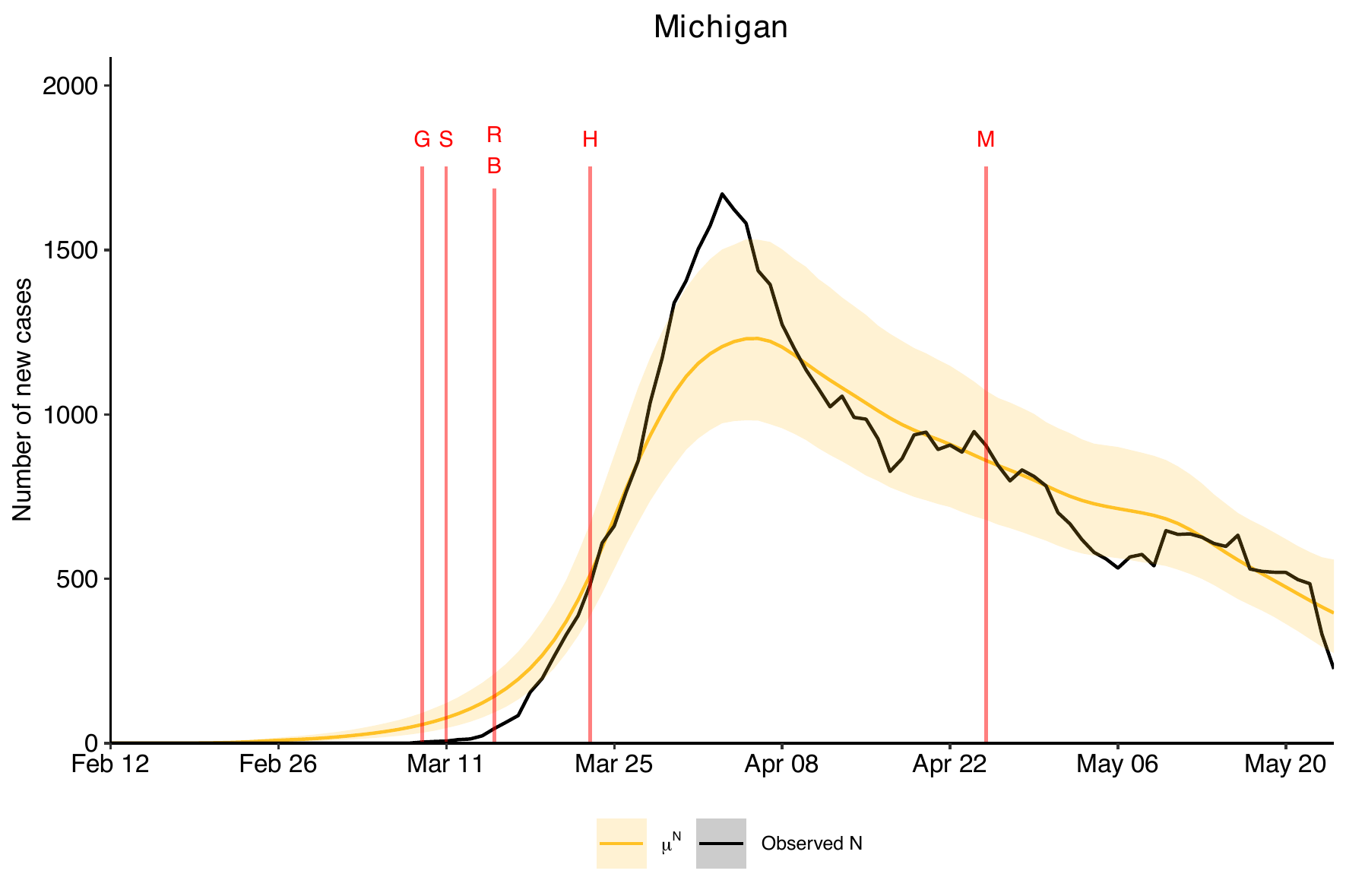

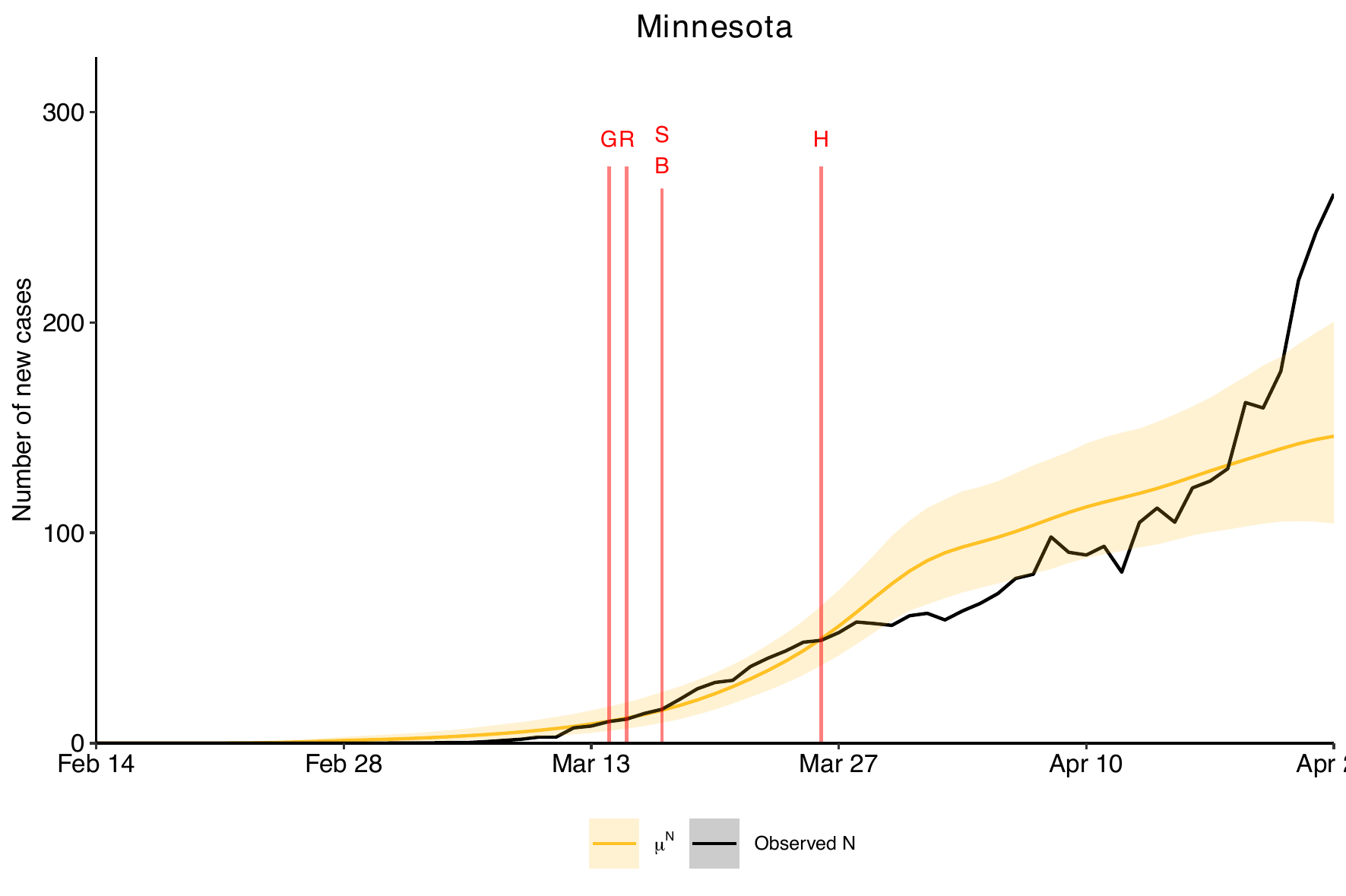

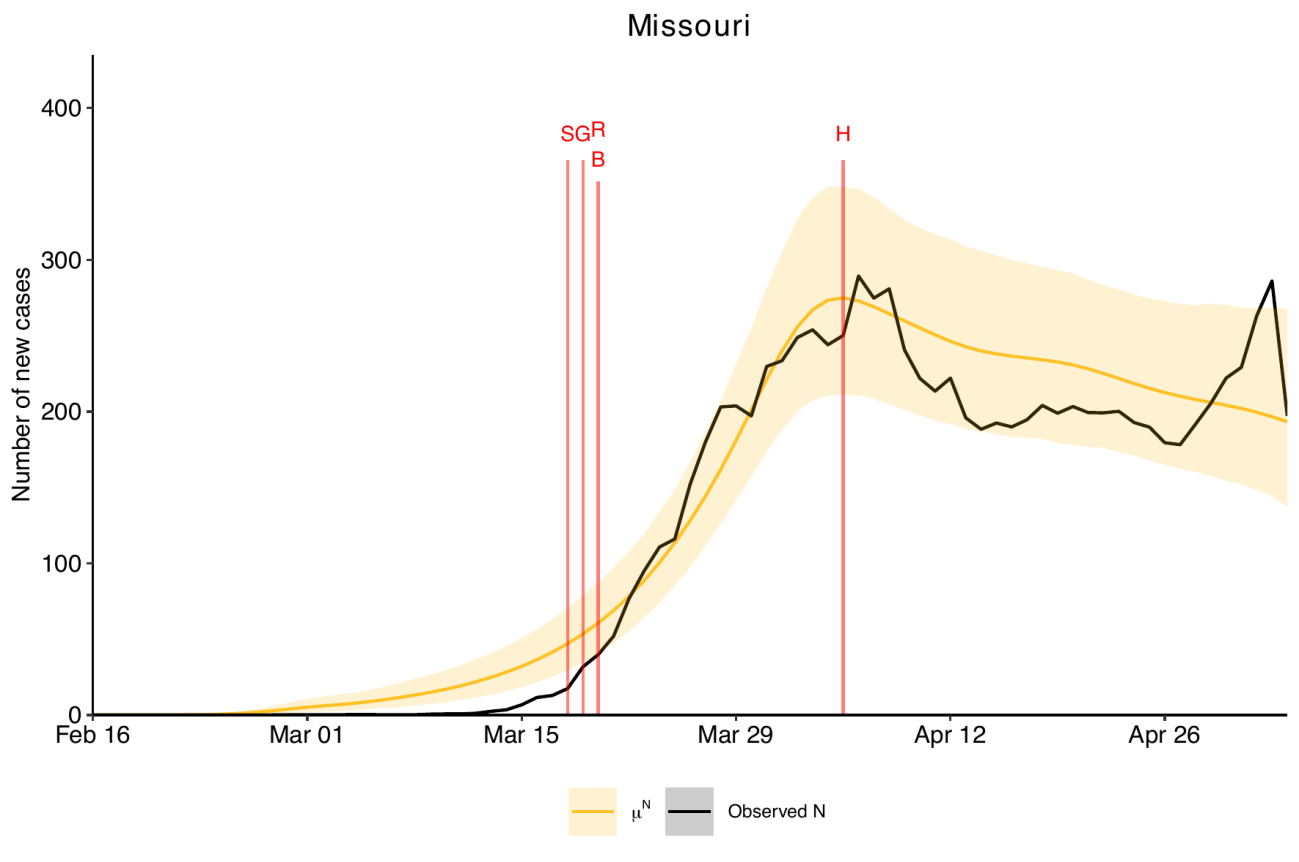

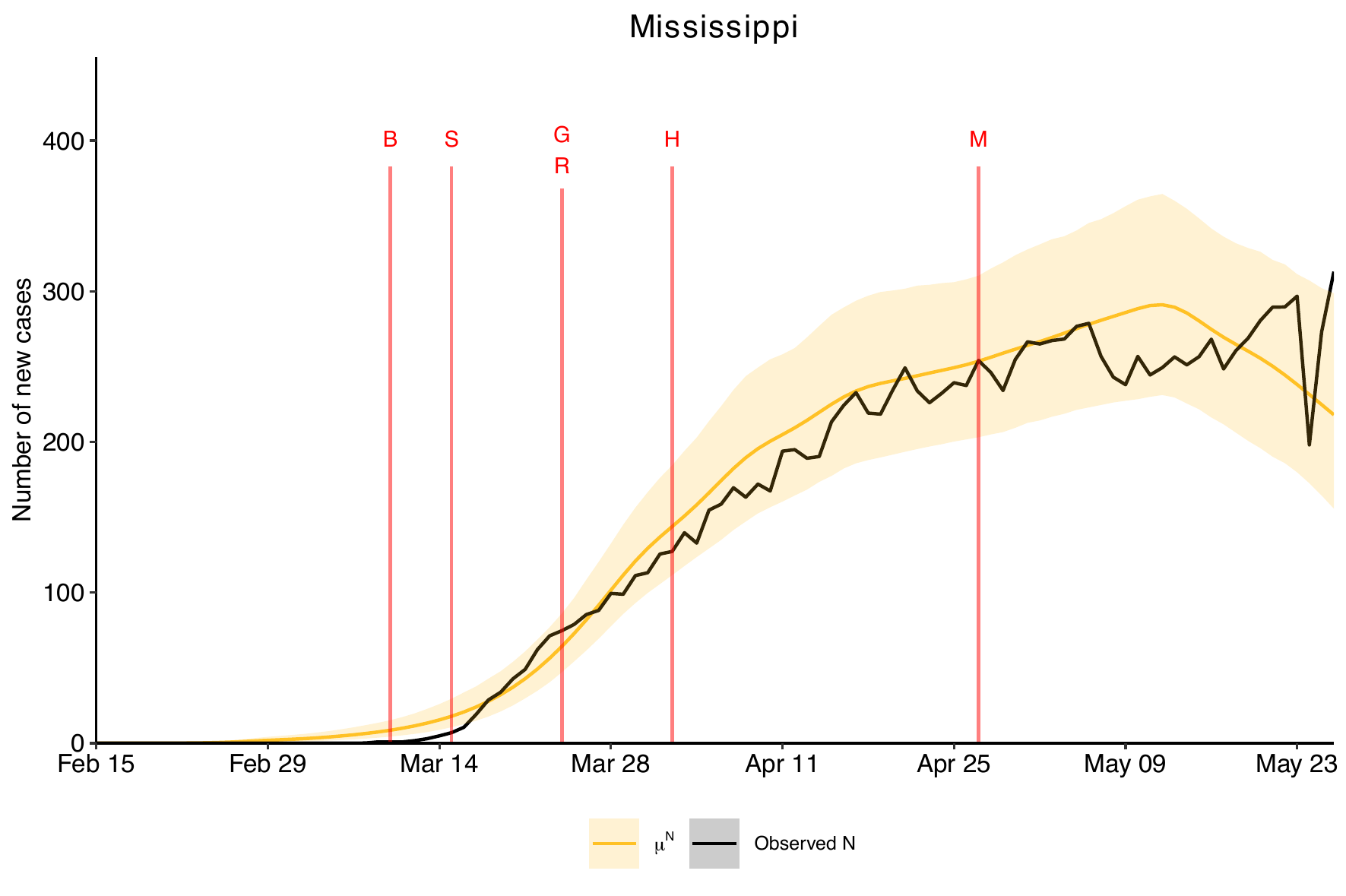

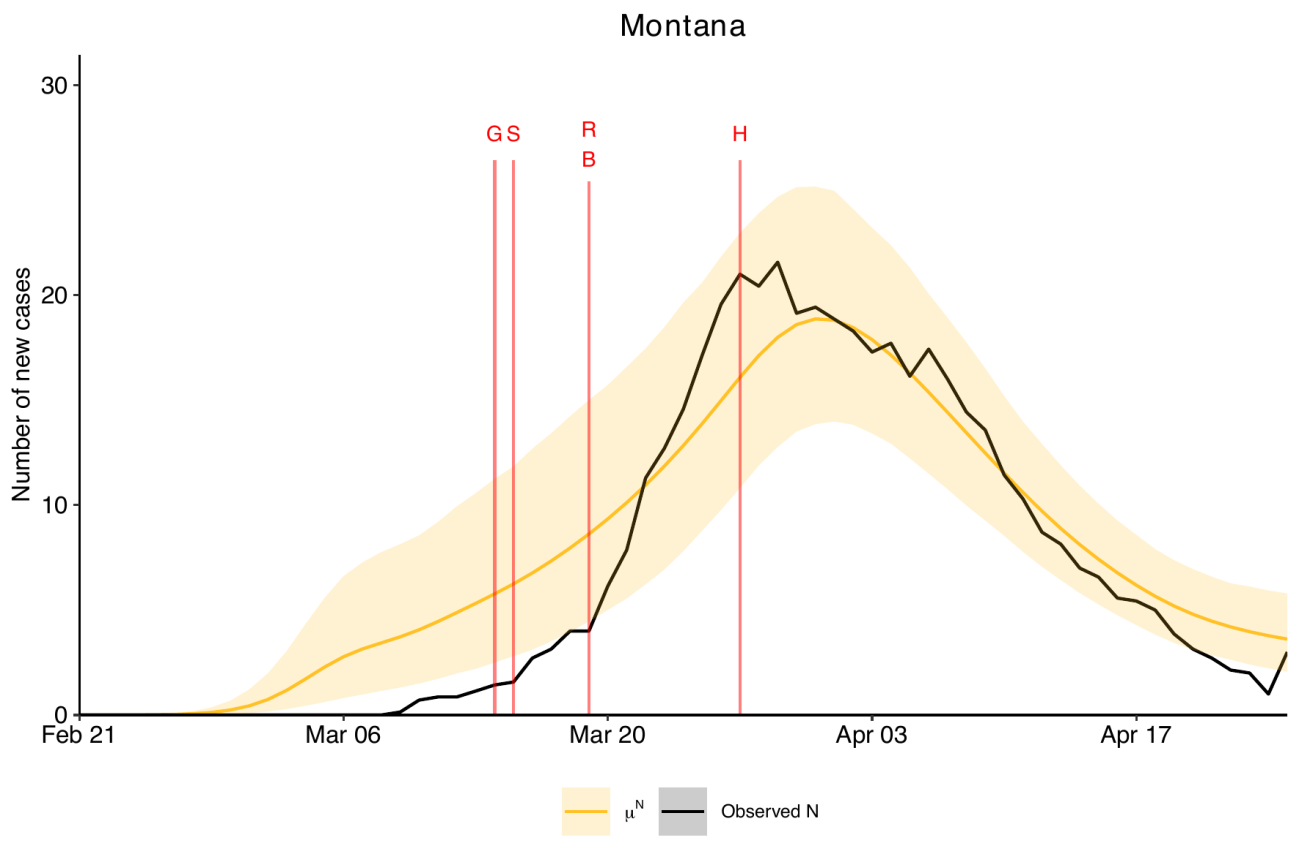

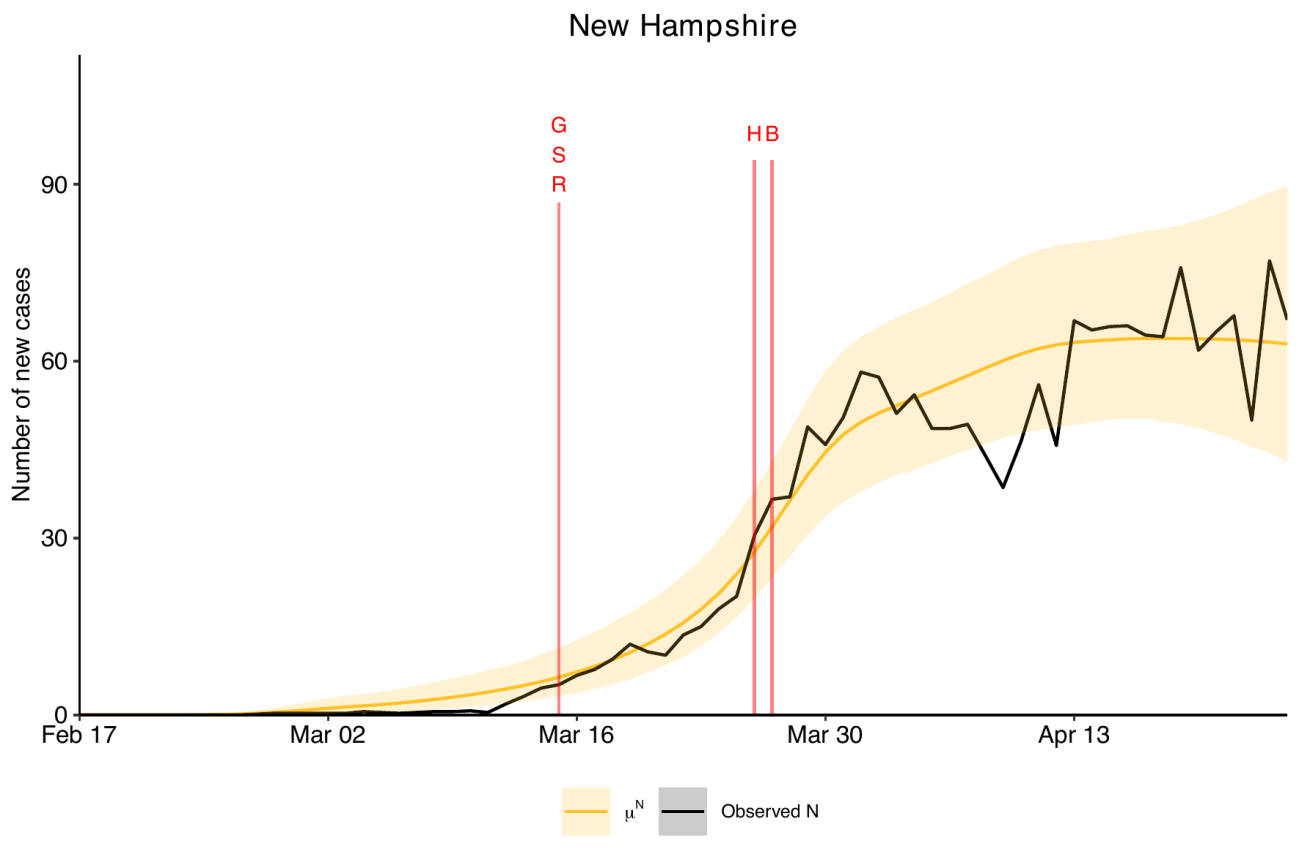

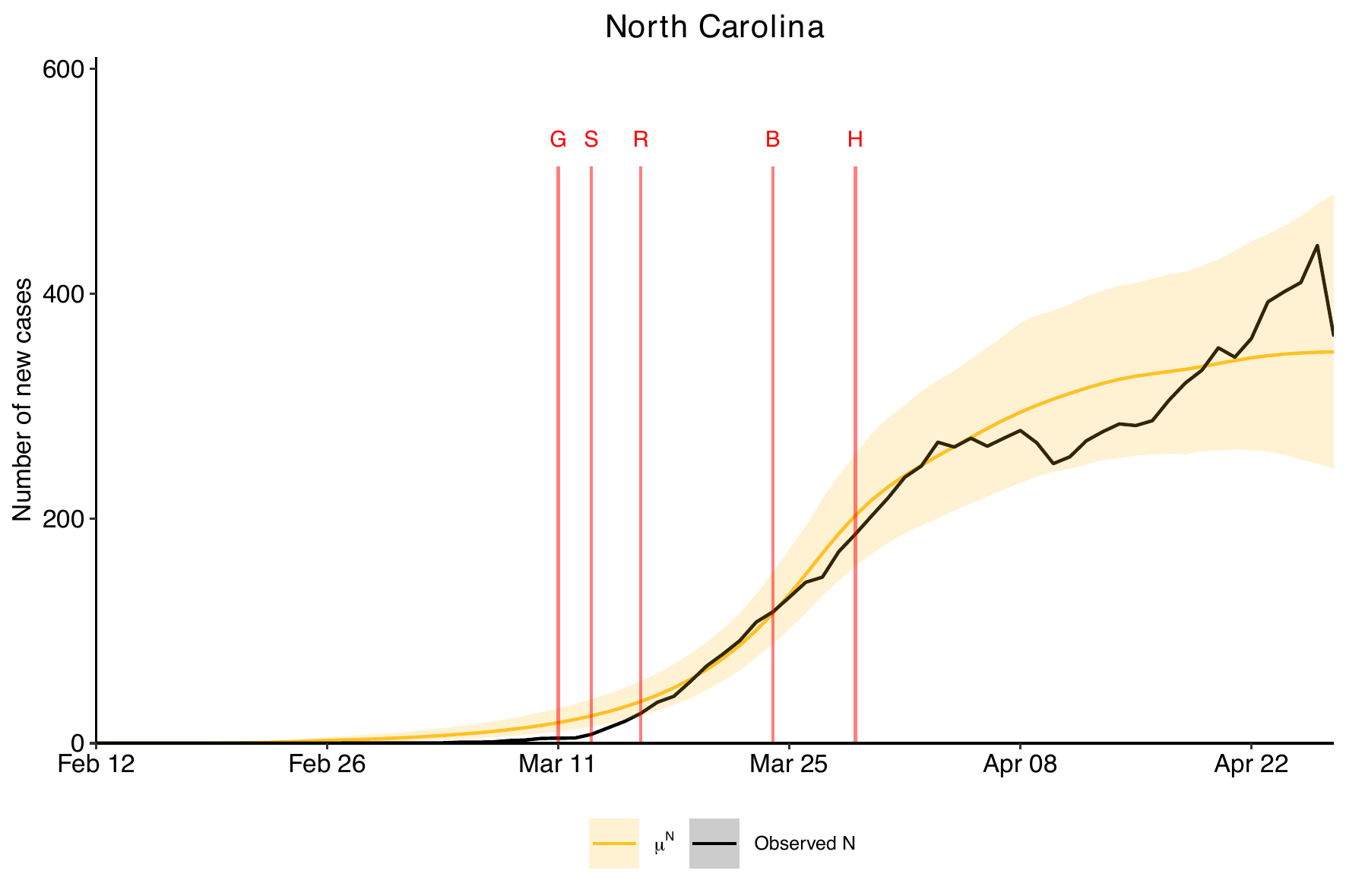

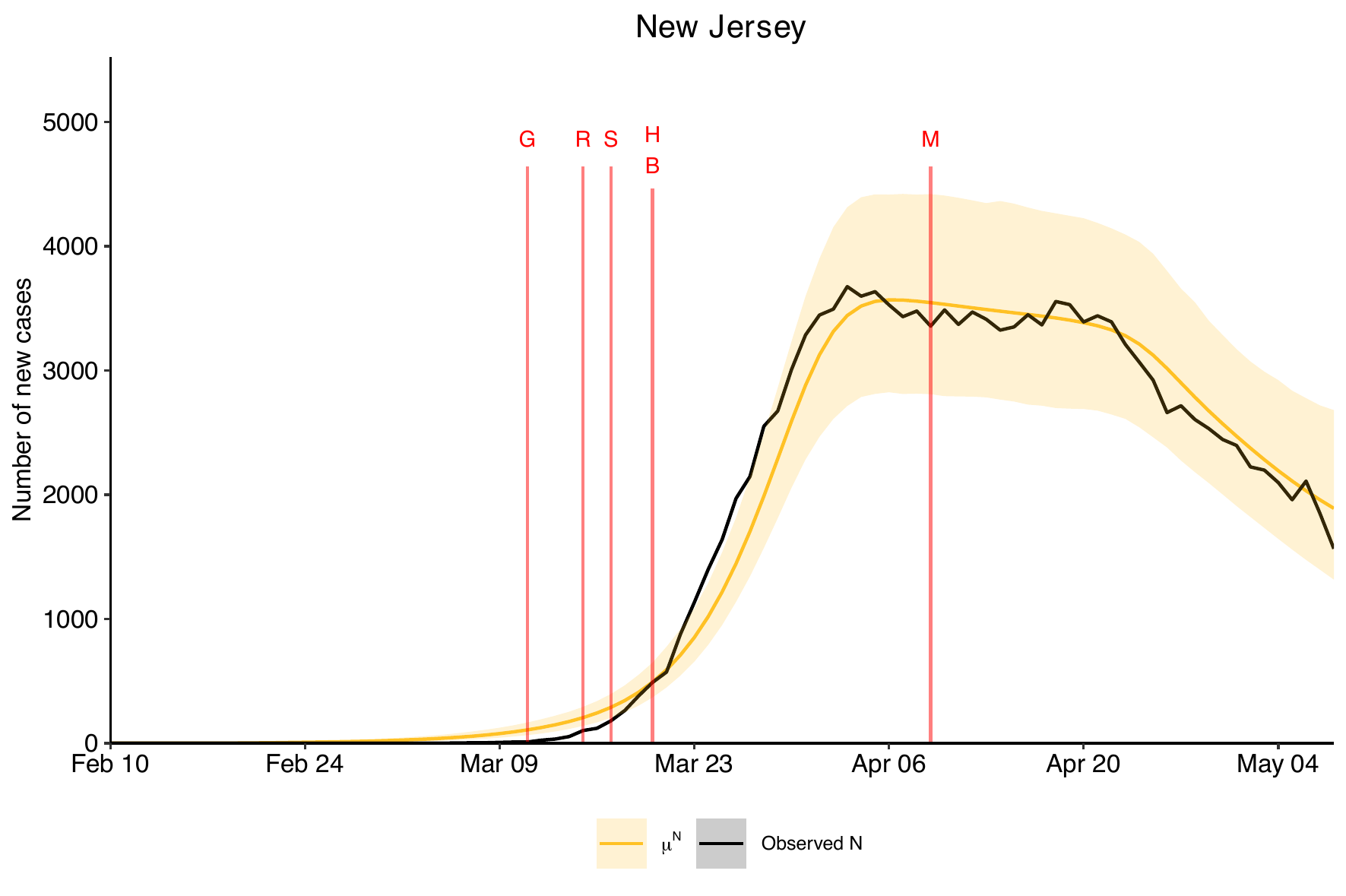

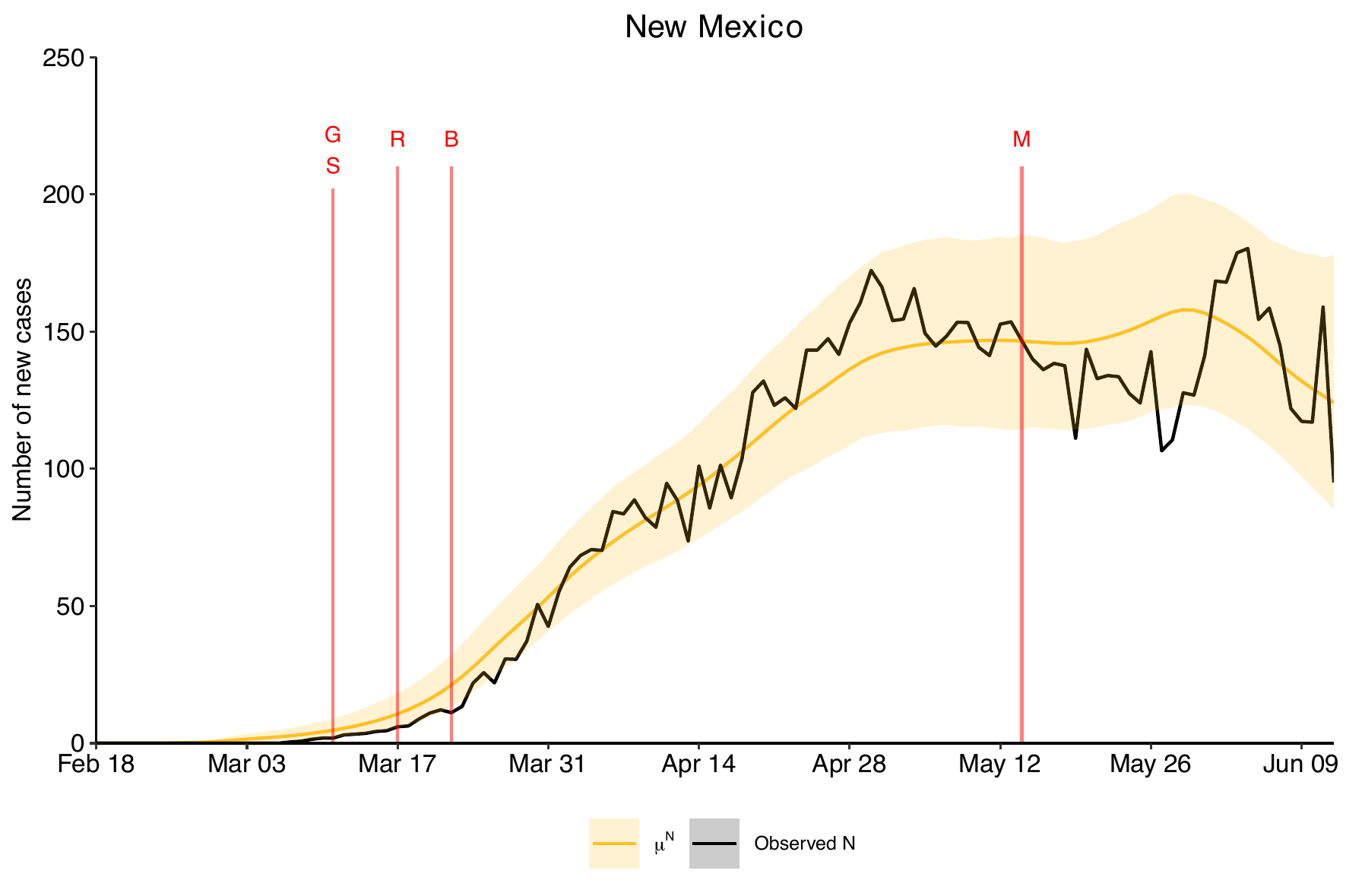

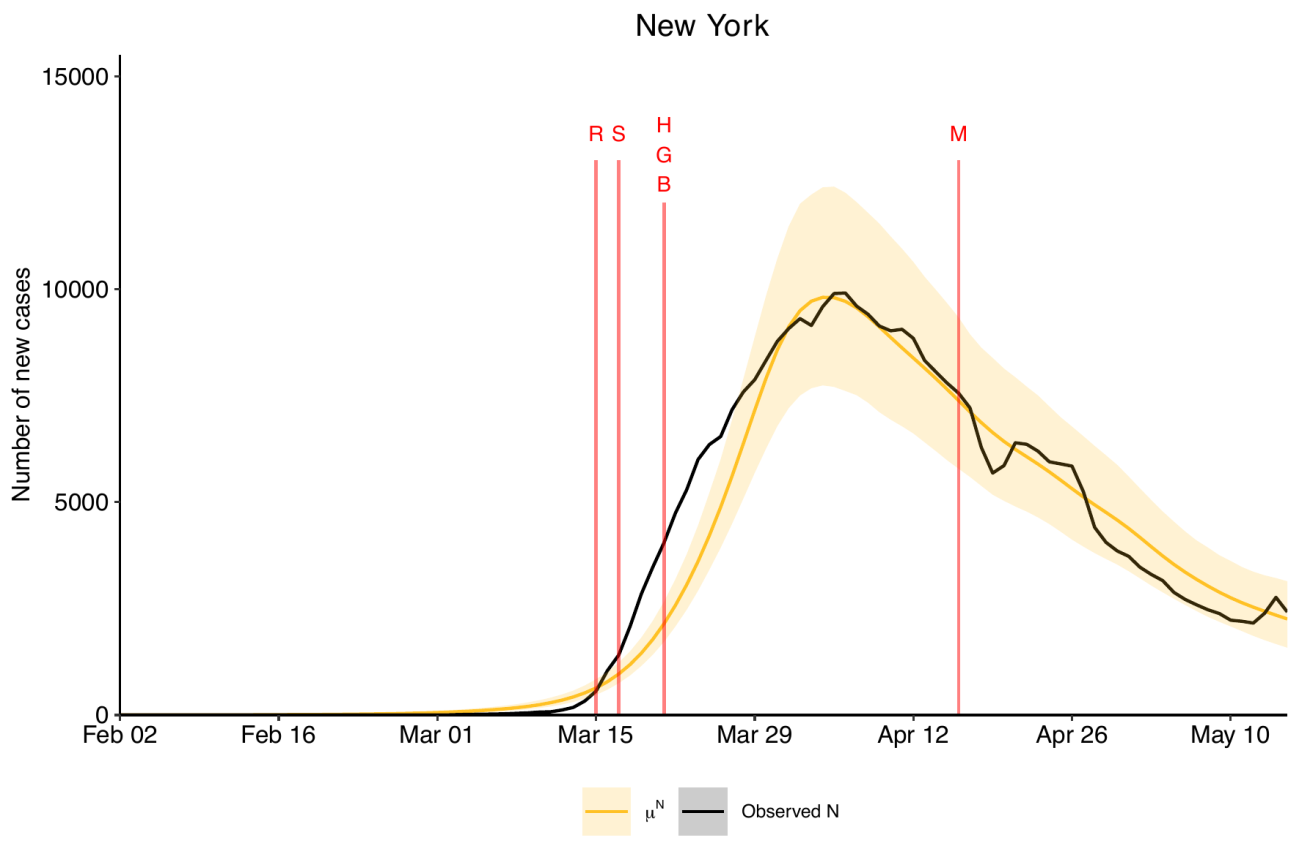

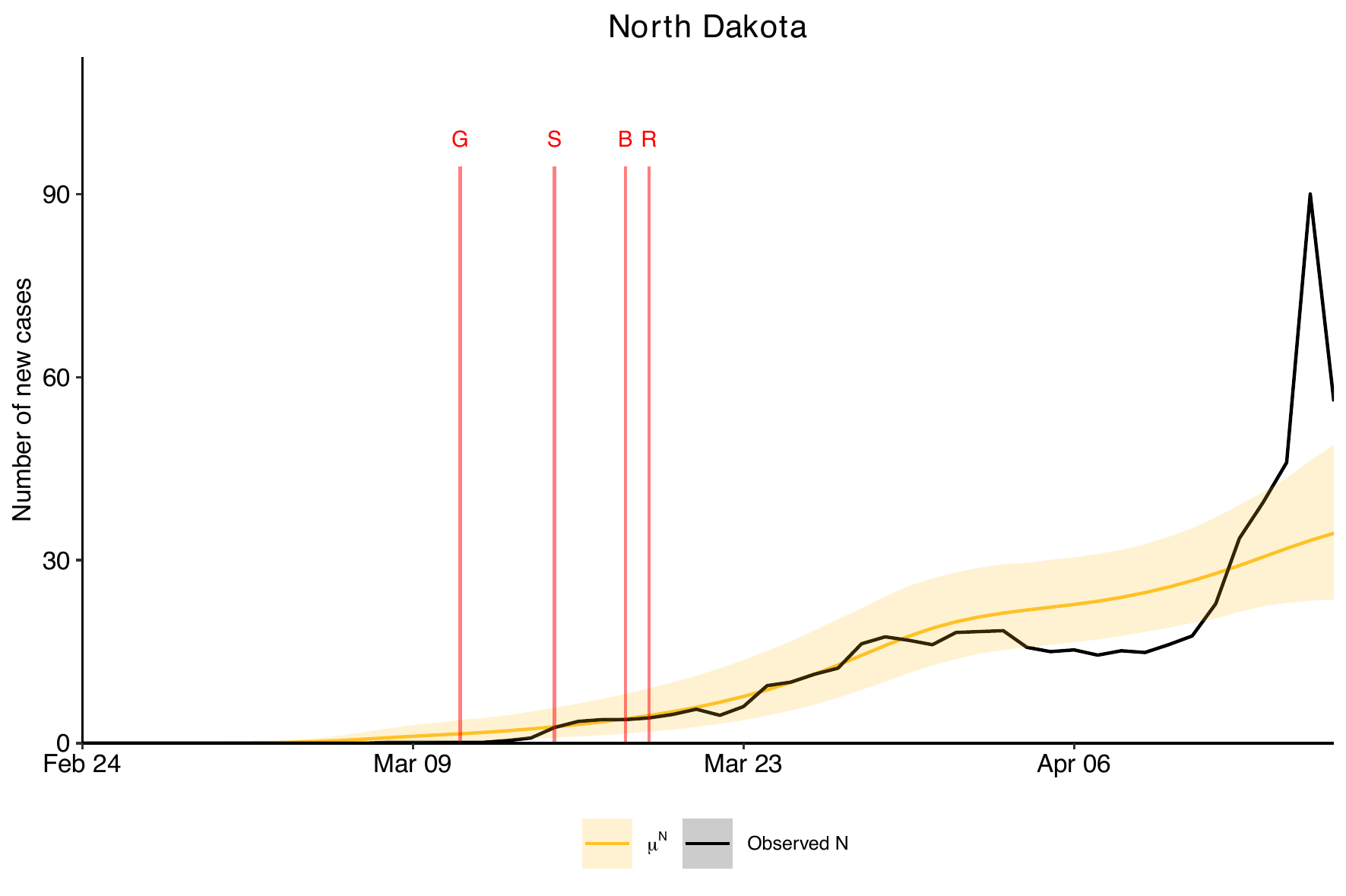

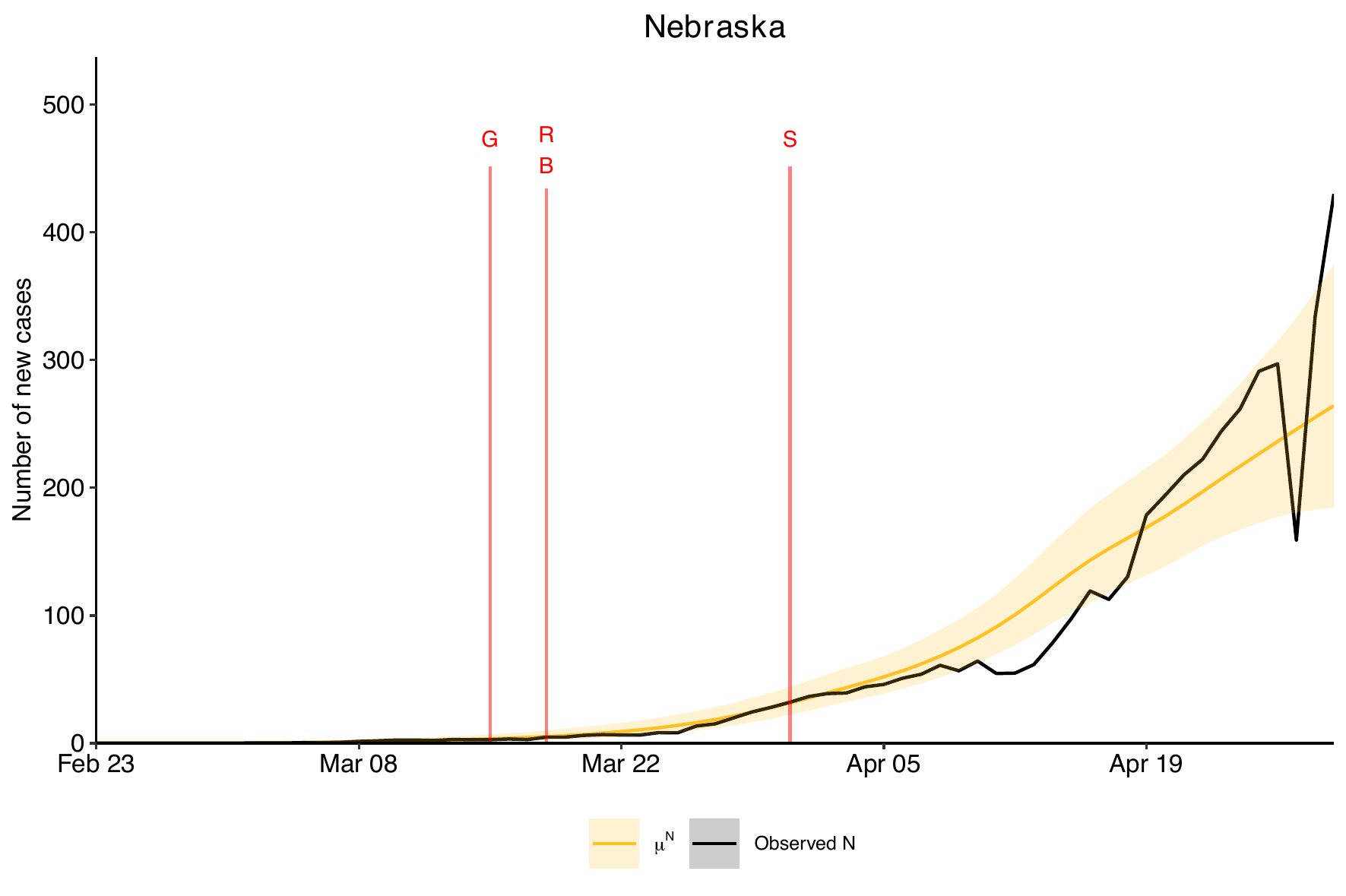

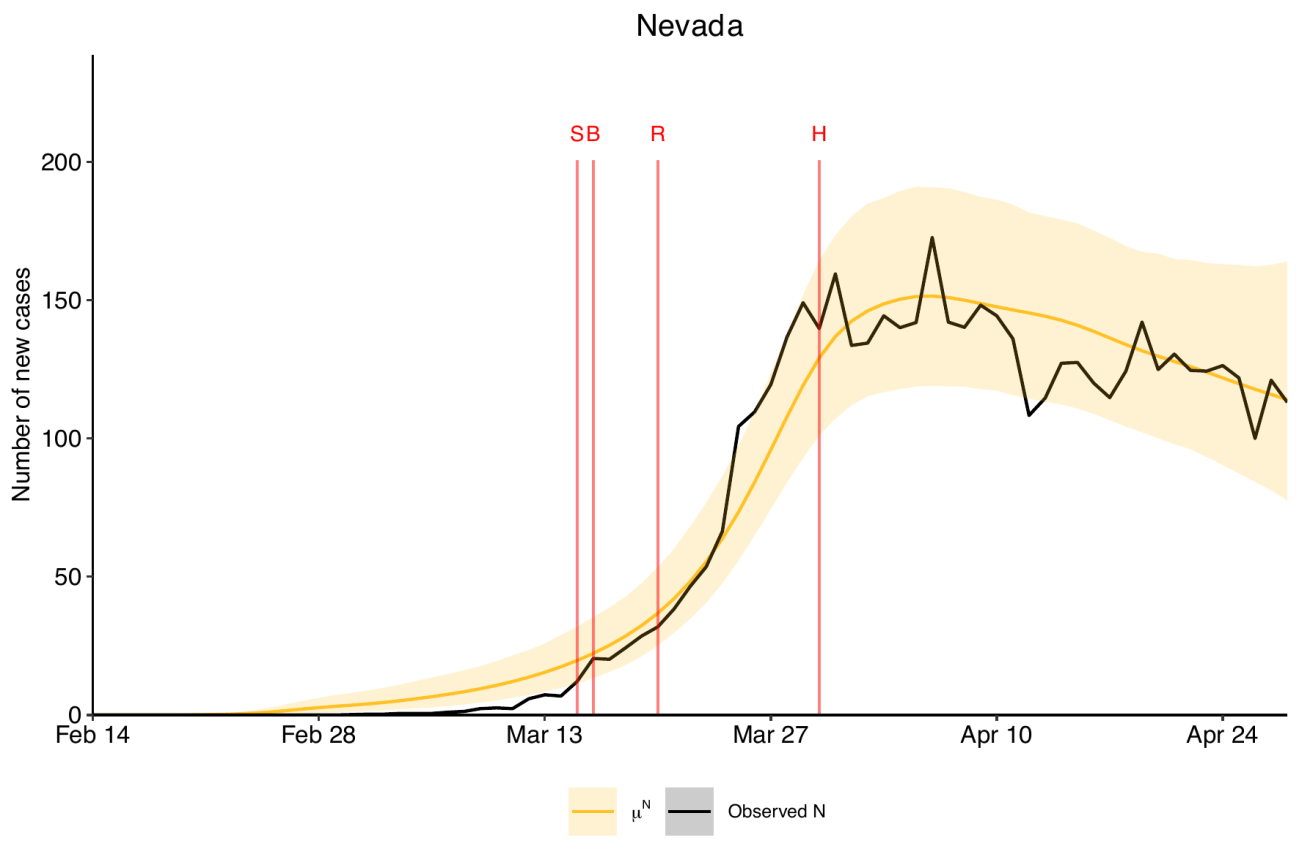

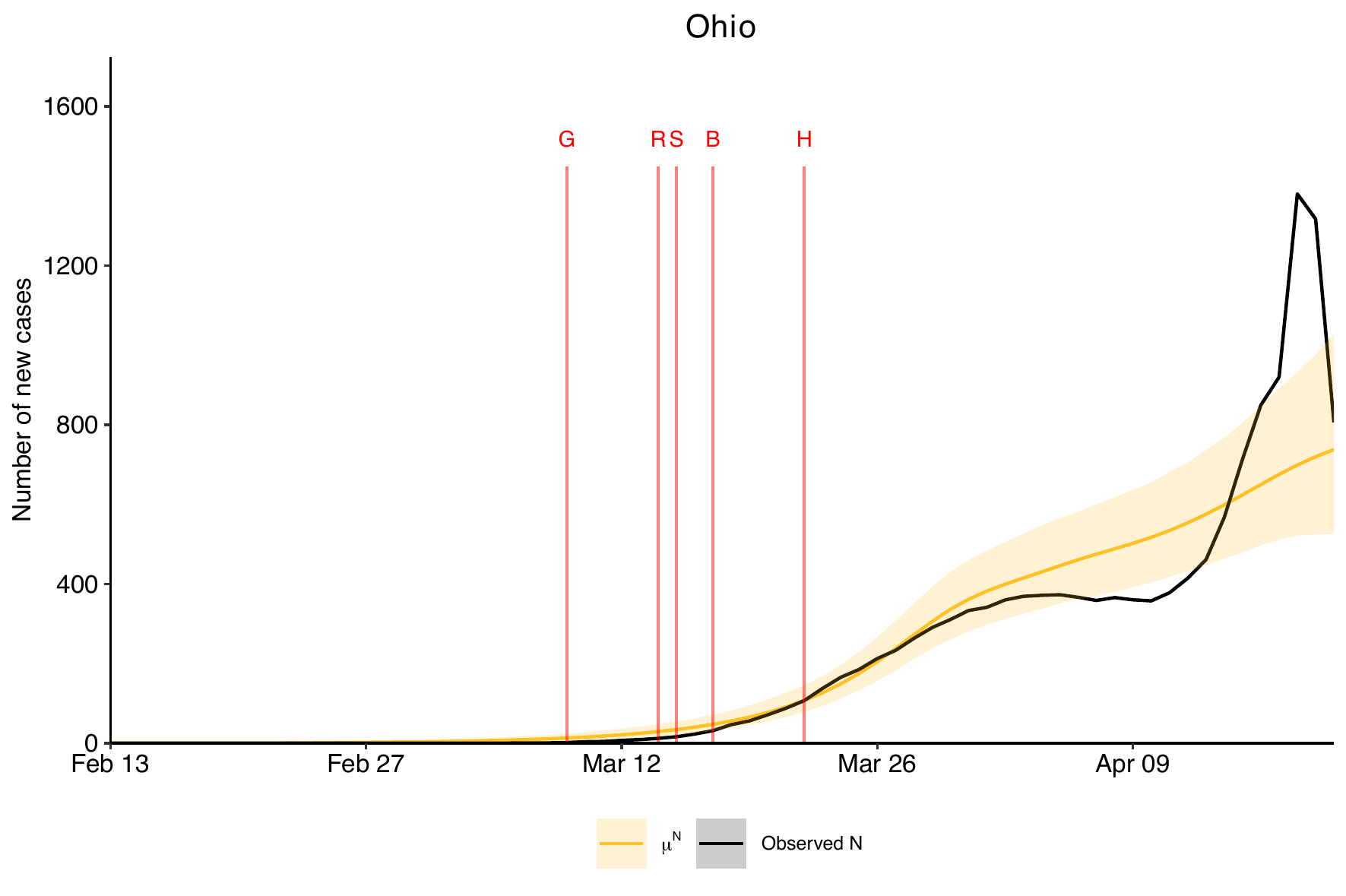

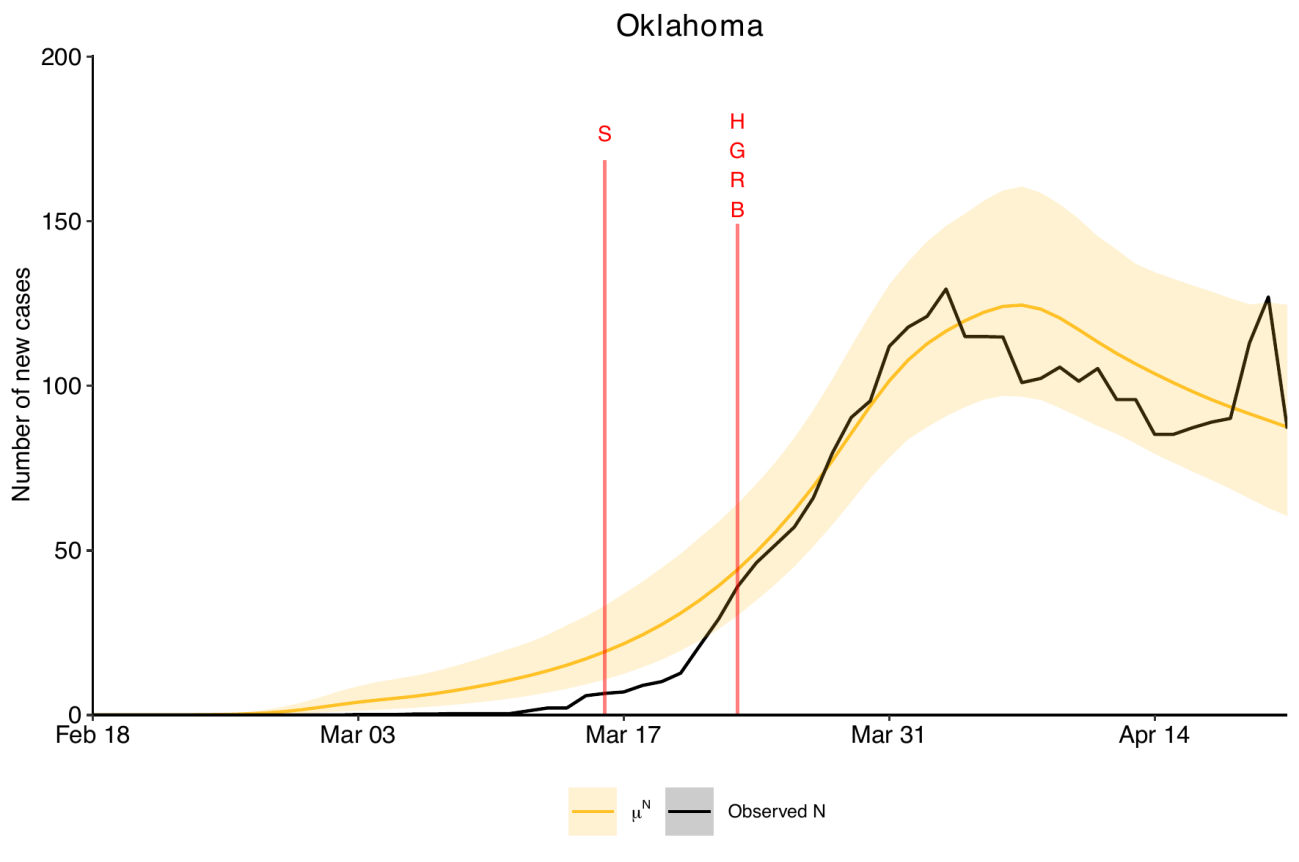

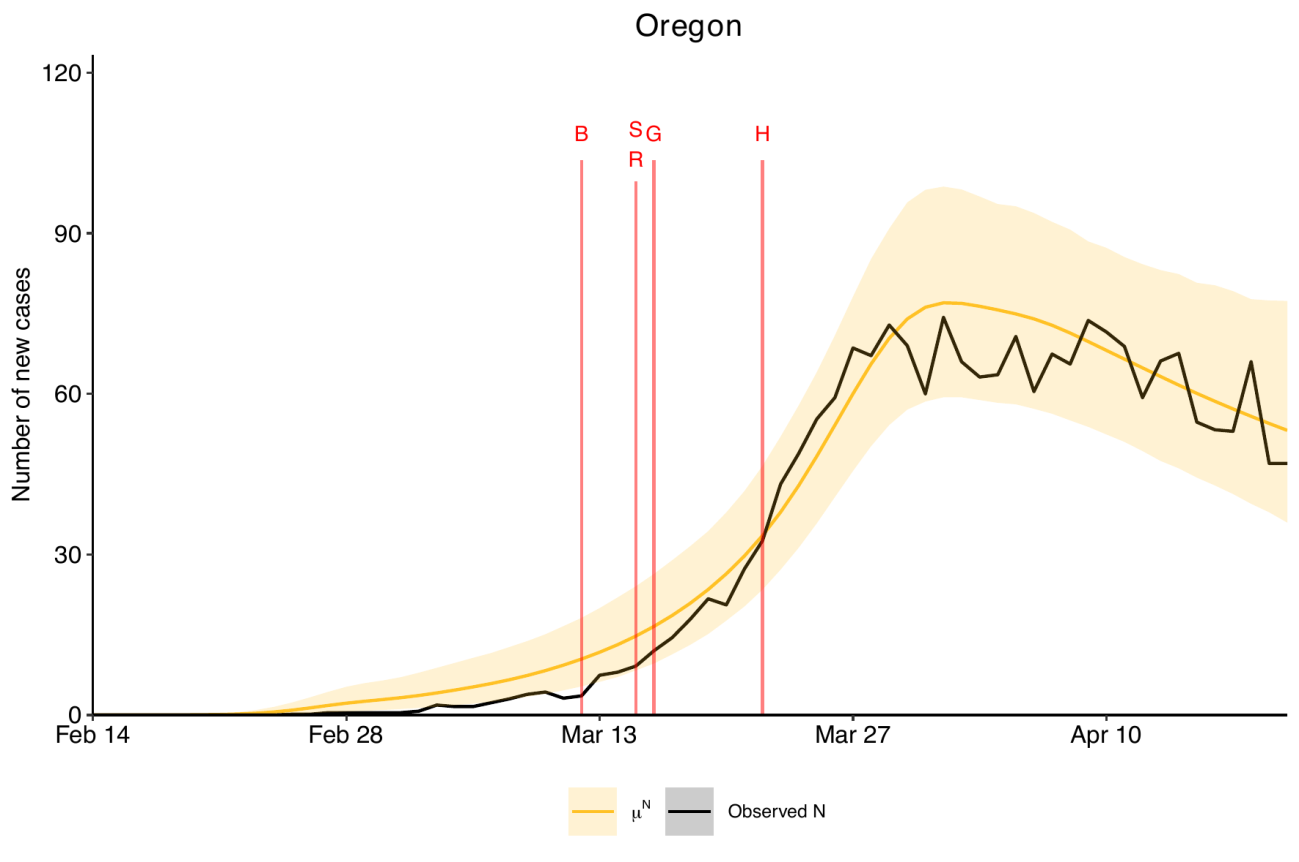

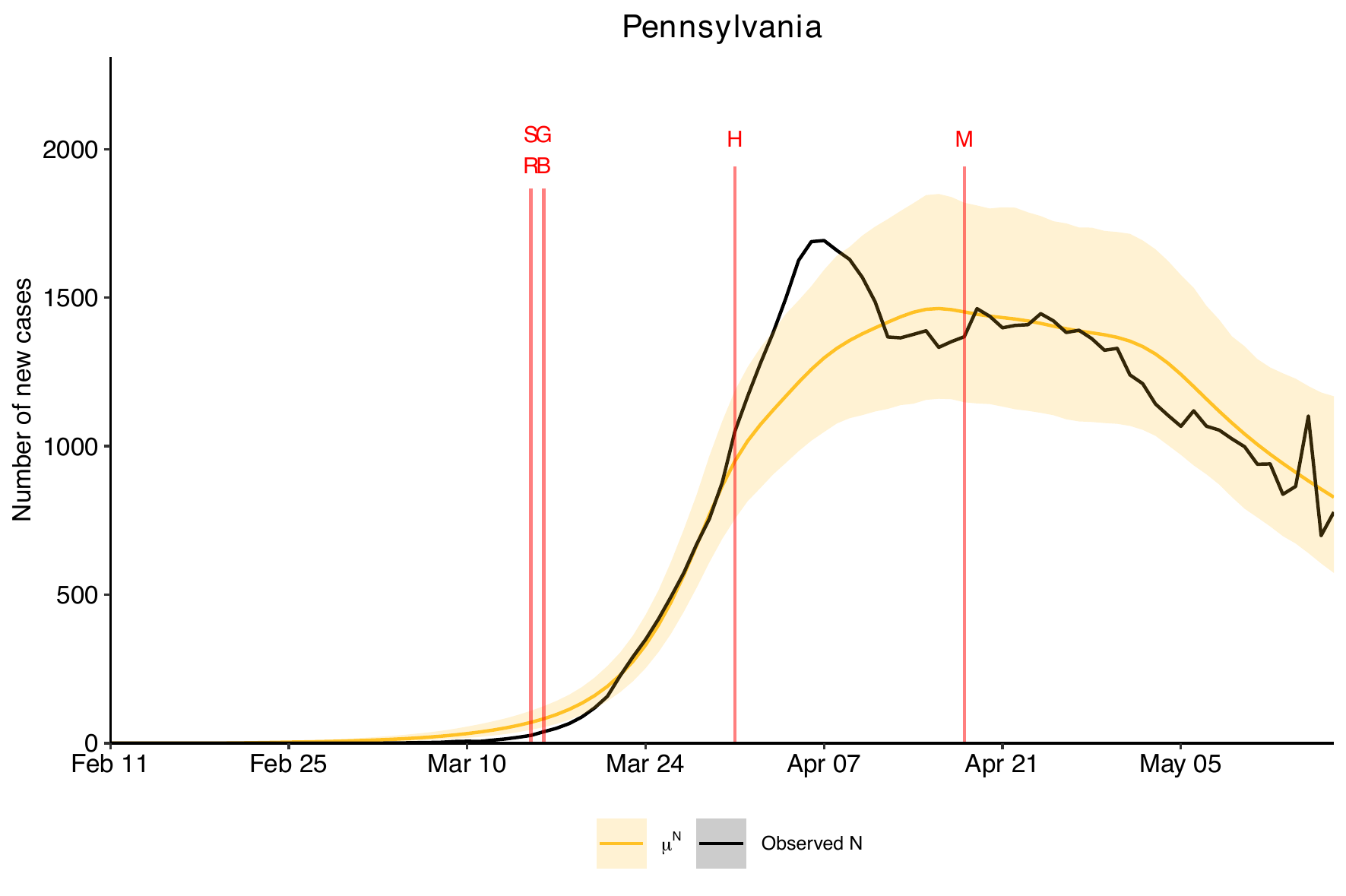

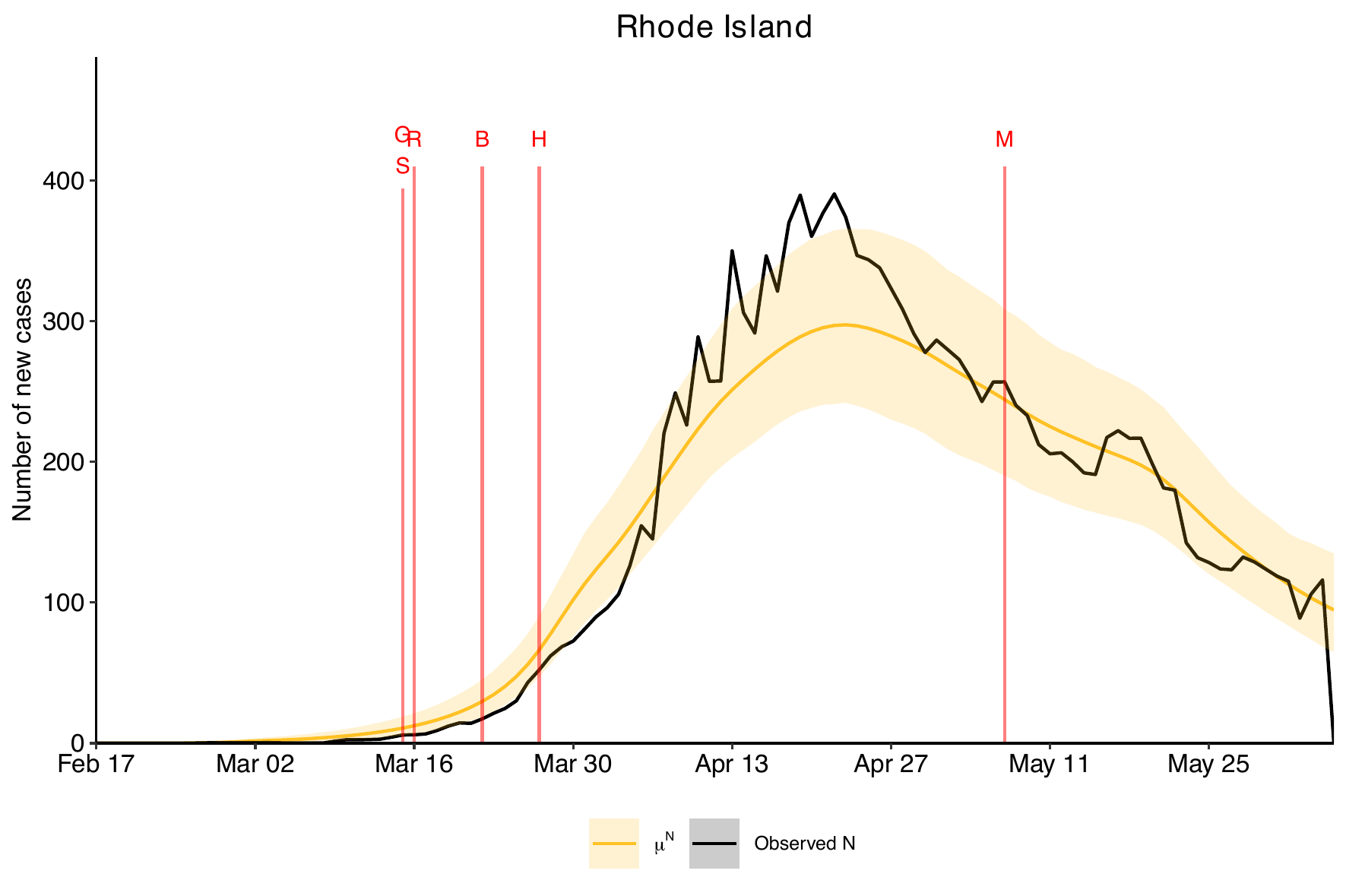

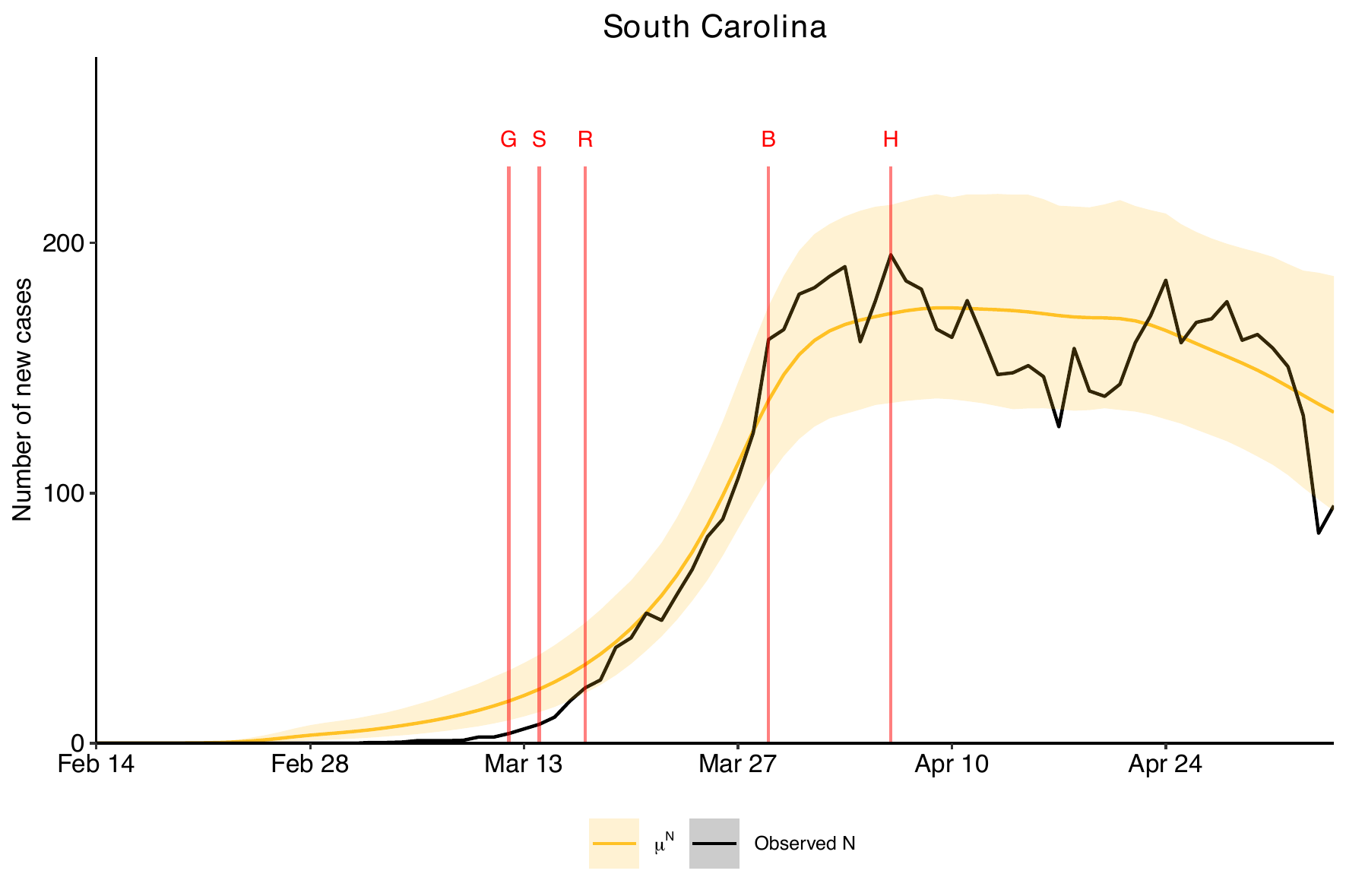

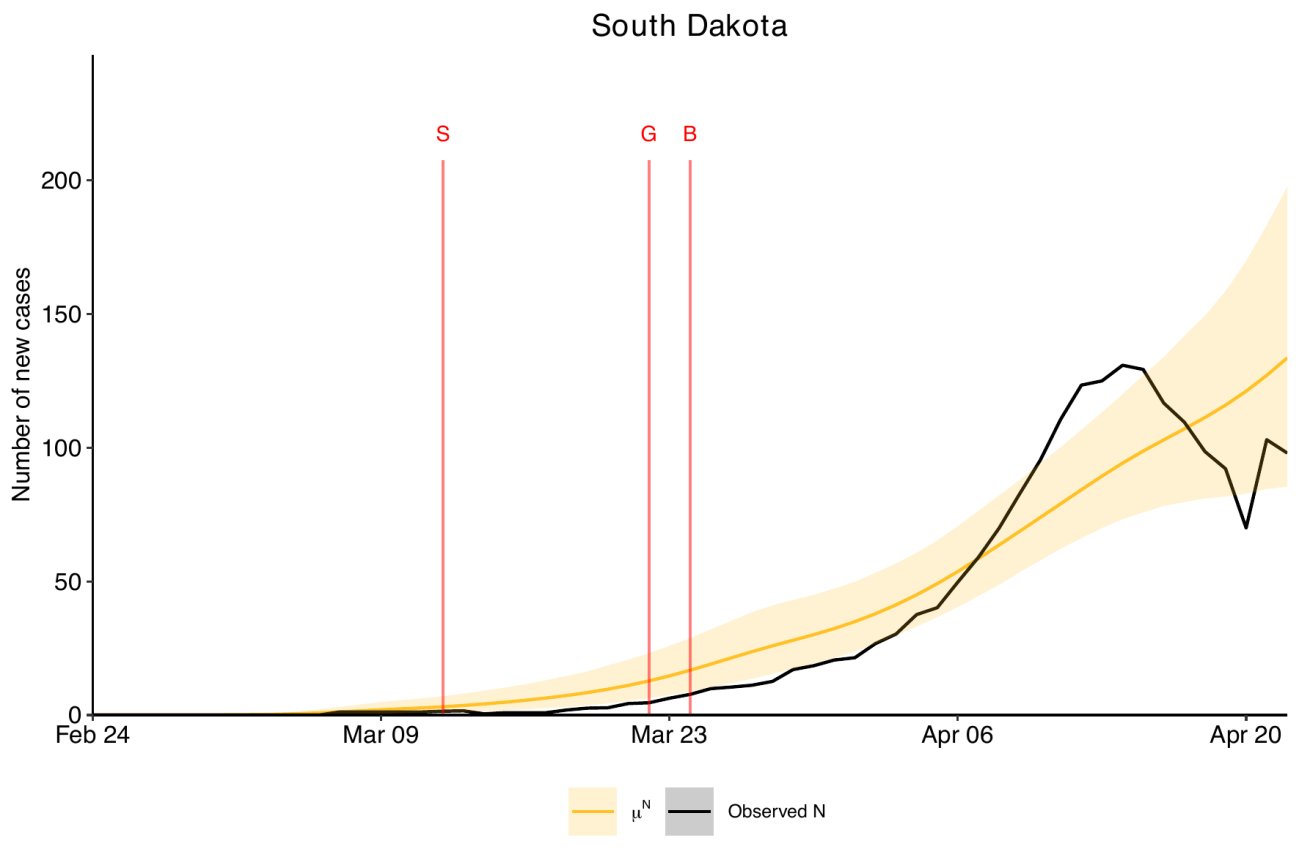

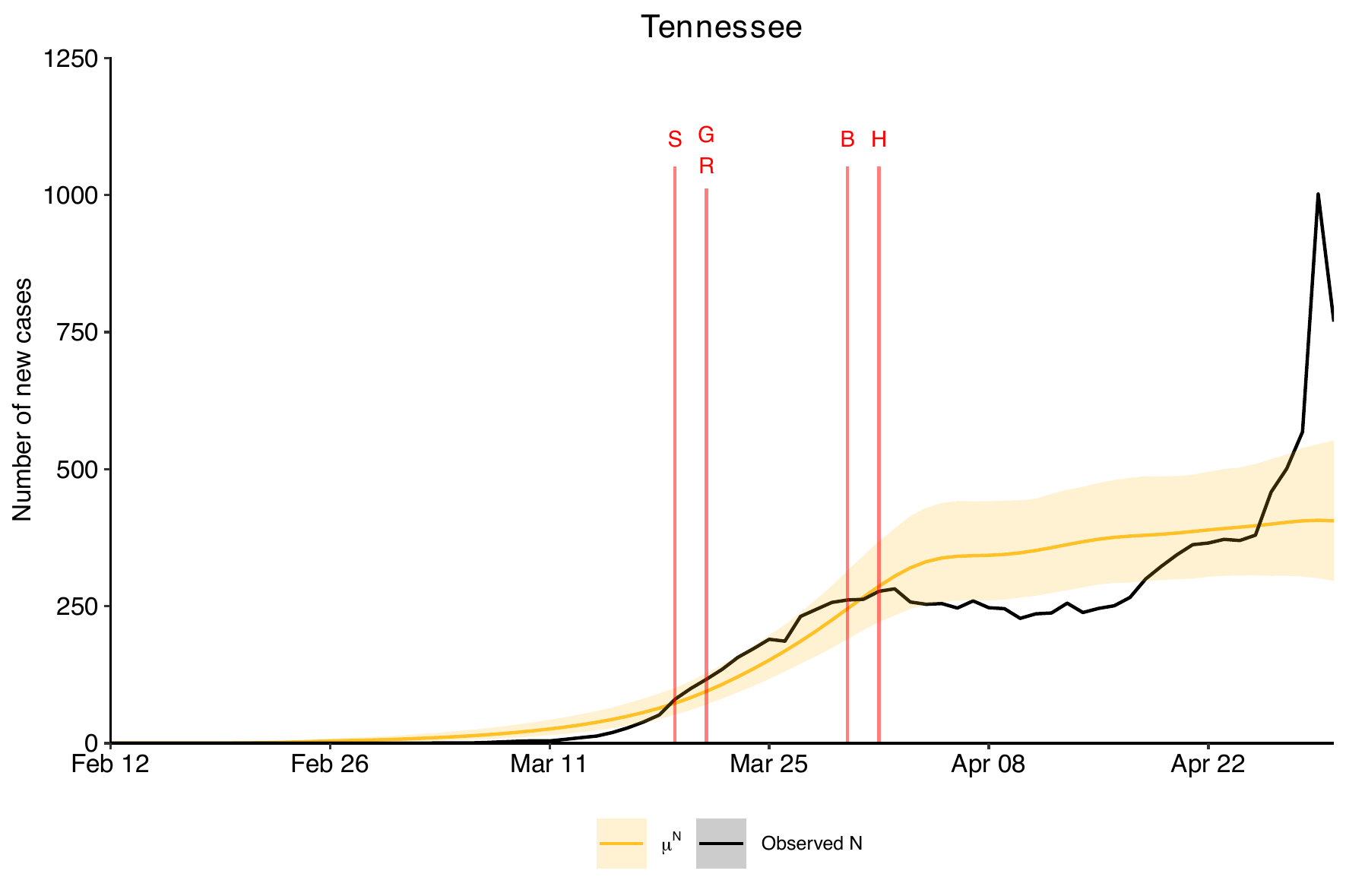

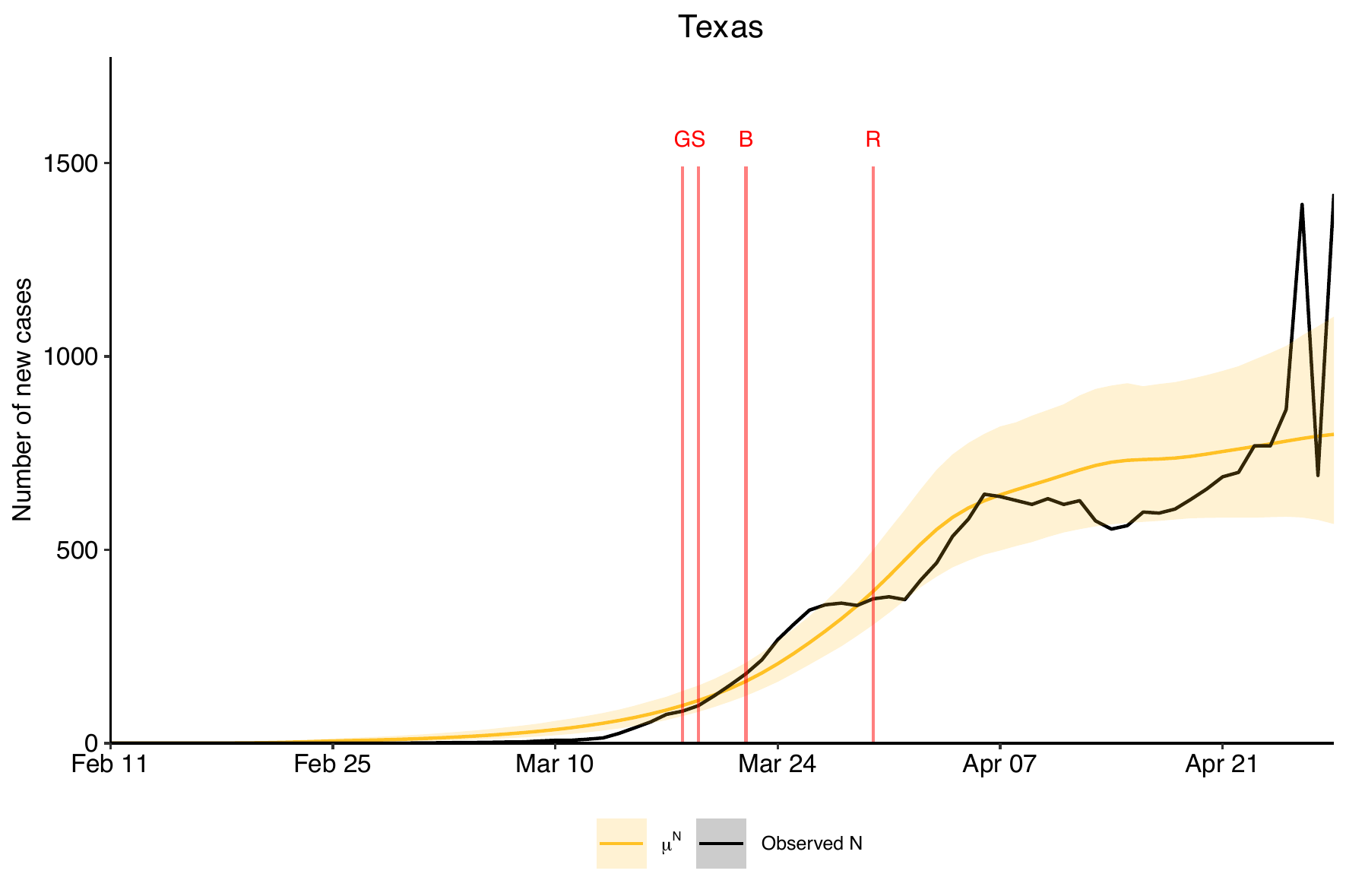

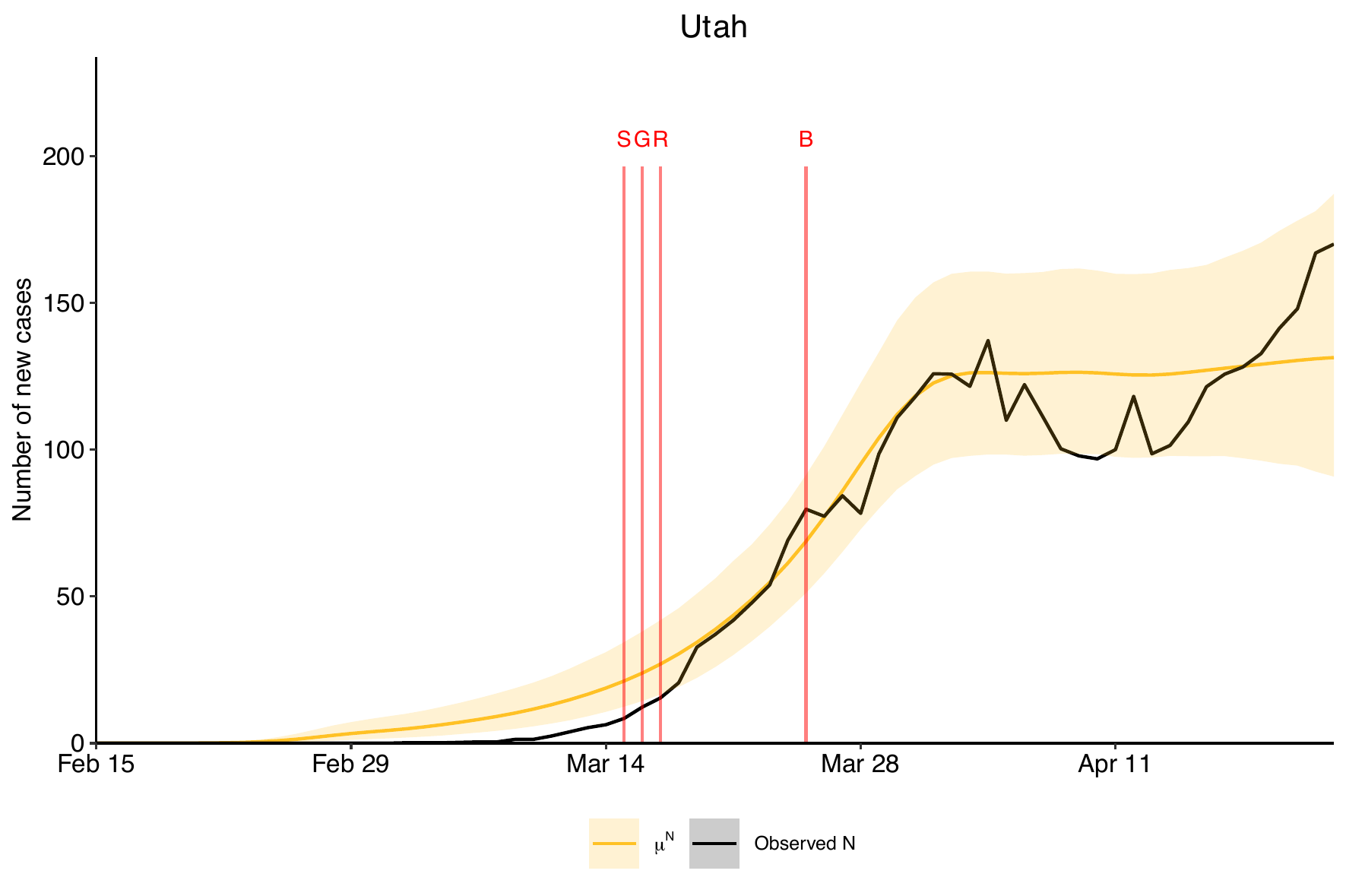

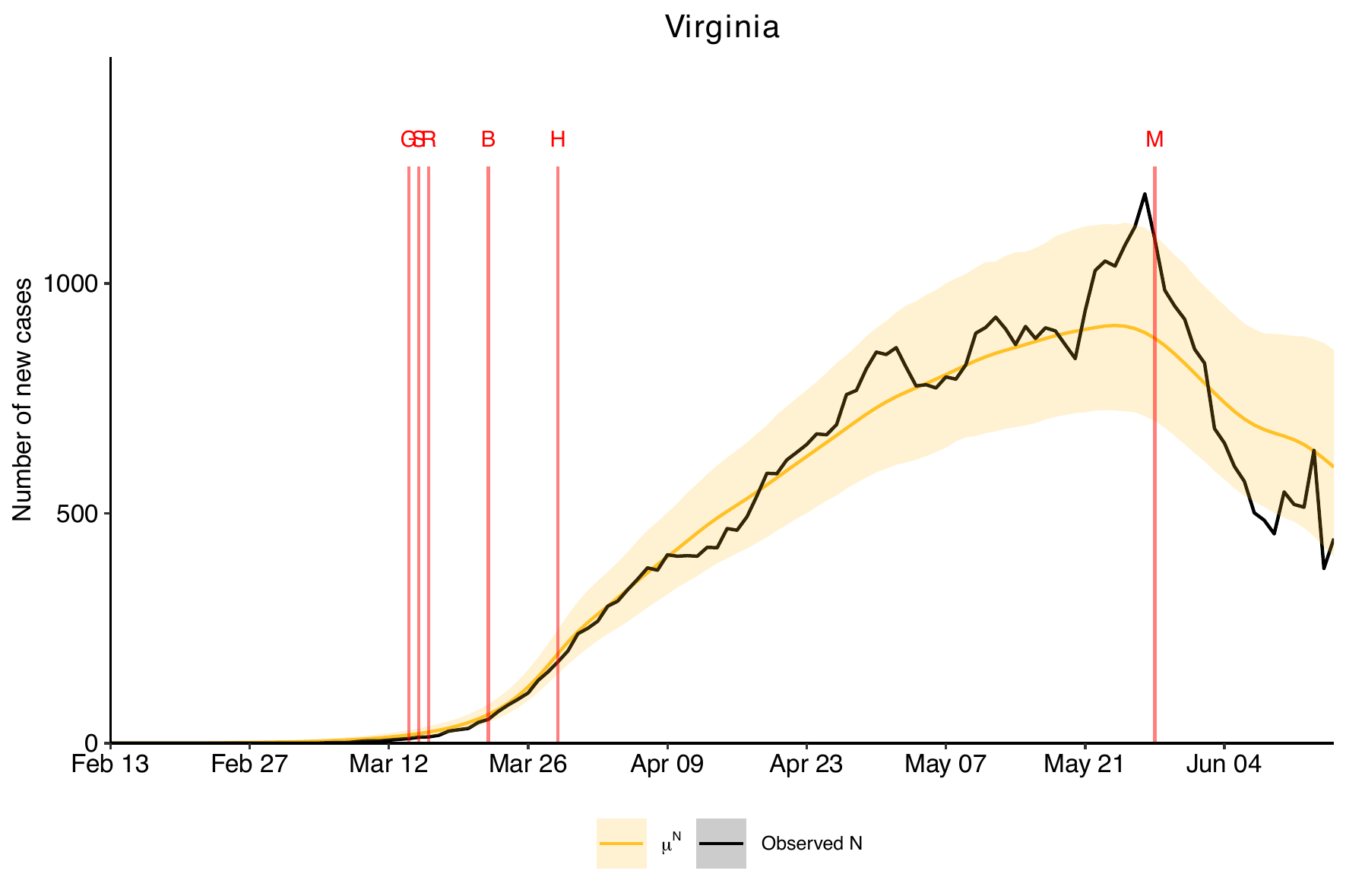

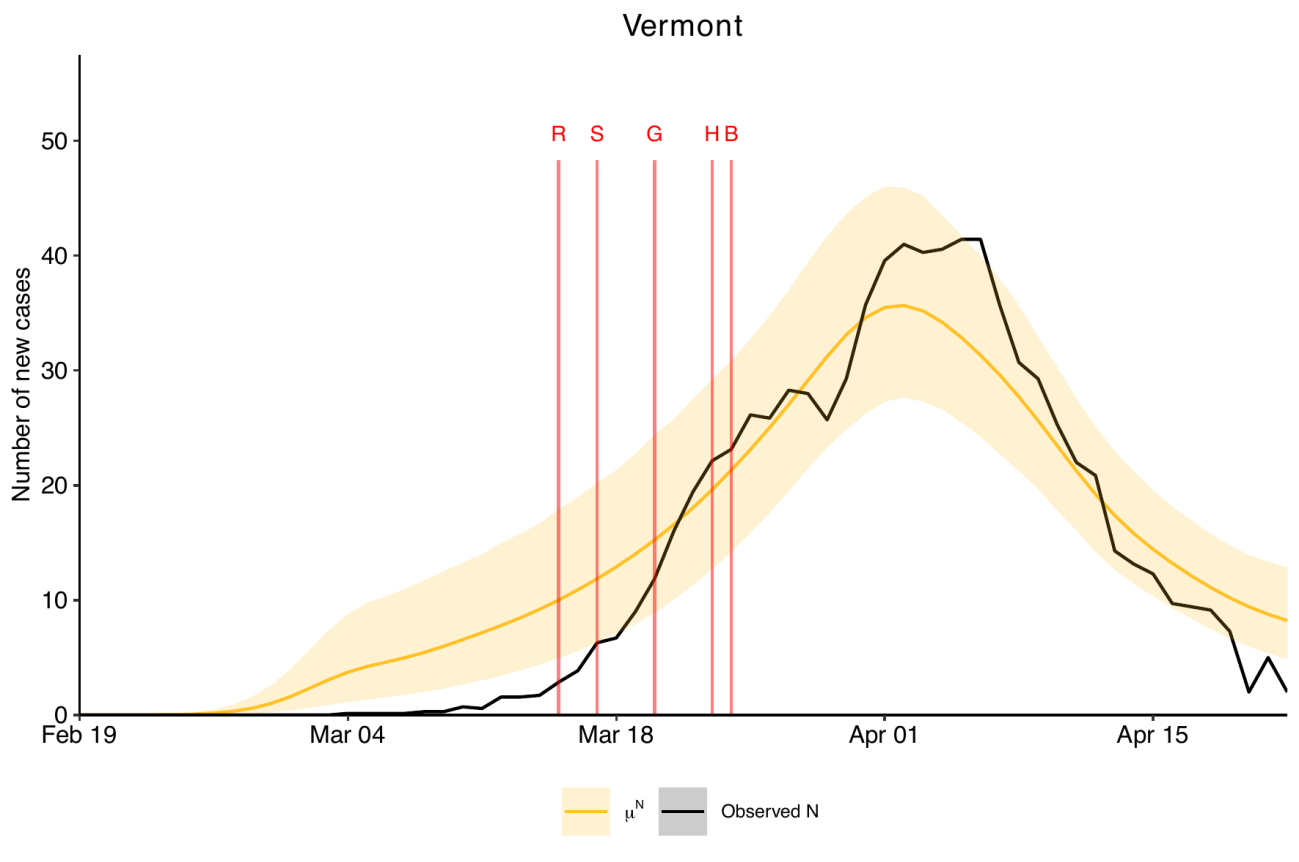

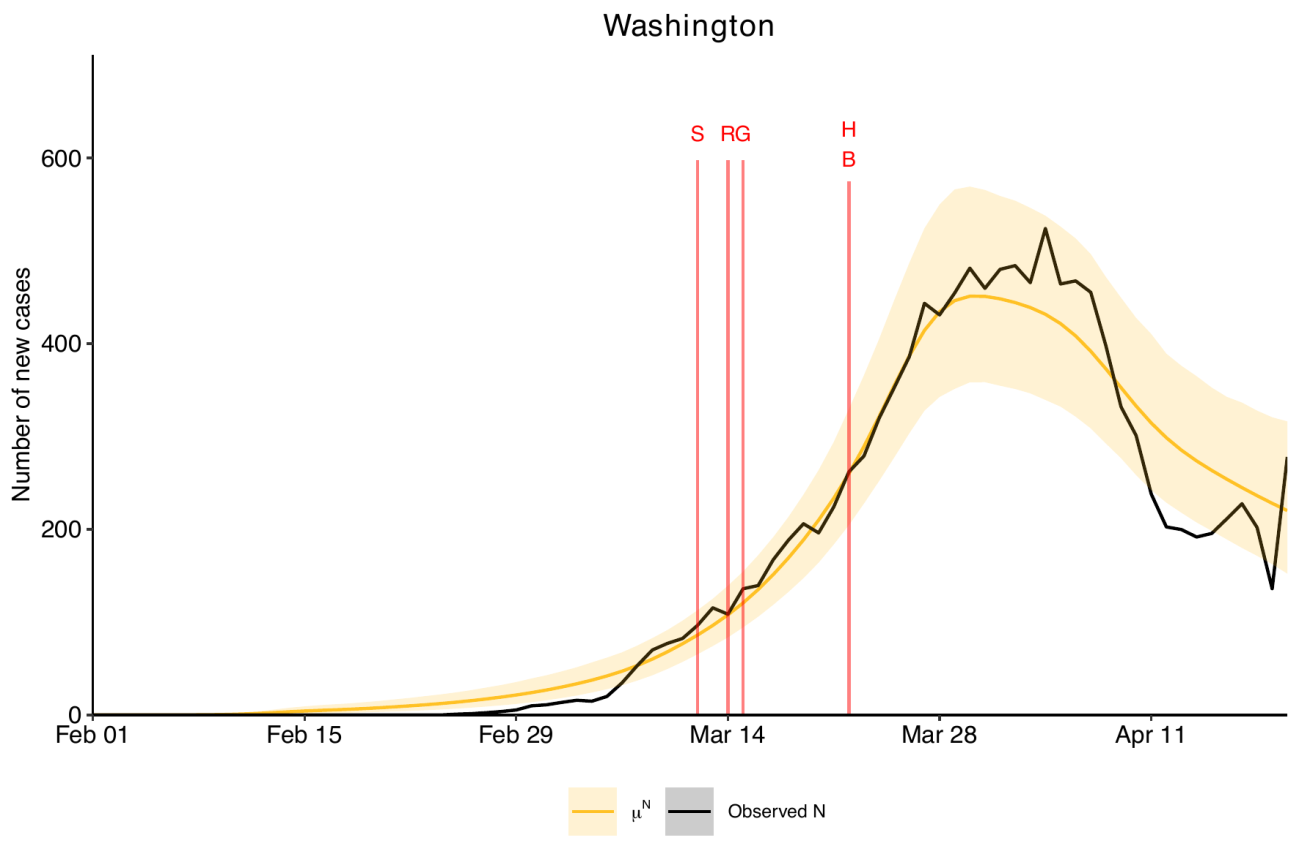


- **Figure List 4:** Advanced Prototypical Bayesian Hierarchical Model Expected Daily Number of True Infection Estimation Over the Study Period by States.

- **References for Methodology Details:**

1. Flaxman, S. et al. Estimating the effects of non-pharmaceutical interventions on COVID-19 in Europe. Nature 584, 257–261 (2020).
2. Verity, R. et al. Estimates of the severity of coronavirus disease 2019: a model-based analysis. Lancet Infect. Dis. 20, 669–677 (2020).
3. Ferguson, N. et al. Report 9: Impact of non-pharmaceutical interventions (NPIs) to reduce COVID19 mortality and healthcare demand.
4. Bi, Q. et al. Epidemiology and transmission of COVID-19 in 391 cases and 1286 of their close contacts in Shenzhen, China: a retrospective cohort study. Lancet Infect. Dis. 20, 911–919 (2020)
5. Ying, L., Gayle Albert, A., Annelies, W.-S. & Joacim, R. The reproductive number of COVID-19 is higher compared to SARS coronavirus.[J]. J Travel Med (2020).
6. Mishra, S. et al. Comparing the responses of the UK, Sweden and Denmark to COVID-19 using counterfactual modelling. Sci. Rep. 11, 16342 (2021).
7. Dong, E., Du, H. & Gardner, L. An interactive web-based dashboard to track COVID-19 in real time. Lancet Infect. Dis. 20, 533–534 (2020).
8. Banholzer, N. et al. Estimating the effects of non-pharmaceutical interventions on the number of new infections with COVID-19 during the first epidemic wave. PLoS ONE 16(6): e0252827. <https://doi.org/10.1371/journal.pone.0252827>
9. McAloon C, Collins A, Hunt K, Barber A, Byrne AW, Butler F, et al. Incubation period of COVID- 19: a rapid systematic review and meta-analysis of observational research. BMJ Open. 2020 4;10 (8):e 039652.
10. Cereda D, Tirani M, Rovida F, Demicheli V, Ajelli M, Poletti P, et al. The early phase of the COVID-19 outbreak in Lombardy, Italy. arXiv. 2020.
11. Lehtinen S, Ashcroft P, Bonhoeffer S. On the relationship between serial interval, infectiousness profile and generation time. medRxiv. 2020.
12. Ferretti L, Wymant C, Kendall M, Zhao L, Nurtay A, Abeler-Dörner L, et al. Quantifying SARS-CoV-2 transmission suggests epidemic control with digital contact tracing. Science. 2020; 368 (6491): eabb6936.

- **References for Summary Tables**

1. Azimi S.S., et al. Estimation of the Basic Reproduction Number of the COVID-19 Epidemic in Iran. *Med J Islam Repub Iran*. **34**: 95 (2020). doi: [10.34171/mjiri.34.95](https://doi.org/10.34171%2Fmjiri.34.95)
2. Banholzer N, van Weenen E, Lison A, Cenedese A, Seeliger A, Kratzwald B, et al. (2021) Estimating the effects of non-pharmaceutical interventions on the number of new infections with COVID-19 during the first epidemic wave. *PLoS ONE* 16(6): e0252827. <https://doi.org/10.1371/journal.pone.0252827>
3. Bo et al. Effectiveness of Non-Pharmaceutical Interventions on COVID-19 Transmission in 190 Countries from 23 January to 13 April 2020. *Int H Infect Dis.* **102**: 247-253 (2021) <https://doi.org/10.1016/j.ijid.2020.10.066>
4. Chernozhukov, V., Kasahara, H. & Schrimpf, P. Causal impact of masks, policies, behavior on early covid-19 pandemic in the U.S. *J. Econom.* **220**, 23–62 (2021).
5. Dreher N., et al. Policy Interventions, Social Distancing, and SARS-CoV-2 Transmission in the United States: A Retrospective State-Level Analysis. *Am J Med Sci.* 2021. **361**: 575-584. <https://doi.org/10.1016/j.amjms.2021.01.007>
6. Ebrahim, S. *et al.* Reduction of COVID-19 Incidence and Nonpharmacologic Interventions: Analysis Using a US County–Level Policy Data Set (Preprint). *Jmir preprint*. **24614** (2020) doi:10.2196/preprints.24614.
7. Esra R., et al. Evaluating the Impact of Non-Pharmaceutical Interventions for SARS-CoV-2 on a Global Scale. *MedRxiv*, 2020. doi: <https://doi.org/10.1101/2020.07.30.20164939>
8. Flaxman, S., Mishra, S., Gandy, A. *et al.* Estimating the effects of non-pharmaceutical interventions on COVID-19 in Europe. *Nature* **584**, 257–261 (2020). <https://doi.org/10.1038/s41586-020-2405-7>
9. Ivorra, B., Ferrández, M., Vela-Pérez, M., & Ramos, A. Mathematical modeling of the spread of the coronavirus disease 2019 (COVID-19) taking into account the undetected infections. The case of China. *Commun Nonlinear Sci Numer Simul*. Vol. **88**, 105303 (2020). doi:[10.1016/j.cnsns.2020.105303](https://doi.org/10.1016/j.cnsns.2020.105303)
10. Jalali, A. M. *et al.* Delayed Interventions, Low Compliance, and Health Disparities Amplified the Early Spread of COVID-19. *Medrixiv* (2020). doi:10.1101/2020.07.31.20165654.
11. Leffler C.T., et al. Association of Country-wide Coronavirus Mortality with Demographics, Testing, Lockdowns, and Public Wearing of Masks. *Am J Trop Med Hyg*. 2020. **103**: 2400-2411. <https://doi.org/10.4269/ajtmh.20-1015>
12. Li Y., et al. The Impact of Policy Measures on Human Mobility, COVID-19 Cases, and Mortality in the US: A Spatiotemporal Perspective. *Int J Environ Res Public Health.* 2021; **18**: 996. <https://doi.org/10.3390/ijerph18030996>
13. Mishra, S., Scott, J.A., Laydon, D.J.*et al.* Comparing the responses of the UK, Sweden and Denmark to COVID-19 using counterfactual modelling. *Sci Rep* **11,**16342 (2021). <https://doi.org/10.1038/s41598-021-95699-9>
14. Olney, A. M., Smith, J., Sen, S., Thomas, F. & Unwin, H. J. T. Estimating the Effect of Social Distancing Interventions on COVID-19 in the United States. *Am. J. Epidemiol.* **190**, 1504–1509 (2021).
15. Pozo-Martin F., et al. The Impact of Non-Pharmaceutical Interventions on COVID-19 Epidemic Growth in the OECD Member States. *Eur J Epidemiol.* 2021. <https://doi.org/10.1007/s10654-021-00766-0>
16. Sharma M., et al. Understanding the Effectiveness of Government Interventions Against the Resurgence of COVID-19 in Europe. *Nature Communications,* **12** : 5820. (2021) doi: [10.1038/s41467-021-26013-4](https://doi.org/10.1038%2Fs41467-021-26013-4)
17. White, L. & Pagano, M. A likelihood-based method for real-time estimation of the serial interval and reproductive number of an epidemic. *Stat Med*. Vol. **27** (16), 2999-3016 (2008). doi:[10.1002/sim.3136](https://doi.org/10.1002/sim.3136)
18. Zhang X. & Warner M.E. Covid-19 Policy Differences Across US States: Shutdowns, Reopening, and Mask Mandates. *Int J Environ Res Public Health*. **17**: 9520 (2020). <https://doi.org/10.3390/ijerph17249520>
19. Courtemanche et al. Strong Social Distancing Measures In The United States Reduced The COVID-19 Growth Rate. Health Affairs. VOL. 39, NO. 7 (2020).
    <https://doi.org/10.1377/hlthaff.2020.00608>
20. Brauner, J. M. et al. Inferring the effectiveness of government interventions against COVID-19. Science 371, eabd9338 (2021).
21. Flaxman, S. et al. Estimating the effects of non-pharmaceutical interventions on COVID-19 in Europe. Nature 584, 257–261 (2020).
22. Fountoulakis K.N., Fountoulakis N.K., Koupidis S.A., Prezerakos P.E. Factors determining different death rates because of the COVID-19 outbreak among countries. J Public Health, 42 (4) (2020)
23. N. Haug, L. Geyrhofer, A. Londei, E. Dervic, A. Desvars-Larrive, V. Loreto, *et al.* Ranking the effectiveness of worldwide COVID-19 government interventions. Nat Hum Behav, 4 (12) (2020)
24. Hunter P, Colón-González F, Brainard J. Impact of non-pharmaceutical interventions against COVID-19 in Europe: a quasi-experimental study. MedRxiv 2020. doi:10.1101/2020.05.01.20088260.
25. Islam N, Sharp SJ, Chowell G, Shabnam S, Kawachi I, Lacey B, et al. Physical distancing interventions and incidence of coronavirus disease 2019: natural experiment in 149 countries. BMJ 2020;370. doi:10.1136/bmj.m2743.
26. Koh WC, Naing L, Wong J. Estimating the impact of physical distancing measures in containing COVID-19: an empirical analysis. Int J Infect Dis 2020;100:42–9. doi:10.1016/j.ijid.2020.08.026.
27. Liu Y, Morgenstern C, Kelly J, Lowe R, Jit M. The impact of non-pharmaceutical interventions on SARS-CoV-2 transmission across 130 countries and territories. BMC Med 2021;19(1):40. doi:10.1186/s12916-020-01872-8.
28. Papadopoulos DI, Donkov I, Charitopoulos K. The impact of lockdown measures on COVID-19: a worldwide comparison. MedRxiv 2020. doi:10.1101/2020. 05.22.20106476.
29. Piovani D, Christodoulou MN, Hadjidemetriou A, Pantavou K, Zaza P, Bagos PG, et al. Effect of early application of social distancing interventions on COVID-19 mortality over the first pandemic wave: an analysis of longitudinal data from 37 countries. J Infect 2021;82(1):133–42. doi:10.1016/j.jinf.2020.11.033.
30. Stokes J, Turner AJ, Anselmi L, Morciano M, Hone T. The relative effects of nonpharmaceutical interventions on early COVID-19 mortality: natural experiment in 130 countries. MedRxiv 2020. doi:10.1101/2020.10.05.20206888
31. Wibbens PD, Koo WWY, McGahan AM. Which COVID policies are most effective? A Bayesian analysis of COVID-19 by jurisdiction. PLoS ONE 2020;15(12):e0244177. doi:10.1371/journal.pone.0244177.
